# Identification of candidate DNA methylation biomarkers related to Alzheimer’s disease risk by integrating genome and blood methylome data

**DOI:** 10.1038/s41398-023-02695-w

**Published:** 2023-12-13

**Authors:** Yanfa Sun, Jingjing Zhu, Yaohua Yang, Zichen Zhang, Hua Zhong, Guanghua Zeng, Dan Zhou, Richard S. Nowakowski, Jirong Long, Chong Wu, Lang Wu

**Affiliations:** 1 College of Life Science, Fujian Provincial Key Laboratory for the Prevention and Control of Animal Infectious Diseases and Biotechnology, Fujian Provincial Universities Key Laboratory of Preventive Veterinary Medicine and Biotechnology (Longyan University), Longyan University, Longyan, Fujian 364012 P. R. China; 2 Cancer Epidemiology Division, Population Sciences in the Pacific Program, University of Hawaii Cancer Center, University of Hawaii at Manoa, Honolulu, HI 96813 USA; 3 Center for Public Health Genomics, Department of Public Health Sciences, UVA Comprehensive Cancer Center, School of Medicine, University of Virginia, Charlottesville, VA 22093 USA; 4 Department of Biostatistics, The University of Texas MD Anderson Cancer Center, Houston, TX 77030 USA; 5 School of Public Health and the Second Affiliated Hospital, Zhejiang University School of Medicine, Hangzhou, Zhejiang 310058 P.R. China; 6 Department of Biomedical Sciences, Florida State University, Tallahassee, FL 32304 USA; 7 Division of Epidemiology, Department of Medicine, Vanderbilt Epidemiology Center, Vanderbilt-Ingram Cancer Center, Vanderbilt University Medical Center, Nashville, TN 37203 USA

**Keywords:** Diseases, Biomarkers, Epigenetics in the nervous system

## Abstract

Alzheimer disease (AD) is a common neurodegenerative disease with a late onset. It is critical to identify novel blood-based DNA methylation biomarkers to better understand the extent of the molecular pathways affected in AD. Two sets of blood DNA methylation genetic prediction models developed using different reference panels and modelling strategies were leveraged to evaluate associations of genetically predicted DNA methylation levels with AD risk in 111,326 (46,828 proxy) cases and 677,663 controls. A total of 1,168 cytosine-phosphate-guanine (CpG) sites showed a significant association with AD risk at a false discovery rate (FDR) < 0.05. Methylation levels of 196 CpG sites were correlated with expression levels of 130 adjacent genes in blood. Overall, 52 CpG sites of 32 genes showed consistent association directions for the methylation-gene expression-AD risk, including nine genes (*CNIH4*, *THUMPD3*, *SERPINB9*, *MTUS1*, *CISD1*, *FRAT2*, *CCDC88B*, *FES*, and *SSH*2) firstly reported as AD risk genes. Nine of 32 genes were enriched in dementia and AD disease categories (*P* values ranged from 1.85 × 10^-4^ to 7.46 × 10^-6^), and 19 genes in a neurological disease network (score = 54) were also observed. Our findings improve the understanding of genetics and etiology for AD.

## Introduction

As the most common form of neurodegenerative illness, Alzheimer’s disease (AD) remains the sixth leading cause of death in the United States and the fifth leading cause of death among Americans age ≥ 65 years [1]. AD is a slowly progressing neurodegenerative disorder, which can start 20-30 years before the appearance of the first clinical symptoms [2]. An improved understanding of AD etiology is critical to reduce the public health burden of this common disease.

Epidemiological studies provide strong support for a genetic predisposition to AD [3]. To date, genome-wide association studies (GWAS) have identified more than 56 gene loci [4] and transcriptome wide association studies (TWAS) [5–14] and splicing TWAS [15] have identified 29 genomic loci and over 280 genes associated with AD risk. However, together these variants and genes explain only a proportion of the familial relative risk of AD [16, 17]. One potential explanation is that for AD, some risk associated single nucleotide polymorphisms (SNPs) may regulate the expression of their target genes through influencing DNA methylation levels. As the most extensively investigated epigenetic marker, DNA methylation represents one kind of molecular regulatory mechanisms affecting gene expression that could further influence the risk of phenotypes [18]. It has been reported that changes of specific aberrant DNA methylation trigger alterations on the transcriptional levels of genes involved in the pathogenesis of AD [19]. Indeed, previous work reported that lower DNA methylation levels at *TREM2* intron 1 increased the AD risk because the lower methylation caused the higher *TREM2* mRNA expression in the leukocytes of AD patients than in healthy controls [20]. DNA methylation at *SORL1*, *SIRT1*, *UQCRC1*, *ABCA7*, *CNP*, and *DPYSL2* [21–24] have also been reported to influence AD through similar mechanisms. However, a comprehensive study to assess methylation markers that potentially influence AD risk through the DNA methylation-gene expression-AD risk pathway is largely lacking.

Herein, in this study, we leveraged two sets of DNA methylation prediction models built using large reference methylation datasets in blood (Framingham Heart Study (FHS) and Biobank-based integrative omics study (BIOS); up to 4008) with different modelling strategies [25–27], to evaluate the associations of genetically predicted DNA methylation levels with AD risk. For the association analyses with AD risk, we used the latest data from the AD GWAS involving 111,326 (46,828 proxy) cases and 677,663 controls of European ancestry from ten consortia/datasets, including the European Alzheimer & Dementia Biobank (EADB) datasets, the Genomic Research at Ace study (GR@ACE), the European Alzheimer’s Disease Initiative Consortium (EADI), Genetic and Environmental Risk in AD/Defining Genetic, Polygenic and Environmental Risk for Alzheimer’s Disease Consortium (GERAD/PERADES), the Norwegian DemGene Network (DemGene), Bonn Studies (Bonn), the Rotterdam study, the Copenhagen City Heart Study (CCHS), the Neocodex–Murcia study (NxC) and the UK Biobank (UKBB) [28].

## Materials and methods

### DNA methylation genetic prediction models

DNA methylation genetic prediction models. Two sets of DNA methylation prediction models established using different modelling strategies, FHS [25, 26] and BIOS [27], were used in the current study.

#### FHS models

The detailed information for FHS models has been described in previous studies [25, 26, 29, 30]. In brief, the individual level genome-wide genotyping and white blood cell DNA methylation data were obtained from the FHS Offspring Cohort (dbGaP accession numbers: phs000342 and phs000724) [25]. A total of 1595 genetically unrelated subjects of European descent with genetic and DNA methylation data were used to build FHS DNA methylation prediction models. Genomic DNA was genotyped using the Affymetrix 500 K array, and DNA methylation was measured using the Illumina HumanMethylation450 BeadChip. The genotype data were imputed to the Haplotype Reference Consortium reference panel [31]. SNPs meeting the following conditions were used to build DNA methylation prediction models: (1) high imputation quality (R^2^ ≥ 0.8), (2) minor allele frequency ≥0.05, included in the HapMap Phase 2 version, and (3) not strand ambiguous. For DNA methylation data, quality control and normalization were performed using the “minfi” package [32]. The quality control steps include: removing low-quality samples, excluding low-quality methylation probes, estimating cell-type composition, and calculating methylation beta values. The same scale methylation profile of each sample was first acquired using quantile normalization. A standard normal distribution of methylation values of each cytosine-phosphate-guanine (CpG) site was further obtained using rank normalization. The DNA methylation data was adjusted for age, sex, cell type composition variables, and top 10 principal components (PCs). DNA methylation level of each CpG site was predicted using the elastic net method as implemented in the “glmnet” package of R, with α = 0.5 [26, 33]. In short, we estimated the genetically regulated component of methylation levels for each CpG by including variants within a 2 MB window flanking the CpG site, inclusive. The square of the correlation between predicted and observed levels (R^2^) were generated to estimate the prediction performance of each of the CpG prediction models established.

#### BIOS models

BIOS DNA methylation prediction models were built using whole-blood methylation data from the BIOS Consortium involving 4008 samples (Illumina 450 K arrays). The detailed information of the model building has been described elsewhere [27, 34]. Briefly, in total, 881,977 unambiguous HapMap SNPs in the genetic data meeting the following criteria were retained: (1) minor allele frequency >5%, (2) minor allele count >10, and (3) imputation info score >0.8. The genotype data were also imputed to the Haplotype Reference Consortium reference panel [31]. For methylation quantitative trait loci (meQTL) analysis, linear regression on each SNP-CpG site pair closer than 250 kb was performed. At a false discovery rate (FDR) of 5% (*P* < 9.3 × 10^−5^), there were 151,729 CpG sites with a significant meQTL. For each CpG with a significant meQTL, a prediction model of methylation was established based on local SNPs within 250 kb using glmnet, which is a weighted linear combination of SNPs. We derived the unstandardized prediction models leveraging the original standardized models and standard deviation of variants in European populations of the 1000 Genomes Project data.

### Associations between predicted methylation levels and AD risk

Associations between genetically predicted DNA methylation levels and AD risk were analyzed using S-PrediXcan [33] by applying FHS and BIOS DNA methylation prediction models to summary statistics of AD GWAS. These summary data were generated from 111,326 (46,828 proxy) cases and 677,663 controls of European ancestry from ten consortia/datasets, including EADB, GR@ACE, EADI, GERAD/PERADES, DemGene, Bonn, the Rotterdam study, the CCHS study, NxC and the UKBB [28]. Instead of using the conventional approach of including clinically diagnosed AD alone, in this dataset both clinically confirmed and parental diagnoses based by-proxy phenotypes were included, which has been demonstrated to confer great value in substantially increasing statistical power [35]. It has been found that AD-by-proxy, based on parental diagnoses, shows quite strong genetic correlation with AD (r_g_ = 0.81) [35]. Detailed information on study participants, genotyping, and imputation methods have been included in the original GWAS paper [28]. In our association analysis, the FDR-corrected *P* value threshold of ≤ 0.05 was used to determine significant associations between genetically predicted DNA methylation levels and AD risk.

To further pinpoint the putative causal CpG sites for AD risk, fine-mapping of causal gene sets (FOCUS), as described elsewhere, was applied [36]. The two sets of blood methylation prediction models and results of main association analyses were used as inputs, and for each independent LD Block defined by LDetect [37], the posterior probability for each CpG site in the LD Block was outputted. For the FOCUS, putative causal CpG sites were prioritized by the default 90% credible CpG sites set.

### Functional annotation of AD-associated CpG sites

Functional annotation of the identified AD-associated CpG sites were conducted using ANNOVAR [38]. The CpG sites were annotated into one of 13 functional categories, including exonic, intronic, intergenic, upstream, 3′-UTR, 5′-UTR, ncRNA intronic, ncRNA exonic, splicing, downstream, upstream/ downstream, 5′UTR/3′-UTR, and exonic/splicing. We evaluated whether the identified AD-associated CpG sites were enriched in DNase I hypersensitive sites (DHSs) and loci overlapping with various histone modification types, including H3K27me3, H3K36me3, H3K4me3, H3K9me3, and H3K4me1 across different tissues and cell lines available in data of the Roadmap Epigenomics Project, the Encyclopedia of DNA Elements (ENCODE), and the BLUPRINT Epigenome, by using eFORGE v2.0 (https://eforge.altiusinstitute.org/) [39, 40]. The detail information for eFORGE has been described elsewhere [26].

### Correlations of AD-associated CpG sites with their nearby genes

For the AD-associated CpG sites, correlation analysis of their methylation and expression levels of their nearby genes was performed using data of 1367 unrelated European individuals from the FHS Offspring Cohort (dbGaP accession number: phs000363 and phs000724). We were not able to use the BIOS Consortium data due to a lack of access to the individual-level data. The detailed information about such DNA methylation and gene expression data has been described elsewhere [25, 26, 29, 30]. After adjusting for age, sex, cell type composition variables and top principal components (PCs), the correlation of the normalized methylation levels and expression levels of genes nearby the AD-associated CpG sites were calculated.

### Associations of potential target genes of CpG sites with AD risk

For identified putative target genes of AD-associated CpG sites, we further assessed associations of their predicted expression in blood with AD risk. Here two sets of gene expression prediction models were used, one established using a modified unified test for molecular signatures (UTMOST) strategy for the Genotype-Tissue Expression Project (GTEx) v8 dataset, and the other developed using LASSO strategy for the BIOS dataset. For the UTMOST models, transcriptome and genome data from the GTEx v8 were used to develop genetic imputation models for genes expressed in whole blood (*N* = 670). The cross-tissue UTMOST framework was used to build models [8]. SNPs within 1 Mb upstream and downstream of each gene of interest were considered as candidate predictors. It was shown that there is no significant difference in prediction quality from applying linkage disequilibrium (LD) pruning [41]. Therefore, LD-pruning (r^2^ = 0.9) was performed before model training to reduce the computational burden. In the joint-tissue prediction model, the effect sizes were estimated by minimizing the loss function with a logistic least absolute shrinkage and selection operator (LASSO) penalty on the columns (within-tissue effects) and a group-LASSO penalty on the rows (cross-tissue effects). The group penalty term implemented sharing of the information from SNP selection across all the tissues. Two hyperparameters, λ_1_ and λ_2_, for the within-tissue and cross-tissue penalization, were used as model optimization. For hyperparameter tuning, five-fold cross-validation was performed. A reliable estimate of the imputation performance was obtained by the modified model training approach. The original model training [8] was modified by unifying the hyperparameter pairs to avoid the overestimation of the prediction performance [42]. For the BIOS gene expression prediction models, a reference transcriptome dataset involving 3344 subjects was used. The detailed information for the establishment of this set of models has been described elsewhere [27]. For each of the 13,870 genes with a significant expression quantitative trait locus (eQTL), a prediction model was fitted in R with glmnet, to assess the potential predictive value of SNPs within 250 kb of the gene for gene expression. We used such sets of gene expression prediction models to estimate the associations between genetically predicted gene expression levels in blood and AD risk, by using the same AD GWAS data, involving 111,326 (46,828 proxy) cases and 677,663 controls as described above [43].

### Consistent direction of effect for the DNA methylation-gene expression-AD risk

To assess the possibility that the genetically predicted DNA methylation might putatively influence AD risk through regulating the expression of nearby target genes, associations showing consistent direction of effect for the DNA methylation-gene expression-AD risk were determined by assessing the associations between genetically predicted DNA methylation levels in blood and AD risk, associations between DNA methylation and gene expression in blood, and the associations between genetically predicted gene expression in blood and AD risk.

### Functional enrichment analysis

For the genes showing consistent directions of associations across DNA methylation, gene expression and AD risk, their top canonical pathways, disease and biological functions categories and networks were performed using Ingenuity pathway analysis (IPA) software (Qiagen Redwood City, Redwood City, USA, version summer release, July 2023).

## Results

### DNA methylation prediction models

#### FHS models

Of a total of 223,592 CpG sites for which we were able to develop DNA methylation prediction models using the FHS dataset, 81,360 showed a prediction performance (*R*^*2*^) of at least 0.01 (≥10% correlation between predicted and measured DNA methylation levels). Considering that DNA methylation measurement for the probe-binding sites tends to be unbiased [26, 42], we focused on 72,848 of those CpG sites for which there were no SNPs located within the probe-binding site. Such models were used for the association analyses between their predicted DNA methylation levels and AD risk.

#### BIOS models

As described elsewhere [27], leveraging the BIOS data, DNA methylation prediction models for 151,729 CpG sites were established, of which 103,354 showed a prediction performance (*R*^2^) of at least 0.01. For 93,442 of those CpG sites, there were no SNPs residing within the binding site. These models were also used for the association analyses.

Overall, models for a total of 104,102 unique CpG sites (either the FHS or BIOS models) were used in our association analyses for AD risk. Of them, for 62,188 CpG sites both sets of models were used; for 10,660 CpG sites only FHS models were used; and for the remaining 31,254 CpG sites only BIOS models were used (Supplementary Fig. S1).

### Association between genetically predicted methylation levels and AD risk

**Fig. 1:**
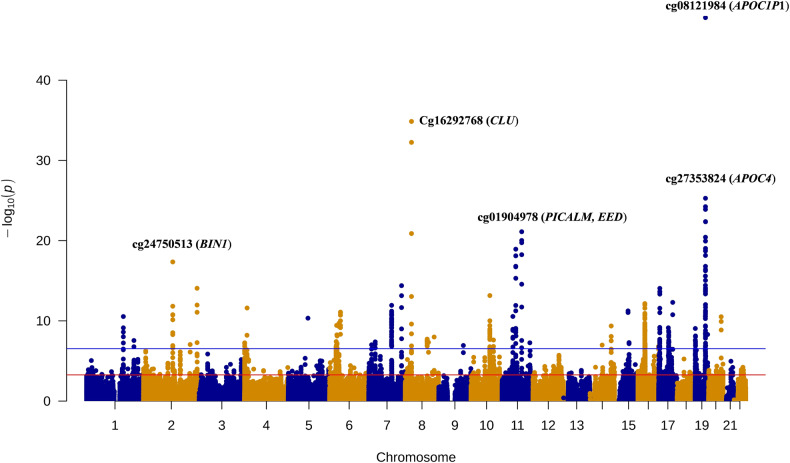
A Manhattan plot of the association results from the Alzheimer’s disease methylome-wide association study. The x axis represents the genomic position of the corresponding CpG site, and the y axis represents -log_10-_tansformed *P* value of the associations. Each dot represents the genetically predicted DNA methylation of one specific CpG site. The red line represents *P* = 5.55 × 10^-4^ for the false discovery rate significance threshold and blue line represents *P* = 3.01 × 10^-7^ for the Bonferroni correction threshold (0.05/166,290). The name of top five CpG sites and their nearby genes on four chromosomes were annotated.

Of the 104,102 CpG sites, genetically predicted DNA methylation of 1168 were associated with AD risk at the false discovery rate significance threshold (*FDR* ≤ 0.05), including 123 sites that met the more stringent Bonferroni correction threshold (*P* < 3.01 × 10^-7^, 0.05/166,290) (Supplementary Tables S1, S2 and Manhattan plot in Fig. 1), after removing 253 CpG sites in LD regions. Of the 1168 associated CpG sites, 750 showed significant associations using the FHS methylation prediction models and 827 showed associations using the BIOS prediction models. There were 409 CpG sites showing significant associations using both sets of prediction models **(**Supplementary Fig. S2). Reassuringly, the CpG sites showed the same association directions with AD risk for using the two sets of models (Supplementary Tables S1 and S2). Of those 1168 CpG sites associated with AD risk, 509 sites were located at more than 500 kb away from any known AD risk variants from GWAS studies (Supplementary Table S1). Of these 509 CpG sites, a positive association between predicted DNA methylation levels and AD risk was observed for 266 sites; conversely, an inverse association with AD risk was observed for 243 CpG sites. The remaining 659 CpG sites were located at known AD risk loci (Supplementary Table S2).

Based on analyses of the FOCUS, 26 CpG sites of 27 associations were further prioritized as putatively causal CpG sties for AD risk (Table 1). Of them, four CpG sites (cg09323728, cg18059933, cg26140475, and cg20555462) were located at more than 500 kb away from any known AD risk variants (Supplementary Table S1), involving genes *NDUFAF6*, *TRIB1*, *LINC00861*, and *UBASH3B*.

**Table 1:** Twenty-six putatively causal CpG sites for AD risk prioritized by FOCUS.

CpG^a^	Chr	Position	Classification	Closest gene	Model	TWAS *P* value after FDR^b^	FOCUS
cg22376361	2	127,815,133	exonic	*BIN1*	FHS	1.19 × 10^-7^	0.99
cg24750513	2	127,819,455	intronic	*BIN1*	BIOS	3.42 × 10^-14^	1.00
cg00436254	2	127,862,614	intronic	*BIN1*	FHS	7.49 × 10^-5^	0.99
cg14012546	2	233,981,788	intronic	*INPP5D*	BIOS	4.02 × 10^-11^	1.00
cg17634650	4	10,966,220	intergenic	*CLNK, MIR572*	BIOS	5.40 × 10^-6^	0.95
cg11284959	5	86,205,515	intergenic	*LINC02059*, *MIR4280*	BIOS	7.94 × 10^-8^	1.00
cg02130027	6	47,444,894	upstream	*CD2AP*	FHS	1.94 × 10^-8^	0.98
cg05908241	7	143,109,367	ncRNA_intronic	*EPHA1-AS1*	BIOS	2.53 × 10^-10^	1.00
cg13879655	8	27,450,777	intergenic	*EPHX2*, *CLU*	FHS	3.14 × 10^-10^	1.00
cg16292768	8	27,467,783	intronic	*CLU*	BIOS	1.16 × 10^-30^	1.00
cg09323728	8	95,962,352	intronic	*NDUFAF6*	FHS	1.96 × 10^-5^	1.00
cg18059933	8	95,962,463	intronic	*NDUFAF6*	FHS	2.22 × 10^-5^	1.00
cg26140475	8	126,525,558	intergenic	*TRIB1*, *LINC00861*	BIOS	8.31 × 10^-6^	1.00
cg14313833	9	107,666,037	UTR5	*ABCA1*	BIOS	6.80 × 10^-5^	0.99
cg24949488	10	98,064,362	exonic	*DNTT*	FHS	2.89 × 10^-4^	0.97
cg07180834	11	85,838,833	intergenic	*PICALM, EED*	BIOS	5.00 × 10^-15^	1.00
cg04895225	11	85,862,822	intergenic	*PICALM*, *EED*	BIOS	5.41 × 10^-9^	0.99
cg20555462	11	122,535,518	intronic	*UBASH3B*	BIOS	7.62 × 10^-4^	1.00
cg18696900	14	53,419,080	intergenic	*FERMT2*, *DDHD1*	BIOS	6.11 × 10^-5^	0.99
cg08898775	15	59,042,684	upstream	*ADAM10*	BIOS	2.11 × 10^-8^	1.00
					FHS	1.44 × 10^-8^	1.00
cg20401945	16	29,912,460	exonic	*ASPHD1*	FHS	4.61 × 10^-5^	0.99
cg10426084	17	1,640,472	intronic	*WDR81*	FHS	8.73 × 10^-5^	0.96
cg16837973	17	5,138,634	ncRNA_exonic	*LOC100130950*	FHS	9.11 × 10^-11^	1.00
cg27455331	17	47,338,178	intergenic	*FLJ40194*, *MIR6129*	BIOS	1.57 × 10^-4^	0.98
cg21657705	17	61,574,500	exonic	*ACE*	BIOS	3.40 × 10^-8^	1.00
cg20157577	17	61,780,203	UTR3	*STRADA*	BIOS	1.62 × 10^-4^	1.00

^a^*BIOS* Biobank-based Integrative Omics Studies, *Chr* chromosome, *CpG* CpG sites, *FHS* Framingham Heart Study, *kb* kilobase, *ncRNA* noncoding RNA, *UTR* untranslated region.

^b^TWAS associations with FDR-corrected *P* value < 0.05 considered significant.

Through the annotation using ANNOVAR [38], we compared the regional locations of the 1421 AD-associated CpG sites (including 253 CpG sites in LD regions) with the overall tested 104,102 CpG sites. We found that there were substantial inflation of the “exonic” and “ncRNA intronic” regions for the identified 1,421 AD-associated CpG sites (chi-square tests: 11.82% versus 7.53%, *P* = 1.74 × 10^-9^; 5.91% versus 7.57%, *P* = 4.31 ×10^-5^) (Supplementary Table S3). Conversely, there was deflation of the “intergenic” region (chi-square test: 17.52% versus 24.56%, *P* = 1.07 ×10^-9^) (Supplementary Table S3).

Based on annotation using eFORGE v2.0 (https://eforge.altiusinstitute.org/) [39, 40], positions of the 509 novel AD-associated CpG sites were overlapped with regions containing lysine 4 mono-methylated H3 histone (H3K4me1) markers across 36 of 39 cell types in the consolidated Roadmap Epigenomics Project, including blood (primary T cells from cord blood and peripheral blood, primary B cells, natural killer cells and monocytes from peripheral blood, and primary hematopoietic stem cells G-CSF-mobili) (Supplementary Fig. S3). These results indicated that our identified CpG sites associated with AD risk might be enriched in enhancers and transcriptional activation, further confirming the potential functional significance of our findings.

### Potential target genes of associated CpG sites

Whether DNA methylation of the associated CpG sites could influence flanking gene expression was investigated by analyzing the FHS data. Of 1168 AD-associated CpG sites, correlation analyses were performed for 1038 pairs of 892 CpG sites and their 485 flanking genes. Two hundred and five CpG site-gene pairs were observed to have statistically significant correlations at FDR *P*-value < 0.05, including 196 CpG sites and 130 genes (Supplementary Table S4). Of these 205 significant correlations, 131 were negative and 74 were positive. The associations between genetically predicted expression of these 130 genes in blood and AD risk were further evaluated using the same summary statistics of AD GWAS which consisted of 71,880 (proxy) cases and 383,378 (proxy) controls of European ancestry. Of these 130 genes, 46 showed an association with AD risk at FDR *P*-value < 0.05 (Supplementary Table S5).

To explore whether DNA methylation at associated CpG sites and their flanking genes have consistent effects on AD risk, we further compared directions of two-way associations of DNA methylation, gene expression and AD risk. We observed 52 consistent directions of associations across 51 CpG sites, 32 genes, and AD risk (Table 2). Taking the CpG sites cg09070378 and cg07356342 located at 3’ untranslated region (UTR3) of *NDUFS2* as an example, their DNA methylation levels were both positively associated with the expression of *NDUFS2* (coefficient = -0.10, *P* = 1.77 × 10^-3^, and coefficient = -0.07, *P* = 4.41 × 10^-2^, respectively); the genetically predicted DNA methylation of cg09070378 and cg07356342 were associated with an increased AD risk (OR = 1.10, *P* = 9.02 × 10^-7^, and OR = 1.10, *P* = 5.17 × 10^-8^ in BIOS model, respectively); and the predicted expression of *NDUFS2* was inversely associated with AD risk (OR = 0.85, *P* = 1.19 × 10^-3^ in BIOS model). The consistent DNA methylation-gene expression-AD associations observed for those 52 CpG sites suggested a potential mediating role of their neighboring gene expression on the associations between DNA methylation and AD risk. These 52 CpG sites and their 32 neighboring genes may affect AD risk, including previous GWAS [28, 35, 44–52] and/or TWAS studies [6–9] identified genes (*NDUFS2*, *FCER1G*, *BIN1*, *CD2AP*, *EPHA1*, *TP53INP1*, *CCDC6*, *TSPAN14*, *PLEKHA1*, *MS4A6A*, *CHRNE*, *SLC24A4*, *TBX6*, *YPEL3*, *TMEM106B*, *STX4*, and *CNN2*), six genes located within 500 kb of known AD susceptibility variants [28, 35, 45, 49] (*LRRFIP2*, *GAL3ST4*, *EPHX2*, *MAPK3*, *COASY*, and *MPO*) and nine novel genes reported in this study (*CNIH4*, *THUMPD3*, *SERPINB9*, *MTUS1*, *CISD1*, *FRAT2*, *CCDC88B*, *FES*, and *SSH*2).

**Table 2:** Fifty-two consistent directions of associations across DNA methylation, gene expression and AD risk.

CpG^a^	Chr	Position	DNA methylation and AD risk			DNA methylation and gene expression		Gene expression and AD risk			Associated gene
			Model	OR (95% CI)^b^	*P* value	Correlation coefficient	*P* value	Model	OR (95% CI)^2^	*P* value	
cg09070378	1	161,183,762	FHS	1.10 (1.07 ± 1.13)	9.02 × 10^-7^	-0.10	1.77 × 10^-3^	BIOS	0.85 (0.79 ± 0.91)	1.19× 10^-3^	*NDUFS2*
								UTMOST	0.87 (0.81 ± 0.93)	1.27× 10^-2^	
cg07356342	1	161,183,820	BIOS	1.10 (1.07 ± 1.14)	5.17 × 10^-8^	-0.07	4.41 × 10^-2^	BIOS	0.85 (0.79 ± 0.91)	1.19× 10^-3^	*NDUFS2*
			FHS	1.19 (1.12 ± 1.26)	7.89 × 10^-6^			UTMOST	0.87 (0.81 ± 0.93)	1.27× 10^-2^	
cg05659526	1	161,184,528	BIOS	1.14 (1.09 ± 1.20)	3.64 × 10^-5^	-0.13	2.44 × 10^-5^	UTMOST	0.80 (0.74 ± 0.85)	2.39× 10^-7^	*FCER1G*
								BIOS	0.89 (0.85 ± 0.92)	2.90× 10^-7^	
cg15595502	1	224,564,870	FHS	1.04 (1.02 ± 1.07)	1.30 × 10^-2^	-0.17	4.87 × 10^-9^	UTMOST	0.91 (0.87 ± 0.95)	2.01× 10^-3^	*CNIH4*
			BIOS	1.02 (1.01 ± 1.03)	1.84 × 10^-2^			BIOS	0.92 (0.88 ± 0.95)	1.36× 10^-3^	
cg08563189	2	127,780,654	FHS	1.40 (1.27 ± 1.54)	3.40 × 10^-8^	0.07	4.30 × 10^-2^	BIOS	1.28 (1.21 ± 1.34)	2.20× 10^-16^	*BIN1*
			BIOS	1.13 (1.09 ± 1.17)	3.62 × 10^-8^			UTMOST	1.28 (1.19 ± 1.38)	1.45× 10^-7^	
cg19153828	2	127,782,651	FHS	1.06 (1.03 ± 1.08)	1.17 × 10^-3^	0.09	4.31 × 10^-3^	BIOS	1.28 (1.21 ± 1.34)	2.20× 10^-16^	*BIN1*
			BIOS	1.02 (1.01 ± 1.04)	4.40 × 10^-3^			UTMOST	1.28 (1.19 ± 1.38)	1.45× 10^-7^	
cg19590598	2	127,782,813	FHS	1.05 (1.03 ± 1.08)	3.86 × 10^-4^	0.10	2.74 × 10^-3^	BIOS	1.28 (1.21 ± 1.34)	2.20× 10^-16^	*BIN1*
			BIOS	1.02 (1.01 ± 1.03)	6.54 × 10^-3^			UTMOST	1.28 (1.19 ± 1.38)	1.45× 10^-7^	
cg22376361	2	127,815,133	FHS	1.60 (1.39 ± 1.84)	1.19 × 10^-7^	0.10	3.51 × 10^-3^	BIOS	1.28 (1.21 ± 1.34)	2.20× 10-16	*BIN1*
								UTMOST	1.28 (1.19 ± 1.38)	1.45× 10^-7^	
cg00436254	2	127,862,614	FHS	1.10 (1.06 ± 1.13)	7.49 × 10^-5^	0.13	2.59 × 10^-5^	BIOS	1.28 (1.21 ± 1.34)	2.20× 10^-16^	*BIN1*
	2	127,862,614	BIOS	1.03 (1.01 ± 1.04)	1.24 × 10^-2^			UTMOST	1.28 (1.19 ± 1.38)	1.45× 10^-7^	
cg17763743	3	9,343,967	BIOS	0.97 (0.95 ± 0.99)	4.83 × 10^-2^	-0.12	1.59 × 10^-4^	BIOS	1.10 (1.04 ± 1.16)	4.18× 10^-2^	*THUMPD3*
cg06284479	3	37,173,546	FHS	1.09 (1.05 ± 1.13)	5.85 × 10^-3^	-0.11	9.22 × 10^-4^	BIOS	0.89 (0.83 ± 0.94)	1.64× 10^-2^	*LRRFIP2*
	3	37,173,546	BIOS	1.05 (1.02 ± 1.07)	1.39 × 10^-2^			UTMOST	0.89 (0.84 ± 0.94)	3.15× 10^-3^	
cg15934958	3	37,212,084	BIOS	1.04 (1.02 ± 1.07)	1.22 × 10^-2^	-0.12	5.76 × 10^-5^	BIOS	0.89 (0.83 ± 0.94)	1.64× 10^-2^	*LRRFIP2*
			FHS	1.05 (1.02 ± 1.07)	1.42 × 10^-2^			UTMOST	0.89 (0.84 ± 0.94)	3.15× 10^-3^	
cg23963071	6	2,901,712	BIOS	1.02 (1.01 ± 1.02)	3.83 × 10^-3^	-0.24	1.90 × 10^-14^	BIOS	0.91 (0.88 ± 0.95)	7.92× 10^-4^	*SERPINB9*
cg02130027	6	47,444,894	FHS	1.15 (1.11 ± 1.20)	1.94 × 10^-8^	0.15	4.81 × 10^-7^	UTMOST	1.94 (1.54 ± 2.42)	3.86× 10^-6^	*CD2AP*
			BIOS	1.07 (1.05 ± 1.09)	1.57 × 10^-7^						
cg20196966	6	47,445,060	FHS	1.25 (1.17 ± 1.33)	3.77 × 10^-8^	0.12	1.49 × 10^-4^	UTMOST	1.94 (1.54 ± 2.42)	3.86× 10^-6^	*CD2AP*
			BIOS	1.09 (1.06 ± 1.13)	8.85 × 10^-7^						
cg20172563	6	47,487,173	FHS	1.10 (1.07 ± 1.13)	2.18 × 10^-8^	0.23	1.903 × 10^-14^	UTMOST	1.94 (1.54 ± 2.42)	3.86× 10^-6^	*CD2AP*
			BIOS	1.04 (1.02 ± 1.05)	4.26 × 10^-6^						
cg06189038	7	99,767,134	BIOS	1.13 (1.06 ± 1.21)	1.95 × 10^-2^	-0.10	1.96 × 10^-3^	UTMOST	0.01 (0.01 ± 0.00)	6.33× 10^-5^	*GAL3ST4*
cg11291798	7	143,103,859	FHS	1.13 (1.06 ± 1.21)	2.97 × 10^-2^	-0.08	2.25 × 10^-2^	BIOS	0.83 (0.75 ± 0.92)	2.39× 10^-2^	*EPHA1*
cg14709253	8	17,519,419	BIOS	0.97 (0.95 ± 0.98)	9.98 × 10^-3^	0.19	5.27 × 10^-11^	UTMOST	0.76 (0.67 ± 0.86)	2.23× 10^-3^	*MTUS1*
								BIOS	0.93 (0.90 ± 0.96)	7.94× 10^-3^	
cg12548824	8	17,554,892	FHS	1.03 (1.02 ± 1.05)	2.52 × 10^-2^	-0.29	1.90 × 10^-14^	UTMOST	0.76 (0.67 ± 0.86)	2.23× 10^-3^	*MTUS1*
								BIOS	0.93 (0.90 ± 0.96)	7.94× 10^-3^	
cg01993952	8	17,554,904	FHS	1.04 (1.02 ± 1.06)	3.04 × 10^-2^	-0.20	2.21 × 10^-12^	UTMOST	0.76 (0.67 ± 0.86)	2.23× 10^-3^	*MTUS1*
								BIOS	0.93 (0.90 ± 0.96)	7.94× 10^-3^	
cg22099723	8	27,348,453	FHS	1.11 (1.06 ± 1.16)	3.29 × 10^-4^	0.08	1.28 × 10^-2^	BIOS	1.14 (1.06 ± 1.24)	4.70× 10^-2^	*EPHX2*
			BIOS	1.02 (1.01 ± 1.03)	3.66 × 10^-2^						
cg21666367	8	27,450,279	BIOS	1.06 (1.03 ± 1.08)	3.90 × 10^-4^	0.11	3.36 × 10^-4^	UTMOST	1.12 (1.06 ± 1.18)	3.15× 10^-3^	*EPHX2*
								BIOS	1.14 (1.06 ± 1.24)	4.70× 10^-2^	
cg24794833	8	27,450,748	FHS	0.81 (0.76 ± 0.87)	3.82 × 10^-6^	-0.10	2.78 × 10^-3^	UTMOST	1.12 (1.06 ± 1.18)	3.15× 10^-3^	*EPHX2*
								BIOS	1.14 (1.06 ± 1.24)	4.70× 10^-2^	
cg26343298	8	95,960,752	FHS	1.08 (1.05 ± 1.11)	1.81 × 10^-5^	-0.15	1.22 × 10^-6^	UTMOST	0.69 (0.61 ± 0.78)	4.81× 10^-6^	*TP53INP1*
			BIOS	1.04 (1.02 ± 1.05)	6.98 × 10^-5^						
cg16915659	10	60,032,665	BIOS	1.05 (1.02 ± 1.07)	8.16 × 10^-3^	0.16	7.43 × 10^-8^	BIOS	1.06 (1.03 ± 1.10)	4.04× 10^-3^	*CISD1*
								UTMOST	1.06 (1.03 ± 1.10)	4.26× 10^-3^	
cg15320596	10	61,604,738	BIOS	0.97 (0.96 ± 0.99)	2.78 × 10^-2^	0.10	2.37 × 10^-3^	BIOS	0.60 (0.48 ± 0.75)	1.35× 10^-3^	*CCDC6*
			FHS	0.96 (0.93 ± 0.98)	4.83 × 10^-2^			UTMOST	0.86 (0.79 ± 0.93)	2.25× 10^-2^	
cg23858360	10	82,213,490	BIOS	0.94 (0.92 ± 0.96)	1.62 × 10^-5^	0.08	2.42 × 10^-2^	UTMOST	0.76 (0.67 ± 0.87)	4.83× 10^-3^	*TSPAN14*
			FHS	0.90 (0.86 ± 0.94)	3.52 × 10^-4^						
cg16178415	10	82,265,445	FHS	1.09 (1.05 ± 1.13)	6.82 × 10^-4^	-0.13	4.10 × 10^-5^	UTMOST	0.76 (0.67 ± 0.87)	4.83× 10^-3^	*TSPAN14*
			BIOS	1.05 (1.03 ± 1.08)	1.37 × 10^-3^						
cg24590430	10	99,097,076	BIOS	1.02 (1.01 ± 1.03)	3.65 × 10^-2^	-0.10	3.31 × 10^-3^	BIOS	0.91 (0.87 ± 0.96)	2.47× 10^-2^	*FRAT2*
			FHS	1.03 (1.01 ± 1.05)	4.35 × 10^-2^			UTMOST	0.77 (0.67 ± 0.88)	1.65× 10^-2^	
cg00091760	10	124,131,072	BIOS	0.83 (0.77 ± 0.90)	1.00 × 10^-3^	-0.10	3.40 × 10^-3^	UTMOST	1.12 (1.05 ± 1.19)	3.34× 10^-2^	*PLEKHA1*
								BIOS	1.28 (1.14 ± 1.44)	3.11× 10^-3^	
cg04353769	11	59,951,557	BIOS	0.69 (0.64 ± 0.75)	1.22 × 10^-15^	-0.10	1.93 × 10^-3^	BIOS	1.65 (1.47 ± 1.85)	1.77× 10^-13^	*MS4A6A*
cg06881914	11	59,951,663	FHS	0.86 (0.83 ± 0.89)	6.25 × 10^-15^	-0.19	1.43 × 10^-10^	BIOS	1.65 (1.47 ± 1.85)	1.77× 10^-13^	*MS4A6A*
			BIOS	0.91 (0.89 ± 0.93)	2.76 × 10^-12^						
cg04422903	11	64,108,550	FHS	1.03 (1.02 ± 1.05)	4.42 × 10^-2^	-0.10	3.20 × 10^-3^	UTMOST	0.88 (0.83 ± 0.95)	2.25× 10^-2^	*CCDC88B*
cg01120308	11	85,780,971	BIOS	0.87 (0.82 ± 0.92)	3.61 × 10^-4^	-0.14	3.13 × 10^-6^	BIOS	1.11 (1.07 ± 1.15)	5.96× 10^-5^	*CHRNE*
								UTMOST	1.10 (1.07 ± 1.15)	3.21× 10^-5^	
cg11107966	14	92,927,875	FHS	1.05 (1.03 ± 1.07)	7.25 × 10^-6^	-0.12	7.72 × 10^-5^	BIOS	0.92 (0.89 ± 0.95)	1.01× 10^-4^	*SLC24A4*
			BIOS	1.02 (1.01 ± 1.03)	1.57 × 10^-4^			UTMOST	0.82 (0.78 ± 0.86)	2.05× 10^-11^	
cg14021523	14	92,959,873	FHS	1.13 (1.06 ± 1.21)	2.26 × 10^-2^	-0.14	1.29 × 10^-6^	BIOS	0.92 (0.89 ± 0.95)	1.01× 10^-4^	*SLC24A4*
								UTMOST	0.82 (0.78 ± 0.86)	2.05× 10^-11^	
cg05200313	14	92,960,827	BIOS	1.14 (1.08 ± 1.21)	3.52 × 10^-4^	-0.16	2.36 × 10^-8^	BIOS	0.92 (0.89 ± 0.95)	1.01× 10^-4^	*SLC24A4*
			FHS	1.20 (1.11 ± 1.31)	3.62 × 10^-3^			UTMOST	0.82 (0.78 ± 0.86)	2.05× 10^-11^	
cg25647583	15	91,427,184	BIOS	0.93 (0.89 ± 0.97)	4.95 × 10^-2^	-0.26	1.90 × 10^-14^	UTMOST	1.19 (1.08 ± 1.30)	2.25× 10^-2^	*FES*
cg05676562	16	30,102,457	FHS	1.11 (1.07 ± 1.16)	2.59 × 10^-4^	-0.08	1.24 × 10^-2^	BIOS	0.94 (0.90 ± 0.97)	4.68× 10^-2^	*TBX6*
			BIOS	1.03 (1.01 ± 1.05)	2.63 × 10^-2^			UTMOST	0.93 (0.90 ± 0.96)	1.20× 10^-3^	
cg26709300	16	30,106,682	BIOS	0.95 (0.93 ± 0.97)	1.20 × 10^-5^	0.10	3.29 × 10^-3^	BIOS	0.82 (0.76 ± 0.87)	1.89× 10^-6^	*YPEL3*
								UTMOST	0.87 (0.83 ± 0.91)	8.15× 10^-7^	
cg02335376	16	30,124,880	BIOS	0.90 (0.86 ± 0.94)	2.66 × 10^-4^	-0.15	6.23 × 10^-7^	UTMOST	1.07 (1.03 ± 1.10)	1.23× 10^-2^	*MAPK3*
								BIOS	1.12 (1.06 ± 1.17)	1.19× 10^-3^	
cg08464513	16	30,136,024	FHS	0.88 (0.84 ± 0.93)	2.05 × 10^-4^	-0.11	8.45 × 10^-4^	UTMOST	1.07 (1.03 ± 1.10)	1.23× 10^-2^	*MAPK3*
			BIOS	0.93 (0.90 ± 0.96)	7.61 × 10^-4^			BIOS	1.12 (1.06 ± 1.17)	1.19× 10^-3^	
cg00249205	16	31,012,263	FHS	0.94 (0.91 ± 0.96)	5.85 × 10^-3^	0.15	3.64 × 10^-7^	UTMOST	0.81 (0.75 ± 0.88)	1.48× 10^-4^	*TMEM106B*
cg06233904	16	31,044,135	BIOS	1.10 (1.060 ± 1.14)	1.41 × 10^-3^	-0.08	1.42 × 10^-2^	BIOS	0.87 (0.81 ± 0.94)	1.65× 10^-2^	*STX4*
			FHS	1.24 (1.13 ± 1.37)	2.04 × 10^-3^						
cg07404961	16	31,049,270	FHS	0.85 (0.79 ± 0.91)	6.47 × 10^-4^	0.09	4.28 × 10^-3^	BIOS	0.87 (0.81 ± 0.94)	1.65× 10^-2^	*STX4*
			BIOS	0.94 (0.92 ± 0.97)	1.14 × 10^-3^						
cg19048010	17	28,084,996	FHS	1.07 (1.03 ± 1.11)	3.58 × 10^-2^	0.16	1.05 × 10^-7^	UTMOST	1.28 (1.11 ± 1.48)	3.62× 10^-2^	*SSH2*
cg00685795	17	40,713,781	FHS	1.12 (1.06 ± 1.20)	2.96 × 10^-2^	0.10	1.56 × 10^-3^	BIOS	1.44 (1.21 ± 1.73)	6.42× 10^-3^	*COASY*
			BIOS	1.06 (1.03 ± 1.01)	4.80 × 10^-2^						
cg04266202	17	56,352,895	FHS	1.16 (1.08 ± 1.24)	5.90 × 10^-3^	-0.19	1.43 × 10^-10^	BIOS	0.85 (0.77 ± 0.93)	2.73× 10^-2^	*MPO*
cg11015549	19	1,026,207	FHS	0.81 (0.72 ± 0.90)	2.56 × 10^-2^	-0.19	3.63 × 10^-11^	UTMOST	1.11 (1.05 ± 1.18)	3.31× 10^-2^	*CNN2*
cg04401876	19	45,445,449	BIOS	1.12 (1.07 ± 1.16)	7.38 × 10^-5^	0.08	1.71 × 10^-2^	UTMOST	1.12 (1.06 ± 1.18)	3.15× 10^-3^	*EPHX2*
								BIOS	1.14 (1.06 ± 1.24)	4.70× 10^-2^	

^a^*BIOS* Biobank-based Integrative Omics Studies, *Chr* chromosome, *CI* confidence interval, *CpG* CpG sites, *FHS* Framingham Heart Study, *OR* odds ratio per SD increase in genetically predicated DNA methylation level (continuous variable); *P* value: *P* value after false discovery rate (FDR) correction; *UTR* untranslated region.

^b^MetaXcan was used to estimate ORs, 95% CIs and *P* value. All statistical tests were two-sided.

It’s worth noting that for 14 genes, namely, *DGKQ*, *CR1*, *CPSF3*, *INPP5D*, *SERPINB1*, *MAFK*, *TMEM184* *A*, *PARP10*, *RNF43*, *UBASH3B*, *BCKDK*, *PVR*, *NKPD1*, and CASS4, there were inconsistent directions of associations for the DNA methylation-gene expression-AD risk pathway (Supplementary Table S5). Future work is needed to better understand their exact relationships.

### Functional enrichment analysis results

The top canonical pathways, disease and biological functions categories, and networks of the 32 genes that showed consistent directions of associations across DNA methylation, gene expression and AD risk were analyzed by IPA software. Ten genes, including *MPO*, *FES*, *MAPK3*, *CNN2*, *FCER1G*, *TSPAN14*, *COASY*, *CISD1*, *EPHA1* and STX4, were enriched in ten top canonical pathways (Supplementary Table S6). A total of 17 genes were enriched in ten top disease and biological functions categories (Supplementary Table S7). Nine of them (*TMEM106B*, *EPHA1*, *CD2AP*, *CHRNE*, *MPO*, *BIN1*, *MS4A6A*, *EPHX2*, and *MAPK3*) were enriched in neurological disease categories including four dementia and AD categories (*P* values ranged from 4.94 × 10^-2^ to 7.25 × 10^-5^). Thirty-two genes were enriched in two networks (Supplementary Fig. S4 and Supplementary Table S8). Nineteen genes (*BIN1*, *CCDC6*, *CD2AP*, *CNIH4*, *CNN2*, *COASY*, *EPHA1*, *EPHX2*, *FCER1G*, *FES*, *MAPK3*, *MS4A6A*, *MTUS1*, *PLEKHA1*, *STX4*, *TBX*6, *TMEM106B*, *TP53INP1*, and *YPEL3*) were in the top network related to neurological disease (Supplementary Fig. S4A and Supplementary Table S8). Some genes, such as *CD2AP*, *FCER1G*, *MAPK3*, and *EPHX2*, were in nodes or core nodes of this neurological disease network, indicating that the CpG sites and these target genes may influence AD development.

## Discussion

This is the first large-scale study to comprehensively evaluate associations of genetically predicted DNA methylation levels in blood with AD risk. Using two sets of DNA methylation prediction models developed using different reference datasets and modelling strategies, we identified 1186 CpG sites with predicted DNA methylation levels in blood to be associated with AD risk, including 509 located at novel loci. Through additional analyses involving gene expression, 52 CpG sites and their 32 nearby putative target genes have consistent effects influencing AD risk. Our study provided substantial information to improve the understanding of genetics and etiology for AD.

Previous work has supported that specific DNA methylation biomarkers could potentially be useful for AD risk assessment [18, 53]. For example, methylation at *COASY*, *BDNF*, *BER*, *HOXB6* and *BIN1* had been reported to be potentially associated with AD risk [18, 54–57]. However, some of the findings have not been entirely consistent [58], potential due to several limitations in conventional epidemiological studies, including selection bias, uncontrolled confounding, and reverse causation [26]. One strategy to reduce some of these biases is to use genetic instruments to assess the association between DNA methylation levels and AD risk. Similar to a design of transcriptome-wide association study (TWAS) [41], a genetically determined proportion of DNA methylation levels is expected to be less susceptible to selection bias and reverse causation. We have conducted several such methylome-wide association studies (MWAS) and identified multiple candidate DNA methylation biomarkers for the risk of several diseases [25, 26, 29].

In our study, we used two sets of DNA methylation genetic prediction models to estimate the genetically predicted DNA methylation levels in blood. The fact that the identified associated CpG sites were suggested by both sets of models when available provided further assurance for the robustness of the associated methylation markers. Importantly, our design of using comprehensive methylation prediction models as instruments is more powerful than studies based on the single-meQTL approach [25, 26]. Our analyses leveraging large number of available cases and controls also provide substantial higher power than studies evaluating directly measured methylation levels in relatively smaller samples. As a comparison, for example, a previous study for AD risk evaluating directly measured methylation levels in 120 LOAD patients and 115 controls only had an a priori power to detect differences of about 5% in mean methylation levels for the six genes under investigation, and there were no significant findings probably due to the low statistical power [58].

Several potential limitations need to be considered for appropriate interpretation of our findings. Similar to results from TWAS, the associations observed in our analyses focusing on CpG sites are also vulnerable to confounding due to pleiotropy and co-localization of genetic signals [26]. Correlated total methylation levels across CpG sites, correlated predicted DNA methylation across CpG sites, as well as shared genetic variants between DNA methylation genetic prediction models, could all lead to spurious associations in our analyses [26, 59]. When faced with two correlated predictors, regularized regression models will randomly down weight one of them, which may be the true causal variant [26].

Despite these potential limitations, our study has several potential implications. Frist, our study can help fill the gap for systematic methylation analysis of AD risk which can provide insights in the etiology of AD [60]. DNA methylation (of CpG sites) can be inherited [61] and plays a key role in regulating gene expression in a wide range of diseases and biological processes [62]. For AD, it has been shown that blood DNA methylation levels of specific CpG sites were changed in AD patients compared with controls [63, 64] and they could be associated with AD risk [65]. In our study, we identified 1186 CpG sites and 485 nearby target genes in blood tissue for AD, which may substantially improve our understanding of etiology of this disease.

Especially, we identified 52 CpG sites and their 32 nearby genes consistent associations of DNA methylation-gene expression-AD risk through integrating the methylation, gene expression and AD data. Most of these CpG sites target genes were known AD risk genes, such as *FCER1G*, *BIN1*, and *MS4A6A*. *FCER1G* encodes a high affinity IgE receptor that is involved in the innate immunity. A recent study showed that higher expression of this protein in microglia was related with pathologic inflammatory responses in brain as amyloid accumulation increased [66]. For blood tissue, previous research showed that *FCER1G* was down-regulated (Log_2_fold change =-0.02, FDR-adjusted *P* = 3.63 × 10^-3^) in AD (*n* = 49) patients compared with controls (*n* = 67) (GEO: GSE63060) [67]. In our study, we also detected an inverse association between predicted expression of *FCER1G* and AD risk (OR = 0.97, FDR-adjusted *P* = 7.57 × 10^-5^). These results are intriguing and warrant further investigation. *BIN1* encodes bridging integrator 1 and is a key susceptibility gene for LOAD [68]. The lower methylation levels of *BIN1* promoter in peripheral blood for Chinese subjective cognitive declining participants with significant AD biological characteristics were found when compared with controls based on analyses of the Chinese Alzheimer’s Biomarker and LifestylE (CABLE) database [68]. Another study showed that decreased methylation levels of three CpG sites in *BIN1* 3’ intergenic region were observed in 50 LOAD cases compared with 50 age and sex-matched controls [57]. In our study, higher predicted expression levels of *BIN1* and methylation levels of its’ intergenic or exonic region CpG sites (cg08563189, cg19153828, cg19590598 and cg22376361) were associated with increased AD risk. *MS4A6A*, a member of the membrane-spanning 4A gene family, encodes membrane-spanning 4-domains A6A. Previous studies have revealed that *MS4A6A* was a risk gene for AD [69–71]. Previous investigation has also reported that *MS4A6A* transcripts were increased in blood tissue of AD patients compared with that of controls [71], which is consistent with findings of the present study. Moreover, we identified novel AD risk-associated CpG sites and their target genes (*CNIH4*, *THUMPD3*, *SERPINB9*, *MTUS1*, *CISD1*, *FRAT2*, *CCDC88B*, *FES*, and *SSH*2). Three target genes (*CNIH4*, *MTUS1*, and *FES*) were enriched in neurological disease-related network. The remaining six genes (*THUMPD3*, *SERPINB9*, *CISD1*, *FRAT2*, *CCDC88B*, and *SSH*2) were enriched in inflammatory response-related network, which was known as one of the pathological features of AD [72]. In the future, functional studies focusing on the implicated CpG sites and target genes are needed to better understand their exact roles in AD development. In the current work we focused on blood for DNA methylation prediction models. It is known that DNA methylation could be tissue-specific. It is unclear whether the DNA methylation markers identified in this study are also associated with AD risk when focusing on more relevant brain tissues. Future research in this area would be needed to identify brain-specific methylation markers relevant to AD risk.

In summary, in an integrative multi-omics study, we identified multiple CpG sites associated with AD risk and that 52 CpG sites might affect AD risk through regulating the expression of putative target genes. Our findings provide new insights into the etiology of AD risk.

### Supplementary information

Supplementary files

## Data Availability

The datasets of FHS used in this study are obtained from publicly available through dbGaP (www.ncbi.nlm.nih.gov/gap): dbGaP Study Accession: phs000342 and phs000724. The summary statistics of AD GWAS by Bellenguez et al. [35] can be downloaded from the European Bioinformatics Institute GWAS Catalog (https://www.ebi.ac.uk/gwas/) under accession no. GCST90027158. The analysis code used for data analysis is available on reasonable request from the corresponding author.
