# Supplementary material for: Identification of candidate DNA methylation biomarkers related to Alzheimer’s disease risk by integrating genome and blood methylome data
Source: Transl Psychiatry. 2023 Dec 13;13:387. doi: 10.1038/s41398-023-02695-w (PMC10719322; doi:10.1038/s41398-023-02695-w)
Supplement: Supplementary file 1 — Supplementary files [file 41398_2023_2695_MOESM1_ESM.docx]

**Identification of candidate** **DNA methylation biomarkers related to Alzheimer’s disease risk by integrating genome and blood methylome data**

**Supplementary files**

**Table S1.** One hundred and sixty-five DNA methylation markers in novel genomic regions for AD risk.

**Table S2.** Two hundred and eighty-seven DNA methylation markers associated with AD risk identified in genomic regions within 500 kb of reported for AD risk.

**Table S3.** Categories were annotated through ANNOVAR.

**Table S4.** Correlation between DNA methylation of identified associated CpG sites and expression of their flanking genes.

**Table S5.** Associations between genetically predicted mRNA expression levels of 16 candidate target genes of identified 35 CpG sites and AD risk.

**Table S6.** Top canonical pathways of ten genes that consistent association directions for the methylation-gene expression-AD risk pathway.

**Table S7.** Neurological disease categories of ten genes that consistent association directions for the methylation-gene expression-AD risk pathway.

**Table S8.** Network of ten genes that consistent association directions for the methylation-gene expression-AD risk pathway.

**Figure S1.** Venn diagram of CpG sites in both models.

**Figure S2.** Venn diagram of significant CpG sites in both models.

**Figure S3.** Enrichment of AD associated CpG sites in regions overlapping H3K4me1 markers from the consolidated Roadmap Epigenomics data.

**Figure S4.** Network of ten genes that consistent association directions for the methylation-gene expression-AD risk pathway.

**Table S1. Five hundred and nine DNA methylation markers associated with AD risk identified in genomic regions away from 500 kb of susceptibility variants reported for AD risk.**

| **CpG^1^** | **Chr** | **Position** | **Classification** | **Closest gene** | **Model** | **R^2 2^** | **Number**  **of SNPs**  **in model** | **OR (95% CI)^3^** | ***P* value** | ***P* value**  **after FDR^4^** | **Closest AD**  **risk SNP^5^** | **Distance**  **to risk**  **SNP (kb)^6^** |
| --- | --- | --- | --- | --- | --- | --- | --- | --- | --- | --- | --- | --- |
| cg00958217 | 1 | 6,681,584 | exonic | *PHF13* | FHS | 0.01 | 17 | 1.21 (1.09 ± 1.35) | 4.26 × 10^-4^ | 4.19 × 10^-2^ | rs113020870 | -1,899 |
| cg09246103 | 1 | 8,456,466 | intronic | *RERE* | BIOS | 0.01 | 7 | 0.87 (0.80 ± 0.93) | 1.25 × 10^-4^ | 1.76 × 10^-2^ | rs113020870 | 49,146 |
| cg00120948 | 1 | 8,484,417 | intronic | *RERE* | BIOS | 0.15 | 11 | 1.04 (1.02 ± 1.06) | 4.21 × 10^-4^ | 4.15 × 10^-2^ | rs113020870 | -1,808 |
| cg00120948 | 1 | 8,484,417 | intronic | *RERE* | FHS | 0.06 | 10 | 1.11 (1.05 ± 1.18) | 4.67 × 10^-4^ | 4.44 × 10^-2^ | rs113020870 | -3,948 |
| cg20822540 | 1 | 9,070,127 | intronic | *SLC2A7* | FHS | 0.26 | 155 | 1.04 (1.02 ± 1.07) | 1.48 × 10^-4^ | 2.01 × 10^-2^ | rs113020870 | -5,645 |
| cg19164175 | 1 | 20,666,689 | intronic | *VWA5B1* | FHS | 0.02 | 52 | 0.87 (0.81 ± 0.94) | 1.53 × 10^-4^ | 2.04 × 10^-2^ | rs113020870 | -1,159 |
| cg08539965 | 1 | 21,396,338 | intronic | *EIF4G3* | FHS | 0.05 | 28 | 0.88 (0.82 ± 0.94) | 2.00 × 10^-4^ | 2.47 × 10^-2^ | rs113020870 | 61,935 |
| cg11157611 | 1 | 21,499,906 | intronic | *EIF4G3* | BIOS | 0.02 | 11 | 0.88 (0.83 ± 0.93) | 8.98 × 10^-6^ | 2.33 × 10^-3^ | rs113020870 | -7,856 |
| cg08596618 | 1 | 24,275,885 | intergenic | *MIR378F, PNRC2* | FHS | 0.05 | 7 | 1.14 (1.07 ± 1.23) | 1.58 × 10^-4^ | 2.10 × 10^-2^ | rs113020870 | -4,704 |
| cg08596618 | 1 | 24,275,885 | intergenic | *MIR378F, PNRC2* | BIOS | 0.06 | 20 | 1.06 (1.03 ± 1.09) | 2.66 × 10^-4^ | 3.01 × 10^-2^ | rs113020870 | 29,056 |
| cg22358066 | 1 | 24,296,851 | UTR3 | *SRSF10* | FHS | 0.22 | 73 | 0.95 (0.93 ± 0.97) | 6.66 × 10^-5^ | 1.11 × 10^-2^ | rs113020870 | 23,662 |
| cg22358066 | 1 | 24,296,851 | UTR3 | *SRSF10* | BIOS | 0.36 | 49 | 0.99 (0.98 ± 0.99) | 1.76 × 10^-4^ | 2.26 × 10^-2^ | rs113020870 | -1,364 |
| cg23847017 | 1 | 28,764,854 | UTR5 | *PHACTR4* | FHS | 0.12 | 31 | 1.08 (1.04 ± 1.13) | 2.24 × 10^-4^ | 2.68 × 10^-2^ | rs113020870 | - |
| cg00956142 | 1 | 28,765,031 | intronic | *PHACTR4* | FHS | 0.11 | 19 | 1.08 (1.03 ± 1.12) | 4.40 × 10^-4^ | 4.27 × 10^-2^ | rs113020870 | -1,558 |
| cg26328951 | 1 | 28,906,514 | ncRNA_intronic | *SNHG12* | FHS | 0.01 | 14 | 0.82 (0.73 ± 0.92) | 4.69 × 10^-4^ | 4.45 × 10^-2^ | rs113020870 | -3,952 |
| cg16883141 | 1 | 29,508,888 | upstream | *SRSF4* | FHS | 0.04 | 58 | 0.90 (0.86 ± 0.95) | 2.55 × 10^-4^ | 2.93 × 10^-2^ | rs113020870 | 8,465 |
| cg06885782 | 1 | 41,248,209 | intergenic | *NFYC, KCNQ4* | BIOS | 0.31 | 51 | 1.02 (1.01 ± 1.03) | 1.61 × 10^-4^ | 2.12 × 10^-2^ | rs113020870 | -4,730 |
| cg25208677 | 1 | 67,521,487 | intergenic | *SLC35D1, C1orf141* | BIOS | 0.44 | 54 | 0.98 (0.97 ± 0.99) | 4.67 × 10^-4^ | 4.44 × 10^-2^ | rs141749679 | -3,948 |
| cg24474319 | 1 | 156,671,386 | intronic | *CRABP2* | BIOS | 0.02 | 24 | 0.92 (0.87 ± 0.96) | 3.96 × 10^-4^ | 3.95 × 10^-2^ | rs4575098 | -1,536 |
| cg05809481 | 1 | 156,696,467 | intronic | *ISG20L2* | FHS | 0.01 | 4 | 1.39 (1.17 ± 1.65) | 1.31 × 10^-4^ | 1.83 × 10^-2^ | rs4575098 | 18,488 |
| cg05809481 | 1 | 156,696,467 | intronic | *ISG20L2* | BIOS | 0.03 | 6 | 1.09 (1.04 ± 1.15) | 5.10 × 10^-4^ | 4.72 × 10^-2^ | rs4575098 | 957 |
| cg00392257 | 1 | 156,696,747 | exonic | *ISG20L2* | BIOS | 0.04 | 7 | 1.08 (1.04 ± 1.13) | 3.65 × 10^-4^ | 3.74 × 10^-2^ | rs4575098 | -1,064 |
| cg02833725 | 1 | 156,696,768 | exonic | *ISG20L2* | BIOS | 0.06 | 8 | 1.06 (1.03 ± 1.10) | 4.67 × 10^-4^ | 4.44 × 10^-2^ | rs4575098 | -3,948 |
| cg00346446 | 1 | 156,711,439 | upstream, downstream | *MRPL24, HDGF* | FHS | 0.07 | 16 | 1.11 (1.05 ± 1.17) | 9.21 × 10^-5^ | 1.41 × 10^-2^ | rs4575098 | 6,269 |
| cg00346446 | 1 | 156,711,439 | upstream, downstream | *MRPL24, HDGF* | BIOS | 0.10 | 9 | 1.05 (1.02 ± 1.08) | 1.54 × 10^-4^ | 2.04 × 10^-2^ | rs4575098 | -4,563 |
| cg09274826 | 1 | 156,718,357 | intronic | *HDGF* | FHS | 0.02 | 9 | 1.25 (1.1 ± 1.43) | 4.93 × 10^-4^ | 4.62 × 10^-2^ | rs4575098 | 1,127 |
| cg22808351 | 1 | 156,783,633 | intronic | *SH2D2A* | BIOS | 0.04 | 15 | 0.93 (0.90 ± 0.97) | 1.20 × 10^-4^ | 1.72 × 10^-2^ | rs4575098 | 49,146 |
| cg22808351 | 1 | 156,783,633 | intronic | *SH2D2A* | FHS | 0.02 | 17 | 0.84 (0.77 ± 0.92) | 2.36 × 10^-4^ | 2.77 × 10^-2^ | rs4575098 | 1,196 |
| cg25630380 | 1 | 156,784,869 | exonic | *SH2D2A* | BIOS | 0.03 | 3 | 1.10 (1.05 ± 1.15) | 1.13 × 10^-4^ | 1.64 × 10^-2^ | rs4575098 | -5,853 |
| cg25630380 | 1 | 156,784,869 | exonic | *SH2D2A* | FHS | 0.02 | 4 | 1.26 (1.12 ± 1.43) | 2.09 × 10^-4^ | 2.55 × 10^-2^ | rs4575098 | 6,371 |
| cg16438688 | 1 | 156,815,529 | exonic | *INSRR* | BIOS | 0.01 | 8 | 1.14 (1.06 ± 1.22) | 2.13 × 10^-4^ | 2.59 × 10^-2^ | rs4575098 | -9,592 |
| cg00008446 | 1 | 156,815,774 | exonic | *INSRR* | BIOS | 0.02 | 8 | 1.11 (1.05 ± 1.18) | 3.08 × 10^-4^ | 3.34 × 10^-2^ | rs4575098 | 8,893 |
| cg08474334 | 1 | 156,816,117 | upstream | *MARVELD3* | BIOS | 0.01 | 10 | 1.14 (1.07 ± 1.22) | 9.11 × 10^-5^ | 1.40 × 10^-2^ | rs4575098 | 6,269 |
| cg03950000 | 1 | 160,617,327 | upstream | *SLAMF1* | FHS | 0.01 | 34 | 0.84 (0.76 ± 0.93) | 4.34 × 10^-4^ | 4.24 × 10^-2^ | rs4575098 | -2,206 |
| cg18200075 | 1 | 161,706,293 | intergenic | *FCRLB, DUSP12* | FHS | 0.21 | 11 | 0.95 (0.92 ± 0.97) | 4.29 × 10^-5^ | 8.06 × 10^-3^ | rs4575098 | 10,881 |
| cg18200075 | 1 | 161,706,293 | intergenic | *FCRLB, DUSP12* | BIOS | 0.23 | 33 | 0.97 (0.96 ± 0.99) | 2.85 × 10^-4^ | 3.15 × 10^-2^ | rs4575098 | 6,516 |
| cg07969646 | 1 | 181,057,452 | upstream | *IER5* | BIOS | 0.04 | 20 | 1.08 (1.04 ± 1.12) | 9.67 × 10^-5^ | 1.46 × 10^-2^ | rs4575098 | 6,268 |
| cg12857074 | 1 | 200,975,162 | intronic | *KIF21B* | BIOS | 0.03 | 10 | 1.09 (1.04 ± 1.15) | 4.60 × 10^-4^ | 4.39 × 10^-2^ | rs6656401 | 576 |
| cg03936955 | 1 | 205,030,980 | intronic | *CNTN2* | FHS | 0.02 | 10 | 0.77 (0.68 ± 0.88) | 8.13 × 10^-5^ | 1.29 × 10^-2^ | rs6656401 | 6,288 |
| cg24450653 | 1 | 205,060,293 | intronic | *RBBP5* | BIOS | 0.02 | 12 | 0.90 (0.85 ± 0.95) | 2.36 × 10^-4^ | 2.77 × 10^-2^ | rs6656401 | -3,952 |
| cg22711218 | 1 | 205,158,908 | intronic | *DSTYK* | FHS | 0.03 | 24 | 0.88 (0.82 ± 0.94) | 4.89 × 10^-4^ | 4.60 × 10^-2^ | rs6656401 | -3,985 |
| cg00961792 | 1 | 207,120,407 | upstream | *PIGR* | FHS | 0.02 | 36 | 0.86 (0.79 ± 0.93) | 1.88 × 10^-4^ | 2.36 × 10^-2^ | rs6656401 | 16,666 |
| cg18883472 | 1 | 224,548,495 | intronic | *CNIH4* | FHS | 0.03 | 17 | 1.20 (1.11 ± 1.30) | 1.01 × 10^-5^ | 2.56 × 10^-3^ | rs679515 | 20,239 |
| cg18883472 | 1 | 224,548,495 | intronic | *CNIH4* | BIOS | 0.04 | 10 | 1.08 (1.04 ± 1.12) | 1.50 × 10^-5^ | 3.58 × 10^-3^ | rs679515 | 19,986 |
| cg15595502 | 1 | 224,564,870 | UTR3 | *CNIH4* | FHS | 0.29 | 82 | 1.04 (1.02 ± 1.07) | 8.19 × 10^-5^ | 1.30 × 10^-2^ | rs679515 | 6,282 |
| cg15595502 | 1 | 224,564,870 | UTR3 | *CNIH4* | BIOS | 0.29 | 39 | 1.02 (1.01 ± 1.03) | 1.32 × 10^-4^ | 1.84 × 10^-2^ | rs679515 | 18,488 |
| cg08707875 | 1 | 224,575,077 | UTR3 | *WDR26* | BIOS | 0.26 | 22 | 1.03 (1.02 ± 1.04) | 6.36 × 10^-6^ | 1.76 × 10^-3^ | rs679515 | -32,762 |
| cg08707875 | 1 | 224,575,077 | UTR3 | *WDR26* | FHS | 0.28 | 67 | 1.05 (1.03 ± 1.07) | 1.49 × 10^-5^ | 3.57 × 10^-3^ | rs679515 | 20,007 |
| cg07957995 | 1 | 224,644,750 | intronic | *CNIH3* | BIOS | 0.03 | 9 | 0.93 (0.90 ± 0.97) | 4.44 × 10^-4^ | 4.29 × 10^-2^ | rs679515 | -1,572 |
| cg11314684 | 1 | 244,006,288 | intronic | *AKT3* | BIOS | 0.01 | 6 | 0.87 (0.80 ± 0.94) | 5.38 × 10^-4^ | 4.91 × 10^-2^ | rs679515 | 1,424 |
| cg25542745 | 2 | 497,296 | intergenic | *LINC01874, LINC01875* | BIOS | 0.08 | 48 | 1.04 (1.02 ± 1.07) | 9.25 × 10^-5^ | 1.42 × 10^-2^ | rs72777026 | 6,269 |
| cg25542745 | 2 | 497,296 | intergenic | *LINC01874, LINC01875* | FHS | 0.03 | 5 | 1.19 (1.09 ± 1.30) | 1.47 × 10^-4^ | 1.99 × 10^-2^ | rs72777026 | -8,535 |
| cg02557922 | 2 | 595,312 | intergenic | *LOC105373352, TMEM18* | FHS | 0.03 | 14 | 1.16 (1.07 ± 1.25) | 2.84 × 10^-4^ | 3.15 × 10^-2^ | rs72777026 | 6,543 |
| cg05360477 | 2 | 12,959,313 | intergenic | *TRIB2, LOC100506474* | FHS | 0.03 | 23 | 0.85 (0.79 ± 0.93) | 2.75 × 10^-4^ | 3.07 × 10^-2^ | rs72777026 | 10,244 |
| cg23599110 | 2 | 12,969,272 | intergenic | *TRIB2, LOC100506474* | BIOS | 0.01 | 8 | 1.15 (1.07 ± 1.23) | 2.54 × 10^-4^ | 2.93 × 10^-2^ | rs72777026 | 18,909 |
| cg06269559 | 2 | 15,298,785 | intergenic | *LRATD1, NBAS* | BIOS | 0.25 | 56 | 1.02 (1.01 ± 1.04) | 4.11 × 10^-5^ | 7.80 × 10^-3^ | rs72777026 | 10,895 |
| cg06269559 | 2 | 15,298,785 | intergenic | *LRATD1, NBAS* | FHS | 0.04 | 32 | 1.15 (1.07 ± 1.23) | 1.38 × 10^-4^ | 1.91 × 10^-2^ | rs72777026 | 11,822 |
| cg19276736 | 2 | 24,625,812 | intergenic | *ITSN2, NCOA1* | FHS | 0.08 | 36 | 0.93 (0.89 ± 0.97) | 4.27 × 10^-4^ | 4.19 × 10^-2^ | rs17020490 | -1,901 |
| cg22647546 | 2 | 26,394,518 | intergenic | *RAB10, GAREM2* | FHS | 0.02 | 36 | 0.84 (0.76 ± 0.92) | 2.26 × 10^-4^ | 2.69 × 10^-2^ | rs17020490 | - |
| cg07177860 | 2 | 26,402,318 | intronic | *GAREM2* | BIOS | 0.12 | 15 | 0.96 (0.94 ± 0.98) | 4.40 × 10^-4^ | 4.27 × 10^-2^ | rs17020490 | -1,546 |
| cg25748958 | 2 | 43,672,652 | intronic | *THADA* | BIOS | 0.26 | 52 | 0.98 (0.97 ± 0.99) | 9.89 × 10^-5^ | 1.49 × 10^-2^ | rs17020490 | 6,261 |
| cg11659317 | 2 | 43,863,985 | upstream | *PLEKHH2* | FHS | 0.02 | 8 | 0.81 (0.72 ± 0.91) | 5.48 × 10^-4^ | 4.95 × 10^-2^ | rs115186657 | - |
| cg18186672 | 2 | 71,679,841 | upstream | *DYSF* | FHS | 0.06 | 28 | 0.90 (0.85 ± 0.96) | 4.58 × 10^-4^ | 4.38 × 10^-2^ | rs115186657 | 1,169 |
| cg24925163 | 2 | 128,458,248 | upstream, downstream | *SFT2D3, WDR33* | FHS | 0.01 | 52 | 0.85 (0.78 ± 0.93) | 1.73 × 10^-4^ | 2.24 × 10^-2^ | rs6733839 | 14,342 |
| cg11548732 | 2 | 160,465,499 | intronic | *BAZ2B* | FHS | 0.10 | 176 | 0.94 (0.91 ± 0.97) | 1.16 × 10^-4^ | 1.68 × 10^-2^ | rs6733839 | 5,295 |
| cg21319458 | 2 | 160,472,940 | ncRNA_exonic | *LOC643072* | BIOS | 0.07 | 11 | 0.93 (0.91 ± 0.96) | 7.50 × 10^-7^ | 3.08 × 10^-4^ | rs6733839 | -16,825 |
| cg21319458 | 2 | 160,472,940 | ncRNA_exonic | *LOC643072* | FHS | 0.05 | 17 | 0.86 (0.81 ± 0.92) | 4.91 × 10^-6^ | 1.42 × 10^-3^ | rs6733839 | -32,722 |
| cg04706995 | 2 | 160,473,062 | intronic | *ARHGAP45* | BIOS | 0.03 | 17 | 0.91 (0.87 ± 0.94) | 1.88 × 10^-6^ | 6.58 × 10^-4^ | rs6733839 | -3,260 |
| cg04706995 | 2 | 160,473,062 | ncRNA_exonic | *LOC643072* | FHS | 0.03 | 10 | 0.82 (0.76 ± 0.90) | 8.09 × 10^-6^ | 2.15 × 10^-3^ | rs6733839 | -74,591 |
| cg19903071 | 2 | 160,473,251 | ncRNA_exonic | *LOC643072* | BIOS | 0.01 | 28 | 0.91 (0.88 ± 0.95) | 9.10 × 10^-6^ | 2.35 × 10^-3^ | rs6733839 | 47,882 |
| cg15995592 | 2 | 160,614,976 | intronic | *MARCHF7* | BIOS | 0.05 | 9 | 0.92 (0.89 ± 0.96) | 2.68 × 10^-5^ | 5.66 × 10^-3^ | rs6733839 | -7,000 |
| cg15995592 | 2 | 160,614,976 | intronic | *MARCHF7* | FHS | 0.02 | 38 | 0.87 (0.81 ± 0.94) | 4.56 × 10^-4^ | 4.37 × 10^-2^ | rs6733839 | 1,169 |
| cg08347373 | 2 | 160,653,686 | intronic | *CD302, LY75-CD302* | FHS | 0.21 | 30 | 0.94 (0.92 ± 0.97) | 2.53 × 10^-5^ | 5.39 × 10^-3^ | rs6733839 | -1,228 |
| cg08347373 | 2 | 160,653,686 | UTR5 | *RASAL1* | BIOS | 0.30 | 27 | 0.97 (0.96 ± 0.98) | 2.95 × 10^-5^ | 6.01 × 10^-3^ | rs6733839 | -2,627 |
| cg04735129 | 2 | 160,654,086 | intronic | *CD302, LY75-CD302* | FHS | 0.04 | 16 | 1.13 (1.06 ± 1.20) | 3.55 × 10^-4^ | 3.68 × 10^-2^ | rs6733839 | 4,508 |
| cg24859623 | 2 | 160,654,372 | intronic | *CD302, LY75-CD302* | BIOS | 0.02 | 4 | 1.18 (1.10 ± 1.27) | 6.48 × 10^-6^ | 1.78 × 10^-3^ | rs6733839 | -32,762 |
| cg15352315 | 2 | 160,655,036 | intronic | *LY75-CD302* | FHS | 0.04 | 6 | 1.18 (1.09 ± 1.27) | 3.16 × 10^-5^ | 6.31 × 10^-3^ | rs6733839 | 30,075 |
| cg03432176 | 2 | 160,655,066 | intronic | *LY75-CD302* | FHS | 0.01 | 7 | 1.34 (1.17 ± 1.54) | 3.52 × 10^-5^ | 6.93 × 10^-3^ | rs6733839 | 12,254 |
| cg09643312 | 2 | 160,655,081 | intronic | *LY75-CD302* | FHS | 0.03 | 13 | 1.15 (1.07 ± 1.24) | 1.46 × 10^-4^ | 1.99 × 10^-2^ | rs6733839 | 1,705 |
| cg03457195 | 2 | 162,284,206 | intergenic | *TBR1, AHCTF1P1* | BIOS | 0.03 | 10 | 0.94 (0.92 ± 0.97) | 1.45 × 10^-4^ | 1.98 × 10^-2^ | rs6733839 | 1,714 |
| cg18910534 | 2 | 201,729,768 | upstream | *CLK1* | BIOS | 0.13 | 16 | 1.03 (1.01 ± 1.04) | 5.10 × 10^-4^ | 4.72 × 10^-2^ | rs6733839 | 957 |
| cg14795672 | 2 | 201,735,780 | UTR3 | *PPIL3* | BIOS | 0.06 | 9 | 0.96 (0.93 ± 0.98) | 3.65 × 10^-4^ | 3.74 × 10^-2^ | rs6733839 | -1,031 |
| cg22981993 | 2 | 202,484,029 | upstream, downstream | *C2CD6, TMEM237* | BIOS | 0.04 | 29 | 0.94 (0.91 ± 0.97) | 4.34 × 10^-4^ | 4.24 × 10^-2^ | rs6733839 | -2,215 |
| cg16661269 | 2 | 223,103,743 | intronic | *PAX3* | FHS | 0.07 | 11 | 0.91 (0.86 ± 0.96) | 5.04 × 10^-4^ | 4.68 × 10^-2^ | rs10933431 | 1,108 |
| cg11641595 | 2 | 224,902,102 | intronic | *SERPINE2* | BIOS | 0.01 | 2 | 1.16 (1.07 ± 1.26) | 5.44 × 10^-4^ | 4.93 × 10^-2^ | rs10933431 | -14,920 |
| cg15773893 | 2 | 225,702,600 | intronic | *DOCK10* | BIOS | 0.03 | 6 | 0.92 (0.87 ± 0.96) | 2.36 × 10^-4^ | 2.78 × 10^-2^ | rs10933431 | 3,300 |
| cg20363891 | 2 | 241,938,818 | intronic | *SNED1* | BIOS | 0.08 | 25 | 0.96 (0.93 ± 0.98) | 4.84 × 10^-4^ | 4.56 × 10^-2^ | rs7597763 | -3,975 |
| cg17763743 | 3 | 9,343,967 | intergenic | *SRGAP3-AS4, THUMPD3* | BIOS | 0.10 | 59 | 0.97 (0.95 ± 0.99) | 5.26 × 10^-4^ | 4.83 × 10^-2^ | rs184384746 | -593 |
| cg14220170 | 3 | 36,966,224 | intronic | *TRANK1* | BIOS | 0.25 | 47 | 1.03 (1.02 ± 1.05) | 4.85 × 10^-5^ | 8.80 × 10^-3^ | rs184384746 | 3,494 |
| cg14220170 | 3 | 36,966,224 | intronic | *TRANK1* | FHS | 0.34 | 21 | 1.04 (1.02 ± 1.07) | 1.15 × 10^-4^ | 1.66 × 10^-2^ | rs184384746 | 5,295 |
| cg03052030 | 3 | 36,985,516 | intronic | *TRANK1* | BIOS | 0.03 | 27 | 0.92 (0.89 ± 0.96) | 9.83 × 10^-5^ | 1.49 × 10^-2^ | rs184384746 | 6,267 |
| cg11870261 | 3 | 36,986,679 | intronic | *CYSTM1* | BIOS | 0.01 | 20 | 1.13 (1.07 ± 1.20) | 2.19 × 10^-5^ | 4.79 × 10^-3^ | rs184384746 | -29,150 |
| cg17445812 | 3 | 36,986,805 | upstream | *TRANK1* | BIOS | 0.52 | 46 | 1.02 (1.01 ± 1.03) | 4.07 × 10^-5^ | 7.74 × 10^-3^ | rs184384746 | 10,895 |
| cg17445812 | 3 | 36,986,805 | upstream | *TRANK1* | FHS | 0.43 | 24 | 1.04 (1.02 ± 1.06) | 1.04 × 10^-4^ | 1.55 × 10^-2^ | rs184384746 | 3,792 |
| cg09352908 | 3 | 37,017,749 | intergenic | *TRANK1, EPM2AIP1* | BIOS | 0.03 | 2 | 0.90 (0.85 ± 0.95) | 7.51 × 10^-5^ | 1.22 × 10^-2^ | rs184384746 | 6,673 |
| cg17024523 | 3 | 37,038,591 | intronic | *MLH1* | BIOS | 0.02 | 7 | 0.86 (0.80 ± 0.93) | 7.37 × 10^-5^ | 1.21 × 10^-2^ | rs184384746 | -53,779 |
| cg06284479 | 3 | 37,173,546 | intronic | *LRRFIP2* | FHS | 0.11 | 25 | 1.09 (1.05 ± 1.13) | 2.84 × 10^-5^ | 5.85 × 10^-3^ | rs184384746 | -115,467 |
| cg06284479 | 3 | 37,173,546 | intronic | *LRRFIP2* | BIOS | 0.12 | 24 | 1.05 (1.02 ± 1.07) | 8.96 × 10^-5^ | 1.39 × 10^-2^ | rs184384746 | 6,269 |
| cg15934958 | 3 | 37,212,084 | intronic | *LRRFIP2* | BIOS | 0.15 | 8 | 1.04 (1.02 ± 1.07) | 7.48 × 10^-5^ | 1.22 × 10^-2^ | rs184384746 | -55,799 |
| cg15934958 | 3 | 37,212,084 | intronic | *LRRFIP2* | FHS | 0.27 | 46 | 1.05 (1.02 ± 1.07) | 9.27 × 10^-5^ | 1.42 × 10^-2^ | rs184384746 | 6,269 |
| cg22985146 | 3 | 37,219,077 | exonic | *DUSP6* | BIOS | 0.23 | 15 | 0.97 (0.95 ± 0.98) | 4.58 × 10^-5^ | 8.45 × 10^-3^ | rs184384746 | 9,560 |
| cg22985146 | 3 | 37,219,077 | intergenic | *LRRFIP2, LOC152048* | FHS | 0.19 | 55 | 0.95 (0.92 ± 0.97) | 1.02 × 10^-4^ | 1.52 × 10^-2^ | rs184384746 | 6,261 |
| cg11321190 | 3 | 37,239,890 | upstream | *STX1B* | FHS | 0.01 | 27 | 0.78 (0.71 ± 0.87) | 1.38 × 10^-6^ | 5.08 × 10^-4^ | rs184384746 | 9,202 |
| cg11321190 | 3 | 37,239,890 | intergenic | *LRRFIP2, LOC152048* | BIOS | 0.02 | 13 | 0.91 (0.86 ± 0.95) | 5.14 × 10^-5^ | 9.15 × 10^-3^ | rs184384746 | 2,893 |
| cg21328643 | 3 | 37,258,149 | intronic | *KANSL1* | BIOS | 0.36 | 13 | 0.97 (0.96 ± 0.99) | 3.68 × 10^-5^ | 7.15 × 10^-3^ | rs184384746 | 26,203 |
| cg13609040 | 3 | 118,960,340 | ncRNA_intronic | *B4GALT4-AS1* | FHS | 0.02 | 41 | 1.17 (1.08 ± 1.27) | 6.21 × 10^-5^ | 1.06 × 10^-2^ | rs16824536 | -2,080 |
| cg13609040 | 3 | 118,960,340 | ncRNA_intronic | *B4GALT4-AS1* | BIOS | 0.02 | 24 | 1.08 (1.04 ± 1.13) | 2.34 × 10^-4^ | 2.76 × 10^-2^ | rs16824536 | -2,449 |
| cg24072947 | 3 | 136,674,783 | intergenic | *NCK1, IL20RB* | BIOS | 0.02 | 16 | 1.11 (1.06 ± 1.17) | 2.62 × 10^-5^ | 5.54 × 10^-3^ | rs16824536 | -7,000 |
| cg13702222 | 3 | 152,017,240 | UTR5 | *MBNL1* | FHS | 0.01 | 10 | 0.78 (0.69 ± 0.89) | 1.65 × 10^-4^ | 2.15 × 10^-2^ | rs16824536 | 3,191 |
| cg13702222 | 3 | 152,017,240 | UTR5 | *MBNL1* | BIOS | 0.04 | 8 | 0.92 (0.89 ± 0.96) | 2.73 × 10^-4^ | 3.06 × 10^-2^ | rs16824536 | 12,458 |
| cg14097619 | 3 | 183,945,054 | intergenic | *ABCF3, VWA5B2* | FHS | 0.02 | 9 | 0.81 (0.72 ± 0.91) | 5.01 × 10^-4^ | 4.66 × 10^-2^ | rs61762319 | 1,112 |
| cg26870570 | 3 | 183,951,128 | exonic | *VWA5B2* | BIOS | 0.10 | 10 | 1.05 (1.02 ± 1.07) | 4.49 × 10^-5^ | 8.31 × 10^-3^ | rs61762319 | 9,828 |
| cg26870570 | 3 | 183,951,128 | exonic | *VWA5B2* | FHS | 0.03 | 7 | 1.32 (1.15 ± 1.51) | 1.07 × 10^-4^ | 1.58 × 10^-2^ | rs61762319 | 3,792 |
| cg25447717 | 3 | 183,952,205 | intronic | *VWA5B2* | BIOS | 0.15 | 23 | 1.04 (1.02 ± 1.06) | 1.90 × 10^-5^ | 4.32 × 10^-3^ | rs61762319 | 2,770 |
| cg25447717 | 3 | 183,952,205 | intronic | *VWA5B2* | FHS | 0.10 | 25 | 1.09 (1.04 ± 1.14) | 1.13 × 10^-4^ | 1.64 × 10^-2^ | rs61762319 | 4,918 |
| cg00985388 | 3 | 183,952,212 | intronic | *VWA5B2* | BIOS | 0.18 | 25 | 1.04 (1.02 ± 1.06) | 6.79 × 10^-6^ | 1.85 × 10^-3^ | rs61762319 | -34,391 |
| cg00985388 | 3 | 183,952,212 | intronic | *VWA5B2* | FHS | 0.11 | 18 | 1.09 (1.04 ± 1.13) | 7.17 × 10^-5^ | 1.19 × 10^-2^ | rs61762319 | -43,351 |
| cg06242000 | 3 | 183,966,155 | intronic | *ALG3* | BIOS | 0.02 | 11 | 0.89 (0.83 ± 0.94) | 1.76 × 10^-4^ | 2.26 × 10^-2^ | rs61762319 | -2,022 |
| cg13731523 | 4 | 3,047,190 | intergenic | *GRK4, HTT-AS* | BIOS | 0.69 | 57 | 0.98 (0.97 ± 0.99) | 4.40 × 10^-4^ | 4.27 × 10^-2^ | rs3822030 | -1,558 |
| cg26275355 | 4 | 12,252,318 | ncRNA_intronic | *LINC02270* | FHS | 0.04 | 65 | 0.89 (0.84 ± 0.94) | 9.79 × 10^-5^ | 1.48 × 10^-2^ | rs6448451 | 6,268 |
| cg10582946 | 4 | 18,023,955 | upstream | *LCORL* | BIOS | 0.01 | 2 | 1.11 (1.05 ± 1.17) | 2.53 × 10^-4^ | 2.92 × 10^-2^ | rs6448451 | 2,074 |
| cg13792823 | 4 | 18,024,445 | upstream | *LCORL* | BIOS | 0.02 | 8 | 0.91 (0.87 ± 0.95) | 7.00 × 10^-6^ | 1.90 × 10^-3^ | rs6448451 | -73,837 |
| cg01089331 | 4 | 18,024,929 | intergenic | *LCORL, NONE* | BIOS | 0.03 | 5 | 0.93 (0.89 ± 0.96) | 1.11 × 10^-5^ | 2.77 × 10^-3^ | rs6448451 | 20,053 |
| cg01090611 | 4 | 83,292,493 | intronic | *HNRNPD* | FHS | 0.06 | 15 | 1.11 (1.05 ± 1.18) | 1.57 × 10^-4^ | 2.08 × 10^-2^ | rs2245466 | -4,704 |
| cg01090611 | 4 | 83,292,493 | intronic | *HNRNPD* | BIOS | 0.05 | 22 | 1.06 (1.03 ± 1.09) | 1.85 × 10^-4^ | 2.34 × 10^-2^ | rs2245466 | -5,636 |
| cg13259925 | 4 | 155,665,832 | exonic | *LRAT* | FHS | 0.13 | 13 | 0.93 (0.90 ± 0.97) | 2.18 × 10^-4^ | 2.62 × 10^-2^ | rs2245466 | -9,660 |
| cg13259925 | 4 | 155,665,832 | exonic | *LRAT* | BIOS | 0.16 | 8 | 0.97 (0.95 ± 0.99) | 3.76 × 10^-4^ | 3.82 × 10^-2^ | rs2245466 | -1,255 |
| cg23427912 | 4 | 189,059,510 | intergenic | *TRIML2, TRIML1* | BIOS | 0.20 | 44 | 1.03 (1.02 ± 1.05) | 6.65 × 10^-5^ | 1.11 × 10^-2^ | rs2245466 | 23,684 |
| cg09662430 | 5 | 307,591 | intronic | *AHRR, PDCD6* | FHS | 0.01 | 36 | 1.21 (1.09 ± 1.35) | 4.59 × 10^-4^ | 4.39 × 10^-2^ | rs112403360 | -4,005 |
| cg26427908 | 5 | 1,225,074 | UTR3 | *SLC6A19* | BIOS | 0.01 | 32 | 0.91 (0.86 ± 0.96) | 3.30 × 10^-4^ | 3.51 × 10^-2^ | rs112403360 | -2,759 |
| cg25222089 | 5 | 17,351,261 | intergenic | *BASP1, LINC02111* | BIOS | 0.05 | 25 | 1.08 (1.04 ± 1.13) | 1.83 × 10^-4^ | 2.33 × 10^-2^ | rs112403360 | -4,087 |
| cg03642555 | 5 | 34,502,788 | intergenic | *NONE, RAI14* | BIOS | 0.15 | 11 | 0.96 (0.94 ± 0.98) | 3.79 × 10^-4^ | 3.84 × 10^-2^ | rs112403360 | -1,273 |
| cg22673064 | 5 | 37,952,447 | intergenic | *LINC02110, LINC02107* | FHS | 0.01 | 18 | 0.75 (0.65 ± 0.88) | 2.77 × 10^-4^ | 3.09 × 10^-2^ | rs112403360 | 10,244 |
| cg21155100 | 5 | 56,147,662 | intronic | *MAP3K1* | FHS | 0.06 | 24 | 1.11 (1.05 ± 1.17) | 1.13 × 10^-4^ | 1.64 × 10^-2^ | rs62374257 | -5,817 |
| cg21155100 | 5 | 56,147,662 | intronic | *MAP3K1* | BIOS | 0.06 | 20 | 1.04 (1.02 ± 1.06) | 2.81 × 10^-4^ | 3.12 × 10^-2^ | rs62374257 | 6,560 |
| cg18294332 | 5 | 56,148,274 | intronic | *MAP3K1* | BIOS | 0.07 | 3 | 1.04 (1.02 ± 1.06) | 5.98 × 10^-5^ | 1.03 × 10^-2^ | rs62374257 | 13,423 |
| cg18294332 | 5 | 56,148,274 | intronic | *MAP3K1* | FHS | 0.08 | 23 | 1.09 (1.04 ± 1.13) | 1.27 × 10^-4^ | 1.78 × 10^-2^ | rs62374257 | 43,430 |
| cg24949191 | 5 | 73,969,223 | intronic | *HEXB* | BIOS | 0.17 | 29 | 0.96 (0.95 ± 0.98) | 4.64 × 10^-6^ | 1.36 × 10^-3^ | rs62374257 | -32,580 |
| cg24949191 | 5 | 73,969,223 | intronic | *HEXB* | FHS | 0.11 | 24 | 0.91 (0.87 ± 0.95) | 2.84 × 10^-5^ | 5.85 × 10^-3^ | rs62374257 | -148,861 |
| cg18248994 | 5 | 124,229,058 | intergenic | *ZNF608, LOC101927421* | FHS | 0.02 | 5 | 0.75 (0.66 ± 0.86) | 4.19 × 10^-5^ | 7.92 × 10^-3^ | rs871269 | 10,895 |
| cg10441691 | 5 | 139,520,490 | intergenic | *IGIP, LOC101929719* | FHS | 0.14 | 20 | 1.07 (1.03 ± 1.12) | 3.60 × 10^-4^ | 3.71 × 10^-2^ | rs871269 | 1,606 |
| cg17356056 | 5 | 139,526,226 | intergenic | *IGIP, LOC101929719* | FHS | 0.02 | 27 | 0.85 (0.79 ± 0.92) | 4.47 × 10^-5^ | 8.28 × 10^-3^ | rs871269 | 9,829 |
| cg17356056 | 5 | 139,526,226 | intergenic | *IGIP, LOC101929719* | BIOS | 0.05 | 15 | 0.94 (0.91 ± 0.98) | 4.91 × 10^-4^ | 4.61 × 10^-2^ | rs871269 | -4,013 |
| cg12295988 | 5 | 139,529,063 | intergenic | *IGIP, LOC101929719* | BIOS | 0.01 | 9 | 0.87 (0.81 ± 0.94) | 2.87 × 10^-4^ | 3.17 × 10^-2^ | rs871269 | 6,510 |
| cg01081189 | 5 | 139,537,190 | ncRNA_intronic | *LOC101929719* | FHS | 0.18 | 14 | 1.06 (1.03 ± 1.10) | 1.00 × 10^-4^ | 1.50 × 10^-2^ | rs871269 | 6,261 |
| cg01081189 | 5 | 139,537,190 | ncRNA_intronic | *LOC101929719* | BIOS | 0.24 | 35 | 1.03 (1.02 ± 1.05) | 2.04 × 10^-4^ | 2.51 × 10^-2^ | rs871269 | 23,975 |
| cg00275181 | 5 | 139,537,207 | ncRNA_intronic | *LOC101929719* | FHS | 0.07 | 21 | 1.12 (1.07 ± 1.18) | 9.53 × 10^-6^ | 2.44 × 10^-3^ | rs871269 | 20,260 |
| cg00275181 | 5 | 139,537,207 | ncRNA_intronic | *LOC101929719* | BIOS | 0.13 | 22 | 1.05 (1.02 ± 1.07) | 1.89 × 10^-4^ | 2.36 × 10^-2^ | rs871269 | -1,088 |
| cg00095846 | 5 | 139,551,088 | intergenic | *LOC101929719, CYSTM1* | BIOS | 0.01 | 5 | 1.16 (1.08 ± 1.25) | 2.83 × 10^-5^ | 5.85 × 10^-3^ | rs871269 | -115,467 |
| cg26211634 | 5 | 139,558,579 | intronic | *CYSTM1* | FHS | 0.19 | 17 | 1.05 (1.02 ± 1.09) | 4.35 × 10^-4^ | 4.24 × 10^-2^ | rs871269 | -2,409 |
| cg00287773 | 5 | 139,594,619 | upstream | *TRANK1* | BIOS | 0.15 | 6 | 1.04 (1.02 ± 1.06) | 2.19 × 10^-5^ | 4.79 × 10^-3^ | rs871269 | -29,150 |
| cg00287773 | 5 | 139,594,619 | intronic | *CYSTM1* | FHS | 0.13 | 28 | 1.08 (1.04 ± 1.12) | 2.38 × 10^-5^ | 5.13 × 10^-3^ | rs871269 | -29,164 |
| cg19622675 | 5 | 139,683,183 | upstream | *PFDN1* | FHS | 0.11 | 22 | 1.08 (1.04 ± 1.12) | 1.04 × 10^-4^ | 1.55 × 10^-2^ | rs871269 | 6,261 |
| cg10043954 | 5 | 140,603,034 | UTR5 | *PCDHB14* | BIOS | 0.01 | 6 | 0.87 (0.81 ± 0.93) | 4.92 × 10^-5^ | 8.87 × 10^-3^ | rs871269 | 2,015 |
| cg01139541 | 5 | 140,604,600 | exonic | *PCDHB14* | FHS | 0.04 | 23 | 0.84 (0.77 ± 0.93) | 3.59 × 10^-4^ | 3.71 × 10^-2^ | rs871269 | 1,716 |
| cg12487901 | 5 | 140,872,334 | intronic | *PCDH cluster* | FHS | 0.01 | 22 | 1.21 (1.09 ± 1.33) | 2.66 × 10^-4^ | 3.01 × 10^-2^ | rs871269 | 29,449 |
| cg00296018 | 5 | 141,205,206 | intergenic | *ARAP3, PCDH1* | FHS | 0.09 | 70 | 1.08 (1.04 ± 1.12) | 1.28 × 10^-4^ | 1.80 × 10^-2^ | rs871269 | 26,116 |
| cg07366082 | 5 | 141,233,463 | UTR3 | *PCDH1* | BIOS | 0.07 | 39 | 1.05 (1.03 ± 1.08) | 1.08 × 10^-4^ | 1.59 × 10^-2^ | rs871269 | -3,246 |
| cg00514353 | 5 | 141,233,646 | exonic | *PCDH1* | BIOS | 0.07 | 21 | 1.06 (1.03 ± 1.09) | 2.14 × 10^-4^ | 2.60 × 10^-2^ | rs871269 | -9,607 |
| cg19767205 | 5 | 141,255,282 | intronic | *PCDH1* | FHS | 0.02 | 12 | 1.29 (1.13 ± 1.46) | 1.39 × 10^-4^ | 1.92 × 10^-2^ | rs871269 | 11,822 |
| cg19811076 | 5 | 146,938,282 | intergenic | *DPYSL3, JAKMIP2-AS1* | BIOS | 0.05 | 8 | 1.07 (1.04 ± 1.11) | 1.03 × 10^-5^ | 2.60 × 10^-3^ | rs871269 | 20,239 |
| cg11935896 | 5 | 148,409,702 | intronic | *SH3TC2* | FHS | 0.03 | 18 | 1.16 (1.07 ± 1.25) | 2.29 × 10^-4^ | 2.72 × 10^-2^ | rs871269 | -2,361 |
| cg01831454 | 5 | 148,417,172 | intronic | *SH3TC2* | FHS | 0.39 | 22 | 1.04 (1.02 ± 1.06) | 1.79 × 10^-4^ | 2.29 × 10^-2^ | rs871269 | -4,045 |
| cg01831454 | 5 | 148,417,172 | intronic | *SH3TC2* | BIOS | 0.36 | 33 | 1.02 (1.01 ± 1.03) | 3.16 × 10^-4^ | 3.40 × 10^-2^ | rs871269 | 8,343 |
| cg07868561 | 5 | 148,434,089 | intronic | *SH3TC2* | FHS | 0.01 | 13 | 0.66 (0.54 ± 0.81) | 5.18 × 10^-5^ | 9.21 × 10^-3^ | rs871269 | 2,889 |
| cg07868561 | 5 | 148,434,089 | intergenic | *TTC17, MIR670* | BIOS | 0.01 | 13 | 0.86 (0.81 ± 0.93) | 5.40 × 10^-5^ | 9.47 × 10^-3^ | rs871269 | 1,998 |
| cg18465082 | 5 | 176,734,745 | intronic | *MXD3* | BIOS | 0.06 | 41 | 1.06 (1.02 ± 1.09) | 3.44 × 10^-4^ | 3.62 × 10^-2^ | rs113706587 | 11,607 |
| cg26087598 | 5 | 176,739,400 | upstream | *MXD3* | BIOS | 0.02 | 2 | 1.12 (1.06 ± 1.19) | 1.04 × 10^-4^ | 1.55 × 10^-2^ | rs113706587 | 6,261 |
| cg01140102 | 5 | 176,766,085 | intronic | *LMAN2* | FHS | 0.02 | 14 | 1.28 (1.13 ± 1.45) | 1.17 × 10^-4^ | 1.69 × 10^-2^ | rs113706587 | 49,147 |
| cg01140102 | 5 | 176,766,085 | intronic | *LMAN2* | BIOS | 0.02 | 7 | 1.10 (1.04 ± 1.15) | 4.52 × 10^-4^ | 4.35 × 10^-2^ | rs113706587 | 503 |
| cg14599956 | 5 | 176,779,173 | upstream | *LMAN2* | BIOS | 0.02 | 8 | 0.90 (0.85 ± 0.95) | 1.79 × 10^-4^ | 2.29 × 10^-2^ | rs113706587 | -4,015 |
| cg00303876 | 5 | 177,630,081 | intergenic | *GMCL2, HNRNPAB* | FHS | 0.04 | 18 | 1.13 (1.06 ± 1.22) | 5.45 × 10^-4^ | 4.93 × 10^-2^ | rs113706587 | - |
| cg22561794 | 5 | 180,335,743 | exonic | *BTNL8* | BIOS | 0.02 | 31 | 1.07 (1.03 ± 1.11) | 5.53 × 10^-4^ | 4.97 × 10^-2^ | rs113706587 | - |
| cg17487705 | 6 | 707,897 | UTR5 | *CCDC81* | BIOS | 0.03 | 25 | 1.07 (1.04 ± 1.11) | 7.97 × 10^-5^ | 1.28 × 10^-2^ | rs9271192 | 6,288 |
| cg06148264 | 6 | 2,841,468 | intronic | *SERPINB1* | FHS | 0.08 | 28 | 0.91 (0.87 ± 0.95) | 6.28 × 10^-5^ | 1.07 × 10^-2^ | rs9271192 | -2,175 |
| cg22977317 | 6 | 2,842,550 | upstream | *SERPINB1* | FHS | 0.30 | 92 | 0.96 (0.94 ± 0.98) | 2.73 × 10^-4^ | 3.06 × 10^-2^ | rs9271192 | 12,458 |
| cg23963071 | 6 | 2,901,712 | intronic | *SERPINB9* | BIOS | 0.51 | 122 | 1.02 (1.01 ± 1.02) | 1.63 × 10^-5^ | 3.83 × 10^-3^ | rs9271192 | 35,827 |
| cg09414983 | 6 | 9,399,677 | intergenic | *LOC100506207, TFAP2A* | BIOS | 0.01 | 28 | 0.92 (0.88 ± 0.96) | 4.18 × 10^-4^ | 4.13 × 10^-2^ | rs9271192 | -1,808 |
| cg12688576 | 6 | 18,387,407 | upstream | *RNF144B* | BIOS | 0.20 | 41 | 1.03 (1.01 ± 1.05) | 2.59 × 10^-4^ | 2.95 × 10^-2^ | rs9271192 | -11,577 |
| cg07523189 | 6 | 19,155,350 | ncRNA_intronic | *LOC101928519* | BIOS | 0.12 | 18 | 0.95 (0.93 ± 0.97) | 8.26 × 10^-6^ | 2.18 × 10^-3^ | rs9271192 | 10,878 |
| cg07523189 | 6 | 19,155,350 | ncRNA_intronic | *LOC101928519* | FHS | 0.06 | 46 | 0.92 (0.87 ± 0.96) | 3.03 × 10^-4^ | 3.30 × 10^-2^ | rs9271192 | -18,756 |
| cg01097611 | 6 | 25,992,243 | downstream | *TRIM38* | FHS | 0.01 | 20 | 1.28 (1.13 ± 1.45) | 1.60 × 10^-4^ | 2.11 × 10^-2^ | rs9271192 | -4,720 |
| cg10519906 | 6 | 34,563,243 | intronic | *ILRUN* | BIOS | 0.04 | 12 | 0.94 (0.91 ± 0.97) | 4.43 × 10^-4^ | 4.28 × 10^-2^ | rs1846190 | -1,571 |
| cg17167053 | 6 | 34,664,215 | ncRNA_exonic | *LOC101929243* | BIOS | 0.03 | 4 | 0.92 (0.89 ± 0.96) | 3.44 × 10^-5^ | 6.82 × 10^-3^ | rs1846190 | 12,254 |
| cg17167053 | 6 | 34,664,215 | ncRNA_exonic | *LOC101929243* | FHS | 0.03 | 15 | 0.85 (0.78 ± 0.92) | 8.05 × 10^-5^ | 1.29 × 10^-2^ | rs1846190 | 6,288 |
| cg11304212 | 6 | 34,759,018 | upstream | *UHRF1BP1* | FHS | 0.02 | 5 | 1.29 (1.12 ± 1.49) | 3.63 × 10^-4^ | 3.73 × 10^-2^ | rs1846190 | 1,591 |
| cg04326499 | 6 | 45,627,080 | intergenic | *RUNX2, CLIC5* | FHS | 0.05 | 11 | 0.89 (0.84 ± 0.95) | 4.68 × 10^-4^ | 4.45 × 10^-2^ | rs9381563 | -3,950 |
| cg12682931 | 6 | 90,119,710 | intronic | *RRAGD* | FHS | 0.03 | 36 | 0.85 (0.78 ± 0.93) | 4.26 × 10^-4^ | 4.18 × 10^-2^ | rs785129 | -1,879 |
| cg06866423 | 6 | 90,926,672 | intronic | *BACH2* | BIOS | 0.29 | 22 | 1.03 (1.02 ± 1.05) | 3.82 × 10^-5^ | 7.37 × 10^-3^ | rs785129 | 10,903 |
| cg06866423 | 6 | 90,926,672 | intronic | *BACH2* | FHS | 0.27 | 47 | 1.05 (1.02 ± 1.07) | 1.49 × 10^-4^ | 2.01 × 10^-2^ | rs785129 | -929 |
| cg09757525 | 6 | 90,928,574 | intronic | *BACH2* | BIOS | 0.15 | 38 | 1.04 (1.02 ± 1.05) | 1.88 × 10^-4^ | 2.36 × 10^-2^ | rs785129 | 16,666 |
| cg09757525 | 6 | 90,928,574 | intronic | *BACH2* | FHS | 0.12 | 18 | 1.07 (1.03 ± 1.11) | 3.55 × 10^-4^ | 3.68 × 10^-2^ | rs785129 | 4,551 |
| cg18477569 | 6 | 90,951,134 | intronic | *BACH2* | FHS | 0.71 | 66 | 1.03 (1.01 ± 1.04) | 1.48 × 10^-4^ | 2.01 × 10^-2^ | rs785129 | -6,174 |
| cg18477569 | 6 | 90,951,134 | intronic | *BACH2* | BIOS | 0.71 | 61 | 1.02 (1.01 ± 1.03) | 1.77 × 10^-4^ | 2.26 × 10^-2^ | rs785129 | -3,975 |
| cg10669449 | 6 | 106,676,237 | intronic | *ATG5* | BIOS | 0.03 | 4 | 0.90 (0.86 ± 0.95) | 7.20 × 10^-5^ | 1.19 × 10^-2^ | rs785129 | -43,416 |
| cg17829936 | 6 | 132,910,681 | exonic | *TAAR5* | BIOS | 0.77 | 34 | 0.98 (0.98 ± 0.99) | 5.44 × 10^-4^ | 4.93 × 10^-2^ | rs785129 | - |
| cg03054050 | 6 | 157,963,702 | exonic | *ZDHHC14* | BIOS | 0.02 | 15 | 0.89 (0.84 ± 0.95) | 5.13 × 10^-4^ | 4.74 × 10^-2^ | rs785129 | 957 |
| cg23707590 | 6 | 158,028,962 | intronic | *ZDHHC14* | BIOS | 0.29 | 98 | 0.97 (0.96 ± 0.99) | 3.48 × 10^-4^ | 3.64 × 10^-2^ | rs785129 | 4,609 |
| cg05937818 | 6 | 168,391,643 | intergenic | *HGC6.3, KIF25-AS1* | BIOS | 0.02 | 18 | 1.09 (1.04 ± 1.15) | 5.36 × 10^-4^ | 4.89 × 10^-2^ | rs785129 | 7,058 |
| cg13881452 | 6 | 169,601,968 | intergenic | *LINC02544, LOC101929523* | BIOS | 0.02 | 15 | 1.11 (1.05 ± 1.18) | 2.17 × 10^-4^ | 2.62 × 10^-2^ | rs785129 | -9,633 |
| cg17267654 | 6 | 170,411,557 | intergenic | *LINC00574, LOC102724511* | BIOS | 0.05 | 39 | 0.94 (0.91 ± 0.97) | 2.62 × 10^-4^ | 2.97 × 10^-2^ | rs785129 | 29,646 |
| cg03584351 | 7 | 1,184,214 | intergenic | *C7orf50, ZFAND2A* | FHS | 0.09 | 48 | 1.07 (1.03 ± 1.11) | 3.82 × 10^-4^ | 3.85 × 10^-2^ | rs6943429 | -1,421 |
| cg25510838 | 7 | 1,560,381 | intergenic | *INTS1, MAFK* | FHS | 0.05 | 5 | 0.88 (0.83 ± 0.94) | 2.59 × 10^-4^ | 2.96 × 10^-2^ | rs6943429 | -17,117 |
| cg25510838 | 7 | 1,560,381 | intergenic | *INTS1, MAFK* | BIOS | 0.05 | 5 | 0.93 (0.89 ± 0.97) | 2.73 × 10^-4^ | 3.07 × 10^-2^ | rs6943429 | 12,456 |
| cg04344923 | 7 | 1,569,228 | intergenic | *INTS1, MAFK* | BIOS | 0.06 | 8 | 0.94 (0.90 ± 0.97) | 1.26 × 10^-4^ | 1.78 × 10^-2^ | rs6943429 | 49,146 |
| cg04344923 | 7 | 1,569,228 | intergenic | *INTS1, MAFK* | FHS | 0.01 | 16 | 0.77 (0.66 ± 0.89) | 3.20 × 10^-4^ | 3.43 × 10^-2^ | rs6943429 | 7,951 |
| cg01784819 | 7 | 1,569,242 | intergenic | *INTS1, MAFK* | BIOS | 0.07 | 4 | 0.93 (0.90 ± 0.97) | 5.67 × 10^-5^ | 9.84 × 10^-3^ | rs6943429 | 29,737 |
| cg01784819 | 7 | 1,569,242 | intergenic | *INTS1, MAFK* | FHS | 0.04 | 12 | 0.85 (0.78 ± 0.92) | 6.51 × 10^-5^ | 1.10 × 10^-2^ | rs6943429 | 23,686 |
| cg10111599 | 7 | 1,573,451 | ncRNA_exonic | *LOC100128653* | FHS | 0.02 | 10 | 1.20 (1.08 ± 1.32) | 3.68 × 10^-4^ | 3.76 × 10^-2^ | rs6943429 | -1,068 |
| cg13613976 | 7 | 1,574,413 | ncRNA_exonic | *LOC100128653* | FHS | 0.10 | 9 | 0.92 (0.89 ± 0.97) | 3.55 × 10^-4^ | 3.68 × 10^-2^ | rs6943429 | 4,248 |
| cg15095913 | 7 | 1,576,976 | intronic | *MAFK* | BIOS | 0.03 | 4 | 0.90 (0.85 ± 0.95) | 1.70 × 10^-4^ | 2.21 × 10^-2^ | rs6943429 | 14,343 |
| cg23871276 | 7 | 1,577,697 | intergenic | *NRTN, FUT6* | BIOS | 0.03 | 8 | 0.91 (0.87 ± 0.96) | 8.91 × 10^-5^ | 1.38 × 10^-2^ | rs6943429 | 6,274 |
| cg23871276 | 7 | 1,577,697 | intronic | *MAFK* | FHS | 0.01 | 18 | 0.81 (0.73 ± 0.91) | 1.88 × 10^-4^ | 2.36 × 10^-2^ | rs6943429 | 7,473 |
| cg07198170 | 7 | 1,581,217 | UTR3 | *MAFK* | FHS | 0.03 | 8 | 1.19 (1.09 ± 1.31) | 2.54 × 10^-4^ | 2.93 × 10^-2^ | rs6943429 | 1,872 |
| cg07198170 | 7 | 1,581,217 | UTR3 | *MAFK* | BIOS | 0.01 | 3 | 1.16 (1.07 ± 1.25) | 3.50 × 10^-4^ | 3.66 × 10^-2^ | rs6943429 | 4,603 |
| cg04501217 | 7 | 1,583,129 | UTR3 | *TMEM184A* | FHS | 0.02 | 15 | 1.22 (1.11 ± 1.33) | 1.95 × 10^-5^ | 4.39 × 10^-3^ | rs6943429 | -29,143 |
| cg04501217 | 7 | 1,583,129 | UTR3 | *TMEM184A* | BIOS | 0.06 | 6 | 1.07 (1.04 ± 1.11) | 1.11 × 10^-4^ | 1.62 × 10^-2^ | rs6943429 | 4,312 |
| cg22500518 | 7 | 1,585,359 | UTR3 | *TMEM184A* | FHS | 0.02 | 30 | 1.18 (1.08 ± 1.29) | 1.51 × 10^-4^ | 2.03 × 10^-2^ | rs6943429 | -1,071 |
| cg07071809 | 7 | 1,585,771 | UTR3 | *TMEM184A* | FHS | 0.21 | 19 | 1.06 (1.03 ± 1.09) | 1.83 × 10^-4^ | 2.33 × 10^-2^ | rs6943429 | -4,087 |
| cg07071809 | 7 | 1,585,771 | UTR3 | *TMEM184A* | BIOS | 0.32 | 9 | 1.03 (1.01 ± 1.04) | 2.74 × 10^-4^ | 3.07 × 10^-2^ | rs6943429 | 10,300 |
| cg01753241 | 7 | 1,587,459 | exonic | *TMEM184A* | BIOS | 0.01 | 2 | 1.19 (1.08 ± 1.31) | 4.40 × 10^-4^ | 4.27 × 10^-2^ | rs6943429 | -1,562 |
| cg03958363 | 7 | 1,587,692 | intronic | *TMEM184A* | FHS | 0.70 | 47 | 1.03 (1.01 ± 1.04) | 2.53 × 10^-4^ | 2.92 × 10^-2^ | rs6943429 | 2,074 |
| cg03958363 | 7 | 1,587,692 | intronic | *TMEM184A* | BIOS | 0.74 | 15 | 1.02 (1.01 ± 1.03) | 3.66 × 10^-4^ | 3.74 × 10^-2^ | rs6943429 | -1,068 |
| cg18705808 | 7 | 1,588,284 | exonic | *TMEM184A* | FHS | 0.70 | 45 | 1.03 (1.01 ± 1.04) | 2.54 × 10^-4^ | 2.93 × 10^-2^ | rs6943429 | 18,902 |
| cg18705808 | 7 | 1,588,284 | exonic | *TMEM184A* | BIOS | 0.78 | 32 | 1.02 (1.01 ± 1.03) | 4.25 × 10^-4^ | 4.18 × 10^-2^ | rs6943429 | -1,863 |
| cg04633141 | 7 | 1,588,319 | exonic | *TMEM184A* | FHS | 0.72 | 42 | 1.03 (1.01 ± 1.04) | 1.18 × 10^-4^ | 1.69 × 10^-2^ | rs6943429 | 49,147 |
| cg04633141 | 7 | 1,588,319 | exonic | *TMEM184A* | BIOS | 0.77 | 9 | 1.02 (1.01 ± 1.03) | 3.14 × 10^-4^ | 3.38 × 10^-2^ | rs6943429 | 8,344 |
| cg23404435 | 7 | 1,588,396 | intronic | *TMEM184A* | FHS | 0.63 | 36 | 1.03 (1.01 ± 1.04) | 2.30 × 10^-4^ | 2.72 × 10^-2^ | rs6943429 | -2,369 |
| cg23404435 | 7 | 1,588,396 | intronic | *TMEM184A* | BIOS | 0.72 | 14 | 1.02 (1.01 ± 1.03) | 3.50 × 10^-4^ | 3.66 × 10^-2^ | rs6943429 | 4,602 |
| cg09495282 | 7 | 1,588,579 | intronic | *TMEM184A* | FHS | 0.05 | 15 | 1.13 (1.07 ± 1.20) | 6.17 × 10^-5^ | 1.05 × 10^-2^ | rs6943429 | -1,979 |
| cg09495282 | 7 | 1,588,579 | intronic | *TMEM184A* | BIOS | 0.06 | 4 | 1.07 (1.03 ± 1.10) | 4.92 × 10^-4^ | 4.61 × 10^-2^ | rs6943429 | -5,688 |
| cg12234009 | 7 | 1,589,689 | intronic | *TMEM184A* | BIOS | 0.02 | 5 | 1.13 (1.06 ± 1.21) | 1.12 × 10^-4^ | 1.63 × 10^-2^ | rs6943429 | 782 |
| cg10633906 | 7 | 1,595,602 | intronic | *TMEM184A* | FHS | 0.09 | 12 | 0.92 (0.88 ± 0.96) | 1.29 × 10^-4^ | 1.80 × 10^-2^ | rs6943429 | 18,583 |
| cg10633906 | 7 | 1,595,602 | intronic | *TMEM184A* | BIOS | 0.48 | 14 | 0.98 (0.97 ± 0.99) | 2.56 × 10^-4^ | 2.94 × 10^-2^ | rs6943429 | 4,810 |
| cg00411097 | 7 | 1,596,050 | upstream | *TMEM184A* | BIOS | 0.57 | 34 | 0.98 (0.97 ± 0.99) | 1.66 × 10^-4^ | 2.16 × 10^-2^ | rs6943429 | -2,142 |
| cg00411097 | 7 | 1,596,050 | upstream | *TMEM184A* | FHS | 0.37 | 33 | 0.96 (0.94 ± 0.98) | 2.04 × 10^-4^ | 2.51 × 10^-2^ | rs6943429 | 23,975 |
| cg09990600 | 7 | 1,596,103 | upstream | *TMEM184A* | FHS | 0.34 | 15 | 0.96 (0.94 ± 0.98) | 2.11 × 10^-4^ | 2.56 × 10^-2^ | rs6943429 | 6,369 |
| cg12503394 | 7 | 1,596,118 | upstream | *TMEM184A* | FHS | 0.52 | 26 | 0.97 (0.95 ± 0.98) | 1.85 × 10^-4^ | 2.34 × 10^-2^ | rs6943429 | -5,636 |
| cg12503394 | 7 | 1,596,118 | upstream | *TMEM184A* | BIOS | 0.64 | 23 | 0.98 (0.97 ± 0.99) | 3.20 × 10^-4^ | 3.43 × 10^-2^ | rs6943429 | 7,948 |
| cg21368161 | 7 | 1,596,136 | upstream | *TMEM184A* | FHS | 0.39 | 38 | 0.96 (0.95 ± 0.98) | 1.54 × 10^-4^ | 2.04 × 10^-2^ | rs6943429 | -1,229 |
| cg21368161 | 7 | 1,596,136 | upstream | *TMEM184A* | BIOS | 0.58 | 40 | 0.98 (0.97 ± 0.99) | 3.23 × 10^-4^ | 3.45 × 10^-2^ | rs6943429 | 7,816 |
| cg21005607 | 7 | 24,376,838 | intergenic | *NPY, MPP6* | FHS | 0.04 | 59 | 1.12 (1.06 ± 1.19) | 4.88 × 10^-5^ | 8.85 × 10^-3^ | rs1160871 | 2,023 |
| cg21005607 | 7 | 24,376,838 | intergenic | *NPY, MPP6* | BIOS | 0.11 | 39 | 1.03 (1.02 ± 1.05) | 1.86 × 10^-4^ | 2.35 × 10^-2^ | rs1160871 | -5,636 |
| cg16826055 | 7 | 41,087,207 | intergenic | *LINC01450, LINC01449* | BIOS | 0.22 | 36 | 1.03 (1.01 ± 1.04) | 2.97 × 10^-4^ | 3.25 × 10^-2^ | rs2718058 | 6,501 |
| cg16826055 | 7 | 41,087,207 | intergenic | *LINC01450, LINC01449* | FHS | 0.22 | 43 | 1.05 (1.02 ± 1.08) | 4.00 × 10^-4^ | 3.99 × 10^-2^ | rs2718058 | -1,622 |
| cg14402224 | 7 | 50,570,049 | intronic | *DDC* | BIOS | 0.02 | 16 | 1.11 (1.06 ± 1.17) | 6.66 × 10^-6^ | 1.82 × 10^-3^ | rs76928645 | -32,762 |
| cg20336341 | 7 | 50,628,841 | UTR3 | *TAP2* | FHS | 0.02 | 20 | 0.83 (0.77 ± 0.91) | 3.69 × 10^-5^ | 7.15 × 10^-3^ | rs76928645 | 10,906 |
| cg18283321 | 7 | 50,629,542 | intronic | *DDC* | BIOS | 0.06 | 18 | 1.07 (1.04 ± 1.10) | 2.71 × 10^-6^ | 8.77 × 10^-4^ | rs76928645 | 11,137 |
| cg05493751 | 7 | 50,862,035 | upstream | *GRB10* | BIOS | 0.01 | 12 | 0.91 (0.87 ± 0.95) | 1.28 × 10^-4^ | 1.80 × 10^-2^ | rs76928645 | 25,577 |
| cg24012595 | 7 | 99,150,098 | upstream | *FAM200A* | FHS | 0.02 | 20 | 1.20 (1.09 ± 1.32) | 1.43 × 10^-4^ | 1.96 × 10^-2^ | rs7384878 | 5,532 |
| cg00353353 | 7 | 157,371,980 | intronic | *PTPRN2* | FHS | 0.02 | 16 | 0.76 (0.66 ± 0.88) | 2.16 × 10^-4^ | 2.62 × 10^-2^ | rs114360492 | -9,630 |
| cg08312330 | 8 | 6,783,670 | upstream | *DEFA6* | FHS | 0.02 | 33 | 1.16 (1.07 ± 1.25) | 3.64 × 10^-4^ | 3.74 × 10^-2^ | rs1065712 | -1,030 |
| cg14709253 | 8 | 17,519,419 | intronic | *MTUS1* | BIOS | 0.14 | 40 | 0.97 (0.95 ± 0.98) | 5.78 × 10^-5^ | 9.98 × 10^-3^ | rs1065712 | 29,677 |
| cg12548824 | 8 | 17,554,892 | UTR5 | *MTUS1* | FHS | 0.50 | 40 | 1.03 (1.02 ± 1.05) | 2.06 × 10^-4^ | 2.52 × 10^-2^ | rs1065712 | 19,043 |
| cg01993952 | 8 | 17,554,904 | UTR5 | *MTUS1* | FHS | 0.42 | 21 | 1.04 (1.02 ± 1.06) | 2.70 × 10^-4^ | 3.04 × 10^-2^ | rs1065712 | 12,614 |
| cg09433588 | 8 | 21,899,862 | downstream | *KLF16* | BIOS | 0.08 | 42 | 0.95 (0.92 ± 0.97) | 2.05 × 10^-5^ | 4.55 × 10^-3^ | rs28834970 | -29,149 |
| cg09433588 | 8 | 21,899,862 | downstream | *LYPD5* | FHS | 0.02 | 60 | 0.88 (0.83 ± 0.94) | 6.58 × 10^-5^ | 1.10 × 10^-2^ | rs28834970 | 23,686 |
| cg26343298 | 8 | 95,960,752 | intronic | *NDUFAF6, TP53INP1* | FHS | 0.22 | 9 | 1.08 (1.05 ± 1.11) | 2.43 × 10^-8^ | 1.81 × 10^-5^ | rs61732533 | -23,291 |
| cg26343298 | 8 | 95,960,752 | intronic | *NDUFAF6, TP53INP1* | BIOS | 0.28 | 29 | 1.04 (1.02 ± 1.05) | **1.25 × 10^-7^** | 6.98 × 10^-5^ | rs61732533 | 4,371 |
| cg16049864 | 8 | 95,962,084 | intronic | *NDUFAF6* | FHS | 0.71 | 54 | 0.96 (0.95 ± 0.98) | 1.91 × 10^-8^ | 1.46 × 10^-5^ | rs61732533 | -20,515 |
| cg16049864 | 8 | 95,962,084 | intronic | *EXOC3L2* | BIOS | 0.77 | 67 | 0.98 (0.97 ± 0.99) | **1.10 × 10^-7^** | 6.35 × 10^-5^ | rs61732533 | 4,372 |
| cg09323728 | 8 | 95,962,352 | intronic | *NDUFAF6* | FHS | 0.49 | 51 | 0.95 (0.94 ± 0.97) | 2.67 × 10^-8^ | 1.96 × 10^-5^ | rs61732533 | -23,291 |
| cg09323728 | 8 | 95,962,352 | intronic | *NDUFAF6* | BIOS | 0.68 | 47 | 0.97 (0.97 ± 0.98) | 4.16 × 10^-8^ | 2.84 × 10^-5^ | rs61732533 | 42,367 |
| cg23172400 | 8 | 95,962,367 | intronic | *NDUFAF6* | FHS | 0.53 | 53 | 0.96 (0.94 ± 0.97) | 3.75 × 10^-8^ | 2.60 × 10^-5^ | rs61732533 | -28,524 |
| cg23172400 | 8 | 95,962,367 | intronic | *NDUFAF6* | BIOS | 0.64 | 84 | 0.98 (0.97 ± 0.98) | **1.42 × 10^-7^** | 7.69 × 10^-5^ | rs61732533 | 4,340 |
| cg13393036 | 8 | 95,962,371 | intronic | *NDUFAF6* | FHS | 0.60 | 80 | 0.96 (0.95 ± 0.97) | 3.44 × 10^-8^ | 2.41 × 10^-5^ | rs61732533 | -27,921 |
| cg13393036 | 8 | 95,962,371 | intronic | *NDUFAF6* | BIOS | 0.71 | 58 | 0.98 (0.97 ± 0.98) | 6.85 × 10^-8^ | 4.20 × 10^-5^ | rs61732533 | 4,372 |
| cg20039814 | 8 | 95,962,383 | intronic | *NDUFAF6* | FHS | 0.66 | 61 | 0.96 (0.95 ± 0.98) | 4.50 × 10^-8^ | 3.03 × 10^-5^ | rs61732533 | 4,459 |
| cg20039814 | 8 | 95,962,383 | intronic | *NDUFAF6* | BIOS | 0.73 | 103 | 0.98 (0.97 ± 0.98) | 5.42 × 10^-8^ | 3.52 × 10^-5^ | rs61732533 | 4,444 |
| cg18059933 | 8 | 95,962,463 | intronic | *NDUFAF6* | BIOS | 0.74 | 49 | 0.98 (0.97 ± 0.98) | 2.69 × 10^-8^ | 1.96 × 10^-5^ | rs61732533 | -23,311 |
| cg18059933 | 8 | 95,962,463 | intronic | *NDUFAF6* | FHS | 0.64 | 54 | 0.96 (0.95 ± 0.98) | 3.12 × 10^-8^ | 2.22 × 10^-5^ | rs61732533 | -27,780 |
| cg03629107 | 8 | 101,678,439 | intergenic | *SNX31, PABPC1* | FHS | 0.03 | 13 | 1.28 (1.17 ± 1.40) | 4.77 × 10^-8^ | 3.19 × 10^-5^ | rs61732533 | 4,459 |
| cg03629107 | 8 | 101,678,439 | intergenic | *SNX31, PABPC1* | BIOS | 0.03 | 17 | 1.12 (1.07 ± 1.17) | 8.37 × 10^-7^ | 3.38 × 10^-4^ | rs61732533 | -16,894 |
| cg17448481 | 8 | 118,991,759 | intronic | *EXT1* | FHS | 0.02 | 23 | 0.86 (0.79 ± 0.93) | 2.52 × 10^-4^ | 2.91 × 10^-2^ | rs61732533 | 2,933 |
| cg06895664 | 8 | 119,531,106 | intronic | *SAMD12* | FHS | 0.05 | 33 | 1.12 (1.05 ± 1.20) | 5.01 × 10^-4^ | 4.66 × 10^-2^ | rs61732533 | 1,108 |
| cg26140475 | 8 | 126,525,558 | intergenic | *TRIB1, LINC00861* | BIOS | 0.01 | 7 | 0.79 (0.73 ± 0.86) | **1.04 × 10^-8^** | 8.31 × 10^-6^ | rs61732533 | -20,411 |
| cg08843064 | 8 | 126,619,805 | intergenic | *TRIB1, LINC00861* | BIOS | 0.04 | 11 | 0.92 (0.89 ± 0.96) | 1.49 × 10^-4^ | 2.01 × 10^-2^ | rs61732533 | -6,188 |
| cg08843064 | 8 | 126,619,805 | intergenic | *TRIB1, LINC00861* | FHS | 0.02 | 6 | 0.77 (0.67 ± 0.89) | 3.53 × 10^-4^ | 3.68 × 10^-2^ | rs61732533 | 4,551 |
| cg27031099 | 8 | 126,620,534 | intergenic | *TRIB1, LINC00861* | FHS | 0.23 | 10 | 0.95 (0.92 ± 0.97) | 1.76 × 10^-4^ | 2.26 × 10^-2^ | rs61732533 | -1,432 |
| cg27031099 | 8 | 126,620,534 | intergenic | *TRIB1, LINC00861* | BIOS | 0.32 | 11 | 0.98 (0.96 ± 0.99) | 2.23 × 10^-4^ | 2.68 × 10^-2^ | rs61732533 | - |
| cg14105019 | 9 | 95,844,054 | intronic | *SUSD3* | BIOS | 0.29 | 17 | 0.98 (0.97 ± 0.99) | 1.87 × 10^-4^ | 2.36 × 10^-2^ | rs1800978 | 16,667 |
| cg14105019 | 9 | 95,844,054 | intronic | *SUSD3* | FHS | 0.30 | 33 | 0.96 (0.94 ± 0.98) | 3.37 × 10^-4^ | 3.56 × 10^-2^ | rs1800978 | 11,608 |
| cg04582938 | 9 | 139,971,429 | upstream | *UAP1L1* | BIOS | 0.21 | 36 | 1.04 (1.02 ± 1.06) | 3.07 × 10^-4^ | 3.34 × 10^-2^ | rs1800978 | 8,929 |
| cg07571038 | 9 | 140,352,910 | intronic | *NSMF* | BIOS | 0.02 | 20 | 0.92 (0.88 ± 0.96) | 8.93 × 10^-5^ | 1.39 × 10^-2^ | rs1800978 | 6,271 |
| cg18989491 | 10 | 6,185,502 | intergenic | *RBM17, PFKFB3* | FHS | 0.31 | 116 | 0.95 (0.92 ± 0.98) | 5.19 × 10^-4^ | 4.77 × 10^-2^ | rs11257238 | -1,046 |
| cg00694258 | 10 | 32,038,939 | intergenic | *ZEB1, ARHGAP12* | FHS | 0.55 | 27 | 1.03 (1.01 ± 1.05) | 1.85 × 10^-4^ | 2.34 × 10^-2^ | rs7912495 | -5,636 |
| cg00694258 | 10 | 32,038,939 | intergenic | *ZEB1, ARHGAP12* | BIOS | 0.52 | 31 | 1.01 (1.01 ± 1.02) | 2.55 × 10^-4^ | 2.93 × 10^-2^ | rs7912495 | 8,742 |
| cg20442379 | 10 | 60,024,634 | intronic | *IPMK* | BIOS | 0.03 | 10 | 1.10 (1.05 ± 1.14) | 3.88 × 10^-6^ | 1.18 × 10^-3^ | rs7902657 | -32,580 |
| cg20442379 | 10 | 60,024,634 | intronic | *IPMK* | FHS | 0.02 | 22 | 1.29 (1.15 ± 1.44) | 1.00 × 10^-5^ | 2.53 × 10^-3^ | rs7902657 | 20,239 |
| cg16915659 | 10 | 60,032,665 | intronic | *CISD1* | BIOS | 0.07 | 18 | 1.05 (1.02 ± 1.07) | 4.36 × 10^-5^ | 8.16 × 10^-3^ | rs7902657 | 10,838 |
| cg22876739 | 10 | 70,319,774 | upstream | *TET1* | BIOS | 0.18 | 31 | 0.99 (0.98 ± 0.99) | 4.40 × 10^-4^ | 4.27 × 10^-2^ | rs7068231 | -1,444 |
| cg10850838 | 10 | 72,201,600 | intronic | *NODAL* | BIOS | 0.24 | 44 | 0.97 (0.96 ± 0.99) | 5.00 × 10^-4^ | 4.66 × 10^-2^ | rs6586028 | 1,126 |
| cg07934434 | 10 | 87,898,692 | exonic | *GRID1* | FHS | 0.31 | 9 | 1.04 (1.02 ± 1.06) | 5.49 × 10^-4^ | 4.95 × 10^-2^ | rs6586028 | - |
| cg08300860 | 10 | 88,428,257 | intronic | *LDB3* | BIOS | 0.04 | 12 | 1.06 (1.03 ± 1.10) | 4.94 × 10^-4^ | 4.63 × 10^-2^ | rs6586028 | 1,126 |
| cg26338757 | 10 | 88,428,295 | UTR5 | *LDB3* | FHS | 0.28 | 25 | 1.04 (1.02 ± 1.07) | 3.92 × 10^-4^ | 3.92 × 10^-2^ | rs6586028 | -1,532 |
| cg02879656 | 10 | 88,441,539 | exonic | *LDB3* | FHS | 0.01 | 15 | 1.27 (1.13 ± 1.43) | 3.98 × 10^-5^ | 7.63 × 10^-3^ | rs6586028 | 10,895 |
| cg26860750 | 10 | 98,955,810 | ncRNA_intronic | *ARHGAP19-SLIT1* | FHS | 0.05 | 36 | 1.11 (1.05 ± 1.18) | 1.99 × 10^-4^ | 2.47 × 10^-2^ | rs6584063 | 106,594 |
| cg26860750 | 10 | 98,955,810 | ncRNA_intronic | *ARHGAP19-SLIT1* | BIOS | 0.06 | 4 | 1.06 (1.03 ± 1.10) | 4.24 × 10^-4^ | 4.17 × 10^-2^ | rs6584063 | -1,829 |
| cg24590430 | 10 | 99,097,076 | intergenic | *FRAT2, RRP12* | BIOS | 0.64 | 35 | 1.02 (1.01 ± 1.03) | 3.49 × 10^-4^ | 3.65 × 10^-2^ | rs6584063 | 4,603 |
| cg24590430 | 10 | 99,097,076 | intergenic | *FRAT2, RRP12* | FHS | 0.51 | 20 | 1.03 (1.01 ± 1.05) | 4.52 × 10^-4^ | 4.35 × 10^-2^ | rs6584063 | -879 |
| cg25603927 | 10 | 99,185,264 | ncRNA_intronic | *LOC644215* | FHS | 0.11 | 115 | 0.94 (0.91 ± 0.97) | 9.19 × 10^-5^ | 1.41 × 10^-2^ | rs6584063 | 6,269 |
| cg07052063 | 10 | 99,255,236 | intronic | *MMS19* | FHS | 0.19 | 43 | 0.94 (0.91 ± 0.96) | 8.72 × 10^-6^ | 2.27 × 10^-3^ | rs6584063 | 8,279 |
| cg07052063 | 10 | 99,255,236 | intronic | *MMS19* | BIOS | 0.22 | 35 | 0.97 (0.95 ± 0.98) | 3.11 × 10^-5^ | 6.23 × 10^-3^ | rs6584063 | 30,076 |
| cg08217716 | 10 | 102,589,250 | UTR3 | *PAX2* | BIOS | 0.01 | 4 | 0.87 (0.80 ± 0.94) | 4.55 × 10^-4^ | 4.37 × 10^-2^ | rs6584063 | 26,479 |
| cg03057409 | 10 | 102,729,640 | intergenic | *SLF2, SEMA4G* | FHS | 0.02 | 8 | 1.27 (1.12 ± 1.43) | 1.61 × 10^-4^ | 2.11 × 10^-2^ | rs6584063 | -4,720 |
| cg03057409 | 10 | 102,729,640 | intergenic | *SLF2, SEMA4G* | BIOS | 0.08 | 19 | 1.05 (1.02 ± 1.07) | 2.16 × 10^-4^ | 2.62 × 10^-2^ | rs6584063 | -9,630 |
| cg05962511 | 10 | 102,730,022 | intergenic | *SLF2, SEMA4G* | FHS | 0.19 | 17 | 1.05 (1.02 ± 1.08) | 4.74 × 10^-4^ | 4.49 × 10^-2^ | rs6584063 | -3,968 |
| cg05428706 | 10 | 102,730,130 | intergenic | *SLF2, SEMA4G* | FHS | 0.04 | 13 | 1.13 (1.06 ± 1.21) | 1.92 × 10^-4^ | 2.40 × 10^-2^ | rs6584063 | -1,916 |
| cg09319822 | 10 | 102,746,444 | UTR3 | *MRPL43* | FHS | 0.03 | 9 | 1.18 (1.09 ± 1.28) | 7.69 × 10^-5^ | 1.25 × 10^-2^ | rs6584063 | 6,297 |
| cg09319822 | 10 | 102,746,444 | UTR3 | *MRPL43* | BIOS | 0.04 | 40 | 1.05 (1.02 ± 1.08) | 3.59 × 10^-4^ | 3.71 × 10^-2^ | rs6584063 | 1,724 |
| cg19914904 | 10 | 102,756,165 | upstream | *LZTS2* | BIOS | 0.02 | 11 | 0.90 (0.85 ± 0.95) | 2.05 × 10^-4^ | 2.52 × 10^-2^ | rs6584063 | 23,972 |
| cg07188104 | 10 | 107,838,889 | intergenic | *LINC02627, LINC02624* | BIOS | 0.05 | 9 | 1.06 (1.03 ± 1.10) | 1.27 × 10^-4^ | 1.79 × 10^-2^ | rs6584063 | 43,430 |
| cg03075966 | 10 | 120,981,763 | intronic | *GRK5* | BIOS | 0.34 | 41 | 1.02 (1.01 ± 1.03) | 4.53 × 10^-4^ | 4.35 × 10^-2^ | rs7908662 | 26,518 |
| cg03075966 | 10 | 120,981,763 | intronic | *GRK5* | FHS | 0.37 | 39 | 1.04 (1.02 ± 1.06) | 4.58 × 10^-4^ | 4.38 × 10^-2^ | rs7908662 | -3,863 |
| cg07984614 | 10 | 126,314,798 | intronic | *FAM53B* | BIOS | 0.02 | 7 | 1.09 (1.04 ± 1.15) | 5.44 × 10^-4^ | 4.93 × 10^-2^ | rs7908662 | - |
| cg04839683 | 11 | 15,737,281 | intergenic | *LOC102724957, SOX6* | FHS | 0.04 | 22 | 1.13 (1.06 ± 1.20) | 2.95 × 10^-4^ | 3.24 × 10^-2^ | rs3740688 | 6,501 |
| cg08354889 | 11 | 33,037,530 | exonic | *DEPDC7* | FHS | 0.05 | 1 | 0.87 (0.81 ± 0.93) | 1.30 × 10^-4^ | 1.82 × 10^-2^ | rs3740688 | 18,488 |
| cg26268276 | 11 | 33,038,313 | intronic | *DEPDC7* | FHS | 0.32 | 57 | 0.96 (0.94 ± 0.98) | 2.21 × 10^-4^ | 2.65 × 10^-2^ | rs3740688 | -9,660 |
| cg13201808 | 11 | 43,569,269 | intergenic | *FHL2, LOC285000* | FHS | 0.04 | 25 | 0.88 (0.82 ± 0.93) | 5.40 × 10^-5^ | 9.47 × 10^-3^ | rs3740688 | -708 |
| cg18557707 | 11 | 48,922,269 | intergenic | *OR4A47, TRIM49B* | FHS | 0.01 | 15 | 1.21 (1.09 ± 1.34) | 4.01 × 10^-4^ | 3.99 × 10^-2^ | rs10838725 | -1,633 |
| cg11886777 | 11 | 48,922,271 | intergenic | *OR4A47, TRIM49B* | FHS | 0.02 | 34 | 1.23 (1.11 ± 1.37) | 1.45 × 10^-4^ | 1.98 × 10^-2^ | rs10838725 | -20,320 |
| cg09727084 | 11 | 48,990,211 | intergenic | *OR4A47, TRIM49B* | FHS | 0.04 | 25 | 1.10 (1.04 ± 1.16) | 4.06 × 10^-4^ | 4.04 × 10^-2^ | rs10838725 | -1,649 |
| cg00606739 | 11 | 49,580,019 | upstream | *LOC440040* | FHS | 0.01 | 29 | 0.83 (0.75 ± 0.92) | 4.93 × 10^-4^ | 4.62 × 10^-2^ | rs10838725 | -16,004 |
| cg01165560 | 11 | 49,911,386 | intergenic | *LOC440040, OR4C13* | FHS | 0.04 | 23 | 1.15 (1.07 ± 1.23) | 8.70 × 10^-5^ | 1.36 × 10^-2^ | rs10838725 | 6,279 |
| cg08354187 | 11 | 60,776,399 | intronic | *CD6* | FHS | 0.01 | 17 | 1.49 (1.30 ± 1.70) | **7.70 × 10^-9^** | 6.46 × 10^-6^ | rs1582763 | -8,085 |
| cg01727686 | 11 | 63,997,306 | intronic | *NUDT22* | FHS | 0.01 | 7 | 1.24 (1.10 ± 1.40) | 4.38 × 10^-4^ | 4.26 × 10^-2^ | rs1582763 | -2,477 |
| cg17155859 | 11 | 64,026,541 | exonic | *PLCB3* | BIOS | 0.04 | 5 | 1.08 (1.03 ± 1.12) | 3.33 × 10^-4^ | 3.54 × 10^-2^ | rs1582763 | -3,394 |
| cg03399927 | 11 | 64,036,876 | UTR3 | *PLCB3* | FHS | 0.01 | 37 | 1.16 (1.07 ± 1.26) | 3.51 × 10^-4^ | 3.66 × 10^-2^ | rs1582763 | 4,587 |
| cg16978571 | 11 | 64,066,625 | ncRNA_intronic | *KCNK4-TEX40* | BIOS | 0.03 | 15 | 0.92 (0.89 ± 0.96) | 2.56 × 10^-4^ | 2.93 × 10^-2^ | rs1582763 | -2,239 |
| cg24580001 | 11 | 64,106,532 | intergenic | *PRDX5, CCDC88B* | BIOS | 0.01 | 6 | 0.87 (0.80 ± 0.94) | 4.01 × 10^-4^ | 3.99 × 10^-2^ | rs1582763 | -1,627 |
| cg04422903 | 11 | 64,108,550 | intronic | *CCDC88B* | FHS | 0.40 | 22 | 1.03 (1.02 ± 1.05) | 4.63 × 10^-4^ | 4.42 × 10^-2^ | rs1582763 | -3,948 |
| cg16718999 | 11 | 64,108,790 | exonic | *CCDC88B* | BIOS | 0.02 | 9 | 1.10 (1.04 ± 1.17) | 5.27 × 10^-4^ | 4.83 × 10^-2^ | rs1582763 | -1,387 |
| cg08904394 | 11 | 64,108,902 | exonic | *CCDC88B* | BIOS | 0.08 | 17 | 0.94 (0.92 ± 0.97) | 9.31 × 10^-5^ | 1.42 × 10^-2^ | rs1582763 | 6,268 |
| cg04024572 | 11 | 65,657,704 | upstream | *CCDC85B* | BIOS | 0.04 | 31 | 1.06 (1.03 ± 1.10) | 1.15 × 10^-4^ | 1.66 × 10^-2^ | rs1582763 | -5,853 |
| cg04024572 | 11 | 65,657,704 | upstream | *CCDC85B* | FHS | 0.01 | 5 | 1.30 (1.13 ± 1.50) | 1.89 × 10^-4^ | 2.36 × 10^-2^ | rs1582763 | -1,088 |
| cg22360649 | 11 | 65,657,733 | upstream | *CCDC85B* | BIOS | 0.21 | 56 | 1.03 (1.02 ± 1.04) | 1.70 × 10^-5^ | 3.94 × 10^-3^ | rs1582763 | 18,113 |
| cg22360649 | 11 | 65,657,733 | upstream | *CCDC85B* | FHS | 0.10 | 42 | 1.07 (1.03 ± 1.11) | 1.76 × 10^-4^ | 2.26 × 10^-2^ | rs1582763 | -1,364 |
| cg16073427 | 11 | 65,657,742 | upstream | *CCDC85B* | BIOS | 0.20 | 35 | 1.03 (1.02 ± 1.05) | 4.46 × 10^-5^ | 8.28 × 10^-3^ | rs1582763 | 10,749 |
| cg16073427 | 11 | 65,657,742 | upstream | *CCDC85B* | FHS | 0.06 | 6 | 1.12 (1.06 ± 1.19) | 1.12 × 10^-4^ | 1.64 × 10^-2^ | rs1582763 | -11,422 |
| cg09157813 | 11 | 104,769,087 | intronic | *CASP12* | FHS | 0.42 | 17 | 1.03 (1.02 ± 1.05) | 3.84 × 10^-4^ | 3.86 × 10^-2^ | rs11218343 | -1,467 |
| cg18471635 | 11 | 104,769,411 | upstream | *CASP12* | FHS | 0.21 | 39 | 1.05 (1.02 ± 1.08) | 5.04 × 10^-4^ | 4.68 × 10^-2^ | rs11218343 | 957 |
| cg18471635 | 11 | 104,769,411 | upstream | *CASP12* | BIOS | 0.21 | 7 | 1.03 (1.01 ± 1.04) | 5.46 × 10^-4^ | 4.94 × 10^-2^ | rs11218343 | - |
| cg03725573 | 11 | 113,962,901 | intronic | *ZBTB16* | FHS | 0.04 | 55 | 0.88 (0.83 ± 0.94) | 4.29 × 10^-5^ | 8.06 × 10^-3^ | rs11218343 | 10,874 |
| cg20373012 | 11 | 122,523,943 | intergenic | *MIR100HG, UBASH3B* | FHS | 0.03 | 18 | 1.26 (1.15 ± 1.38) | 5.37 × 10^-7^ | 2.31 × 10^-4^ | rs11218343 | -16,814 |
| cg20373012 | 11 | 122,523,943 | intergenic | *MIR100HG, UBASH3B* | BIOS | 0.06 | 17 | 1.10 (1.05 ± 1.15) | 5.83 × 10^-5^ | 1.00 × 10^-2^ | rs11218343 | 23,179 |
| cg05931265 | 11 | 122,527,736 | intronic | *UBASH3B* | BIOS | 0.06 | 20 | 0.92 (0.88 ± 0.96) | 2.80 × 10^-4^ | 3.10 × 10^-2^ | rs11218343 | 6,601 |
| cg20555462 | 11 | 122,535,518 | intronic | *UBASH3B* | BIOS | 0.21 | 29 | 0.95 (0.92 ± 0.97) | 2.30 × 10^-6^ | 7.62 × 10^-4^ | rs11218343 | -5,600 |
| cg25994988 | 11 | 122,652,382 | intronic | *UBASH3B* | FHS | 0.01 | 18 | 1.38 (1.21 ± 1.58) | 1.93 × 10^-6^ | 6.74 × 10^-4^ | rs11218343 | -3,270 |
| cg25994988 | 11 | 122,652,382 | intronic | *UBASH3B* | BIOS | 0.05 | 24 | 1.07 (1.03 ± 1.10) | 5.60 × 10^-5^ | 9.77 × 10^-3^ | rs11218343 | 31,871 |
| cg03812107 | 11 | 122,849,037 | exonic | *APOBEC2* | FHS | 0.02 | 39 | 0.87 (0.81 ± 0.93) | 9.90 × 10^-5^ | 1.49 × 10^-2^ | rs11218343 | 6,261 |
| cg05868564 | 11 | 123,351,497 | intronic | *GRAMD1B* | BIOS | 0.08 | 27 | 1.05 (1.02 ± 1.07) | 2.01 × 10^-4^ | 2.47 × 10^-2^ | rs11218343 | 23,975 |
| cg01699740 | 11 | 123,396,486 | UTR5 | *GRAMD1B* | BIOS | 0.03 | 23 | 1.10 (1.05 ± 1.15) | 1.11 × 10^-4^ | 1.62 × 10^-2^ | rs11218343 | 4,312 |
| cg17105005 | 11 | 130,260,541 | ncRNA_intronic | *ZBTB44-DT* | BIOS | 0.07 | 16 | 1.05 (1.02 ± 1.08) | 3.66 × 10^-4^ | 3.74 × 10^-2^ | rs11218343 | -1,066 |
| cg19286986 | 12 | 6,931,017 | UTR5 | *GPR162* | BIOS | 0.03 | 34 | 1.06 (1.03 ± 1.09) | 3.97 × 10^-4^ | 3.96 × 10^-2^ | rs6489896 | -1,617 |
| cg21701774 | 12 | 7,126,016 | upstream | *LPCAT3* | FHS | 0.01 | 64 | 1.27 (1.12 ± 1.45) | 2.78 × 10^-4^ | 3.09 × 10^-2^ | rs6489896 | 6,629 |
| cg01777341 | 12 | 51,784,862 | intronic | *GALNT6* | BIOS | 0.04 | 19 | 1.08 (1.04 ± 1.12) | 4.84 × 10^-5^ | 8.80 × 10^-3^ | rs6489896 | 9,177 |
| cg01777341 | 12 | 51,784,862 | intronic | *GALNT6* | FHS | 0.02 | 5 | 1.28 (1.13 ± 1.45) | 1.01 × 10^-4^ | 1.51 × 10^-2^ | rs6489896 | 6,261 |
| cg00274203 | 12 | 56,661,537 | intronic | *COQ10A* | BIOS | 0.02 | 19 | 0.93 (0.90 ± 0.97) | 1.50 × 10^-4^ | 2.02 × 10^-2^ | rs6489896 | -929 |
| cg01814191 | 12 | 89,744,524 | intergenic | *LRRFIP2, LOC152048* | FHS | 0.12 | 65 | 0.93 (0.90 ± 0.96) | 4.58 × 10^-5^ | 8.45 × 10^-3^ | rs6489896 | 9,227 |
| cg01814191 | 12 | 89,744,524 | exonic | *DUSP6* | BIOS | 0.19 | 36 | 0.98 (0.97 ± 0.99) | 5.48 × 10^-4^ | 4.95 × 10^-2^ | rs6489896 | - |
| cg17740822 | 12 | 89,744,609 | exonic | *DUSP6* | BIOS | 0.16 | 40 | 0.97 (0.96 ± 0.99) | 3.80 × 10^-4^ | 3.85 × 10^-2^ | rs6489896 | -1,275 |
| cg10233654 | 12 | 89,744,621 | exonic | *DUSP6* | FHS | 0.03 | 23 | 0.87 (0.80 ± 0.94) | 2.80 × 10^-4^ | 3.10 × 10^-2^ | rs6489896 | 6,601 |
| cg05789837 | 12 | 89,747,417 | intergenic | *DUSP6, POC1B* | BIOS | 0.05 | 31 | 1.06 (1.03 ± 1.09) | 1.51 × 10^-4^ | 2.02 × 10^-2^ | rs6489896 | -1,071 |
| cg04822495 | 12 | 94,676,688 | intronic | *PLXNC1* | BIOS | 0.03 | 19 | 0.93 (0.89 ± 0.97) | 3.64 × 10^-4^ | 3.74 × 10^-2^ | rs6489896 | -1,030 |
| cg11080731 | 12 | 94,939,916 | intergenic | *CEP83-DT, MIR5700* | BIOS | 0.02 | 13 | 0.89 (0.85 ± 0.95) | 8.95 × 10^-5^ | 1.39 × 10^-2^ | rs6489896 | 6,271 |
| cg07197522 | 12 | 107,349,153 | upstream | *TMEM263* | BIOS | 0.03 | 10 | 0.92 (0.88 ± 0.96) | 2.30 × 10^-4^ | 2.72 × 10^-2^ | rs6489896 | -2,369 |
| cg06250904 | 12 | 107,351,181 | intronic | *TMEM263* | FHS | 0.02 | 16 | 1.22 (1.09 ± 1.36) | 3.01 × 10^-4^ | 3.29 × 10^-2^ | rs6489896 | 556 |
| cg19998289 | 12 | 121,861,616 | UTR3 | *RNF34* | FHS | 0.05 | 21 | 0.88 (0.82 ± 0.95) | 4.94 × 10^-4^ | 4.63 × 10^-2^ | rs6489896 | 1,126 |
| cg05298252 | 12 | 123,311,608 | UTR3 | *CCDC62* | BIOS | 0.07 | 7 | 1.05 (1.02 ± 1.07) | 1.41 × 10^-4^ | 1.95 × 10^-2^ | rs6489896 | -32,305 |
| cg05298252 | 12 | 123,311,608 | UTR3 | *CCDC62* | FHS | 0.05 | 8 | 1.10 (1.04 ± 1.17) | 4.93 × 10^-4^ | 4.62 × 10^-2^ | rs6489896 | 1,133 |
| cg21167157 | 12 | 123,326,461 | intronic | *HIP1R* | FHS | 0.04 | 18 | 1.18 (1.09 ± 1.27) | 6.26 × 10^-5^ | 1.07 × 10^-2^ | rs6489896 | -2,080 |
| cg13548946 | 12 | 123,350,077 | UTR3 | *VPS37B* | BIOS | 0.02 | 5 | 0.68 (0.55 ± 0.83) | 2.74 × 10^-4^ | 3.07 × 10^-2^ | rs6489896 | 10,300 |
| cg13548946 | 12 | 123,350,077 | UTR3 | *VPS37B* | FHS | 0.01 | 10 | 0.81 (0.73 ± 0.91) | 3.08 × 10^-4^ | 3.34 × 10^-2^ | rs6489896 | 8,894 |
| cg13299728 | 12 | 123,352,600 | intronic | *VPS37B* | BIOS | 0.01 | 4 | 1.12 (1.05 ± 1.19) | 3.63 × 10^-4^ | 3.73 × 10^-2^ | rs6489896 | 1,591 |
| cg22608507 | 12 | 123,379,308 | intronic | *VPS37B* | BIOS | 0.05 | 37 | 1.05 (1.02 ± 1.07) | 8.14 × 10^-5^ | 1.30 × 10^-2^ | rs6489896 | 6,283 |
| cg22608507 | 12 | 123,379,308 | intronic | *VPS37B* | FHS | 0.06 | 29 | 1.14 (1.06 ± 1.22) | 3.72 × 10^-4^ | 3.79 × 10^-2^ | rs6489896 | -1,210 |
| cg05298628 | 12 | 123,379,569 | intronic | *VPS37B* | FHS | 0.01 | 18 | 1.22 (1.10 ± 1.36) | 2.59 × 10^-4^ | 2.96 × 10^-2^ | rs6489896 | -12,198 |
| cg12095353 | 12 | 125,369,577 | intergenic | *SCARB1, UBC* | FHS | 0.01 | 12 | 1.25 (1.10 ± 1.42) | 5.43 × 10^-4^ | 4.93 × 10^-2^ | rs6489896 | -14,699 |
| cg14700609 | 13 | 24,463,713 | intronic | *PCOTH* | BIOS | 0.01 | 4 | 0.86 (0.78 ± 0.93) | 3.85 × 10^-4^ | 3.87 × 10^-2^ | - | - |
| cg03587877 | 13 | 24,471,371 | upstream | *C1QTNF9B* | FHS | 0.01 | 6 | 1.43 (1.17 ± 1.75) | 5.44 × 10^-4^ | 4.93 × 10^-2^ | - | - |
| cg26325430 | 13 | 33,639,059 | UTR3 | *KL* | BIOS | 0.05 | 52 | 1.06 (1.03 ± 1.09) | 3.07 × 10^-4^ | 3.34 × 10^-2^ | - | - |
| cg11582226 | 13 | 77,587,296 | intronic | *FBXL3* | BIOS | 0.06 | 16 | 1.04 (1.02 ± 1.05) | 1.49 × 10^-4^ | 2.01 × 10^-2^ | - | - |
| cg18011803 | 13 | 77,597,325 | intronic | *FBXL3* | FHS | 0.02 | 19 | 1.25 (1.11 ± 1.40) | 2.35 × 10^-4^ | 2.77 × 10^-2^ | - | - |
| cg06800231 | 14 | 24,398,826 | intergenic | *DHRS2, DHRS4-AS1* | FHS | 0.01 | 41 | 1.24 (1.12 ± 1.38) | 6.05 × 10^-5^ | 1.04 × 10^-2^ | rs7146179 | 13,423 |
| cg20908785 | 14 | 54,863,471 | upstream | *CDKN3* | BIOS | 0.01 | 6 | 0.89 (0.83 ± 0.95) | 5.40 × 10^-4^ | 4.91 × 10^-2^ | rs17125944 | -7,081 |
| cg14053764 | 14 | 55,761,432 | intronic | *FBXO34* | BIOS | 0.03 | 17 | 1.06 (1.03 ± 1.10) | 4.97 × 10^-4^ | 4.64 × 10^-2^ | rs17125944 | 1,126 |
| cg04853218 | 14 | 55,769,688 | intronic | *FBXO34* | BIOS | 0.17 | 7 | 1.03 (1.02 ± 1.05) | 6.77 × 10^-5^ | 1.13 × 10^-2^ | rs17125944 | 7,937 |
| cg04853218 | 14 | 55,769,688 | intronic | *FBXO34* | FHS | 0.13 | 27 | 1.07 (1.03 ± 1.11) | 3.42 × 10^-4^ | 3.60 × 10^-2^ | rs17125944 | 11,607 |
| cg12192145 | 14 | 55,849,528 | intronic | *ATG14* | FHS | 0.02 | 14 | 1.23 (1.10 ± 1.37) | 1.53 × 10^-4^ | 2.04 × 10^-2^ | rs17125944 | -1,229 |
| cg15390894 | 14 | 91,719,356 | exonic | *PPM1N* | FHS | 0.10 | 107 | 0.94 (0.91 ± 0.97) | 1.83 × 10^-5^ | 4.18 × 10^-3^ | rs10498633 | 2,770 |
| cg15390894 | 14 | 91,719,356 | intronic | *GPR68* | BIOS | 0.24 | 85 | 0.98 (0.97 ± 0.99) | 1.91 × 10^-4^ | 2.39 × 10^-2^ | rs10498633 | -1,100 |
| cg15742858 | 14 | 91,730,744 | intergenic | *GPR68, CCDC88C* | FHS | 0.13 | 3 | 1.08 (1.04 ± 1.13) | 1.10 × 10^-4^ | 1.62 × 10^-2^ | rs10498633 | 4,371 |
| cg10454568 | 14 | 96,890,632 | intronic | *AK7* | FHS | 0.01 | 36 | 0.80 (0.71 ± 0.90) | 1.84 × 10^-4^ | 2.33 × 10^-2^ | rs12590654 | -5,636 |
| cg06819357 | 14 | 102,928,437 | intronic | *TECPR2* | FHS | 0.08 | 96 | 0.93 (0.90 ± 0.97) | 5.28 × 10^-4^ | 4.83 × 10^-2^ | rs7157106 | 13,177 |
| cg00876272 | 14 | 103,265,614 | intronic | *TRAF3* | BIOS | 0.01 | 15 | 0.90 (0.84 ± 0.95) | 1.92 × 10^-4^ | 2.40 × 10^-2^ | rs7157106 | -1,413 |
| cg13996522 | 14 | 103,294,363 | intronic | *TRAF3* | BIOS | 0.02 | 14 | 0.88 (0.83 ± 0.93) | 2.45 × 10^-5^ | 5.24 × 10^-3^ | rs7157106 | -2,060 |
| cg23300486 | 14 | 103,294,375 | intronic | *TRAF3* | BIOS | 0.01 | 3 | 0.86 (0.80 ± 0.94) | 3.41 × 10^-4^ | 3.60 × 10^-2^ | rs7157106 | 11,607 |
| cg18185554 | 14 | 103,294,574 | intronic | *TRAF3* | BIOS | 0.01 | 16 | 0.90 (0.84 ± 0.95) | 4.99 × 10^-4^ | 4.65 × 10^-2^ | rs7157106 | 1,126 |
| cg26115667 | 14 | 103,294,656 | intronic | *TRAF3* | BIOS | 0.04 | 33 | 0.94 (0.91 ± 0.97) | 1.04 × 10^-4^ | 1.54 × 10^-2^ | rs7157106 | 6,261 |
| cg23520688 | 14 | 103,294,740 | intronic | *TRAF3* | BIOS | 0.04 | 31 | 0.93 (0.90 ± 0.96) | 5.30 × 10^-5^ | 9.37 × 10^-3^ | rs7157106 | 2,862 |
| cg24682159 | 14 | 104,026,912 | exonic | *BAG5* | BIOS | 0.01 | 25 | 0.89 (0.84 ± 0.95) | 1.87 × 10^-4^ | 2.36 × 10^-2^ | rs7157106 | -5,636 |
| cg21496948 | 14 | 104,153,614 | intronic | *KLC1* | BIOS | 0.04 | 8 | 0.94 (0.90 ± 0.97) | 1.24 × 10^-4^ | 1.75 × 10^-2^ | rs7157106 | 49,146 |
| cg21496948 | 14 | 104,153,614 | intronic | *KLC1* | FHS | 0.02 | 7 | 0.85 (0.77 ± 0.93) | 4.88 × 10^-4^ | 4.59 × 10^-2^ | rs7157106 | -3,978 |
| cg12950030 | 14 | 104,356,109 | intergenic | *LINC00637, ATP5MPL* | BIOS | 0.02 | 5 | 0.90 (0.85 ± 0.95) | 3.29 × 10^-4^ | 3.50 × 10^-2^ | rs7157106 | -2,758 |
| cg11092048 | 15 | 37,180,325 | intergenic | *LOC145845, MEIS2* | BIOS | 0.08 | 36 | 0.96 (0.94 ± 0.98) | 2.00 × 10^-4^ | 2.47 × 10^-2^ | rs442495 | 61,935 |
| cg13074682 | 15 | 40,113,167 | intronic | *GPR176* | BIOS | 0.18 | 16 | 0.97 (0.95 ± 0.98) | 2.63 × 10^-4^ | 2.98 × 10^-2^ | rs442495 | 29,609 |
| cg06232205 | 15 | 40,120,808 | intronic | *GPR176* | BIOS | 0.02 | 11 | 0.90 (0.84 ± 0.95) | 3.21 × 10^-4^ | 3.44 × 10^-2^ | rs442495 | 7,948 |
| cg17445845 | 15 | 50,280,871 | intronic | *ATP8B4* | BIOS | 0.01 | 10 | 1.13 (1.05 ± 1.21) | 5.14 × 10^-4^ | 4.74 × 10^-2^ | rs442495 | -910 |
| cg16831889 | 15 | 50,558,051 | upstream | *HDC* | FHS | 0.01 | 16 | 0.80 (0.71 ± 0.89) | 1.19 × 10^-4^ | 1.71 × 10^-2^ | rs442495 | 49,146 |
| cg06844932 | 15 | 50,716,705 | UTR3 | *SETD1A* | BIOS | 0.01 | 11 | 1.13 (1.07 ± 1.19) | 2.75 × 10^-5^ | 5.72 × 10^-3^ | rs442495 | -43,094 |
| cg09837656 | 15 | 65,149,245 | intronic | *PLEKHO2* | BIOS | 0.43 | 24 | 0.98 (0.98 ± 0.99) | 3.61 × 10^-4^ | 3.72 × 10^-2^ | rs3848143 | 1,593 |
| cg24360197 | 15 | 66,662,775 | intergenic | *TIPIN, MAP2K1* | FHS | 0.01 | 9 | 1.39 (1.18 ± 1.65) | 1.27 × 10^-4^ | 1.78 × 10^-2^ | rs3848143 | 49,146 |
| cg15808604 | 15 | 74,419,428 | ncRNA_exonic | *LOC283731* | BIOS | 0.02 | 11 | 0.93 (0.90 ± 0.97) | 5.39 × 10^-4^ | 4.91 × 10^-2^ | rs12592898 | 1,424 |
| cg16906765 | 15 | 75,475,038 | intergenic | *PPCDC, C15orf39* | FHS | 0.03 | 1 | 0.81 (0.73 ± 0.91) | 1.76 × 10^-4^ | 2.26 × 10^-2^ | rs12592898 | -2,354 |
| cg10767818 | 15 | 90,806,036 | intergenic | *TTLL13P, NGRN* | FHS | 0.01 | 46 | 1.20 (1.10 ± 1.30) | 2.85 × 10^-5^ | 5.85 × 10^-3^ | rs12592898 | 14,417 |
| cg27310024 | 15 | 90,950,101 | intronic | *IQGAP1* | FHS | 0.04 | 30 | 1.12 (1.05 ± 1.19) | 4.89 × 10^-4^ | 4.60 × 10^-2^ | rs12592898 | -3,985 |
| cg25647583 | 15 | 91,427,184 | upstream, downstream | *FES, FURIN* | BIOS | 0.04 | 16 | 0.93 (0.89 ± 0.97) | 5.48 × 10^-4^ | 4.95 × 10^-2^ | rs12592898 | - |
| cg00882281 | 15 | 96,346,592 | intergenic | *LINC00924, LOC105369212* | FHS | 0.47 | 15 | 0.97 (0.95 ± 0.99) | 3.27 × 10^-4^ | 3.48 × 10^-2^ | rs12592898 | -2,533 |
| cg03397724 | 16 | 375,541 | intronic | *AXIN1* | BIOS | 0.05 | 18 | 1.07 (1.03 ± 1.11) | 2.04 × 10^-4^ | 2.51 × 10^-2^ | rs1140239 | 23,975 |
| cg02870744 | 16 | 412,727 | intergenic | *AXIN1, MRPL28* | BIOS | 0.14 | 22 | 0.96 (0.94 ± 0.98) | 3.10 × 10^-4^ | 3.36 × 10^-2^ | rs1140239 | 8,871 |
| cg10805676 | 16 | 421,990 | exonic | *TMEM8A* | FHS | 0.13 | 21 | 0.91 (0.87 ± 0.96) | 2.25 × 10^-4^ | 2.69 × 10^-2^ | rs1140239 | - |
| cg04168554 | 16 | 572,062 | UTR3 | *RAB11FIP3* | BIOS | 0.13 | 31 | 1.04 (1.02 ± 1.07) | 2.55 × 10^-4^ | 2.93 × 10^-2^ | rs1140239 | 8,306 |
| cg05646515 | 16 | 964,974 | intronic | *LMF1* | BIOS | 0.02 | 13 | 0.92 (0.87 ± 0.96) | 3.09 × 10^-4^ | 3.34 × 10^-2^ | rs1140239 | 8,888 |
| cg04122657 | 16 | 4,014,295 | UTR3 | *ADCY9* | BIOS | 0.03 | 21 | 0.93 (0.89 ± 0.97) | 4.46 × 10^-4^ | 4.31 × 10^-2^ | rs1140239 | -1,581 |
| cg05249026 | 16 | 11,031,524 | intronic | *DEXI* | FHS | 0.07 | 19 | 1.11 (1.05 ± 1.17) | 1.68 × 10^-4^ | 2.18 × 10^-2^ | rs1140239 | 31,643 |
| cg09770579 | 16 | 17,407,429 | intronic | *XYLT1* | BIOS | 0.02 | 34 | 1.09 (1.05 ± 1.14) | 4.62 × 10^-5^ | 8.49 × 10^-3^ | rs1140239 | 9,199 |
| cg06321596 | 16 | 17,562,960 | intronic | *XYLT1* | BIOS | 0.14 | 75 | 1.03 (1.02 ± 1.05) | 1.83 × 10^-4^ | 2.33 × 10^-2^ | rs1140239 | -4,087 |
| cg01724917 | 16 | 17,563,028 | intronic | *XYLT1* | BIOS | 0.11 | 33 | 1.05 (1.02 ± 1.07) | 8.62 × 10^-5^ | 1.35 × 10^-2^ | rs1140239 | 6,279 |
| cg04359840 | 16 | 17,563,300 | intronic | *XYLT1* | BIOS | 0.18 | 46 | 1.03 (1.01 ± 1.04) | 1.99 × 10^-4^ | 2.47 × 10^-2^ | rs1140239 | -8,825 |
| cg06129210 | 16 | 17,565,129 | upstream | *XYLT1* | BIOS | 0.21 | 52 | 0.98 (0.96 ± 0.99) | 5.41 × 10^-4^ | 4.92 × 10^-2^ | rs1140239 | -7,117 |
| cg06685949 | 16 | 19,721,858 | exonic | *KNOP1* | FHS | 0.01 | 14 | 0.70 (0.57 ± 0.84) | 2.27 × 10^-4^ | 2.70 × 10^-2^ | rs1140239 | - |
| cg01578585 | 16 | 19,721,878 | intergenic | *SLC43A2, SCARF1* | FHS | 0.04 | 11 | 0.82 (0.74 ± 0.90) | 3.00 × 10^-5^ | 6.08 × 10^-3^ | rs1140239 | -19,778 |
| cg07736657 | 16 | 19,777,512 | intronic | *IQCK* | BIOS | 0.56 | 10 | 0.98 (0.98 ± 0.99) | 2.72 × 10^-5^ | 5.70 × 10^-3^ | rs1140239 | -43,094 |
| cg07736657 | 16 | 19,777,512 | intronic | *IQCK* | FHS | 0.57 | 45 | 0.96 (0.95 ± 0.98) | 4.61 × 10^-5^ | 8.48 × 10^-3^ | rs1140239 | 9,199 |
| cg07501827 | 16 | 23,392,215 | UTR3 | *SCNN1B* | FHS | 0.02 | 5 | 0.75 (0.66 ± 0.85) | 6.62 × 10^-6^ | 1.81 × 10^-3^ | rs1140239 | -32,762 |
| cg07501827 | 16 | 23,392,215 | downstream | *LYPD5* | BIOS | 0.07 | 7 | 0.91 (0.87 ± 0.95) | 3.07 × 10^-5^ | 6.18 × 10^-3^ | rs1140239 | -23,228 |
| cg07355514 | 16 | 23,420,385 | intronic | *COG7* | BIOS | 0.19 | 16 | 1.03 (1.02 ± 1.05) | 4.94 × 10^-5^ | 8.90 × 10^-3^ | rs1140239 | 2,015 |
| cg04723449 | 16 | 23,420,567 | intronic | *COG7* | BIOS | 0.08 | 6 | 1.05 (1.02 ± 1.07) | 7.38 × 10^-5^ | 1.21 × 10^-2^ | rs1140239 | -54,989 |
| cg09934309 | 16 | 23,461,721 | intronic | *COG7* | BIOS | 0.01 | 4 | 1.18 (1.09 ± 1.27) | 4.96 × 10^-5^ | 8.93 × 10^-3^ | rs1140239 | 1,998 |
| cg08124317 | 16 | 23,478,837 | UTR3 | *GGA2* | BIOS | 0.01 | 8 | 1.11 (1.05 ± 1.17) | 1.23 × 10^-4^ | 1.74 × 10^-2^ | rs1140239 | 49,146 |
| cg00736406 | 16 | 23,504,930 | intronic | *GGA2* | BIOS | 0.01 | 4 | 0.86 (0.81 ± 0.92) | 6.14 × 10^-6^ | 1.71 × 10^-3^ | rs1140239 | -32,761 |
| cg03935956 | 16 | 23,511,855 | intronic | *GGA2* | BIOS | 0.23 | 7 | 0.97 (0.96 ± 0.99) | 8.24 × 10^-5^ | 1.31 × 10^-2^ | rs1140239 | 6,280 |
| cg03935956 | 16 | 23,511,855 | intronic | *GGA2* | FHS | 0.16 | 55 | 0.94 (0.91 ± 0.97) | 2.44 × 10^-4^ | 2.84 × 10^-2^ | rs1140239 | 2,934 |
| cg04666465 | 16 | 23,520,563 | intronic | *GGA2* | FHS | 0.38 | 35 | 1.04 (1.02 ± 1.07) | 1.46 × 10^-5^ | 3.51 × 10^-3^ | rs1140239 | 20,007 |
| cg04666465 | 16 | 23,520,563 | intronic | *GGA2* | BIOS | 0.50 | 26 | 1.02 (1.01 ± 1.03) | 2.23 × 10^-5^ | 4.84 × 10^-3^ | rs1140239 | -29,150 |
| cg07150862 | 16 | 23,520,705 | intronic | *GGA2* | BIOS | 0.43 | 38 | 1.02 (1.01 ± 1.03) | 1.09 × 10^-5^ | 2.73 × 10^-3^ | rs1140239 | 20,188 |
| cg07150862 | 16 | 23,520,705 | intronic | *STX1B* | FHS | 0.32 | 43 | 1.05 (1.03 ± 1.07) | 1.95 × 10^-5^ | 4.39 × 10^-3^ | rs1140239 | -29,149 |
| cg09645925 | 16 | 30,577,298 | intergenic | *ZNF764, ZNF688* | BIOS | 0.04 | 19 | 1.08 (1.04 ± 1.12) | 1.77 × 10^-4^ | 2.27 × 10^-2^ | rs59735493 | -4,005 |
| cg10967191 | 16 | 49,888,604 | intronic | *ZNF423* | BIOS | 0.15 | 55 | 1.03 (1.01 ± 1.05) | 5.25 × 10^-4^ | 4.82 × 10^-2^ | rs59735493 | -593 |
| cg01266287 | 16 | 66,959,833 | intergenic | *SMG1P2, SPN* | BIOS | 0.04 | 24 | 0.94 (0.92 ± 0.97) | 2.92 × 10^-5^ | 5.97 × 10^-3^ | rs450674 | 13,499 |
| cg08281777 | 16 | 70,679,178 | intronic | *IL34* | BIOS | 0.04 | 16 | 1.07 (1.04 ± 1.11) | 6.97 × 10^-5^ | 1.16 × 10^-2^ | rs450674 | -18,298 |
| cg26831220 | 16 | 70,680,406 | intronic | *IL34* | BIOS | 0.01 | 11 | 1.11 (1.05 ± 1.17) | 2.68 × 10^-4^ | 3.02 × 10^-2^ | rs450674 | 26,007 |
| cg00147286 | 16 | 70,713,933 | exonic | *MTSS2* | FHS | 0.03 | 12 | 1.16 (1.07 ± 1.25) | 3.35 × 10^-4^ | 3.55 × 10^-2^ | rs450674 | -10,869 |
| cg01304894 | 16 | 70,715,140 | intronic | *MTSS2* | BIOS | 0.02 | 18 | 1.10 (1.05 ± 1.14) | 2.56 × 10^-6^ | 8.36 × 10^-4^ | rs450674 | 12,906 |
| cg07838048 | 16 | 70,720,440 | upstream, downstream | *MTSS2, VAC14* | FHS | 0.02 | 16 | 0.85 (0.77 ± 0.93) | 4.29 × 10^-4^ | 4.21 × 10^-2^ | rs450674 | -1,903 |
| cg08843902 | 16 | 70,737,371 | intronic | *VAC14* | BIOS | 0.03 | 31 | 1.06 (1.03 ± 1.09) | 9.79 × 10^-6^ | 2.50 × 10^-3^ | rs450674 | 20,241 |
| cg00259097 | 16 | 70,770,604 | intronic | *VAC14* | BIOS | 0.05 | 23 | 1.06 (1.03 ± 1.09) | 1.99 × 10^-4^ | 2.47 × 10^-2^ | rs450674 | 106,789 |
| cg09794131 | 16 | 71,264,658 | exonic | *SPATA2L* | BIOS | 0.01 | 5 | 1.18 (1.09 ± 1.28) | 8.73 × 10^-5^ | 1.37 × 10^-2^ | rs450674 | 6,276 |
| cg02616906 | 16 | 71,265,770 | intergenic | *HYDIN, CMTR2* | FHS | 0.08 | 18 | 1.10 (1.04 ± 1.15) | 1.64 × 10^-4^ | 2.14 × 10^-2^ | rs450674 | 3,191 |
| cg08769634 | 16 | 71,657,362 | intergenic | *TAT, MARVELD3* | FHS | 0.06 | 17 | 0.91 (0.87 ± 0.96) | 1.26 × 10^-4^ | 1.78 × 10^-2^ | rs450674 | 49,146 |
| cg09326345 | 16 | 71,660,044 | intronic | *INSRR, NTRK1* | BIOS | 0.01 | 6 | 0.87 (0.82 ± 0.94) | 9.11 × 10^-5^ | 1.40 × 10^-2^ | rs450674 | 6,269 |
| cg06952752 | 16 | 71,660,908 | exonic | *DDX39B* | BIOS | 0.04 | 16 | 1.07 (1.04 ± 1.11) | 8.81 × 10^-5^ | 1.37 × 10^-2^ | rs450674 | 6,274 |
| cg05708656 | 16 | 71,792,305 | ncRNA_exonic | *SNORD71* | BIOS | 0.02 | 9 | 0.86 (0.80 ± 0.93) | 2.37 × 10^-4^ | 2.78 × 10^-2^ | rs450674 | 2,962 |
| cg05550612 | 16 | 74,641,310 | upstream | *GLG1* | BIOS | 0.02 | 10 | 0.90 (0.85 ± 0.95) | 3.84 × 10^-4^ | 3.86 × 10^-2^ | rs450674 | -1,421 |
| cg10001987 | 17 | 7,381,769 | intronic | *ZBTB4* | FHS | 0.02 | 20 | 0.83 (0.74 ± 0.92) | 5.28 × 10^-4^ | 4.83 × 10^-2^ | rs9916042 | 19,078 |
| cg00688810 | 17 | 7,517,138 | intronic | *FXR2* | FHS | 0.03 | 32 | 0.87 (0.80 ± 0.94) | 3.69 × 10^-4^ | 3.77 × 10^-2^ | rs9916042 | -1,136 |
| cg24893073 | 17 | 7,742,126 | intergenic | *DNAH2, KDM6B* | BIOS | 0.03 | 20 | 1.08 (1.03 ± 1.12) | 2.99 × 10^-4^ | 3.27 × 10^-2^ | rs9916042 | 6,501 |
| cg01046511 | 17 | 7,742,971 | upstream | *KDM6B* | BIOS | 0.09 | 44 | 1.05 (1.03 ± 1.07) | 5.94 × 10^-6^ | 1.68 × 10^-3^ | rs9916042 | -32,761 |
| cg01757806 | 17 | 8,378,795 | UTR3 | *MYH10* | BIOS | 0.03 | 25 | 1.09 (1.04 ± 1.14) | 4.08 × 10^-4^ | 4.06 × 10^-2^ | rs9916042 | -1,663 |
| cg19048010 | 17 | 28,084,996 | intronic | *SSH2* | FHS | 0.10 | 67 | 1.07 (1.03 ± 1.11) | 3.39 × 10^-4^ | 3.58 × 10^-2^ | rs2242595 | 11,607 |
| cg27034606 | 17 | 28,928,453 | ncRNA_intronic | *SMURF2P1-LRRC37BP1* | FHS | 0.11 | 25 | 0.93 (0.89 ± 0.97) | 4.70 × 10^-4^ | 4.46 × 10^-2^ | rs2242595 | -3,965 |
| cg03898962 | 17 | 30,815,228 | exonic | *CDK5R1* | FHS | 0.12 | 10 | 1.07 (1.03 ± 1.11) | 5.35 × 10^-4^ | 4.89 × 10^-2^ | rs5848 | 10,965 |
| cg18200150 | 17 | 30,822,561 | intronic | *MYO1D* | FHS | 0.71 | 75 | 0.98 (0.96 ± 0.99) | 2.11 × 10^-4^ | 2.57 × 10^-2^ | rs5848 | -8,142 |
| cg18200150 | 17 | 30,822,561 | intronic | *MYO1D* | BIOS | 0.73 | 48 | 0.98 (0.98 ± 0.99) | 5.11 × 10^-4^ | 4.73 × 10^-2^ | rs5848 | 957 |
| cg25809561 | 17 | 30,822,961 | intronic | *MYO1D* | FHS | 0.32 | 20 | 0.96 (0.94 ± 0.98) | 1.76 × 10^-4^ | 2.26 × 10^-2^ | rs5848 | -754 |
| cg25809561 | 17 | 30,822,961 | intronic | *MYO1D* | BIOS | 0.38 | 14 | 0.98 (0.97 ± 0.99) | 5.40 × 10^-4^ | 4.92 × 10^-2^ | rs5848 | -7,081 |
| cg09324608 | 17 | 30,823,087 | intronic | *MYO1D* | FHS | 0.37 | 22 | 0.96 (0.94 ± 0.98) | 2.23 × 10^-4^ | 2.68 × 10^-2^ | rs5848 | -11,650 |
| cg09324608 | 17 | 30,823,087 | intronic | *MYO1D* | BIOS | 0.37 | 41 | 0.98 (0.97 ± 0.99) | 2.64 × 10^-4^ | 2.99 × 10^-2^ | rs5848 | 29,599 |
| cg23550016 | 17 | 37,821,447 | upstream, downstream | *TCAP, STARD3* | BIOS | 0.02 | 3 | 1.11 (1.05 ± 1.18) | 3.86 × 10^-4^ | 3.88 × 10^-2^ | rs5848 | -1,530 |
| cg02944084 | 17 | 37,827,057 | intergenic | *LINC00533, LINC01623* | BIOS | 0.10 | 20 | 0.95 (0.93 ± 0.97) | 5.93 × 10^-5^ | 1.02 × 10^-2^ | rs5848 | 14,191 |
| cg02944084 | 17 | 37,827,057 | downstream | *PGAP3, PNMT* | FHS | 0.03 | 22 | 0.85 (0.79 ± 0.93) | 1.89 × 10^-4^ | 2.37 × 10^-2^ | rs5848 | -1,092 |
| cg19758448 | 17 | 37,828,296 | UTR3 | *PGAP3* | BIOS | 0.42 | 6 | 1.02 (1.01 ± 1.03) | 2.87 × 10^-4^ | 3.17 × 10^-2^ | rs5848 | 6,510 |
| cg19758448 | 17 | 37,828,296 | UTR3 | *PGAP3* | FHS | 0.31 | 19 | 1.04 (1.02 ± 1.07) | 3.69 × 10^-4^ | 3.77 × 10^-2^ | rs5848 | -1,136 |
| cg15227682 | 17 | 37,843,264 | intronic | *PGAP3* | BIOS | 0.02 | 3 | 1.12 (1.06 ± 1.20) | 2.78 × 10^-4^ | 3.09 × 10^-2^ | rs5848 | 6,629 |
| cg15227682 | 17 | 37,843,264 | intronic | *PGAP3* | FHS | 0.02 | 12 | 1.25 (1.11 ± 1.42) | 3.61 × 10^-4^ | 3.72 × 10^-2^ | rs5848 | 1,597 |
| cg26615017 | 17 | 37,879,657 | exonic | *ERBB2* | FHS | 0.04 | 15 | 0.88 (0.83 ± 0.95) | 3.76 × 10^-4^ | 3.82 × 10^-2^ | rs5848 | -1,237 |
| cg16557858 | 17 | 37,879,740 | intronic | *ERBB2* | FHS | 0.06 | 23 | 0.90 (0.85 ± 0.95) | 4.97 × 10^-4^ | 4.64 × 10^-2^ | rs5848 | 1,126 |
| cg13432737 | 17 | 37,922,420 | exonic | *IKZF3* | BIOS | 0.01 | 6 | 0.86 (0.79 ± 0.94) | 4.75 × 10^-4^ | 4.50 × 10^-2^ | rs5848 | -3,968 |
| cg23207054 | 17 | 38,171,530 | upstream | *CSF3* | BIOS | 0.05 | 12 | 1.06 (1.03 ± 1.09) | 1.92 × 10^-4^ | 2.39 × 10^-2^ | rs5848 | -1,217 |
| cg24704510 | 17 | 38,182,512 | exonic | *MED24* | BIOS | 0.01 | 13 | 1.12 (1.06 ± 1.19) | 5.03 × 10^-5^ | 8.98 × 10^-3^ | rs5848 | 1,998 |
| cg15418287 | 17 | 40,706,385 | ncRNA_exonic | *LOC108783654* | BIOS | 0.03 | 2 | 0.92 (0.88 ± 0.96) | 5.23 × 10^-4^ | 4.80 × 10^-2^ | rs5848 | -1,264 |
| cg00685795 | 17 | 40,713,781 | upstream | *COASY* | FHS | 0.04 | 20 | 1.12 (1.06 ± 1.20) | 2.59 × 10^-4^ | 2.96 × 10^-2^ | rs5848 | -11,721 |
| cg00685795 | 17 | 40,713,781 | upstream | *COASY* | BIOS | 0.07 | 6 | 1.06 (1.03 ± 1.01) | 5.22 × 10^-4^ | 4.80 × 10^-2^ | rs5848 | -1,264 |
| cg24455236 | 17 | 40,824,361 | exonic | *PLEKHH3* | FHS | 0.06 | 67 | 1.09 (1.04 ± 1.14) | 1.46 × 10^-4^ | 1.99 × 10^-2^ | rs5848 | 1,714 |
| cg09509673 | 17 | 40,833,697 | intronic | *CCR10* | FHS | 0.05 | 16 | 0.89 (0.84 ± 0.95) | 1.54 × 10^-4^ | 2.05 × 10^-2^ | rs5848 | -4,703 |
| cg21433558 | 17 | 40,837,037 | exonic | *CNTNAP1* | FHS | 0.48 | 24 | 1.03 (1.01 ± 1.05) | 4.17 × 10^-4^ | 4.13 × 10^-2^ | rs5848 | -1,807 |
| cg12559685 | 17 | 40,839,469 | intronic | *CNTNAP1* | FHS | 0.04 | 30 | 1.14 (1.06 ± 1.22) | 1.91 × 10^-4^ | 2.39 × 10^-2^ | rs5848 | -1,217 |
| cg21946667 | 17 | 40,839,643 | intronic | *CNTNAP1* | BIOS | 0.01 | 7 | 1.12 (1.05 ± 1.19) | 3.11 × 10^-4^ | 3.36 × 10^-2^ | rs5848 | 8,838 |
| cg07871971 | 17 | 40,839,766 | exonic | *CNTNAP1* | FHS | 0.04 | 16 | 1.17 (1.07 ± 1.27) | 2.53 × 10^-4^ | 2.92 × 10^-2^ | rs5848 | 2,201 |
| cg18410271 | 17 | 43,472,435 | UTR3 | *ARHGAP27* | FHS | 0.11 | 35 | 0.92 (0.89 ± 0.96) | 1.95 × 10^-4^ | 2.42 × 10^-2^ | rs708382 | -1,961 |
| cg23659289 | 17 | 43,472,725 | UTR3 | *ARHGAP27* | FHS | 0.14 | 52 | 0.89 (0.86 ± 0.93) | **1.49 × 10^-7^** | 7.92 × 10^-5^ | rs708382 | 4,339 |
| cg00715050 | 17 | 43,472,920 | exonic | *ARHGAP27* | FHS | 0.02 | 20 | 0.68 (0.59 ± 0.79) | 3.24 × 10^-7^ | 1.53 × 10^-4^ | rs708382 | 2,661 |
| cg07067577 | 17 | 43,506,829 | UTR3 | *ARHGAP27* | FHS | 0.07 | 16 | 1.13 (1.07 ± 1.19) | 2.32 × 10^-6^ | 7.67 × 10^-4^ | rs708382 | -5,600 |
| cg08113562 | 17 | 43,508,428 | intronic | *ARHGAP27* | FHS | 0.57 | 42 | 0.96 (0.95 ± 0.98) | 1.54 × 10^-6^ | 5.59 × 10^-4^ | rs708382 | 9,202 |
| cg16281322 | 17 | 43,510,478 | upstream | *ARHGAP27* | FHS | 0.29 | 11 | 1.06 (1.03 ± 1.08) | 1.72 × 10^-6^ | 6.15 × 10^-4^ | rs708382 | 9,104 |
| cg25708777 | 17 | 43,510,841 | upstream | *ARHGAP27* | FHS | 0.02 | 34 | 1.14 (1.06 ± 1.22) | 1.74 × 10^-4^ | 2.25 × 10^-2^ | rs708382 | 3,811 |
| cg26471390 | 17 | 43,511,301 | intergenic | *ARHGAP27, PLEKHM1* | FHS | 0.27 | 36 | 0.94 (0.92 ± 0.96) | **1.77 × 10^-7^** | 9.03 × 10^-5^ | rs708382 | -551 |
| cg06925179 | 17 | 43,578,568 | intergenic | *PLEKHM1, ARL17B* | FHS | 0.22 | 34 | 0.94 (0.92 ± 0.97) | 3.50 × 10^-6^ | 1.08 × 10^-3^ | rs708382 | -32,573 |
| cg04703951 | 17 | 43,578,652 | intergenic | *PLEKHM1, ARL17B* | FHS | 0.41 | 102 | 0.95 (0.94 ± 0.97) | 2.75 × 10^-6^ | 8.86 × 10^-4^ | rs708382 | 11,130 |
| cg03915738 | 17 | 43,651,976 | intergenic | *ARL17A, MAPK8IP1P2* | FHS | 0.35 | 51 | 0.94 (0.91 ± 0.96) | **4.82 × 10^-8^** | 3.21 × 10^-5^ | rs708382 | 4,459 |
| cg12609785 | 17 | 43,660,871 | intergenic | *ARL17A, MAPK8IP1P2* | FHS | 0.61 | 37 | 0.76 (0.70 ± 0.83) | **1.86 × 10^-9^** | 1.90 × 10^-6^ | rs708382 | -5,696 |
| cg01341218 | 17 | 43,662,625 | intergenic | *ARL17A, MAPK8IP1P2* | FHS | 0.02 | 22 | 0.80 (0.71 ± 0.90) | 2.00 × 10^-4^ | 2.47 × 10^-2^ | rs708382 | 57,058 |
| cg04927033 | 17 | 43,679,265 | upstream | *MAPK8IP1P2* | FHS | 0.16 | 48 | 1.26 (1.16 ± 1.38) | **1.73 × 10^-7^** | 8.88 × 10^-5^ | rs708382 | -551 |
| cg23590916 | 17 | 43,697,445 | upstream | *LINC02210, LINC02210-CRHR1* | FHS | 0.08 | 22 | 1.39 (1.23 ± 1.56) | **6.53 × 10^-8^** | 4.05 × 10^-5^ | rs708382 | 4,437 |
| cg15413793 | 17 | 43,700,761 | ncRNA_intronic | *LINC02210* | FHS | 0.07 | 31 | 1.13 (1.05 ± 1.21) | 5.13 × 10^-4^ | 4.74 × 10^-2^ | rs708382 | 957 |
| cg03954353 | 17 | 43,715,162 | ncRNA_exonic | *LINC02210* | FHS | 0.18 | 98 | 0.90 (0.87 ± 0.94) | **1.50 × 10^-7^** | 7.94 × 10^-5^ | rs708382 | 538 |
| cg01882395 | 17 | 43,717,810 | ncRNA_exonic | *LINC02210* | FHS | 0.15 | 45 | 0.91 (0.88 ± 0.95) | 3.52 × 10^-7^ | 1.62 × 10^-4^ | rs708382 | 2,533 |
| cg27551605 | 17 | 43,862,910 | intronic | *CRHR1, LINC02210-CRHR1* | FHS | 0.02 | 13 | 6.11 (2.83 ± 13.19) | 3.99 × 10^-6^ | 1.20 × 10^-3^ | rs708382 | -32,580 |
| cg07778819 | 17 | 43,862,927 | intronic | *CRHR1, LINC02210-CRHR1* | FHS | 0.01 | 9 | 1.50 (1.26 ± 1.78) | 4.09 × 10^-6^ | 1.23 × 10^-3^ | rs708382 | -32,580 |
| cg24063856 | 17 | 43,863,303 | intronic | *CRHR1, LINC02210-CRHR1* | FHS | 0.08 | 28 | 1.14 (1.08 ± 1.21) | 9.66 × 10^-6^ | 2.47 × 10^-3^ | rs708382 | 20,260 |
| cg00025823 | 17 | 43,909,151 | UTR5 | *ZNF232* | FHS | 0.03 | 9 | 0.82 (0.75 ± 0.89) | 3.23 × 10^-6^ | 1.01 × 10^-3^ | rs708382 | -565 |
| cg15072306 | 17 | 43,922,875 | exonic | *SPPL2C* | FHS | 0.03 | 27 | 1.17 (1.08 ± 1.26) | 6.35 × 10^-5^ | 1.07 × 10^-2^ | rs708382 | 1,806 |
| cg05301556 | 17 | 43,971,177 | ncRNA_intronic | *MAPT-AS1* | FHS | 0.18 | 47 | 1.10 (1.06 ± 1.14) | 4.21 × 10^-7^ | 1.87 × 10^-4^ | rs708382 | -16,798 |
| cg00891649 | 17 | 43,972,573 | ncRNA_intronic | *MAPT-AS1* | FHS | 0.12 | 35 | 1.14 (1.09 ± 1.20) | **5.40 × 10^-8^** | 3.52 × 10^-5^ | rs708382 | 4,459 |
| cg18878992 | 17 | 43,974,344 | ncRNA_exonic | *MAPT-IT1* | FHS | 0.14 | 23 | 0.85 (0.81 ± 0.90) | **1.02 × 10^-8^** | 8.23 × 10^-6^ | rs708382 | -19,681 |
| cg10955972 | 17 | 43,976,002 | ncRNA_exonic | *MAPT-IT1* | FHS | 0.02 | 16 | 0.60 (0.49 ± 0.73) | 7.82 × 10^-7^ | 3.18 × 10^-4^ | rs708382 | -16,825 |
| cg22291189 | 17 | 43,978,251 | intronic | *MAPT* | FHS | 0.01 | 6 | 1.34 (1.14 ± 1.58) | 5.01 × 10^-4^ | 4.66 × 10^-2^ | rs708382 | 1,112 |
| cg05772917 | 17 | 44,027,251 | intronic | *MAPT* | FHS | 0.03 | 18 | 0.74 (0.64 ± 0.86) | 5.27 × 10^-5^ | 9.33 × 10^-3^ | rs708382 | 2,862 |
| cg03836283 | 17 | 44,058,856 | intronic | *MAPT* | FHS | 0.03 | 12 | 0.71 (0.62 ± 0.81) | 1.11 × 10^-6^ | 4.26 × 10^-4^ | rs708382 | -36,256 |
| cg01934064 | 17 | 44,064,242 | intronic | *MAPT* | FHS | 0.21 | 138 | 0.91 (0.88 ± 0.94) | **6.80 × 10^-9^** | 5.80 × 10^-6^ | rs708382 | -7,499 |
| cg00480298 | 17 | 44,068,857 | exonic | *MAPT* | FHS | 0.02 | 14 | 0.82 (0.75 ± 0.90) | 2.27 × 10^-5^ | 4.91 × 10^-3^ | rs708382 | -29,150 |
| cg24677220 | 17 | 44,075,684 | intronic | *MAPT* | FHS | 0.01 | 11 | 0.66 (0.57 ± 0.76) | **3.87 × 10^-8^** | 2.67 × 10^-5^ | rs708382 | -40,263 |
| cg07368061 | 17 | 44,090,862 | intronic | *MAPT* | FHS | 0.43 | 56 | 1.09 (1.05 ± 1.12) | **1.45 × 10^-7^** | 7.80 × 10^-5^ | rs708382 | 4,340 |
| cg09764761 | 17 | 44,105,544 | UTR3 | *MAPT* | FHS | 0.38 | 104 | 1.07 (1.05 ± 1.10) | **5.57 × 10^-8^** | 3.58 × 10^-5^ | rs708382 | 4,444 |
| cg13732302 | 17 | 44,222,207 | intergenic | *LRRFIP2, LOC152048* | FHS | 0.17 | 74 | 1.07 (1.04 ± 1.11) | 3.68 × 10^-5^ | 7.15 × 10^-3^ | rs708382 | 10,912 |
| cg21214508 | 17 | 44,248,233 | exonic | *KANSL1* | FHS | 0.16 | 84 | 1.08 (1.04 ± 1.12) | 7.82 × 10^-5^ | 1.26 × 10^-2^ | rs708382 | 6,288 |
| cg02301815 | 17 | 44,249,491 | exonic | *KANSL1* | FHS | 0.13 | 41 | 1.15 (1.08 ± 1.22) | 1.59 × 10^-5^ | 3.76 × 10^-3^ | rs708382 | 19,968 |
| cg19832721 | 17 | 44,249,866 | intronic | *KANSL1* | FHS | 0.66 | 45 | 0.95 (0.94 ± 0.97) | **1.11 × 10^-7^** | 6.39 × 10^-5^ | rs708382 | 4,371 |
| cg19976404 | 17 | 44,250,487 | upstream | *TSPO2* | FHS | 0.11 | 33 | 1.12 (1.06 ± 1.17) | 1.06 × 10^-5^ | 2.66 × 10^-3^ | rs708382 | 20,208 |
| cg08862499 | 17 | 44,271,469 | ncRNA_intronic | *KANSL1-AS1* | FHS | 0.06 | 26 | 1.13 (1.06 ± 1.21) | 3.86 × 10^-4^ | 3.88 × 10^-2^ | rs708382 | -1,529 |
| cg06462185 | 17 | 44,305,081 | intergenic | *KANSL1, ARL17B* | FHS | 0.04 | 20 | 1.18 (1.10 ± 1.27) | 7.87 × 10^-6^ | 2.10 × 10^-3^ | rs708382 | -73,843 |
| cg06291494 | 17 | 44,321,403 | intergenic | *KANSL1, ARL17B* | FHS | 0.14 | 37 | 1.18 (1.11 ± 1.25) | **2.73 × 10^-8^** | 1.98 × 10^-5^ | rs708382 | -23,311 |
| cg14517863 | 17 | 44,321,492 | intergenic | *KANSL1, ARL17B* | FHS | 0.40 | 50 | 0.93 (0.91 ± 0.95) | **6.11 × 10^-9^** | 5.29 × 10^-6^ | rs708382 | -7,499 |
| cg08670715 | 17 | 44,341,754 | intergenic | *KANSL1, ARL17B* | FHS | 0.37 | 36 | 0.89 (0.86 ± 0.93) | **2.80 × 10^-9^** | 2.72 × 10^-6^ | rs708382 | -7,471 |
| cg05159804 | 17 | 44,343,776 | intergenic | *KANSL1, ARL17B* | FHS | 0.61 | 87 | 1.11 (1.07 ± 1.15) | **2.19 × 10^-7^** | 1.09 × 10^-4^ | rs708382 | -19,902 |
| cg18027529 | 17 | 44,344,864 | intergenic | *KANSL1, ARL17B* | FHS | 0.24 | 44 | 0.90 (0.87 ± 0.93) | **2.80 × 10^-8^** | 2.02 × 10^-5^ | rs708382 | -27,779 |
| cg27388763 | 17 | 44,648,385 | intronic | *ARL17A, ARL17B* | FHS | 0.05 | 28 | 1.12 (1.05 ± 1.19) | 2.08 × 10^-4^ | 2.54 × 10^-2^ | rs708382 | 18,780 |
| cg24910739 | 17 | 44,657,394 | upstream | *ARL17A, ARL17B* | FHS | 0.02 | 10 | 0.67 (0.58 ± 0.78) | **3.49 × 10^-7^** | 1.61 × 10^-4^ | rs708382 | 2,632 |
| cg06491244 | 17 | 44,851,046 | exonic | *WNT3* | BIOS | 0.04 | 28 | 0.92 (0.89 ± 0.95) | 2.87 × 10^-6^ | 9.21 × 10^-4^ | rs708382 | -6,141 |
| cg07661564 | 17 | 44,919,658 | intergenic | *WNT3, WNT9B* | BIOS | 0.01 | 36 | 1.11 (1.06 ± 1.16) | 3.06 × 10^-6^ | 9.67 × 10^-4^ | rs708382 | 34,556 |
| cg23955979 | 17 | 45,126,661 | UTR3 | *ARL17A, ARL17B* | FHS | 0.25 | 125 | 0.75 (0.67 ± 0.83) | **4.41 × 10^-8^** | 2.98 × 10^-5^ | rs28394864 | 4,484 |
| cg04049981 | 17 | 48,894,894 | intergenic | *MIR8059, WFIKKN2* | BIOS | 0.02 | 17 | 1.10 (1.05 ± 1.16) | 1.19 × 10^-4^ | 1.71 × 10^-2^ | rs28394864 | 49,146 |
| cg17717010 | 17 | 48,997,124 | intergenic | *TOB1-AS1, SPAG9* | BIOS | 0.02 | 9 | 1.14 (1.08 ± 1.20) | 4.72 × 10^-6^ | 1.37 × 10^-3^ | rs28394864 | -32,580 |
| cg15590387 | 17 | 49,009,145 | intergenic | *TOB1-AS1, SPAG9* | FHS | 0.27 | 16 | 0.95 (0.93 ± 0.97) | 1.55 × 10^-5^ | 3.68 × 10^-3^ | rs28394864 | 19,986 |
| cg15590387 | 17 | 49,009,145 | intergenic | *TOB1-AS1, SPAG9* | BIOS | 0.36 | 50 | 0.98 (0.97 ± 0.99) | 3.26 × 10^-4^ | 3.48 × 10^-2^ | rs28394864 | -2,397 |
| cg05526498 | 17 | 49,012,893 | intergenic | *TOB1-AS1, SPAG9* | BIOS | 0.02 | 12 | 1.14 (1.08 ± 1.20) | 2.99 × 10^-6^ | 9.53 × 10^-4^ | rs28394864 | 62,371 |
| cg05735639 | 17 | 49,021,875 | intergenic | *TOB1-AS1, SPAG9* | FHS | 0.02 | 10 | 0.78 (0.70 ± 0.87) | 1.37 × 10^-5^ | 3.34 × 10^-3^ | rs28394864 | 20,014 |
| cg05735639 | 17 | 49,021,875 | intergenic | *TOB1-AS1, SPAG9* | BIOS | 0.03 | 5 | 0.89 (0.85 ± 0.94) | 1.66 × 10^-5^ | 3.88 × 10^-3^ | rs28394864 | 35,827 |
| cg17852791 | 17 | 49,022,312 | intergenic | *TOB1-AS1, SPAG9* | FHS | 0.11 | 17 | 1.08 (1.04 ± 1.13) | 6.67 × 10^-5^ | 1.11 × 10^-2^ | rs28394864 | 23,662 |
| cg06329491 | 17 | 49,031,751 | intergenic | *TOB1-AS1, SPAG9* | FHS | 0.13 | 12 | 1.08 (1.04 ± 1.12) | 1.08 × 10^-4^ | 1.59 × 10^-2^ | rs28394864 | -3,246 |
| cg01940943 | 17 | 57,287,791 | exonic | *SMG8* | FHS | 0.02 | 16 | 1.17 (1.07 ± 1.28) | 4.38 × 10^-4^ | 4.27 × 10^-2^ | rs2632516 | 2,324 |
| cg21380024 | 17 | 61,042,803 | upstream | *TANC2* | BIOS | 0.08 | 17 | 1.04 (1.02 ± 1.06) | 4.14 × 10^-4^ | 4.11 × 10^-2^ | rs6504163 | -1,780 |
| cg22141237 | 18 | 29,671,618 | upstream | *RNF138* | FHS | 0.11 | 13 | 1.11 (1.05 ± 1.18) | 2.52 × 10^-4^ | 2.92 × 10^-2^ | rs76726049 | 2,933 |
| cg16292851 | 18 | 29,710,780 | UTR3 | *RNF138* | BIOS | 0.01 | 4 | 0.83 (0.76 ± 0.90) | 5.73 × 10^-6^ | 1.63 × 10^-3^ | rs76726049 | -32,722 |
| cg24811864 | 18 | 55,019,711 | upstream | *ST8SIA3* | BIOS | 0.03 | 9 | 0.93 (0.89 ± 0.97) | 2.94 × 10^-4^ | 3.23 × 10^-2^ | rs76726049 | 6,501 |
| cg04017533 | 18 | 55,020,099 | exonic | *ST8SIA3* | FHS | 0.02 | 24 | 0.86 (0.79 ± 0.93) | 2.51 × 10^-4^ | 2.90 × 10^-2^ | rs76726049 | 2,934 |
| cg04017533 | 18 | 55,020,099 | exonic | *ST8SIA3* | BIOS | 0.03 | 2 | 0.93 (0.90 ± 0.97) | 3.06 × 10^-4^ | 3.33 × 10^-2^ | rs76726049 | 12,649 |
| cg17804850 | 18 | 60,052,386 | UTR3 | *TNFRSF11A* | FHS | 0.04 | 54 | 1.12 (1.05 ± 1.19) | 4.55 × 10^-4^ | 4.37 × 10^-2^ | rs76726049 | 1,170 |
| cg06062848 | 18 | 60,194,352 | intronic | *ZCCHC2* | BIOS | 0.07 | 17 | 0.95 (0.92 ± 0.98) | 3.82 × 10^-4^ | 3.86 × 10^-2^ | rs76726049 | -1,421 |
| cg18493147 | 19 | 474,445 | intronic | *ODF3L2* | BIOS | 0.02 | 13 | 0.89 (0.83 ± 0.95) | 3.34 × 10^-4^ | 3.55 × 10^-2^ | rs12151021 | -10,026 |
| cg24060040 | 19 | 5,802,267 | exonic | *FLOT1* | FHS | 0.19 | 64 | 0.93 (0.91 ± 0.96) | 6.35 × 10^-6^ | 1.76 × 10^-3^ | rs149080927 | -32,761 |
| cg24060040 | 19 | 5,802,267 | intronic | *RNF43* | BIOS | 0.29 | 65 | 0.98 (0.96 ± 0.99) | 2.60 × 10^-5^ | 5.50 × 10^-3^ | rs149080927 | -6,999 |
| cg03635300 | 19 | 5,802,444 | intergenic | *DUS3L, NRTN* | FHS | 0.08 | 16 | 0.87 (0.82 ± 0.92) | 4.54 × 10^-7^ | 1.99 × 10^-4^ | rs149080927 | -16,798 |
| cg03635300 | 19 | 5,802,444 | intergenic | *DUS3L, NRTN* | BIOS | 0.09 | 46 | 0.96 (0.94 ± 0.98) | 3.20 × 10^-5^ | 6.38 × 10^-3^ | rs149080927 | 30,075 |
| cg08524344 | 19 | 5,803,863 | intergenic | *DUS3L, NRTN* | FHS | 0.04 | 23 | 0.85 (0.79 ± 0.92) | 8.79 × 10^-5^ | 1.37 × 10^-2^ | rs149080927 | 6,276 |
| cg14731657 | 19 | 5,805,771 | intergenic | *DUS3L, NRTN* | BIOS | 0.01 | 11 | 1.14 (1.06 ± 1.22) | 2.28 × 10^-4^ | 2.71 × 10^-2^ | rs149080927 | 28,900 |
| cg05881698 | 19 | 5,818,763 | intergenic | *DUS3L, NRTN* | BIOS | 0.03 | 21 | 0.93 (0.90 ± 0.97) | 5.04 × 10^-4^ | 4.68 × 10^-2^ | rs149080927 | 1,104 |
| cg24035928 | 19 | 5,822,514 | intergenic | *DUS3L, NRTN* | FHS | 0.11 | 40 | 0.92 (0.88 ± 0.96) | 2.70 × 10^-4^ | 3.04 × 10^-2^ | rs149080927 | 12,458 |
| cg07997737 | 19 | 5,822,630 | intergenic | *DUS3L, NRTN* | FHS | 0.10 | 74 | 0.93 (0.89 ± 0.96) | 5.33 × 10^-5^ | 9.40 × 10^-3^ | rs149080927 | 2,849 |
| cg25741533 | 19 | 5,829,199 | upstream | *CR1L* | FHS | 0.24 | 11 | 0.94 (0.92 ± 0.97) | 7.52 × 10^-5^ | 1.22 × 10^-2^ | rs149080927 | 6,297 |
| cg25741533 | 19 | 5,829,199 | downstream | *NRTN* | BIOS | 0.24 | 23 | 0.98 (0.96 ± 0.99) | 2.36 × 10^-4^ | 2.77 × 10^-2^ | rs149080927 | 1,208 |
| cg03008525 | 19 | 5,829,415 | intronic | *MAFK* | FHS | 0.14 | 8 | 0.93 (0.89 ± 0.96) | 8.91 × 10^-5^ | 1.38 × 10^-2^ | rs149080927 | 6,272 |
| cg13572146 | 19 | 5,832,296 | exonic | *FUT6* | FHS | 0.02 | 22 | 0.81 (0.73 ± 0.90) | 9.29 × 10^-5^ | 1.42 × 10^-2^ | rs149080927 | 6,268 |
| cg00579402 | 19 | 5,838,999 | UTR5 | *FUT6* | FHS | 0.10 | 75 | 1.08 (1.04 ± 1.12) | 2.29 × 10^-4^ | 2.72 × 10^-2^ | rs149080927 | -1,463 |
| cg00579402 | 19 | 5,838,999 | UTR5 | *FUT6* | BIOS | 0.14 | 66 | 1.03 (1.01 ± 1.05) | 4.14 × 10^-4^ | 4.11 × 10^-2^ | rs149080927 | -1,806 |
| cg10139370 | 19 | 5,867,332 | exonic | *FUT5* | BIOS | 0.01 | 14 | 1.12 (1.06 ± 1.19) | 1.55 × 10^-4^ | 2.06 × 10^-2^ | rs149080927 | -4,703 |
| cg06581718 | 19 | 7,541,760 | downstream | *PEX11G* | BIOS | 0.04 | 41 | 0.94 (0.91 ± 0.97) | 1.20 × 10^-4^ | 1.72 × 10^-2^ | rs149080927 | 49,146 |
| cg27268574 | 19 | 17,858,101 | upstream | *FCHO1* | BIOS | 0.03 | 20 | 0.93 (0.89 ± 0.97) | 3.55 × 10^-4^ | 3.68 × 10^-2^ | rs149080927 | 4,259 |
| cg22904711 | 19 | 44,278,628 | exonic | *KCNN4* | BIOS | 0.11 | 52 | 1.04 (1.02 ± 1.07) | 3.38 × 10^-4^ | 3.57 × 10^-2^ | rs429358 | 11,608 |
| cg15977816 | 19 | 44,285,297 | upstream | *KCNN4* | FHS | 0.07 | 26 | 1.10 (1.04 ± 1.16) | 4.84 × 10^-4^ | 4.56 × 10^-2^ | rs429358 | -3,975 |
| cg14066757 | 19 | 44,285,568 | upstream | *KCNN4* | BIOS | 0.29 | 53 | 1.03 (1.01 ± 1.05) | 1.33 × 10^-4^ | 1.85 × 10^-2^ | rs429358 | 18,488 |
| cg14066757 | 19 | 44,285,568 | upstream | *KCNN4* | FHS | 0.22 | 30 | 1.06 (1.03 ± 1.09) | 2.46 × 10^-4^ | 2.85 × 10^-2^ | rs429358 | 2,934 |
| cg24900654 | 19 | 44,285,594 | upstream | *KCNN4* | FHS | 0.06 | 18 | 1.17 (1.08 ± 1.26) | 3.68 × 10^-5^ | 7.15 × 10^-3^ | rs429358 | 10,906 |
| cg18506018 | 19 | 44,285,974 | upstream | *KCNN4* | BIOS | 0.03 | 12 | 1.13 (1.07 ± 1.20) | 1.21 × 10^-5^ | 2.99 × 10^-3^ | rs429358 | 20,014 |
| cg04833845 | 19 | 44,286,076 | upstream | *KCNN4* | FHS | 0.06 | 18 | 1.16 (1.09 ± 1.24) | 8.70 × 10^-6^ | 2.27 × 10^-3^ | rs429358 | 9,080 |
| cg04833845 | 19 | 44,286,076 | upstream | *KCNN4* | BIOS | 0.06 | 20 | 1.07 (1.03 ± 1.11) | 1.11 × 10^-4^ | 1.62 × 10^-2^ | rs429358 | 4,079 |
| cg06420512 | 19 | 44,299,940 | UTR3 | *SCNN1B* | BIOS | 0.04 | 8 | 1.10 (1.05 ± 1.15) | 3.07 × 10^-5^ | 6.18 × 10^-3^ | rs429358 | 30,076 |
| cg06420512 | 19 | 44,299,940 | upstream | *FGF17* | FHS | 0.03 | 14 | 1.19 (1.09 ± 1.30) | 6.58 × 10^-5^ | 1.10 × 10^-2^ | rs429358 | 23,684 |
| cg04590610 | 19 | 44,303,858 | exonic | *LYPD5* | BIOS | 0.08 | 14 | 1.07 (1.03 ± 1.11) | 1.03 × 10^-4^ | 1.54 × 10^-2^ | rs429358 | 6,261 |
| cg04590610 | 19 | 44,303,858 | exonic | *LYPD5* | FHS | 0.03 | 18 | 1.17 (1.07 ± 1.28) | 3.72 × 10^-4^ | 3.79 × 10^-2^ | rs429358 | -1,219 |
| cg00515669 | 19 | 44,307,449 | intronic | *LYPD5* | BIOS | 0.11 | 18 | 1.05 (1.02 ± 1.07) | 4.26 × 10^-4^ | 4.18 × 10^-2^ | rs429358 | -1,879 |
| cg18106548 | 19 | 44,455,140 | upstream | *ZNF221* | FHS | 0.14 | 24 | 1.10 (1.06 ± 1.14) | 3.05 × 10^-7^ | 1.44 × 10^-4^ | rs429358 | 6,717 |
| cg18106548 | 19 | 44,455,140 | upstream | *ZNF221* | BIOS | 0.14 | 19 | 1.03 (1.01 ± 1.05) | 1.42 × 10^-4^ | 1.96 × 10^-2^ | rs429358 | -32,687 |
| cg19591950 | 19 | 44,455,176 | upstream | *ZNF221* | FHS | 0.03 | 21 | 1.24 (1.14 ± 1.34) | 4.12 × 10^-7^ | 1.84 × 10^-4^ | rs429358 | 572 |
| cg19792908 | 19 | 44,455,181 | upstream | *ZNF221* | FHS | 0.09 | 13 | 1.13 (1.08 ± 1.19) | 5.02 × 10^-7^ | 2.18 × 10^-4^ | rs429358 | -16,814 |
| cg19792908 | 19 | 44,455,181 | upstream | *ZNF221* | BIOS | 0.04 | 12 | 1.06 (1.03 ± 1.09) | 3.50 × 10^-4^ | 3.66 × 10^-2^ | rs429358 | 4,602 |
| cg04577268 | 19 | 44,455,327 | upstream | *ZNF221* | BIOS | 0.02 | 11 | 0.93 (0.89 ± 0.97) | 3.37 × 10^-4^ | 3.56 × 10^-2^ | rs429358 | 11,615 |
| cg15602972 | 19 | 47,151,481 | UTR3 | *DACT3* | BIOS | 0.02 | 10 | 0.89 (0.83 ± 0.95) | 4.43 × 10^-4^ | 4.28 × 10^-2^ | rs76320948 | -1,571 |
| cg01406381 | 19 | 47,288,263 | intergenic | *LINC00533, LINC01623* | BIOS | 0.03 | 25 | 1.11 (1.06 ± 1.17) | 5.76 × 10^-5^ | 9.96 × 10^-3^ | rs76320948 | 29,736 |
| cg00993388 | 19 | 47,505,824 | UTR3 | *ARHGAP35* | BIOS | 0.40 | 5 | 0.98 (0.96 ± 0.99) | 1.64 × 10^-4^ | 2.14 × 10^-2^ | rs76320948 | -9,812 |
| cg00993388 | 19 | 47,505,824 | UTR3 | *ARHGAP35* | FHS | 0.28 | 12 | 0.95 (0.92 ± 0.98) | 1.80 × 10^-4^ | 2.30 × 10^-2^ | rs76320948 | -4,085 |
| cg24657473 | 20 | 986,485 | intergenic | *RSPO4, PSMF1* | BIOS | 0.31 | 26 | 1.02 (1.01 ± 1.04) | 2.56 × 10^-4^ | 2.93 × 10^-2^ | rs1358782 | -726 |
| cg24657473 | 20 | 986,485 | intergenic | *RSPO4, PSMF1* | FHS | 0.09 | 15 | 1.09 (1.04 ± 1.14) | 4.76 × 10^-4^ | 4.50 × 10^-2^ | rs1358782 | -3,975 |
| cg24616366 | 20 | 1,781,084 | intergenic | *LOC100289473, SIRPA* | BIOS | 0.01 | 11 | 0.86 (0.79 ± 0.93) | 1.47 × 10^-4^ | 2.00 × 10^-2^ | rs1358782 | 10,052 |
| cg22163406 | 20 | 35,917,429 | upstream | *MANBAL* | BIOS | 0.11 | 24 | 1.02 (1.01 ± 1.04) | 4.40 × 10^-5^ | 8.20 × 10^-3^ | rs6069737 | 10,838 |
| cg04541293 | 20 | 41,819,125 | upstream | *PTPRT* | FHS | 0.02 | 43 | 1.16 (1.07 ± 1.26) | 3.94 × 10^-4^ | 3.94 × 10^-2^ | rs6069737 | -1,534 |
| cg05072995 | 20 | 44,030,646 | ncRNA_intronic | *SYS1-DBNDD2* | BIOS | 0.01 | 7 | 0.88 (0.82 ± 0.95) | 5.46 × 10^-4^ | 4.94 × 10^-2^ | rs6069737 | - |
| cg10619398 | 20 | 47,937,607 | intergenic | *ZFAS1, KCNB1* | BIOS | 0.14 | 21 | 0.96 (0.95 ± 0.98) | 2.58 × 10^-4^ | 2.95 × 10^-2^ | rs6069737 | 3,754 |
| cg24112000 | 20 | 60,950,667 | ncRNA_intronic | *MIR1-1HG-AS1* | FHS | 0.55 | 36 | 0.97 (0.95 ± 0.99) | 2.54 × 10^-4^ | 2.93 × 10^-2^ | rs6742 | 21,842 |
| cg24112000 | 20 | 60,950,667 | ncRNA_intronic | *MIR1-1HG-AS1* | BIOS | 0.70 | 52 | 0.98 (0.98 ± 0.99) | 3.78 × 10^-4^ | 3.84 × 10^-2^ | rs6742 | -1,258 |
| cg20000468 | 21 | 34,601,686 | upstream | *IFNAR2* | BIOS | 0.01 | 6 | 0.86 (0.80 ± 0.93) | 1.20 × 10^-4^ | 1.72 × 10^-2^ | rs2154482 | 49,146 |
| cg17977197 | 21 | 34,601,762 | upstream | *IFNAR2* | BIOS | 0.06 | 35 | 0.95 (0.93 ± 0.98) | 3.52 × 10^-4^ | 3.66 × 10^-2^ | rs2154482 | 4,587 |
| cg12829325 | 21 | 34,638,188 | ncRNA_exonic | *IL10RB-DT* | FHS | 0.03 | 36 | 0.86 (0.80 ± 0.92) | 1.09 × 10^-5^ | 2.73 × 10^-3^ | rs2154482 | 20,053 |
| cg00702593 | 21 | 42,219,853 | upstream | *DSCAM* | BIOS | 0.02 | 38 | 0.91 (0.87 ± 0.95) | 6.35 × 10^-5^ | 1.07 × 10^-2^ | rs2154482 | 24,493 |
| cg15377887 | 21 | 42,440,661 | intergenic | *DSCAM, LINC00323* | FHS | 0.49 | 44 | 1.03 (1.01 ± 1.05) | 2.69 × 10^-4^ | 3.03 × 10^-2^ | rs2154482 | 18,990 |
| cg15504662 | 22 | 31,644,160 | intronic | *LIMK2* | BIOS | 0.06 | 25 | 1.05 (1.02 ± 1.09) | 3.97 × 10^-4^ | 3.96 × 10^-2^ | - | -1,585 |
| cg20742981 | 22 | 31,644,190 | intronic | *LIMK2* | BIOS | 0.04 | 24 | 1.07 (1.03 ± 1.11) | 3.68 × 10^-4^ | 3.76 × 10^-2^ | - | -1,069 |
| cg07713946 | 22 | 31,675,144 | UTR3 | *LIMK2* | BIOS | 0.19 | 9 | 0.97 (0.95 ± 0.98) | 1.22 × 10^-4^ | 1.73 × 10^-2^ | - | 49,146 |
| cg07713946 | 22 | 31,675,144 | UTR3 | *LIMK2* | FHS | 0.16 | 24 | 0.95 (0.92 ± 0.98) | 3.64 × 10^-4^ | 3.74 × 10^-2^ | - | 1,590 |
| cg01550271 | 22 | 31,798,207 | intronic | *DRG1* | BIOS | 0.01 | 7 | 1.14 (1.06 ± 1.23) | 2.19 × 10^-4^ | 2.63 × 10^-2^ | - | -9,660 |
| cg01133262 | 22 | 38,863,905 | upstream | *KDELR3* | BIOS | 0.04 | 23 | 1.06 (1.03 ± 1.09) | 3.73 × 10^-4^ | 3.79 × 10^-2^ | - | -1,220 |
| cg13604020 | 22 | 38,902,925 | upstream | *DDX17* | FHS | 0.01 | 65 | 1.14 (1.07 ± 1.21) | 6.15 × 10^-5^ | 1.05 × 10^-2^ | - | 6,586 |
| cg09282085 | 22 | 39,713,086 | intronic | *RPL3* | BIOS | 0.15 | 25 | 0.96 (0.94 ± 0.98) | 2.14 × 10^-4^ | 2.60 × 10^-2^ | - | -9,592 |
| cg23558213 | 22 | 41,215,939 | upstream | *SLC25A17* | BIOS | 0.12 | 13 | 0.96 (0.94 ± 0.98) | 3.25 × 10^-4^ | 3.47 × 10^-2^ | - | 4,967 |
| cg23558213 | 22 | 41,215,939 | upstream | *SLC25A17* | FHS | 0.07 | 15 | 0.91 (0.86 ± 0.96) | 3.59 × 10^-4^ | 3.71 × 10^-2^ | - | 1,716 |
| cg23684502 | 22 | 41,217,753 | intergenic | *SLC25A17, ST13* | FHS | 0.04 | 15 | 1.14 (1.07 ± 1.22) | 1.44 × 10^-4^ | 1.97 × 10^-2^ | - | -20,320 |

^1^BIOS: Biobank-based Integrative Omics Studies; Chr: chromosome; CI: confidence interval; CpG: CpG sites; FHS: Framingham Heart Study; kb: kilobase; ncRNA: noncoding RNA; OR: odds ratio per SD increase in genetically predicated DNA methylation level (continuous variable); SNP: single-nucleotide polymorphisms; UTR: untranslated region; ^2^R^2^: prediction performance; ^3^S-PrediXcan was used to estimate association ORs, 95% CIs and *P* value. All statistical tests were two-sided; ^4^associations with FDR-corrected *P* value < 0.05 considered significant; ^5^the closest risk SNPs identified in previous GWAS studies[1-4]; ^6^the distance from CpG site to risk SNP and “-” indicate the CpG site located downstream of the risk SNP.

**Table S2. Six hundred and fifty-nine DNA methylation markers associated with AD risk identified in genomic regions within 500 kb of susceptibility variants reported for AD risk.**

| **CpG^1^** | **Chr** | **Position** | **Classification** | **Closest gene** | **Model** | **R^2 2^** | **Number**  **of SNPs**  **in model** | **OR (95% CI)^3^** | ***P* value** | ***P* value**  **after FDR^4^** | **closest AD**  **risk SNP^5^** | **Distance**  **To risk**  **SNP (kb)^6^** |
| --- | --- | --- | --- | --- | --- | --- | --- | --- | --- | --- | --- | --- |
| cg17938867 | 1 | 109,418,898 | upstream | *GPSM2* | FHS | 0.01 | 7 | 0.73 (0.62 ± 0.86) | 2.17 × 10^-4^ | 2.62 × 10^-2^ | rs141749679 | 469.53 |
| cg27582585 | 1 | 161,069,839 | intronic | *KLHDC9* | FHS | 0.01 | 5 | 0.62 (0.49 ± 0.78) | 7.72 × 10^-5^ | 1.25 × 10^-2^ | rs4575098 | 85.55 |
| cg15516558 | 1 | 161,091,980 | exonic | *DEDD* | FHS | 0.24 | 10 | 1.07 (1.04 ± 1.10) | 3.28 × 10^-7^ | 1.53 × 10^-4^ | rs4575098 | 63.41 |
| cg15516558 | 1 | 161,091,980 | exonic | *DEDD* | BIOS | 0.26 | 26 | 1.03 (1.02 ± 1.05) | 5.79 × 10^-6^ | 1.64 × 10^-3^ | rs4575098 | 63.41 |
| cg07178064 | 1 | 161,111,994 | intergenic | *LOC112543491, UFC1* | FHS | 0.03 | 9 | 0.75 (0.67 ± 0.84) | 4.11 × 10^-7^ | 1.84 × 10^-4^ | rs4575098 | 43.40 |
| cg07178064 | 1 | 161,111,994 | intergenic | *LOC112543491, UFC1* | BIOS | 0.03 | 7 | 0.91 (0.87 ± 0.95) | 7.05 × 10^-5^ | 1.17 × 10^-2^ | rs4575098 | 43.40 |
| cg16673712 | 1 | 161,122,960 | upstream | *UFC1* | BIOS | 0.09 | 8 | 0.95 (0.92 ± 0.97) | 2.88 × 10^-5^ | 5.90 × 10^-3^ | rs4575098 | 32.43 |
| cg09070378 | 1 | 161,183,762 | UTR3 | *NDUFS2* | FHS | 0.17 | 34 | 1.10 (1.07 ± 1.13) | **7.41 × 10^-10^** | 9.02 × 10^-7^ | rs4575098 | -28.37 |
| cg09070378 | 1 | 161,183,762 | exonic | *YPEL3* | BIOS | 0.18 | 12 | 1.05 (1.03 ± 1.07) | **2.51 × 10^-9^** | 2.48 × 10^-6^ | rs4575098 | -28.37 |
| cg07356342 | 1 | 161,183,820 | UTR3 | *NDUFS2* | BIOS | 0.04 | 22 | 1.10 (1.07 ± 1.14) | **2.92 × 10^-11^** | 5.17 × 10^-8^ | rs4575098 | -28.43 |
| cg07356342 | 1 | 161,183,820 | UTR3 | *NDUFS2* | FHS | 0.05 | 16 | 1.19 (1.12 ± 1.26) | **9.73 × 10^-9^** | 7.89 × 10^-6^ | rs4575098 | -28.43 |
| cg05659526 | 1 | 161,184,528 | upstream, downstream | *FCER1G, NDUFS2* | BIOS | 0.02 | 19 | 1.14 (1.09 ± 1.20) | **5.72 × 10^-8^** | 3.64 × 10^-5^ | rs4575098 | -29.14 |
| cg09106231 | 1 | 207,455,715 | intronic | *DGKQ* | BIOS | 0.06 | 19 | 0.94 (0.91 ± 0.97) | 2.43 × 10^-5^ | 5.21 × 10^-3^ | rs6656401 | 236.33 |
| cg09106231 | 1 | 207,455,715 | intergenic | *C4BPA, CD55* | FHS | 0.06 | 41 | 0.90 (0.85 ± 0.96) | 3.81 × 10^-4^ | 3.85 × 10^-2^ | rs6656401 | 236.33 |
| cg02152968 | 1 | 207,494,213 | upstream | *CD55* | BIOS | 0.17 | 11 | 1.05 (1.03 ± 1.07) | **2.85 × 10^-8^** | 2.04 × 10^-5^ | rs6656401 | 197.84 |
| cg02152968 | 1 | 207,494,213 | upstream | *CD55* | FHS | 0.24 | 32 | 1.08 (1.05 ± 1.11) | **1.70 × 10^-7^** | 8.81 × 10^-5^ | rs6656401 | 197.84 |
| cg00797651 | 1 | 207,494,336 | intronic | *YWHAQ* | FHS | 0.57 | 59 | 1.04 (1.02 ± 1.05) | 1.40 × 10^-5^ | 3.39 × 10^-3^ | rs6656401 | 197.71 |
| cg00797651 | 1 | 207,494,336 | upstream | *CD55* | BIOS | 0.64 | 36 | 1.02 (1.01 ± 1.03) | 1.61 × 10^-5^ | 3.80 × 10^-3^ | rs6656401 | 197.71 |
| cg05825950 | 1 | 207,669,576 | UTR5 | *CR1* | FHS | 0.05 | 22 | 1.11 (1.04 ± 1.17) | 5.54 × 10^-4^ | 4.97 × 10^-2^ | rs6656401 | 22.47 |
| cg21110645 | 1 | 207,815,933 | downstream | *CR1* | BIOS | 0.57 | 97 | 1.02 (1.01 ± 1.03) | 8.66 × 10^-6^ | 2.26 × 10^-3^ | rs679515 | -65.37 |
| cg21110645 | 1 | 207,815,933 | downstream | *CR1* | FHS | 0.42 | 109 | 1.03 (1.02 ± 1.05) | 5.44 × 10^-5^ | 9.53 × 10^-3^ | rs679515 | -65.37 |
| cg26119563 | 1 | 207,817,838 | upstream | *CR1L* | FHS | 0.10 | 41 | 0.92 (0.88 ± 0.96) | 4.67 × 10^-5^ | 8.56 × 10^-3^ | rs679515 | -67.27 |
| cg26119563 | 1 | 207,817,838 | upstream | *CR1L* | BIOS | 0.11 | 13 | 0.95 (0.93 ± 0.98) | 1.86 × 10^-4^ | 2.35 × 10^-2^ | rs679515 | -67.27 |
| cg15718572 | 1 | 207,818,003 | upstream | *CR1L* | FHS | 0.05 | 30 | 0.90 (0.85 ± 0.95) | 2.44 × 10^-4^ | 2.84 × 10^-2^ | rs679515 | -67.44 |
| cg13234569 | 1 | 207,818,036 | downstream | *NRTN* | FHS | 0.02 | 24 | 0.82 (0.74 ± 0.90) | 7.52 × 10^-5^ | 1.22 × 10^-2^ | rs679515 | -67.47 |
| cg24557058 | 1 | 207,818,493 | UTR5 | *CR1L* | FHS | 0.02 | 39 | 0.80 (0.73 ± 0.88) | 7.92 × 10^-6^ | 2.11 × 10^-3^ | rs679515 | -67.93 |
| cg21106486 | 1 | 207,843,084 | intronic | *CR1L* | FHS | 0.07 | 19 | 1.12 (1.06 ± 1.18) | 4.25 × 10^-5^ | 8.02 × 10^-3^ | rs679515 | -92.52 |
| cg22500738 | 1 | 207,928,706 | intronic | *CD46* | FHS | 0.12 | 50 | 1.08 (1.04 ± 1.12) | 4.01 × 10^-5^ | 7.66 × 10^-3^ | rs679515 | -178.14 |
| cg15770106 | 1 | 208,133,116 | intergenic | *CD34, PLXNA2* | FHS | 0.11 | 21 | 0.93 (0.89 ± 0.96) | 1.93 × 10^-4^ | 2.41 × 10^-2^ | rs679515 | -382.55 |
| cg13884879 | 2 | 9,540,759 | intronic | *ASAP2* | FHS | 0.05 | 26 | 0.86 (0.81 ± 0.92) | 5.40 × 10^-6^ | 1.55 × 10^-3^ | rs72777026 | 158.25 |
| cg26306976 | 2 | 9,564,901 | UTR5 | *CPSF3* | BIOS | 0.54 | 35 | 0.98 (0.97 ± 0.99) | 5.69 × 10^-7^ | 2.43 × 10^-4^ | rs72777026 | 134.11 |
| cg26306976 | 2 | 9,564,901 | UTR5 | *CPSF3* | FHS | 0.57 | 22 | 0.96 (0.94 ± 0.98) | 7.78 × 10^-7^ | 3.17 × 10^-4^ | rs72777026 | 134.11 |
| cg07974891 | 2 | 9,565,042 | UTR5 | *CPSF3* | BIOS | 0.03 | 15 | 0.92 (0.88 ± 0.96) | 6.04 × 10^-5^ | 1.03 × 10^-2^ | rs72777026 | 133.97 |
| cg04210100 | 2 | 9,614,471 | upstream | *IAH1* | BIOS | 0.02 | 20 | 1.09 (1.04 ± 1.14) | 1.32 × 10^-4^ | 1.84 × 10^-2^ | rs72777026 | 84.54 |
| cg04254681 | 2 | 9,679,744 | intronic | *ADAM17* | BIOS | 0.10 | 6 | 1.06 (1.03 ± 1.08) | 4.76 × 10^-5^ | 8.69 × 10^-3^ | rs72777026 | 19.27 |
| cg01231165 | 2 | 9,695,142 | intronic | *ADAM17* | FHS | 0.02 | 4 | 0.67 (0.56 ± 0.79) | 4.20 × 10^-6^ | 1.26 × 10^-3^ | rs72777026 | 3.87 |
| cg20291846 | 2 | 9,696,659 | upstream | *ADAM17* | BIOS | 0.02 | 9 | 0.92 (0.88 ± 0.96) | 2.79 × 10^-4^ | 3.10 × 10^-2^ | rs72777026 | 2.35 |
| cg18861713 | 2 | 9,766,266 | upstream | *CD55* | BIOS | 0.01 | 6 | 1.19 (1.10 ± 1.28) | 1.40 × 10^-5^ | 3.39 × 10^-3^ | rs72777026 | -67.26 |
| cg01598371 | 2 | 37,423,428 | upstream | *CEBPZOS, SULT6B1* | BIOS | 0.03 | 6 | 0.93 (0.90 ± 0.97) | 1.07 × 10^-4^ | 1.58 × 10^-2^ | rs17020490 | 108.51 |
| cg14207553 | 2 | 106,071,427 | intronic | *SH3TC2* | BIOS | 0.01 | 18 | 1.10 (1.05 ± 1.15) | 5.40 × 10^-5^ | 9.47 × 10^-3^ | rs115186657 | 164.00 |
| cg10152097 | 2 | 106,200,350 | intergenic | *FHL2, LOC285000* | FHS | 0.01 | 29 | 1.26 (1.12 ± 1.41) | 7.70 × 10^-5^ | 1.25 × 10^-2^ | rs115186657 | 35.08 |
| cg25961567 | 2 | 106,363,603 | intronic | *NCK2* | BIOS | 0.06 | 49 | 1.05 (1.02 ± 1.07) | 4.38 × 10^-4^ | 4.27 × 10^-2^ | rs143080277 | 2.45 |
| cg13704531 | 2 | 106,406,742 | intronic | *NCK2* | BIOS | 0.02 | 7 | 0.88 (0.83 ± 0.94) | 8.32 × 10^-5^ | 1.32 × 10^-2^ | rs143080277 | -40.69 |
| cg17501982 | 2 | 106,417,701 | intronic | *NCK2* | BIOS | 0.07 | 69 | 1.04 (1.02 ± 1.06) | 4.20 × 10^-4^ | 4.14 × 10^-2^ | rs143080277 | -51.65 |
| cg26377276 | 2 | 106,485,483 | intronic | *NCK2* | FHS | 0.01 | 32 | 1.19 (1.08 ± 1.31) | 3.40 × 10^-4^ | 3.59 × 10^-2^ | rs143080277 | -119.43 |
| cg26377276 | 2 | 106,485,483 | intronic | *NCK2* | BIOS | 0.01 | 12 | 1.12 (1.05 ± 1.19) | 4.09 × 10^-4^ | 4.06 × 10^-2^ | rs143080277 | -119.43 |
| cg03181966 | 2 | 106,493,892 | intronic | *PRSS36* | BIOS | 0.03 | 31 | 1.09 (1.05 ± 1.13) | 2.57 × 10^-5^ | 5.45 × 10^-3^ | rs143080277 | -127.84 |
| cg05208056 | 2 | 106,502,915 | UTR5 | *TBX6* | BIOS | 0.02 | 25 | 1.13 (1.07 ± 1.19) | 1.92 × 10^-5^ | 4.34 × 10^-3^ | rs143080277 | -136.86 |
| cg00409696 | 2 | 106,646,656 | exonic | *APOBEC2* | FHS | 0.11 | 53 | 1.09 (1.04 ± 1.13) | 6.30 × 10^-5^ | 1.07 × 10^-2^ | rs143080277 | -280.60 |
| cg08563189 | 2 | 127,780,654 | intergenic | *TEX51, BIN1* | FHS | 0.03 | 9 | 1.40 (1.27 ± 1.54) | **1.72 × 10^-11^** | 3.40 × 10^-8^ | rs4663105 | 110.77 |
| cg08563189 | 2 | 127,780,654 | intergenic | *TEX51, BIN1* | BIOS | 0.05 | 10 | 1.13 (1.09 ± 1.17) | **1.87 × 10^-11^** | 3.62 × 10^-8^ | rs4663105 | 110.77 |
| cg19153828 | 2 | 127,782,651 | intergenic | *TEX51, BIN1* | FHS | 0.27 | 35 | 1.06 (1.03 ± 1.08) | 3.85 × 10^-6^ | 1.17 × 10^-3^ | rs4663105 | 108.78 |
| cg19153828 | 2 | 127,782,651 | intergenic | *TEX51, BIN1* | BIOS | 0.43 | 27 | 1.02 (1.01 ± 1.04) | 1.96 × 10^-5^ | 4.40 × 10^-3^ | rs4663105 | 108.78 |
| cg19590598 | 2 | 127,782,813 | intergenic | *TEX51, BIN1* | FHS | 0.34 | 37 | 1.05 (1.03 ± 1.08) | 9.82 × 10^-7^ | 3.86 × 10^-4^ | rs4663105 | 108.61 |
| cg19590598 | 2 | 127,782,813 | intergenic | *TEX51, BIN1* | BIOS | 0.51 | 36 | 1.02 (1.01 ± 1.03) | 3.29 × 10^-5^ | 6.54 × 10^-3^ | rs4663105 | 108.61 |
| cg03766620 | 2 | 127,783,082 | intergenic | *TEX51, BIN1* | FHS | 0.10 | 18 | 1.13 (1.08 ± 1.18) | **1.23 × 10^-7^** | 6.94 × 10^-5^ | rs4663105 | 108.35 |
| cg03766620 | 2 | 127,783,082 | intergenic | *TEX51, BIN1* | BIOS | 0.12 | 18 | 1.05 (1.03 ± 1.08) | **2.50 × 10^-7^** | 1.21 × 10^-4^ | rs4663105 | 108.35 |
| cg21554034 | 2 | 127,785,512 | intergenic | *TEX51, BIN1* | BIOS | 0.04 | 44 | 1.11 (1.08 ± 1.14) | **1.51 × 10^-12^** | 4.33 × 10^-9^ | rs4663105 | 105.92 |
| cg09006514 | 2 | 127,800,616 | intergenic | *TEX51, BIN1* | BIOS | 0.01 | 2 | 0.81 (0.76 ± 0.87) | **5.21 × 10^-9^** | 4.61 × 10^-6^ | rs4663105 | 90.81 |
| cg22883290 | 2 | 127,800,646 | intergenic | *TEX51, BIN1* | BIOS | 0.04 | 11 | 0.90 (0.87 ± 0.93) | **3.02 × 10^-9^** | 2.89 × 10^-6^ | rs4663105 | 90.78 |
| cg22376361 | 2 | 127,815,133 | exonic | *BIN1* | FHS | 0.01 | 9 | 1.60 (1.39 ± 1.84) | **7.36 × 10^-11^** | 1.19 × 10^-7^ | rs4663105 | 76.29 |
| cg24750513 | 2 | 127,819,455 | intronic | *BIN1* | BIOS | 0.02 | 14 | 0.81 (0.77 ± 0.85) | **4.53 × 10^-18^** | 3.42 × 10^-14^ | rs4663105 | 71.97 |
| cg26467631 | 2 | 127,826,083 | intronic | *BIN1* | BIOS | 0.02 | 32 | 0.91 (0.87 ± 0.95) | 9.12 × 10^-5^ | 1.40 × 10^-2^ | rs4663105 | 65.34 |
| cg00436254 | 2 | 127,862,614 | intronic | *BIN1* | FHS | 0.16 | 23 | 1.10 (1.06 ± 1.13) | **1.37 × 10^-7^** | 7.49 × 10^-5^ | rs4663105 | 28.81 |
| cg00436254 | 2 | 127,862,614 | intronic | *BIN1* | BIOS | 0.24 | 40 | 1.03 (1.01 ± 1.04) | 7.66 × 10^-5^ | 1.24 × 10^-2^ | rs4663105 | 28.81 |
| cg14367014 | 2 | 203,735,128 | intronic | *ICA1L* | FHS | 0.01 | 7 | 0.69 (0.60 ± 0.79) | **8.97 × 10^-8^** | 5.33 × 10^-5^ | rs139643391 | 8.31 |
| cg14367014 | 2 | 203,735,128 | intronic | *ICA1L* | BIOS | 0.03 | 5 | 0.92 (0.89 ± 0.95) | **7.77 × 10^-7^** | 3.17 × 10^-4^ | rs139643391 | 8.31 |
| cg14012546 | 2 | 233,981,788 | intronic | *INPP5D* | BIOS | 0.02 | 13 | 0.81 (0.76 ± 0.85) | **8.73 × 10^-15^** | 4.02 × 10^-11^ | rs10933431 | 0.12 |
| cg14012546 | 2 | 233,981,788 | intergenic | *ZSCAN23, GPX6* | FHS | 0.04 | 33 | 0.82 (0.76 ± 0.89) | 2.14 × 10^-6^ | 7.25 × 10^-4^ | rs10933431 | 0.12 |
| cg05299077 | 2 | 233,981,999 | intronic | *INPP5D* | BIOS | 0.21 | 53 | 0.95 (0.94 ± 0.97) | **1.08 × 10^-12^** | 3.27 × 10^-9^ | rs10933431 | -0.09 |
| cg05299077 | 2 | 233,981,999 | intronic | *INPP5D* | FHS | 0.14 | 145 | 0.93 (0.90 ± 0.95) | **5.21 × 10^-8^** | 3.42 × 10^-5^ | rs10933431 | -0.09 |
| cg07026910 | 2 | 234,070,710 | intronic | *INPP5D* | BIOS | 0.02 | 4 | 1.22 (1.15 ± 1.29) | **8.62 × 10^-12^** | 1.99 × 10^-8^ | rs35349669 | -2.23 |
| cg07026910 | 2 | 234,070,710 | intronic | *INPP5D* | FHS | 0.02 | 6 | 1.30 (1.17 ± 1.45) | 1.33 × 10^-6^ | 4.95 × 10^-4^ | rs35349669 | -2.23 |
| cg16448702 | 2 | 234,072,980 | exonic | *INPP5D* | BIOS | 0.02 | 11 | 1.15 (1.09 ± 1.22) | 2.38 × 10^-6^ | 7.85 × 10^-4^ | rs35349669 | -4.50 |
| cg05711037 | 2 | 234,359,783 | intronic | *DGKD* | FHS | 0.30 | 83 | 0.95 (0.93 ± 0.97) | 1.34 × 10^-5^ | 3.28 × 10^-3^ | rs7597763 | -277.21 |
| cg05711037 | 2 | 234,359,783 | intronic | *DGKD* | BIOS | 0.37 | 45 | 0.99 (0.98 ± 0.99) | 1.00 × 10^-4^ | 1.50 × 10^-2^ | rs7597763 | -277.21 |
| cg20428596 | 2 | 234,370,973 | intronic | *DGKD* | BIOS | 0.02 | 8 | 1.10 (1.05 ± 1.16) | 1.47 × 10^-4^ | 2.00 × 10^-2^ | rs7597763 | -288.40 |
| cg11926460 | 2 | 234,378,272 | UTR3 | *DGKD* | FHS | 0.01 | 10 | 0.79 (0.70 ± 0.90) | 3.47 × 10^-4^ | 3.64 × 10^-2^ | rs7597763 | -295.70 |
| cg10530169 | 3 | 57,529,952 | UTR5 | *DNAH12* | BIOS | 0.12 | 24 | 0.97 (0.95 ± 0.98) | 4.38 × 10^-4^ | 4.26 × 10^-2^ | rs184384746 | -303.80 |
| cg10530169 | 3 | 57,529,952 | UTR5 | *DNAH12* | FHS | 0.09 | 30 | 0.93 (0.89 ± 0.97) | 5.29 × 10^-4^ | 4.84 × 10^-2^ | rs184384746 | -303.80 |
| cg21637884 | 3 | 57,530,016 | UTR5 | *DNAH12* | FHS | 0.02 | 9 | 0.83 (0.75 ± 0.92) | 2.70 × 10^-4^ | 3.04 × 10^-2^ | rs184384746 | -303.87 |
| cg24847554 | 3 | 57,530,606 | upstream | *DNAH12* | FHS | 0.10 | 35 | 0.92 (0.89 ± 0.96) | 1.72 × 10^-4^ | 2.23 × 10^-2^ | rs184384746 | -304.46 |
| cg07465899 | 4 | 696,400 | intergenic | *SLC49A3, PCGF3* | FHS | 0.02 | 11 | 0.84 (0.76 ± 0.92) | 1.15 × 10^-4^ | 1.66 × 10^-2^ | rs3822030 | 290.94 |
| cg25670583 | 4 | 715,865 | intronic | *PCGF3* | FHS | 0.06 | 7 | 1.12 (1.06 ± 1.19) | 9.53 × 10^-5^ | 1.45 × 10^-2^ | rs3822030 | 271.48 |
| cg25712567 | 4 | 715,872 | intronic | *PCGF3* | BIOS | 0.37 | 37 | 1.02 (1.01 ± 1.03) | 7.54 × 10^-5^ | 1.23 × 10^-2^ | rs3822030 | 271.47 |
| cg25712567 | 4 | 715,872 | intronic | *PCGF3* | FHS | 0.39 | 13 | 1.04 (1.02 ± 1.06) | 1.12 × 10^-4^ | 1.63 × 10^-2^ | rs3822030 | 271.47 |
| cg16767590 | 4 | 715,950 | intronic | *PCGF3* | FHS | 0.14 | 20 | 1.07 (1.03 ± 1.11) | 2.07 × 10^-4^ | 2.53 × 10^-2^ | rs3822030 | 271.39 |
| cg16767590 | 4 | 715,950 | intronic | *PCGF3* | BIOS | 0.08 | 22 | 1.05 (1.02 ± 1.07) | 2.10 × 10^-4^ | 2.56 × 10^-2^ | rs3822030 | 271.39 |
| cg23077453 | 4 | 719,011 | intronic | *PCGF3* | BIOS | 0.28 | 49 | 1.03 (1.02 ± 1.04) | 4.33 × 10^-7^ | 1.91 × 10^-4^ | rs3822030 | 268.33 |
| cg23077453 | 4 | 719,011 | intronic | *SLC39A13* | FHS | 0.35 | 36 | 1.05 (1.03 ± 1.07) | 1.23 × 10^-5^ | 3.03 × 10^-3^ | rs3822030 | 268.33 |
| cg07697227 | 4 | 719,908 | intronic | *PCGF3* | BIOS | 0.12 | 35 | 1.04 (1.02 ± 1.06) | 6.03 × 10^-6^ | 1.69 × 10^-3^ | rs3822030 | 267.44 |
| cg07697227 | 4 | 719,908 | intronic | *PCGF3* | FHS | 0.12 | 31 | 1.07 (1.03 ± 1.11) | 1.18 × 10^-4^ | 1.69 × 10^-2^ | rs3822030 | 267.44 |
| cg17397159 | 4 | 720,144 | exonic | *BTNL2* | BIOS | 0.41 | 60 | 1.02 (1.01 ± 1.03) | 1.13 × 10^-6^ | 4.32 × 10^-4^ | rs3822030 | 267.20 |
| cg17397159 | 4 | 720,144 | intronic | *PCGF3* | FHS | 0.39 | 9 | 1.04 (1.02 ± 1.06) | 5.37 × 10^-5^ | 9.46 × 10^-3^ | rs3822030 | 267.20 |
| cg17705041 | 4 | 720,218 | intronic | *PCGF3* | BIOS | 0.09 | 34 | 1.05 (1.03 ± 1.08) | 1.32 × 10^-5^ | 3.23 × 10^-3^ | rs3822030 | 267.13 |
| cg17705041 | 4 | 720,218 | intergenic | *GPX5, ZBED9* | FHS | 0.06 | 7 | 1.13 (1.07 ± 1.19) | 2.24 × 10^-5^ | 4.85 × 10^-3^ | rs3822030 | 267.13 |
| cg10864200 | 4 | 720,809 | intronic | *PCGF3* | BIOS | 0.51 | 65 | 1.02 (1.01 ± 1.03) | 6.58 × 10^-6^ | 1.81 × 10^-3^ | rs3822030 | 266.53 |
| cg10864200 | 4 | 720,809 | intronic | *PCGF3* | FHS | 0.61 | 18 | 1.03 (1.02 ± 1.05) | 6.57 × 10^-5^ | 1.10 × 10^-2^ | rs3822030 | 266.53 |
| cg00614959 | 4 | 720,870 | intronic | *PCGF3* | FHS | 0.60 | 23 | 1.03 (1.02 ± 1.05) | 4.78 × 10^-5^ | 8.71 × 10^-3^ | rs3822030 | 266.47 |
| cg00810986 | 4 | 727,748 | intronic | *PCGF3* | FHS | 0.01 | 11 | 1.31 (1.13 ± 1.52) | 3.14 × 10^-4^ | 3.39 × 10^-2^ | rs3822030 | 259.60 |
| cg16754522 | 4 | 734,600 | intronic | *PCGF3* | BIOS | 0.05 | 15 | 0.94 (0.91 ± 0.96) | 2.76 × 10^-5^ | 5.74 × 10^-3^ | rs3822030 | 252.74 |
| cg16754522 | 4 | 734,600 | intronic | *PCGF3* | FHS | 0.01 | 17 | 0.77 (0.67 ± 0.88) | 2.41 × 10^-4^ | 2.82 × 10^-2^ | rs3822030 | 252.74 |
| cg08766099 | 4 | 734,746 | intronic | *PCGF3* | FHS | 0.03 | 12 | 1.17 (1.08 ± 1.26) | 4.14 × 10^-5^ | 7.84 × 10^-3^ | rs3822030 | 252.60 |
| cg18368669 | 4 | 739,962 | intronic | *PCGF3* | BIOS | 0.03 | 15 | 0.90 (0.86 ± 0.93) | **5.69 × 10^-8^** | 3.64 × 10^-5^ | rs3822030 | 247.38 |
| cg18368669 | 4 | 739,962 | intronic | *PCGF3* | FHS | 0.04 | 4 | 0.86 (0.79 ± 0.92) | 4.26 × 10^-5^ | 8.03 × 10^-3^ | rs3822030 | 247.38 |
| cg13485809 | 4 | 744,127 | intronic | *PCGF3* | BIOS | 0.07 | 28 | 1.08 (1.05 ± 1.11) | 6.07 × 10^-7^ | 2.57 × 10^-4^ | rs3822030 | 243.22 |
| cg13485809 | 4 | 744,127 | intronic | *TPCN1* | FHS | 0.07 | 8 | 1.13 (1.07 ± 1.19) | 2.70 × 10^-5^ | 5.67 × 10^-3^ | rs3822030 | 243.22 |
| cg07079724 | 4 | 780,591 | intronic | *CPLX1* | BIOS | 0.33 | 45 | 1.02 (1.01 ± 1.03) | 1.76 × 10^-4^ | 2.26 × 10^-2^ | rs3822030 | 206.75 |
| cg14183865 | 4 | 781,229 | intronic | *CPLX1* | BIOS | 0.24 | 19 | 1.03 (1.01 ± 1.04) | 3.00 × 10^-4^ | 3.28 × 10^-2^ | rs3822030 | 206.11 |
| cg04113154 | 4 | 875,278 | intronic | *GAK* | BIOS | 0.02 | 9 | 1.43 (1.21 ± 1.69) | 3.47 × 10^-5^ | 6.85 × 10^-3^ | rs3822030 | 112.07 |
| cg23092788 | 4 | 949,558 | exonic | *TMEM175* | BIOS | 0.01 | 21 | 0.87 (0.82 ± 0.93) | 4.27 × 10^-6^ | 1.27 × 10^-3^ | rs3822030 | 37.79 |
| cg06494872 | 4 | 959,128 | intronic | *DGKQ* | BIOS | 0.06 | 21 | 1.06 (1.03 ± 1.09) | 4.59 × 10^-5^ | 8.45 × 10^-3^ | rs3822030 | 28.22 |
| cg24158141 | 4 | 959,998 | intergenic | *C4BPA, CD55* | BIOS | 0.31 | 33 | 1.02 (1.01 ± 1.04) | 2.43 × 10^-5^ | 5.21 × 10^-3^ | rs3822030 | 27.35 |
| cg11107149 | 4 | 960,835 | exonic | *DGKQ* | BIOS | 0.17 | 52 | 1.04 (1.02 ± 1.06) | 2.10 × 10^-5^ | 4.63 × 10^-3^ | rs3822030 | 26.51 |
| cg07007312 | 4 | 961,505 | intronic | *DGKQ* | BIOS | 0.73 | 54 | 1.02 (1.01 ± 1.03) | **5.52 × 10^-8^** | 3.56 × 10^-5^ | rs3822030 | 25.84 |
| cg07007312 | 4 | 961,505 | intronic | *DGKQ* | FHS | 0.46 | 103 | 1.04 (1.02 ± 1.05) | 4.32 × 10^-5^ | 8.11 × 10^-3^ | rs3822030 | 25.84 |
| cg22718636 | 4 | 961,658 | intronic | *DGKQ* | BIOS | 0.72 | 75 | 1.02 (1.01 ± 1.03) | **1.47 × 10^-7^** | 7.89 × 10^-5^ | rs3822030 | 25.69 |
| cg20456258 | 4 | 962,124 | exonic | *DGKQ* | BIOS | 0.76 | 68 | 1.02 (1.02 ± 1.03) | **7.04 × 10^-8^** | 4.30 × 10^-5^ | rs3822030 | 25.22 |
| cg00788025 | 4 | 962,749 | intronic | *DGKQ* | BIOS | 0.02 | 25 | 1.09 (1.04 ± 1.13) | 5.23 × 10^-5^ | 9.27 × 10^-3^ | rs3822030 | 24.59 |
| cg15058036 | 4 | 982,443 | UTR3 | *SLC26A1* | BIOS | 0.02 | 4 | 0.86 (0.80 ± 0.93) | 8.07 × 10^-5^ | 1.29 × 10^-2^ | rs3822030 | 4.90 |
| cg25792473 | 4 | 1,029,484 | intergenic | *FGFRL1, RNF212* | BIOS | 0.01 | 8 | 0.88 (0.82 ± 0.95) | 3.77 × 10^-4^ | 3.82 × 10^-2^ | rs3822030 | -42.14 |
| cg23595451 | 4 | 1,035,462 | intergenic | *FGFRL1, RNF212* | BIOS | 0.06 | 48 | 1.06 (1.03 ± 1.09) | 3.00 × 10^-4^ | 3.28 × 10^-2^ | rs3822030 | -48.12 |
| cg17634650 | 4 | 10,966,220 | intergenic | *CLNK, MIR572* | BIOS | 0.01 | 4 | 1.17 (1.11 ± 1.23) | **6.27 × 10^-9^** | 5.40 × 10^-6^ | rs4504245 | 48.60 |
| cg27508378 | 4 | 10,985,133 | intergenic | *CLNK, MIR572* | BIOS | 0.07 | 26 | 0.95 (0.93 ± 0.97) | 4.23 × 10^-7^ | 1.88 × 10^-4^ | rs4504245 | 29.69 |
| cg27508378 | 4 | 10,985,133 | intergenic | *CLNK, MIR572* | FHS | 0.04 | 14 | 0.84 (0.78 ± 0.90) | 1.35 × 10^-6^ | 5.01 × 10^-4^ | rs4504245 | 29.69 |
| cg04203702 | 4 | 11,369,349 | intergenic | *CLNK, MIR572* | FHS | 0.06 | 54 | 1.13 (1.07 ± 1.18) | 2.46 × 10^-6^ | 8.08 × 10^-4^ | rs6448451 | -344.67 |
| cg07260927 | 4 | 11,428,822 | intronic | *HS3ST1* | FHS | 0.20 | 63 | 0.94 (0.91 ± 0.97) | 3.13 × 10^-4^ | 3.37 × 10^-2^ | rs6448451 | -404.14 |
| cg24029517 | 4 | 11,429,531 | intronic | *HS3ST1* | FHS | 0.09 | 25 | 1.18 (1.13 ± 1.24) | **2.51 × 10^-12^** | 6.73 × 10^-9^ | rs6448451 | -404.85 |
| cg02060717 | 4 | 40,197,355 | intronic | *RHOH* | FHS | 0.02 | 7 | 0.78 (0.68 ± 0.88) | 1.04 × 10^-4^ | 1.55 × 10^-2^ | rs2245466 | 1.49 |
| cg11284959 | 5 | 86,205,515 | intergenic | *LINC02059, MIR4280* | BIOS | 0.01 | 3 | 1.28 (1.19 ± 1.38) | **4.68 × 10^-11^** | 7.94 × 10^-8^ | rs62374257 | 17.68 |
| cg12408990 | 5 | 179,634,671 | intronic | *RASGEF1C* | FHS | 0.19 | 54 | 0.94 (0.92 ± 0.97) | 3.83 × 10^-4^ | 3.86 × 10^-2^ | rs113706587 | -6.52 |
| cg04650094 | 6 | 41,006,974 | upstream | *UNC5CL* | FHS | 0.30 | 61 | 0.95 (0.93 ± 0.97) | 3.15 × 10^-5^ | 6.30 × 10^-3^ | rs187370608 | -64.78 |
| cg22346765 | 6 | 41,007,522 | upstream | *UNC5CL* | FHS | 0.01 | 19 | 0.79 (0.71 ± 0.87) | 1.95 × 10^-6^ | 6.77 × 10^-4^ | rs187370608 | -65.33 |
| cg22346765 | 6 | 41,007,522 | upstream | *UNC5CL* | BIOS | 0.02 | 15 | 0.88 (0.83 ± 0.94) | 5.63 × 10^-5^ | 9.81 × 10^-3^ | rs187370608 | -65.33 |
| cg25533211 | 6 | 41,010,111 | intronic | *KANSL1* | BIOS | 0.01 | 21 | 0.88 (0.83 ± 0.93) | 1.06 × 10^-5^ | 2.66 × 10^-3^ | rs187370608 | -67.92 |
| cg17548735 | 6 | 41,020,969 | UTR5 | *APOBEC2* | FHS | 0.01 | 2 | 0.70 (0.61 ± 0.81) | 2.57 × 10^-6^ | 8.36 × 10^-4^ | rs187370608 | -78.77 |
| cg22375610 | 6 | 41,021,161 | intergenic | *NCK2, ECRG4* | BIOS | 0.01 | 6 | 0.84 (0.77 ± 0.91) | 6.30 × 10^-5^ | 1.07 × 10^-2^ | rs187370608 | -78.97 |
| cg22375610 | 6 | 41,021,161 | intronic | *BSX* | FHS | 0.02 | 12 | 0.77 (0.67 ± 0.88) | 9.90 × 10^-5^ | 1.49 × 10^-2^ | rs187370608 | -78.97 |
| cg10792307 | 6 | 41,123,086 | upstream | *TREML1* | FHS | 0.01 | 30 | 1.25 (1.14 ± 1.37) | 1.49 × 10^-6^ | 5.46 × 10^-4^ | rs187370608 | -180.89 |
| cg03526776 | 6 | 41,159,608 | UTR3 | *TREML2* | BIOS | 0.69 | 83 | 0.97 (0.96 ± 0.98) | **2.02 × 10^-10^** | 2.95 × 10^-7^ | rs187370608 | -217.41 |
| cg03526776 | 6 | 41,159,608 | UTR3 | *TREML2* | FHS | 0.51 | 42 | 0.95 (0.93 ± 0.96) | **5.12 × 10^-10^** | 6.65 × 10^-7^ | rs187370608 | -217.41 |
| cg12453228 | 6 | 41,338,345 | intergenic | *NCR2, LINC01276* | FHS | 0.03 | 59 | 1.14 (1.06 ± 1.22) | 1.65 × 10^-4^ | 2.16 × 10^-2^ | rs187370608 | -396.15 |
| cg09758420 | 6 | 41,343,830 | intergenic | *NCR2, LINC01276* | BIOS | 0.07 | 55 | 1.04 (1.02 ± 1.06) | 2.99 × 10^-4^ | 3.27 × 10^-2^ | rs187370608 | -401.63 |
| cg12968598 | 6 | 47,444,699 | upstream | *CD2AP* | BIOS | 0.61 | 17 | 1.03 (1.02 ± 1.04) | **3.76 × 10^-10^** | 5.04 × 10^-7^ | rs9381563 | -12.06 |
| cg02130027 | 6 | 47,444,894 | upstream | *CD2AP* | FHS | 0.11 | 18 | 1.15 (1.11 ± 1.20) | **8.24 × 10^-12^** | 1.94 × 10^-8^ | rs9381563 | -12.26 |
| cg02130027 | 6 | 47,444,894 | upstream | *CD2AP* | BIOS | 0.13 | 19 | 1.07 (1.05 ± 1.09) | **1.00 × 10^-10^** | 1.57 × 10^-7^ | rs9381563 | -12.26 |
| cg20196966 | 6 | 47,445,060 | upstream | *CD2AP* | FHS | 0.04 | 39 | 1.25 (1.17 ± 1.33) | **1.97 × 10^-11^** | 3.77 × 10^-8^ | rs9381563 | -12.42 |
| cg20196966 | 6 | 47,445,060 | upstream | *CD2AP* | BIOS | 0.07 | 10 | 1.09 (1.06 ± 1.13) | **7.08 × 10^-10^** | 8.85 × 10^-7^ | rs9381563 | -12.42 |
| cg20172563 | 6 | 47,487,173 | intronic | *CD2AP* | FHS | 0.20 | 37 | 1.10 (1.07 ± 1.13) | **9.95 × 10^-12^** | 2.18 × 10^-8^ | rs10948363 | 0.59 |
| cg20172563 | 6 | 47,487,173 | intronic | *CD2AP* | BIOS | 0.31 | 16 | 1.04 (1.02 ± 1.05) | **4.71 × 10^-9^** | 4.26 × 10^-6^ | rs10948363 | 0.59 |
| cg18651470 | 6 | 114,662,697 | downstream | *HDAC2-AS2* | BIOS | 0.04 | 13 | 0.93 (0.89 ± 0.97) | 3.45 × 10^-4^ | 3.62 × 10^-2^ | rs785129 | -49.80 |
| cg08203460 | 7 | 11,999,601 | exonic | *STX1B* | BIOS | 0.10 | 38 | 0.96 (0.94 ± 0.98) | 2.85 × 10^-5^ | 5.85 × 10^-3^ | rs5011436 | 269.16 |
| cg08203460 | 7 | 11,999,601 | UTR3 | *TAP2* | FHS | 0.05 | 51 | 0.89 (0.84 ± 0.94) | 3.63 × 10^-5^ | 7.11 × 10^-3^ | rs5011436 | 269.16 |
| cg19800032 | 7 | 12,134,189 | intergenic | *THSD7A, TMEM106B* | FHS | 0.19 | 64 | 0.94 (0.92 ± 0.97) | 2.01 × 10^-5^ | 4.48 × 10^-3^ | rs5011436 | 134.57 |
| cg19800032 | 7 | 12,134,189 | intergenic | *THSD7A, TMEM106B* | BIOS | 0.25 | 55 | 0.97 (0.96 ± 0.99) | 1.63 × 10^-4^ | 2.14 × 10^-2^ | rs5011436 | 134.57 |
| cg02853428 | 7 | 12,155,179 | intergenic | *THSD7A, TMEM106B* | FHS | 0.01 | 12 | 0.68 (0.58 ± 0.79) | 3.28 × 10^-7^ | 1.53 × 10^-4^ | rs5011436 | 113.58 |
| cg21238440 | 7 | 12,159,718 | intergenic | *THSD7A, TMEM106B* | FHS | 0.12 | 50 | 0.93 (0.90 ± 0.96) | 9.46 × 10^-6^ | 2.43 × 10^-3^ | rs5011436 | 109.04 |
| cg21238440 | 7 | 12,159,718 | intergenic | *THSD7A, TMEM106B* | BIOS | 0.21 | 35 | 0.97 (0.95 ± 0.98) | 5.88 × 10^-5^ | 1.01 × 10^-2^ | rs5011436 | 109.04 |
| cg01643690 | 7 | 12,228,583 | intergenic | *THSD7A, TMEM106B* | FHS | 0.03 | 13 | 0.80 (0.72 ± 0.87) | 1.50 × 10^-6^ | 5.47 × 10^-4^ | rs5011436 | 40.18 |
| cg01643690 | 7 | 12,228,583 | intergenic | *THSD7A, TMEM106B* | BIOS | 0.11 | 18 | 0.95 (0.93 ± 0.97) | 4.09 × 10^-5^ | 7.77 × 10^-3^ | rs5011436 | 40.18 |
| cg15443166 | 7 | 12,247,070 | intergenic | *THSD7A, TMEM106B* | FHS | 0.02 | 19 | 0.77 (0.70 ± 0.85) | **9.78 × 10^-8^** | 5.75 × 10^-5^ | rs5011436 | 21.69 |
| cg15443166 | 7 | 12,247,070 | intergenic | *THSD7A, TMEM106B* | BIOS | 0.04 | 6 | 0.90 (0.87 ± 0.94) | 6.12 × 10^-6^ | 1.71 × 10^-3^ | rs5011436 | 21.69 |
| cg22084182 | 7 | 27,973,667 | intronic | *JAZF1* | BIOS | 0.04 | 26 | 0.93 (0.90 ± 0.96) | 4.73 × 10^-5^ | 8.64 × 10^-3^ | rs1160871 | 195.08 |
| cg16130019 | 7 | 28,060,181 | intronic | *JAZF1* | FHS | 0.08 | 38 | 0.88 (0.84 ± 0.93) | **2.44 × 10^-7^** | 1.18 × 10^-4^ | rs1160871 | 108.56 |
| cg16130019 | 7 | 28,060,181 | intronic | *JAZF1* | BIOS | 0.07 | 35 | 0.96 (0.94 ± 0.98) | 2.45 × 10^-4^ | 2.85 × 10^-2^ | rs1160871 | 108.56 |
| cg26744081 | 7 | 28,176,110 | intronic | *JAZF1* | FHS | 0.05 | 10 | 1.20 (1.13 ± 1.29) | **4.22 × 10^-8^** | 2.86 × 10^-5^ | rs1160871 | -7.37 |
| cg26744081 | 7 | 28,176,110 | intronic | *JAZF1* | BIOS | 0.07 | 15 | 1.07 (1.05 ± 1.10) | **8.11 × 10^-8^** | 4.85 × 10^-5^ | rs1160871 | -7.37 |
| cg26438325 | 7 | 28,176,718 | intronic | *JAZF1* | BIOS | 0.15 | 46 | 1.05 (1.03 ± 1.06) | 2.42 × 10^-6^ | 7.97 × 10^-4^ | rs1160871 | -7.97 |
| cg25941154 | 7 | 28,208,557 | intronic | *PSORS1C1* | BIOS | 0.04 | 8 | 1.10 (1.06 ± 1.14) | **2.41 × 10^-7^** | 1.17 × 10^-4^ | rs1160871 | -39.81 |
| cg25941154 | 7 | 28,208,557 | exonic | *PPP1R13L* | FHS | 0.03 | 17 | 1.24 (1.14 ± 1.35) | 1.31 × 10^-6^ | 4.91 × 10^-4^ | rs1160871 | -39.81 |
| cg02010481 | 7 | 28,218,524 | intronic | *JAZF1* | BIOS | 0.01 | 6 | 1.15 (1.06 ± 1.23) | 3.08 × 10^-4^ | 3.34 × 10^-2^ | rs1160871 | -49.78 |
| cg26102728 | 7 | 28,218,933 | intronic | *JAZF1* | BIOS | 0.24 | 38 | 1.03 (1.02 ± 1.05) | 6.11 × 10^-6^ | 1.71 × 10^-3^ | rs1160871 | -50.19 |
| cg26102728 | 7 | 28,218,933 | intronic | *JAZF1* | FHS | 0.12 | 18 | 1.08 (1.04 ± 1.13) | 1.00 × 10^-4^ | 1.50 × 10^-2^ | rs1160871 | -50.19 |
| cg01883759 | 7 | 28,220,576 | ncRNA_intronic | *JAZF1-AS1* | FHS | 0.32 | 38 | 0.96 (0.94 ± 0.98) | 1.74 × 10^-4^ | 2.25 × 10^-2^ | rs1160871 | -51.83 |
| cg01883759 | 7 | 28,220,576 | ncRNA_intronic | *JAZF1-AS1* | BIOS | 0.33 | 38 | 0.98 (0.96 ± 0.99) | 4.77 × 10^-4^ | 4.51 × 10^-2^ | rs1160871 | -51.83 |
| cg21912938 | 7 | 28,220,594 | ncRNA_intronic | *JAZF1-AS1* | FHS | 0.12 | 21 | 0.93 (0.89 ± 0.97) | 3.12 × 10^-4^ | 3.37 × 10^-2^ | rs1160871 | -51.85 |
| cg24559796 | 7 | 28,223,298 | ncRNA_intronic | *JAZF1-AS1* | BIOS | 0.15 | 16 | 0.97 (0.95 ± 0.98) | 3.93 × 10^-5^ | 7.55 × 10^-3^ | rs1160871 | -54.55 |
| cg24559796 | 7 | 28,223,298 | ncRNA_intronic | *JAZF1-AS1* | FHS | 0.11 | 23 | 0.92 (0.88 ± 0.96) | 8.88 × 10^-5^ | 1.38 × 10^-2^ | rs1160871 | -54.55 |
| cg23418219 | 7 | 28,338,684 | upstream | *CREB5* | FHS | 0.04 | 25 | 0.82 (0.75 ± 0.90) | 5.99 × 10^-5^ | 1.03 × 10^-2^ | rs1160871 | -169.94 |
| cg14714629 | 7 | 99,495,452 | intronic | *TRIM4* | BIOS | 0.12 | 5 | 0.96 (0.93 ± 0.98) | 4.32 × 10^-4^ | 4.22 × 10^-2^ | rs7384878 | 436.60 |
| cg14714629 | 7 | 99,495,452 | intronic | *TRIM4* | FHS | 0.11 | 11 | 0.93 (0.89 ± 0.97) | 4.62 × 10^-4^ | 4.41 × 10^-2^ | rs7384878 | 436.60 |
| cg17152989 | 7 | 99,527,070 | exonic | *GJC3* | FHS | 0.01 | 39 | 0.85 (0.78 ± 0.92) | 1.35 × 10^-4^ | 1.87 × 10^-2^ | rs7384878 | 404.98 |
| cg19465374 | 7 | 99,573,278 | intronic | *AZGP1* | BIOS | 0.01 | 10 | 1.26 (1.17 ± 1.35) | **1.51 × 10^-10^** | 2.26 × 10^-7^ | rs7384878 | 358.77 |
| cg26429636 | 7 | 99,573,747 | upstream | *AZGP1* | FHS | 0.06 | 16 | 1.13 (1.07 ± 1.20) | 2.06 × 10^-5^ | 4.56 × 10^-3^ | rs7384878 | 358.30 |
| cg12019109 | 7 | 99,574,383 | upstream | *AZGP1* | FHS | 0.02 | 35 | 1.23 (1.15 ± 1.33) | **1.04 × 10^-8^** | 8.31 × 10^-6^ | rs7384878 | 357.67 |
| cg12019109 | 7 | 99,574,383 | upstream | *AZGP1* | BIOS | 0.04 | 4 | 1.10 (1.05 ± 1.15) | 7.46 × 10^-5^ | 1.22 × 10^-2^ | rs7384878 | 357.67 |
| cg13202523 | 7 | 99,588,273 | intergenic | *AZGP1P1, LOC105375423* | BIOS | 0.14 | 29 | 1.06 (1.04 ± 1.08) | **4.57 × 10^-8^** | 3.06 × 10^-5^ | rs7384878 | 343.78 |
| cg13202523 | 7 | 99,588,273 | intergenic | *AZGP1P1, LOC105375423* | FHS | 0.06 | 71 | 1.12 (1.08 ± 1.17) | **1.20 × 10^-7^** | 6.83 × 10^-5^ | rs7384878 | 343.78 |
| cg08582801 | 7 | 99,588,335 | intergenic | *AZGP1P1, LOC105375423* | BIOS | 0.10 | 27 | 1.06 (1.04 ± 1.08) | **2.39 × 10^-8^** | 1.79 × 10^-5^ | rs7384878 | 343.71 |
| cg08582801 | 7 | 99,588,335 | intergenic | *AZGP1P1, LOC105375423* | FHS | 0.05 | 11 | 1.18 (1.11 ± 1.25) | **7.22 × 10^-8^** | 4.37 × 10^-5^ | rs7384878 | 343.71 |
| cg14736458 | 7 | 99,595,658 | ncRNA_intronic | *LOC105375423* | FHS | 0.02 | 7 | 0.71 (0.64 ± 0.80) | **4.75 × 10^-9^** | 4.27 × 10^-6^ | rs7384878 | 336.39 |
| cg14736458 | 7 | 99,595,658 | ncRNA_intronic | *LOC105375423* | BIOS | 0.03 | 4 | 0.87 (0.83 ± 0.92) | **1.36 × 10^-7^** | 7.46 × 10^-5^ | rs7384878 | 336.39 |
| cg10547843 | 7 | 99,601,692 | ncRNA_intronic | *LOC105375423* | BIOS | 0.07 | 8 | 1.10 (1.07 ± 1.14) | **7.56 × 10^-10^** | 9.11 × 10^-7^ | rs7384878 | 330.36 |
| cg10547843 | 7 | 99,601,692 | ncRNA_intronic | *LOC105375423* | FHS | 0.05 | 45 | 1.15 (1.10 ± 1.22) | **5.18 × 10^-8^** | 3.42 × 10^-5^ | rs7384878 | 330.36 |
| cg04305808 | 7 | 99,614,209 | intronic | *ZKSCAN1* | FHS | 0.08 | 12 | 1.22 (1.14 ± 1.30) | **8.41 × 10^-9^** | 6.96 × 10^-6^ | rs7384878 | 317.84 |
| cg04305808 | 7 | 99,614,209 | intronic | *ZKSCAN1* | BIOS | 0.15 | 16 | 1.08 (1.05 ± 1.12) | 8.64 × 10^-6^ | 2.26 × 10^-3^ | rs7384878 | 317.84 |
| cg07461273 | 7 | 99,697,172 | intronic | *MCM7* | BIOS | 0.10 | 13 | 1.04 (1.02 ± 1.07) | 1.34 × 10^-4^ | 1.86 × 10^-2^ | rs7384878 | 234.88 |
| cg12358443 | 7 | 99,699,161 | UTR5 | *MCM7* | BIOS | 0.03 | 6 | 1.15 (1.10 ± 1.20) | **6.42 × 10^-10^** | 8.15 × 10^-7^ | rs7384878 | 232.89 |
| cg14002172 | 7 | 99,699,610 | exonic | *AP4M1* | BIOS | 0.04 | 7 | 0.92 (0.89 ± 0.96) | 3.66 × 10^-5^ | 7.14 × 10^-3^ | rs7384878 | 232.44 |
| cg11738421 | 7 | 99,699,621 | intronic | *AP4M1* | FHS | 0.05 | 15 | 0.88 (0.83 ± 0.94) | 3.72 × 10^-5^ | 7.20 × 10^-3^ | rs7384878 | 232.43 |
| cg11738421 | 7 | 99,699,621 | intronic | *AP4M1* | BIOS | 0.13 | 4 | 0.95 (0.93 ± 0.98) | 4.27 × 10^-5^ | 8.04 × 10^-3^ | rs7384878 | 232.43 |
| cg21810604 | 7 | 99,699,701 | intronic | *AP4M1* | FHS | 0.06 | 20 | 1.18 (1.12 ± 1.24) | **7.20 × 10^-10^** | 8.93 × 10^-7^ | rs7384878 | 232.35 |
| cg21810604 | 7 | 99,699,701 | intronic | *AP4M1* | BIOS | 0.14 | 9 | 1.06 (1.04 ± 1.08) | **1.43 × 10^-7^** | 7.72 × 10^-5^ | rs7384878 | 232.35 |
| cg08950287 | 7 | 99,725,807 | exonic | *MBLAC1* | BIOS | 0.04 | 17 | 1.12 (1.08 ± 1.16) | **5.95 × 10^-11^** | 9.80 × 10^-8^ | rs7384878 | 206.24 |
| cg08950287 | 7 | 99,725,807 | exonic | *MBLAC1* | FHS | 0.01 | 9 | 1.51 (1.30 ± 1.74) | **3.57 × 10^-8^** | 2.49 × 10^-5^ | rs7384878 | 206.24 |
| cg24109116 | 7 | 99,725,975 | UTR3 | *MBLAC1* | FHS | 0.03 | 6 | 1.38 (1.25 ± 1.52) | **2.81 × 10^-10^** | 3.99 × 10^-7^ | rs7384878 | 206.07 |
| cg24109116 | 7 | 99,725,975 | UTR3 | *MBLAC1* | BIOS | 0.05 | 22 | 1.08 (1.04 ± 1.12) | 8.17 × 10^-6^ | 2.17 × 10^-3^ | rs7384878 | 206.07 |
| cg22906224 | 7 | 99,728,672 | intergenic | *MBLAC1, LAMTOR4* | FHS | 0.50 | 56 | 0.95 (0.93 ± 0.96) | **1.92 × 10^-10^** | 2.85 × 10^-7^ | rs7384878 | 203.38 |
| cg22906224 | 7 | 99,728,672 | intergenic | *MBLAC1, LAMTOR4* | BIOS | 0.56 | 13 | 0.97 (0.96 ± 0.98) | **7.71 × 10^-10^** | 9.22 × 10^-7^ | rs7384878 | 203.38 |
| cg03818034 | 7 | 99,746,088 | upstream | *LAMTOR4* | BIOS | 0.02 | 7 | 1.20 (1.13 ± 1.28) | **2.13 × 10^-9^** | 2.13 × 10^-6^ | rs7384878 | 185.96 |
| cg03603286 | 7 | 99,746,475 | upstream | *LAMTOR4* | BIOS | 0.01 | 7 | 1.24 (1.16 ± 1.34) | **1.90 × 10^-9^** | 1.93 × 10^-6^ | rs7384878 | 185.57 |
| cg06189038 | 7 | 99,767,134 | upstream, downstream | *GAL3ST4, GPC2* | BIOS | 0.01 | 11 | 1.13 (1.06 ± 1.21) | 1.41 × 10^-4^ | 1.95 × 10^-2^ | rs7384878 | 164.92 |
| cg18090197 | 7 | 99,769,602 | intronic | *GPC2* | BIOS | 0.12 | 30 | 1.05 (1.03 ± 1.07) | **9.03 × 10^-8^** | 5.34 × 10^-5^ | rs7384878 | 162.45 |
| cg18090197 | 7 | 99,769,602 | UTR3 | *GIPR* | FHS | 0.05 | 16 | 1.16 (1.10 ± 1.23) | **1.24 × 10^-7^** | 6.94 × 10^-5^ | rs7384878 | 162.45 |
| cg00048759 | 7 | 99,775,422 | UTR5 | *STAG3* | FHS | 0.03 | 9 | 0.75 (0.69 ± 0.81) | **9.78 × 10^-12^** | 2.17 × 10^-8^ | rs7384878 | 156.63 |
| cg00048759 | 7 | 99,775,422 | UTR5 | *STAG3* | BIOS | 0.08 | 22 | 0.92 (0.90 ± 0.94) | **1.42 × 10^-11^** | 2.99 × 10^-8^ | rs7384878 | 156.63 |
| cg18691434 | 7 | 99,775,425 | UTR5 | *STAG3* | FHS | 0.06 | 12 | 0.82 (0.78 ± 0.87) | **6.28 × 10^-12^** | 1.51 × 10^-8^ | rs7384878 | 156.62 |
| cg18691434 | 7 | 99,775,425 | UTR5 | *STAG3* | BIOS | 0.10 | 13 | 0.92 (0.90 ± 0.95) | **2.08 × 10^-11^** | 3.89 × 10^-8^ | rs7384878 | 156.62 |
| cg13210467 | 7 | 99,775,443 | UTR5 | *STAG3* | FHS | 0.05 | 10 | 0.79 (0.74 ± 0.85) | **1.17 × 10^-12^** | 3.47 × 10^-9^ | rs7384878 | 156.61 |
| cg13210467 | 7 | 99,775,443 | UTR5 | *STAG3* | BIOS | 0.07 | 13 | 0.91 (0.88 ± 0.93) | **1.61 × 10^-11^** | 3.25 × 10^-8^ | rs7384878 | 156.61 |
| cg04778012 | 7 | 99,775,508 | UTR5 | *STAG3* | BIOS | 0.23 | 19 | 0.95 (0.94 ± 0.97) | **2.11 × 10^-11^** | 3.90 × 10^-8^ | rs7384878 | 156.54 |
| cg04778012 | 7 | 99,775,508 | UTR5 | *STAG3* | FHS | 0.11 | 22 | 0.89 (0.86 ± 0.92) | **8.03 × 10^-11^** | 1.28 × 10^-7^ | rs7384878 | 156.54 |
| cg10084644 | 7 | 99,775,521 | UTR5 | *STAG3* | BIOS | 0.20 | 9 | 0.94 (0.93 ± 0.96) | **7.11 × 10^-11^** | 1.16 × 10^-7^ | rs7384878 | 156.53 |
| cg10084644 | 7 | 99,775,521 | UTR5 | *STAG3* | FHS | 0.11 | 33 | 0.90 (0.86 ± 0.93) | **7.42 × 10^-10^** | 9.02 × 10^-7^ | rs7384878 | 156.53 |
| cg15140703 | 7 | 99,775,532 | UTR5 | *STAG3* | BIOS | 0.18 | 14 | 0.94 (0.93 ± 0.96) | **4.42 × 10^-11^** | 7.58 × 10^-8^ | rs7384878 | 156.52 |
| cg15140703 | 7 | 99,775,532 | UTR5 | *STAG3* | FHS | 0.13 | 39 | 0.90 (0.87 ± 0.93) | **1.41 × 10^-10^** | 2.13 × 10^-7^ | rs7384878 | 156.52 |
| cg00553149 | 7 | 99,775,558 | UTR5 | *STAG3* | BIOS | 0.30 | 15 | 0.96 (0.95 ± 0.97) | **1.15 × 10^-10^** | 1.79 × 10^-7^ | rs7384878 | 156.49 |
| cg00553149 | 7 | 99,775,558 | intergenic | *MDC1, TUBB* | FHS | 0.19 | 31 | 0.92 (0.89 ± 0.94) | **3.70 × 10^-10^** | 5.00 × 10^-7^ | rs7384878 | 156.49 |
| cg04697562 | 7 | 99,775,862 | intronic | *STAG3* | FHS | 0.01 | 12 | 0.66 (0.58 ± 0.74) | **1.54 × 10^-11^** | 3.16 × 10^-8^ | rs7384878 | 156.19 |
| cg04697562 | 7 | 99,775,862 | intronic | *TAOK2* | BIOS | 0.03 | 14 | 0.88 (0.84 ± 0.92) | **1.40 × 10^-7^** | 7.61 × 10^-5^ | rs7384878 | 156.19 |
| cg17830204 | 7 | 99,819,110 | UTR3 | *PVRIG* | FHS | 0.30 | 19 | 1.08 (1.05 ± 1.10) | **1.35 × 10^-10^** | 2.06 × 10^-7^ | rs7384878 | 112.94 |
| cg17830204 | 7 | 99,819,110 | UTR3 | *PVRIG* | BIOS | 0.38 | 18 | 1.03 (1.02 ± 1.05) | **2.59 × 10^-9^** | 2.55 × 10^-6^ | rs7384878 | 112.94 |
| cg14047091 | 7 | 100,205,640 | exonic | *PCOLCE* | BIOS | 0.03 | 20 | 0.92 (0.88 ± 0.95) | 1.43 × 10^-5^ | 3.46 × 10^-3^ | rs1476679 | -201.19 |
| cg05920870 | 7 | 100,205,729 | UTR3 | *PCOLCE* | BIOS | 0.02 | 14 | 0.87 (0.82 ± 0.92) | **1.48 × 10^-7^** | 7.89 × 10^-5^ | rs1476679 | -201.28 |
| cg02538169 | 7 | 100,287,481 | upstream | *GIGYF1* | BIOS | 0.02 | 5 | 1.08 (1.03 ± 1.13) | 5.08 × 10^-4^ | 4.70 × 10^-2^ | rs1476679 | -283.04 |
| cg02333467 | 7 | 100,290,845 | intergenic | *GIGYF1, POP7* | FHS | 0.03 | 25 | 1.20 (1.11 ± 1.31) | 1.15 × 10^-5^ | 2.86 × 10^-3^ | rs1476679 | -286.40 |
| cg02333467 | 7 | 100,290,845 | intronic | *GABBR1* | BIOS | 0.05 | 8 | 1.07 (1.04 ± 1.10) | 1.74 × 10^-5^ | 4.01 × 10^-3^ | rs1476679 | -286.40 |
| cg26426488 | 7 | 100,461,644 | intronic | *SLC12A9* | FHS | 0.02 | 23 | 0.79 (0.71 ± 0.88) | 1.66 × 10^-5^ | 3.88 × 10^-3^ | rs1476679 | -457.20 |
| cg05392513 | 7 | 100,483,616 | intronic | *SRRT* | BIOS | 0.06 | 8 | 0.93 (0.90 ± 0.97) | 3.19 × 10^-4^ | 3.42 × 10^-2^ | rs1476679 | -479.17 |
| cg10475014 | 7 | 100,484,435 | exonic | *SRRT* | FHS | 0.03 | 11 | 0.80 (0.71 ± 0.89) | 9.40 × 10^-5^ | 1.43 × 10^-2^ | rs1476679 | -479.99 |
| cg19077231 | 7 | 100,484,514 | intronic | *GABBR1* | FHS | 0.02 | 13 | 0.78 (0.70 ± 0.87) | 1.32 × 10^-5^ | 3.23 × 10^-3^ | rs1476679 | -480.07 |
| cg22813798 | 7 | 100,484,748 | exonic | *SRRT* | FHS | 0.20 | 28 | 0.94 (0.91 ± 0.97) | 2.78 × 10^-5^ | 5.76 × 10^-3^ | rs1476679 | -480.30 |
| cg22813798 | 7 | 100,484,748 | exonic | *SRRT* | BIOS | 0.31 | 8 | 0.97 (0.96 ± 0.99) | 1.92 × 10^-4^ | 2.39 × 10^-2^ | rs1476679 | -480.30 |
| cg25224090 | 7 | 100,486,248 | UTR3 | *SRRT* | FHS | 0.06 | 48 | 0.88 (0.83 ± 0.94) | 4.90 × 10^-5^ | 8.85 × 10^-3^ | rs1476679 | -481.80 |
| cg25224090 | 7 | 100,486,248 | UTR3 | *SRRT* | BIOS | 0.08 | 22 | 0.95 (0.92 ± 0.97) | 1.23 × 10^-4^ | 1.74 × 10^-2^ | rs1476679 | -481.80 |
| cg17365250 | 7 | 100,487,514 | upstream, downstream | *UFSP1, ACHE* | FHS | 0.01 | 20 | 0.75 (0.65 ± 0.88) | 2.33 × 10^-4^ | 2.75 × 10^-2^ | rs1476679 | -483.07 |
| cg16429505 | 7 | 100,488,398 | UTR3 | *ACHE* | BIOS | 0.02 | 24 | 0.93 (0.89 ± 0.97) | 3.83 × 10^-4^ | 3.86 × 10^-2^ | rs1476679 | -483.95 |
| cg04156293 | 7 | 100,488,942 | intergenic | *EXOC2, LOC101927691* | FHS | 0.02 | 8 | 1.22 (1.11 ± 1.35) | 7.97 × 10^-5^ | 1.28 × 10^-2^ | rs1476679 | -484.50 |
| cg04156293 | 7 | 100,488,942 | exonic | *ACHE* | BIOS | 0.03 | 23 | 1.08 (1.04 ± 1.13) | 3.11 × 10^-4^ | 3.37 × 10^-2^ | rs1476679 | -484.50 |
| cg22488857 | 7 | 100,489,022 | intronic | *ACHE* | BIOS | 0.02 | 4 | 1.13 (1.06 ± 1.20) | 2.64 × 10^-4^ | 2.99 × 10^-2^ | rs1476679 | -484.58 |
| cg16934685 | 7 | 143,060,037 | intronic | *FAM131B* | BIOS | 0.02 | 12 | 1.11 (1.06 ± 1.17) | 4.35 × 10^-5^ | 8.15 × 10^-3^ | rs3935067 | 44.29 |
| cg03489712 | 7 | 143,076,881 | ncRNA_exonic | *LOC100507507* | FHS | 0.07 | 58 | 0.85 (0.81 ± 0.89) | **2.24 × 10^-12^** | 6.11 × 10^-9^ | rs3935067 | 27.45 |
| cg03489712 | 7 | 143,076,881 | ncRNA_exonic | *LOC100507507* | BIOS | 0.08 | 36 | 0.95 (0.93 ± 0.97) | 1.94 × 10^-6^ | 6.76 × 10^-4^ | rs3935067 | 27.45 |
| cg03761471 | 7 | 143,077,178 | ncRNA_exonic | *LOC100507507* | FHS | 0.04 | 24 | 0.83 (0.78 ± 0.90) | 8.18 × 10^-7^ | 3.31 × 10^-4^ | rs3935067 | 27.15 |
| cg03761471 | 7 | 143,077,178 | ncRNA_exonic | *LOC100507507* | BIOS | 0.09 | 40 | 0.95 (0.94 ± 0.97) | 3.33 × 10^-6^ | 1.04 × 10^-3^ | rs3935067 | 27.15 |
| cg06631810 | 7 | 143,091,890 | intronic | *EPHA1* | FHS | 0.03 | 53 | 0.83 (0.79 ± 0.88) | **1.01 × 10^-9^** | 1.16 × 10^-6^ | rs3935067 | 12.44 |
| cg06631810 | 7 | 143,091,890 | intronic | *EPHA1* | BIOS | 0.06 | 24 | 0.95 (0.93 ± 0.97) | 3.73 × 10^-5^ | 7.21 × 10^-3^ | rs3935067 | 12.44 |
| cg11291798 | 7 | 143,103,859 | intronic | *EPHA1* | FHS | 0.04 | 23 | 1.13 (1.06 ± 1.21) | 2.61 × 10^-4^ | 2.97 × 10^-2^ | rs3935067 | 0.47 |
| cg05908241 | 7 | 143,109,367 | ncRNA_intronic | *EPHA1-AS1* | FHS | 0.30 | 24 | 1.09 (1.07 ± 1.12) | **4.13 × 10^-15^** | 2.02 × 10^-11^ | rs7810606 | -1.21 |
| cg05908241 | 7 | 143,109,367 | ncRNA_intronic | *EPHA1-AS1* | BIOS | 0.44 | 41 | 1.04 (1.03 ± 1.05) | **7.31 × 10^-14^** | 2.53 × 10^-10^ | rs7810606 | -1.21 |
| cg27033231 | 7 | 143,208,667 | exonic | *OR10AC1* | FHS | 0.14 | 93 | 0.91 (0.88 ± 0.94) | **1.71 × 10^-8^** | 1.32 × 10^-5^ | rs11771145 | -97.91 |
| cg27033231 | 7 | 143,208,667 | exonic | *OR10AC1* | BIOS | 0.20 | 37 | 0.96 (0.95 ± 0.98) | 3.05 × 10^-6^ | 9.66 × 10^-4^ | rs11771145 | -97.91 |
| cg22099723 | 8 | 27,348,453 | upstream | *EPHX2* | FHS | 0.19 | 26 | 1.11 (1.06 ± 1.16) | 8.12 × 10^-7^ | 3.29 × 10^-4^ | rs1532278 | 117.86 |
| cg22099723 | 8 | 27,348,453 | upstream | *EPHX2* | BIOS | 0.22 | 53 | 1.02 (1.01 ± 1.03) | 3.51 × 10^-4^ | 3.66 × 10^-2^ | rs1532278 | 117.86 |
| cg21666367 | 8 | 27,450,279 | upstream | *APOC4* | FHS | 0.01 | 31 | 1.31 (1.19 ± 1.45) | **1.34 × 10^-7^** | 7.38 × 10^-5^ | rs1532278 | 16.04 |
| cg21666367 | 8 | 27,450,279 | intergenic | *EPHX2, CLU* | BIOS | 0.08 | 56 | 1.06 (1.03 ± 1.08) | 1.00 × 10^-6^ | 3.90 × 10^-4^ | rs1532278 | 16.04 |
| cg24794833 | 8 | 27,450,748 | intergenic | *EPHX2, CLU* | FHS | 0.10 | 9 | 0.81 (0.76 ± 0.87) | **4.14 × 10^-9^** | 3.82 × 10^-6^ | rs1532278 | 15.57 |
| cg13879655 | 8 | 27,450,777 | intergenic | *EPHX2, CLU* | FHS | 0.24 | 39 | 0.87 (0.84 ± 0.91) | **9.26 × 10^-14^** | 3.14 × 10^-10^ | rs1532278 | 15.54 |
| cg13879655 | 8 | 27,450,777 | intergenic | *EPHX2, CLU* | BIOS | 0.32 | 81 | 0.96 (0.95 ± 0.97) | **2.51 × 10^-10^** | 3.60 × 10^-7^ | rs1532278 | 15.54 |
| cg16292768 | 8 | 27,467,783 | intronic | *CLU* | BIOS | 0.06 | 4 | 1.24 (1.20 ± 1.29) | **1.40 × 10^-35^** | 1.16 × 10-30 | rs9331896 | -0.10 |
| cg16292768 | 8 | 27,467,783 | intronic | *CLU* | FHS | 0.08 | 11 | 1.35 (1.29 ± 1.42) | **5.68 × 10^-33^** | 3.15 × 10-28 | rs9331896 | -0.10 |
| cg08594681 | 8 | 27,468,684 | intronic | *CLU* | BIOS | 0.02 | 17 | 1.34 (1.27 ± 1.43) | **1.32 × 10^-21^** | 2.20 × 10^-17^ | rs9331896 | -1.00 |
| cg13351161 | 8 | 27,490,921 | upstream | *SCARA3* | FHS | 0.22 | 32 | 0.93 (0.91 ± 0.96) | 5.32 × 10^-7^ | 2.29 × 10^-4^ | rs9331896 | -23.24 |
| cg13351161 | 8 | 27,490,921 | intronic | *SPI1* | BIOS | 0.30 | 35 | 0.97 (0.96 ± 0.98) | 1.80 × 10^-5^ | 4.13 × 10^-3^ | rs9331896 | -23.24 |
| cg24554944 | 8 | 27,776,888 | intronic | *SCARA5* | FHS | 0.02 | 38 | 1.16 (1.07 ± 1.25) | 2.16 × 10^-4^ | 2.62 × 10^-2^ | rs9331896 | -309.20 |
| cg24037389 | 8 | 27,845,182 | intronic | *SCARA5* | FHS | 0.02 | 29 | 0.83 (0.76 ± 0.91) | 8.40 × 10^-5^ | 1.33 × 10^-2^ | rs9331896 | -377.50 |
| cg13124863 | 8 | 27,850,175 | ncRNA_intronic | *FRG2KP* | FHS | 0.04 | 16 | 0.86 (0.79 ± 0.92) | 4.90 × 10^-5^ | 8.85 × 10^-3^ | rs9331896 | -382.49 |
| cg07634191 | 8 | 27,850,178 | UTR5 | *SCARA5* | FHS | 0.04 | 62 | 0.88 (0.83 ± 0.94) | 6.39 × 10^-5^ | 1.08 × 10^-2^ | rs9331896 | -382.49 |
| cg17960164 | 8 | 145,018,928 | UTR5 | *PLEC* | BIOS | 0.01 | 12 | 1.12 (1.05 ± 1.20) | 3.71 × 10^-4^ | 3.78 × 10^-2^ | rs61732533 | 89.22 |
| cg05457903 | 8 | 145,052,304 | intronic | *PARP10* | BIOS | 0.09 | 18 | 1.05 (1.02 ± 1.08) | 3.19 × 10^-4^ | 3.42 × 10^-2^ | rs61732533 | 55.85 |
| cg14313833 | 9 | 107,666,037 | UTR5 | *ABCA1* | BIOS | 0.10 | 6 | 0.95 (0.94 ± 0.97) | **1.19 × 10^-7^** | 6.80 × 10^-5^ | rs1800978 | -0.06 |
| cg14313833 | 9 | 107,666,037 | UTR5 | *ABCA1* | FHS | 0.20 | 63 | 0.94 (0.91 ± 0.96) | 9.26 × 10^-7^ | 3.67 × 10^-4^ | rs1800978 | -0.06 |
| cg20716064 | 10 | 11,652,950 | intronic | *USP6NL* | BIOS | 0.01 | 13 | 1.17 (1.09 ± 1.25) | 1.35 × 10^-5^ | 3.30 × 10^-3^ | rs11257238 | 64.45 |
| cg03475172 | 10 | 11,654,177 | ncRNA_exonic | *LOC107984208* | FHS | 0.01 | 13 | 0.72 (0.62 ± 0.83) | 3.54 × 10^-6^ | 1.09 × 10^-3^ | rs11257238 | 63.22 |
| cg03475172 | 10 | 11,654,177 | ncRNA_exonic | *LOC107984208* | BIOS | 0.01 | 12 | 0.90 (0.86 ± 0.95) | 2.92 × 10^-4^ | 3.21 × 10^-2^ | rs11257238 | 63.22 |
| cg15320596 | 10 | 61,604,738 | intronic | *CCDC6* | BIOS | 0.31 | 26 | 0.97 (0.96 ± 0.99) | 2.36 × 10^-4^ | 2.78 × 10^-2^ | rs7902657 | 133.41 |
| cg15320596 | 10 | 61,604,738 | intronic | *CCDC6* | FHS | 0.24 | 20 | 0.96 (0.93 ± 0.98) | 5.28 × 10^-4^ | 4.83 × 10^-2^ | rs7902657 | 133.41 |
| cg14316118 | 10 | 61,668,184 | intergenic | *CCDC6, LINC01553* | FHS | 0.02 | 26 | 1.18 (1.08 ± 1.28) | 3.42 × 10^-4^ | 3.60 × 10^-2^ | rs7902657 | 69.97 |
| cg23914149 | 10 | 61,725,773 | intergenic | *LINC01553, ANK3* | BIOS | 0.02 | 20 | 1.10 (1.05 ± 1.16) | 1.49 × 10^-4^ | 2.01 × 10^-2^ | rs7902657 | 12.38 |
| cg23637396 | 10 | 81,891,666 | upstream | *PLAC9* | BIOS | 0.08 | 33 | 1.04 (1.02 ± 1.06) | 2.79 × 10^-4^ | 3.10 × 10^-2^ | rs6586028 | 362.32 |
| cg19049754 | 10 | 81,966,990 | upstream | *LINC00857* | BIOS | 0.02 | 80 | 1.05 (1.02 ± 1.07) | 8.28 × 10^-5^ | 1.31 × 10^-2^ | rs6586028 | 286.99 |
| cg06813382 | 10 | 81,967,195 | upstream | *LINC00857* | BIOS | 0.05 | 61 | 1.05 (1.03 ± 1.07) | 3.93 × 10^-7^ | 1.77 × 10^-4^ | rs6586028 | 286.79 |
| cg11647108 | 10 | 81,967,516 | ncRNA_exonic | *LINC00857* | BIOS | 0.05 | 60 | 1.04 (1.02 ± 1.06) | 3.83 × 10^-7^ | 1.73 × 10^-4^ | rs6586028 | 286.47 |
| cg09614479 | 10 | 81,967,536 | ncRNA_exonic | *LINC00857* | BIOS | 0.06 | 42 | 1.05 (1.03 ± 1.07) | 2.30 × 10^-6^ | 7.62 × 10^-4^ | rs6586028 | 286.45 |
| cg10775231 | 10 | 81,967,666 | ncRNA_exonic | *LINC00857* | BIOS | 0.05 | 63 | 1.05 (1.03 ± 1.08) | **1.71 × 10^-7^** | 8.83 × 10^-5^ | rs6586028 | 286.32 |
| cg10125882 | 10 | 82,024,011 | ncRNA_exonic | *LOC101929574* | FHS | 0.01 | 30 | 1.22 (1.11 ± 1.35) | 8.10 × 10^-5^ | 1.29 × 10^-2^ | rs6586028 | 229.97 |
| cg18292394 | 10 | 82,049,450 | upstream | *MAT1A* | FHS | 0.05 | 16 | 1.19 (1.10 ± 1.28) | 8.08 × 10^-6^ | 2.15 × 10^-3^ | rs6586028 | 204.53 |
| cg25535587 | 10 | 82,116,456 | UTR5 | *DYDC1* | FHS | 0.05 | 48 | 0.83 (0.78 ± 0.88) | **3.88 × 10^-10^** | 5.16 × 10^-7^ | rs6586028 | 137.53 |
| cg25535587 | 10 | 82,116,456 | UTR5 | *DYDC1* | BIOS | 0.06 | 14 | 0.92 (0.89 ± 0.95) | 5.21 × 10^-7^ | 2.26 × 10^-4^ | rs6586028 | 137.53 |
| cg01527459 | 10 | 82,116,571 | UTR5 | *DYDC2* | BIOS | 0.02 | 3 | 0.84 (0.79 ± 0.90) | **2.03 × 10^-7^** | 1.01 × 10^-4^ | rs6586028 | 137.41 |
| cg01527459 | 10 | 82,116,571 | UTR5 | *DYDC2* | FHS | 0.01 | 9 | 0.69 (0.59 ± 0.80) | 2.24 × 10^-6^ | 7.49 × 10^-4^ | rs6586028 | 137.41 |
| cg06411551 | 10 | 82,117,119 | intronic | *DYDC2* | FHS | 0.13 | 19 | 0.89 (0.86 ± 0.92) | **3.21 × 10^-9^** | 3.03 × 10^-6^ | rs6586028 | 136.87 |
| cg06411551 | 10 | 82,117,119 | intronic | *DYDC2* | BIOS | 0.21 | 12 | 0.95 (0.94 ± 0.97) | **8.17 × 10^-9^** | 6.79 × 10^-6^ | rs6586028 | 136.87 |
| cg07862129 | 10 | 82,122,651 | ncRNA_exonic | *APOC4* | BIOS | 0.07 | 10 | 1.09 (1.06 ± 1.13) | **1.65 × 10^-9^** | 1.71 × 10^-6^ | rs6586028 | 131.33 |
| cg07862129 | 10 | 82,122,651 | intronic | *DYDC2* | FHS | 0.05 | 31 | 1.22 (1.14 ± 1.30) | **3.74 × 10^-9^** | 3.47 × 10^-6^ | rs6586028 | 131.33 |
| cg25372335 | 10 | 82,168,065 | intronic | *PRXL2A* | FHS | 0.02 | 35 | 0.84 (0.77 ± 0.92) | 1.19 × 10^-4^ | 1.71 × 10^-2^ | rs6586028 | 85.92 |
| cg16751764 | 10 | 82,168,743 | intronic | *PRXL2A* | BIOS | 0.02 | 14 | 0.92 (0.88 ± 0.96) | 3.25 × 10^-4^ | 3.47 × 10^-2^ | rs6586028 | 85.24 |
| cg07091481 | 10 | 82,169,149 | intronic | *PRXL2A* | FHS | 0.03 | 12 | 0.83 (0.76 ± 0.91) | 1.14 × 10^-4^ | 1.65 × 10^-2^ | rs6586028 | 84.84 |
| cg05901522 | 10 | 82,169,480 | intronic | *PRXL2A* | FHS | 0.01 | 28 | 1.23 (1.10 ± 1.37) | 1.74 × 10^-4^ | 2.25 × 10^-2^ | rs6586028 | 84.50 |
| cg05014245 | 10 | 82,172,262 | intronic | *PRXL2A* | FHS | 0.04 | 11 | 1.13 (1.06 ± 1.21) | 4.09 × 10^-4^ | 4.06 × 10^-2^ | rs6586028 | 81.72 |
| cg19862899 | 10 | 82,173,556 | intronic | *PRXL2A* | FHS | 0.10 | 23 | 0.91 (0.86 ± 0.95) | 4.12 × 10^-5^ | 7.81 × 10^-3^ | rs6586028 | 80.43 |
| cg12069540 | 10 | 82,173,570 | intronic | *PRXL2A* | FHS | 0.08 | 22 | 0.91 (0.86 ± 0.95) | 8.20 × 10^-5^ | 1.30 × 10^-2^ | rs6586028 | 80.41 |
| cg05327931 | 10 | 82,178,480 | intronic | *PRXL2A* | FHS | 0.10 | 34 | 1.11 (1.06 ± 1.15) | 1.50 × 10^-6^ | 5.47 × 10^-4^ | rs6586028 | 75.50 |
| cg00277334 | 10 | 82,204,260 | intergenic | *PRXL2A, TSPAN14* | FHS | 0.75 | 51 | 0.97 (0.95 ± 0.98) | 1.85 × 10^-6^ | 6.53 × 10^-4^ | rs6586028 | 49.72 |
| cg00277334 | 10 | 82,204,260 | intergenic | *PRXL2A, TSPAN14* | BIOS | 0.79 | 28 | 0.98 (0.97 ± 0.99) | 4.57 × 10^-6^ | 1.34 × 10^-3^ | rs6586028 | 49.72 |
| cg03267442 | 10 | 82,210,566 | intergenic | *PRXL2A, TSPAN14* | BIOS | 0.71 | 78 | 0.98 (0.97 ± 0.99) | 6.96 × 10^-7^ | 2.89 × 10^-4^ | rs6586028 | 43.42 |
| cg09626299 | 10 | 82,213,104 | upstream | *TSPAN14* | FHS | 0.63 | 55 | 0.96 (0.94 ± 0.97) | **4.21 × 10^-9^** | 3.85 × 10^-6^ | rs6586028 | 40.88 |
| cg09626299 | 10 | 82,213,104 | intergenic | *PRDM7, FAM157B* | BIOS | 0.70 | 42 | 0.98 (0.97 ± 0.99) | **1.84 × 10^-7^** | 9.24 × 10^-5^ | rs6586028 | 40.88 |
| cg10780367 | 10 | 82,213,323 | upstream | *TSPAN14* | BIOS | 0.15 | 18 | 0.94 (0.93 ± 0.96) | **1.96 × 10^-9^** | 1.98 × 10^-6^ | rs6586028 | 40.66 |
| cg10780367 | 10 | 82,213,323 | upstream | *TSPAN14* | FHS | 0.13 | 35 | 0.90 (0.86 ± 0.93) | **1.42 × 10^-8^** | 1.11 × 10^-5^ | rs6586028 | 40.66 |
| cg23858360 | 10 | 82,213,490 | upstream | *TSPAN14* | BIOS | 0.13 | 39 | 0.94 (0.92 ± 0.96) | **2.14 × 10^-8^** | 1.62 × 10^-5^ | rs6586028 | 40.49 |
| cg23858360 | 10 | 82,213,490 | upstream | *TSPAN14* | FHS | 0.09 | 35 | 0.90 (0.86 ± 0.94) | 8.77 × 10^-7^ | 3.52 × 10^-4^ | rs6586028 | 40.49 |
| cg06685340 | 10 | 82,213,598 | upstream | *TSPAN14* | FHS | 0.02 | 25 | 0.72 (0.66 ± 0.80) | **9.89 × 10^-11^** | 1.57 × 10^-7^ | rs6586028 | 40.39 |
| cg06685340 | 10 | 82,213,598 | upstream | *TSPAN14* | BIOS | 0.11 | 31 | 0.94 (0.93 ± 0.96) | **1.72 × 10^-8^** | 1.32 × 10^-5^ | rs6586028 | 40.39 |
| cg22593342 | 10 | 82,219,410 | intronic | *TSPAN14* | FHS | 0.12 | 45 | 0.92 (0.89 ± 0.95) | 2.09 × 10^-6^ | 7.12 × 10^-4^ | rs6586028 | 34.57 |
| cg22593342 | 10 | 82,219,410 | intronic | *TSPAN14* | BIOS | 0.14 | 42 | 0.96 (0.95 ± 0.98) | 1.49 × 10^-4^ | 2.01 × 10^-2^ | rs6586028 | 34.57 |
| cg16260349 | 10 | 82,221,597 | intronic | *TSPAN14* | FHS | 0.06 | 29 | 0.83 (0.79 ± 0.88) | **1.07 × 10^-9^** | 1.21 × 10^-6^ | rs6586028 | 32.39 |
| cg16260349 | 10 | 82,221,597 | intronic | *TSPAN14* | BIOS | 0.11 | 32 | 0.94 (0.92 ± 0.97) | 1.76 × 10^-6^ | 6.27 × 10^-4^ | rs6586028 | 32.39 |
| cg24741873 | 10 | 82,223,998 | intronic | *TSPAN14* | BIOS | 0.02 | 7 | 0.84 (0.79 ± 0.89) | **1.72 × 10^-9^** | 1.78 × 10^-6^ | rs6586028 | 29.99 |
| cg23463186 | 10 | 82,263,737 | intronic | *TSPAN14* | BIOS | 0.03 | 10 | 1.16 (1.12 ± 1.21) | **7.10 × 10^-14^** | 2.51 × 10^-10^ | rs6586028 | -9.75 |
| cg16178415 | 10 | 82,265,445 | intronic | *TSPAN14* | FHS | 0.14 | 11 | 1.09 (1.05 ± 1.13) | 1.98 × 10^-6^ | 6.82 × 10^-4^ | rs6586028 | -11.46 |
| cg16178415 | 10 | 82,265,445 | intronic | *TSPAN14* | BIOS | 0.13 | 8 | 1.05 (1.03 ± 1.08) | 4.71 × 10^-6^ | 1.37 × 10^-3^ | rs6586028 | -11.46 |
| cg13612642 | 10 | 82,291,886 | ncRNA_intronic | *LOC101929574* | FHS | 0.05 | 12 | 1.20 (1.12 ± 1.28) | 4.35 × 10^-7^ | 1.91 × 10^-4^ | rs6586028 | -37.90 |
| cg13612642 | 10 | 82,291,886 | ncRNA_intronic | *LOC101929574* | BIOS | 0.06 | 16 | 1.05 (1.02 ± 1.08) | 2.28 × 10^-4^ | 2.71 × 10^-2^ | rs6586028 | -37.90 |
| cg08989478 | 10 | 82,295,394 | intergenic | *LOC100130698, MAT1A* | FHS | 0.09 | 19 | 1.10 (1.05 ± 1.15) | 8.10 × 10^-5^ | 1.29 × 10^-2^ | rs6586028 | -41.41 |
| cg08989478 | 10 | 82,295,394 | ncRNA_exonic | *LOC101929574* | BIOS | 0.11 | 34 | 1.04 (1.02 ± 1.07) | 2.31 × 10^-4^ | 2.73 × 10^-2^ | rs6586028 | -41.41 |
| cg09623377 | 10 | 82,296,191 | upstream | *LOC101929574* | FHS | 0.05 | 35 | 1.12 (1.07 ± 1.19) | 1.51 × 10^-5^ | 3.60 × 10^-3^ | rs6586028 | -42.21 |
| cg12575674 | 10 | 82,362,642 | intronic | *SH2D4B* | FHS | 0.03 | 32 | 1.15 (1.06 ± 1.24) | 3.20 × 10^-4^ | 3.43 × 10^-2^ | rs6586028 | -108.66 |
| cg06643284 | 10 | 98,009,748 | intronic | *BLNK* | BIOS | 0.02 | 26 | 1.10 (1.05 ± 1.15) | 2.13 × 10^-5^ | 4.69 × 10^-3^ | rs6584063 | 16.66 |
| cg07397033 | 10 | 98,031,201 | upstream | *CSNK1G2* | BIOS | 0.04 | 28 | 1.06 (1.03 ± 1.09) | 3.59 × 10^-5^ | 7.05 × 10^-3^ | rs6584063 | -4.79 |
| cg08901339 | 10 | 98,031,261 | UTR5 | *BLNK* | BIOS | 0.04 | 23 | 1.05 (1.03 ± 1.08) | 1.43 × 10^-4^ | 1.96 × 10^-2^ | rs6584063 | -4.85 |
| cg08901339 | 10 | 98,031,261 | UTR5 | *BLNK* | FHS | 0.01 | 29 | 1.18 (1.07 ± 1.29) | 5.51 × 10^-4^ | 4.96 × 10^-2^ | rs6584063 | -4.85 |
| cg19935065 | 10 | 98,062,687 | intergenic | *BLNK, DNTT* | FHS | 0.22 | 42 | 1.08 (1.05 ± 1.11) | 6.95 × 10^-7^ | 2.89 × 10^-4^ | rs6584063 | -36.28 |
| cg19935065 | 10 | 98,062,687 | intergenic | *BLNK, DNTT* | BIOS | 0.27 | 64 | 1.03 (1.02 ± 1.04) | 3.20 × 10^-6^ | 1.00 × 10^-3^ | rs6584063 | -36.28 |
| cg20728490 | 10 | 98,064,175 | upstream | *DNTT* | FHS | 0.30 | 70 | 1.07 (1.05 ± 1.10) | **2.45 × 10^-8^** | 1.82 × 10^-5^ | rs6584063 | -37.77 |
| cg20728490 | 10 | 98,064,175 | upstream | *DNTT* | BIOS | 0.41 | 68 | 1.03 (1.02 ± 1.04) | **1.56 × 10^-7^** | 8.21 × 10^-5^ | rs6584063 | -37.77 |
| cg24949488 | 10 | 98,064,362 | exonic | *DNTT* | BIOS | 0.19 | 26 | 1.04 (1.03 ± 1.06) | 6.00 × 10^-7^ | 2.55 × 10^-4^ | rs6584063 | -37.96 |
| cg24949488 | 10 | 98,064,362 | exonic | *DNTT* | FHS | 0.07 | 28 | 1.14 (1.08 ± 1.20) | 6.96 × 10^-7^ | 2.89 × 10^-4^ | rs6584063 | -37.96 |
| cg18278519 | 10 | 98,069,610 | intronic | *DNTT* | BIOS | 0.11 | 23 | 1.04 (1.02 ± 1.06) | 2.23 × 10^-4^ | 2.67 × 10^-2^ | rs6584063 | -43.20 |
| cg00091760 | 10 | 124,131,072 | intergenic | *BTBD16, PLEKHA1* | BIOS | 0.01 | 12 | 0.83 (0.77 ± 0.90) | 3.19 × 10^-6^ | 1.00 × 10^-3^ | rs7908662 | 41.84 |
| cg18222240 | 10 | 124,213,527 | upstream | *ARMS2* | BIOS | 0.02 | 8 | 0.90 (0.86 ± 0.95) | 2.11 × 10^-4^ | 2.56 × 10^-2^ | rs7908662 | -40.62 |
| cg00676728 | 10 | 124,213,760 | upstream | *ARMS2* | BIOS | 0.15 | 11 | 1.03 (1.01 ± 1.05) | 2.08 × 10^-4^ | 2.54 × 10^-2^ | rs7908662 | -40.85 |
| cg00676728 | 10 | 124,213,760 | upstream | *ARMS2* | FHS | 0.13 | 11 | 1.07 (1.03 ± 1.11) | 2.70 × 10^-4^ | 3.04 × 10^-2^ | rs7908662 | -40.85 |
| cg24296920 | 10 | 124,214,120 | upstream | *ARMS2* | FHS | 0.05 | 21 | 1.13 (1.06 ± 1.21) | 8.74 × 10^-5^ | 1.37 × 10^-2^ | rs7908662 | -41.21 |
| cg08447739 | 10 | 124,220,359 | upstream | *HTRA1* | FHS | 0.04 | 56 | 0.88 (0.83 ± 0.93) | 1.88 × 10^-5^ | 4.28 × 10^-3^ | rs7908662 | -47.45 |
| cg09576143 | 10 | 124,220,367 | upstream | *HTRA1* | FHS | 0.03 | 40 | 0.89 (0.83 ± 0.95) | 2.62 × 10^-4^ | 2.97 × 10^-2^ | rs7908662 | -47.46 |
| cg02711929 | 10 | 124,220,396 | upstream | *HTRA1* | FHS | 0.07 | 38 | 0.91 (0.86 ± 0.95) | 1.21 × 10^-4^ | 1.73 × 10^-2^ | rs7908662 | -47.48 |
| cg25446361 | 10 | 124,220,504 | upstream | *HTRA1* | BIOS | 0.35 | 45 | 0.98 (0.97 ± 0.99) | 2.10 × 10^-4^ | 2.56 × 10^-2^ | rs7908662 | -47.59 |
| cg25446361 | 10 | 124,220,504 | upstream | *HTRA1* | FHS | 0.28 | 39 | 0.96 (0.94 ± 0.98) | 2.79 × 10^-4^ | 3.10 × 10^-2^ | rs7908662 | -47.59 |
| cg03070550 | 10 | 124,247,208 | intronic | *HTRA1* | BIOS | 0.21 | 78 | 1.03 (1.01 ± 1.04) | 4.30 × 10^-4^ | 4.21 × 10^-2^ | rs7908662 | -74.30 |
| cg22801913 | 11 | 47,176,841 | intronic | *C11orf49* | FHS | 0.02 | 22 | 0.84 (0.78 ± 0.91) | 9.93 × 10^-6^ | 2.52 × 10^-3^ | rs3740688 | 203.50 |
| cg22801913 | 11 | 47,176,841 | intronic | *C11orf49* | BIOS | 0.02 | 8 | 0.91 (0.87 ± 0.96) | 1.92 × 10^-4^ | 2.39 × 10^-2^ | rs3740688 | 203.50 |
| cg24909660 | 11 | 47,276,469 | intronic | *NR1H3* | FHS | 0.03 | 19 | 1.15 (1.07 ± 1.24) | 1.36 × 10^-4^ | 1.88 × 10^-2^ | rs3740688 | 103.87 |
| cg16317516 | 11 | 47,294,196 | intronic | *MADD* | FHS | 0.01 | 5 | 0.70 (0.61 ± 0.81) | 8.99 × 10^-7^ | 3.59 × 10^-4^ | rs3740688 | 86.14 |
| cg16317516 | 11 | 47,294,196 | intronic | *MADD* | BIOS | 0.02 | 6 | 0.89 (0.83 ± 0.95) | 5.18 × 10^-4^ | 4.77 × 10^-2^ | rs3740688 | 86.14 |
| cg03711944 | 11 | 47,377,212 | intronic | *SPI1* | FHS | 0.02 | 6 | 1.38 (1.25 ± 1.51) | **2.86 × 10^-11^** | 5.17 × 10^-8^ | rs3740688 | 3.13 |
| cg03711944 | 11 | 47,377,212 | intronic | *SPI1* | BIOS | 0.10 | 9 | 1.08 (1.05 ± 1.10) | **1.13 × 10^-9^** | 1.27 × 10^-6^ | rs3740688 | 3.13 |
| cg02647874 | 11 | 47,380,751 | upstream | *SCARA3* | FHS | 0.01 | 16 | 1.22 (1.12 ± 1.34) | 1.80 × 10^-5^ | 4.13 × 10^-3^ | rs3740688 | -0.41 |
| cg02647874 | 11 | 47,380,751 | intronic | *SPI1* | BIOS | 0.06 | 15 | 1.05 (1.02 ± 1.09) | 3.54 × 10^-4^ | 3.68 × 10^-2^ | rs3740688 | -0.41 |
| cg07698783 | 11 | 47,395,915 | intronic | *SPI1* | BIOS | 0.04 | 22 | 0.90 (0.87 ± 0.93) | **1.59 × 10^-9^** | 1.68 × 10^-6^ | rs3740688 | -15.58 |
| cg07698783 | 11 | 47,395,915 | intronic | *SPI1* | FHS | 0.02 | 37 | 0.83 (0.78 ± 0.89) | **1.51 × 10^-8^** | 1.17 × 10^-5^ | rs3740688 | -15.58 |
| cg07675031 | 11 | 47,399,893 | intronic | *RNF43* | FHS | 0.02 | 7 | 1.30 (1.15 ± 1.46) | 1.57 × 10^-5^ | 3.71 × 10^-3^ | rs3740688 | -19.55 |
| cg04692506 | 11 | 47,401,027 | upstream | *SPI1* | BIOS | 0.09 | 10 | 1.05 (1.02 ± 1.08) | 3.22 × 10^-4^ | 3.45 × 10^-2^ | rs3740688 | -20.69 |
| cg19668951 | 11 | 47,430,812 | intronic | *SLC39A13* | BIOS | 0.04 | 4 | 1.10 (1.05 ± 1.15) | 9.11 × 10^-6^ | 2.35 × 10^-3^ | rs3740688 | -50.47 |
| cg19668951 | 11 | 47,430,812 | intronic | *PCGF3* | FHS | 0.02 | 15 | 1.21 (1.11 ± 1.32) | 1.23 × 10^-5^ | 3.03 × 10^-3^ | rs3740688 | -50.47 |
| cg17614165 | 11 | 47,468,663 | intronic | *RASAL1* | BIOS | 0.02 | 17 | 1.11 (1.06 ± 1.16) | 2.20 × 10^-5^ | 4.80 × 10^-3^ | rs3740688 | -88.32 |
| cg19460817 | 11 | 47,575,278 | upstream | *CELF1* | BIOS | 0.05 | 27 | 0.94 (0.92 ± 0.96) | **1.82 × 10^-7^** | 9.20 × 10^-5^ | rs10838725 | -17.41 |
| cg25953688 | 11 | 47,575,283 | upstream | *CELF1* | BIOS | 0.04 | 31 | 0.93 (0.90 ± 0.95) | **1.09 × 10^-8^** | 8.67 × 10^-6^ | rs10838725 | -17.41 |
| cg25953688 | 11 | 47,575,283 | upstream | *CELF1* | FHS | 0.01 | 15 | 0.79 (0.71 ± 0.88) | 1.97 × 10^-5^ | 4.41 × 10^-3^ | rs10838725 | -17.41 |
| cg19198483 | 11 | 47,616,096 | upstream | *C1QTNF4* | BIOS | 0.03 | 33 | 1.08 (1.03 ± 1.12) | 3.01 × 10^-4^ | 3.29 × 10^-2^ | rs10838725 | -58.23 |
| cg16516316 | 11 | 47,619,408 | intergenic | *C1QTNF4, MTCH2* | BIOS | 0.02 | 8 | 1.11 (1.06 ± 1.17) | 4.02 × 10^-5^ | 7.67 × 10^-3^ | rs10838725 | -61.54 |
| cg05585544 | 11 | 47,624,801 | intergenic | *C1QTNF4, MTCH2* | FHS | 0.15 | 16 | 1.08 (1.04 ± 1.11) | 1.25 × 10^-5^ | 3.08 × 10^-3^ | rs10838725 | -66.93 |
| cg05585544 | 11 | 47,624,801 | intergenic | *C1QTNF4, MTCH2* | BIOS | 0.15 | 2 | 1.05 (1.02 ± 1.07) | 2.53 × 10^-5^ | 5.39 × 10^-3^ | rs10838725 | -66.93 |
| cg20135002 | 11 | 47,629,003 | intergenic | *C1QTNF4, MTCH2* | FHS | 0.26 | 27 | 1.05 (1.03 ± 1.07) | 4.00 × 10^-5^ | 7.66 × 10^-3^ | rs10838725 | -71.13 |
| cg20135002 | 11 | 47,629,003 | intronic | *SORL1* | BIOS | 0.34 | 8 | 1.03 (1.01 ± 1.04) | 5.66 × 10^-5^ | 9.84 × 10^-3^ | rs10838725 | -71.13 |
| cg18512352 | 11 | 47,633,146 | intergenic | *C1QTNF4, MTCH2* | BIOS | 0.50 | 19 | 1.02 (1.01 ± 1.04) | 2.10 × 10^-5^ | 4.63 × 10^-3^ | rs10838725 | -75.28 |
| cg18512352 | 11 | 47,633,146 | intergenic | *C1QTNF4, MTCH2* | FHS | 0.39 | 14 | 1.04 (1.02 ± 1.06) | 4.03 × 10^-5^ | 7.69 × 10^-3^ | rs10838725 | -75.28 |
| cg08540622 | 11 | 59,806,898 | upstream | *OOSP2* | BIOS | 0.02 | 14 | 1.18 (1.11 ± 1.24) | **2.85 × 10^-9^** | 2.74 × 10^-6^ | rs983392 | 116.61 |
| cg08505647 | 11 | 59,807,594 | upstream | *OOSP2* | BIOS | 0.07 | 20 | 1.07 (1.04 ± 1.10) | 1.88 × 10^-6^ | 6.58 × 10^-4^ | rs983392 | 115.91 |
| cg08111960 | 11 | 59,822,765 | intergenic | *OOSP2, MS4A3* | BIOS | 0.04 | 21 | 1.11 (1.07 ± 1.15) | **6.69 × 10^-8^** | 4.12 × 10^-5^ | rs983392 | 100.74 |
| cg01917716 | 11 | 59,824,541 | intronic | *MS4A3* | BIOS | 0.24 | 20 | 1.04 (1.02 ± 1.06) | 1.80 × 10^-6^ | 6.40 × 10^-4^ | rs983392 | 98.97 |
| cg01917716 | 11 | 59,824,541 | intronic | *MS4A3* | FHS | 0.13 | 12 | 1.09 (1.05 ± 1.13) | 3.03 × 10^-6^ | 9.63 × 10^-4^ | rs983392 | 98.97 |
| cg02771260 | 11 | 59,836,817 | intronic | *MS4A3* | BIOS | 0.77 | 19 | 1.03 (1.02 ± 1.04) | **8.83 × 10^-10^** | 1.03 × 10^-6^ | rs983392 | 86.69 |
| cg02771260 | 11 | 59,836,817 | intronic | *MS4A3* | FHS | 0.66 | 29 | 1.04 (1.03 ± 1.06) | **1.80 × 10^-9^** | 1.85 × 10^-6^ | rs983392 | 86.69 |
| cg18774435 | 11 | 59,838,540 | UTR3 | *MS4A3* | FHS | 0.53 | 30 | 1.06 (1.04 ± 1.08) | **1.21 × 10^-12^** | 3.53 × 10^-9^ | rs983392 | 84.97 |
| cg18774435 | 11 | 59,838,540 | UTR3 | *MS4A3* | BIOS | 0.64 | 8 | 1.03 (1.02 ± 1.04) | **5.72 × 10^-12^** | 1.44 × 10^-8^ | rs983392 | 84.97 |
| cg09905416 | 11 | 59,861,219 | intronic | *MS4A2* | BIOS | 0.02 | 5 | 1.35 (1.26 ± 1.45) | **1.58 × 10^-17^** | 1.08 × 10^-13^ | rs983392 | 62.29 |
| cg09905416 | 11 | 59,861,219 | intronic | *MS4A2* | FHS | 0.01 | 13 | 1.75 (1.54 ± 2.00) | **1.86 × 10^-17^** | 1.19 × 10^-13^ | rs983392 | 62.29 |
| cg04353769 | 11 | 59,951,557 | intronic | *MS4A6A* | BIOS | 0.01 | 8 | 0.69 (0.64 ± 0.75) | **1.17 × 10^-19^** | 1.22 × 10^-15^ | rs983392 | -28.05 |
| cg06881914 | 11 | 59,951,663 | intronic | *MS4A6A* | FHS | 0.16 | 19 | 0.86 (0.83 ± 0.89) | **7.89 × 10^-19^** | 6.25 × 10^-15^ | rs983392 | -28.16 |
| cg06881914 | 11 | 59,951,663 | intronic | *MS4A6A* | BIOS | 0.13 | 32 | 0.91 (0.89 ± 0.93) | **4.98 × 10^-16^** | 2.76 × 10^-12^ | rs983392 | -28.16 |
| cg23754934 | 11 | 60,047,483 | upstream | *MS4A4A* | FHS | 0.33 | 20 | 1.04 (1.02 ± 1.06) | 5.16 × 10^-4^ | 4.76 × 10^-2^ | rs1582763 | -25.54 |
| cg01229998 | 11 | 60,049,447 | intronic | *MS4A4A* | FHS | 0.04 | 36 | 1.21 (1.14 ± 1.29) | **1.01 × 10^-9^** | 1.16 × 10^-6^ | rs1582763 | -27.50 |
| cg27312916 | 11 | 85,469,117 | UTR5 | *SYTL2* | FHS | 0.02 | 19 | 0.76 (0.68 ± 0.86) | 6.43 × 10^-6^ | 1.77 × 10^-3^ | rs561655 | 331.16 |
| cg20891655 | 11 | 85,630,744 | UTR3 | *CCDC83* | BIOS | 0.01 | 4 | 1.10 (1.04 ± 1.16) | 4.52 × 10^-4^ | 4.35 × 10^-2^ | rs561655 | 169.54 |
| cg13536080 | 11 | 85,659,096 | intergenic | *CCDC83, PICALM* | BIOS | 0.02 | 28 | 0.89 (0.85 ± 0.93) | **2.29 × 10^-7^** | 1.13 × 10^-4^ | rs561655 | 141.18 |
| cg23478124 | 11 | 85,776,966 | intronic | *PICALM* | BIOS | 0.07 | 26 | 1.09 (1.05 ± 1.12) | **2.81 × 10^-8^** | 2.02 × 10^-5^ | rs561655 | 23.31 |
| cg01120308 | 11 | 85,780,971 | exonic | *CHRNE* | BIOS | 0.02 | 13 | 0.87 (0.82 ± 0.92) | 9.10 × 10^-7^ | 3.61 × 10^-4^ | rs561655 | 19.31 |
| cg07180834 | 11 | 85,838,833 | intergenic | *PICALM, EED* | FHS | 0.35 | 20 | 0.89 (0.87 ± 0.92) | **1.84 × 10^-20^** | 2.19 × 10^-16^ | rs3844143 | 11.41 |
| cg07180834 | 11 | 85,838,833 | intergenic | *PICALM, EED* | BIOS | 0.46 | 11 | 0.95 (0.94 ± 0.96) | **5.71 × 10^-19^** | 5.00 × 10^-15^ | rs3844143 | 11.41 |
| cg01904978 | 11 | 85,847,072 | intergenic | *PICALM, EED* | BIOS | 0.06 | 40 | 1.15 (1.12 ± 1.18) | **7.85 × 10^-22^** | 1.45 × 10^-17^ | rs3844143 | 3.17 |
| cg01904978 | 11 | 85,847,072 | intergenic | *PICALM, EED* | FHS | 0.06 | 11 | 1.32 (1.25 ± 1.40) | **9.64 × 10^-21^** | 1.34 × 10^-16^ | rs3844143 | 3.17 |
| cg04895225 | 11 | 85,862,822 | intergenic | *PICALM, EED* | FHS | 0.18 | 4 | 1.14 (1.10 ± 1.18) | **2.73 × 10^-15^** | 1.42 × 10^-11^ | rs10792832 | 5.05 |
| cg04895225 | 11 | 85,862,822 | intergenic | *PICALM, EED* | BIOS | 0.22 | 33 | 1.05 (1.04 ± 1.07) | **1.92 × 10^-12^** | 5.41 × 10^-9^ | rs10792832 | 5.05 |
| cg25500444 | 11 | 86,086,101 | exonic | *ACHE* | FHS | 0.13 | 41 | 1.07 (1.04 ± 1.11) | 7.97 × 10^-5^ | 1.28 × 10^-2^ | rs10792832 | -218.23 |
| cg25219643 | 11 | 86,106,473 | intronic | *CCDC81* | BIOS | 0.02 | 37 | 1.08 (1.04 ± 1.12) | 2.37 × 10^-4^ | 2.78 × 10^-2^ | rs10792832 | -238.60 |
| cg08441314 | 11 | 121,446,689 | intergenic | *C1QTNF4, MTCH2* | FHS | 0.01 | 15 | 1.30 (1.15 ± 1.48) | 5.66 × 10^-5^ | 9.84 × 10^-3^ | rs11218343 | -11.10 |
| cg03055520 | 11 | 121,460,793 | exonic | *SORL1* | BIOS | 0.03 | 31 | 1.09 (1.04 ± 1.13) | 3.09 × 10^-5^ | 6.21 × 10^-3^ | rs11218343 | -25.21 |
| cg20069407 | 11 | 121,460,973 | intronic | *SORL1* | BIOS | 0.03 | 39 | 1.10 (1.06 ± 1.13) | **5.46 × 10^-8^** | 3.53 × 10^-5^ | rs11218343 | -25.39 |
| cg21593001 | 12 | 113,531,060 | intronic | *DTX1* | BIOS | 0.04 | 47 | 0.94 (0.91 ± 0.97) | 5.67 × 10^-5^ | 9.84 × 10^-3^ | rs6489896 | 188.73 |
| cg19098710 | 12 | 113,541,698 | intronic | *RASAL1* | FHS | 0.02 | 7 | 1.31 (1.14 ± 1.49) | 9.09 × 10^-5^ | 1.40 × 10^-2^ | rs6489896 | 178.09 |
| cg04227961 | 12 | 113,541,993 | exonic | *RASAL1* | FHS | 0.04 | 9 | 1.16 (1.07 ± 1.26) | 3.80 × 10^-4^ | 3.84 × 10^-2^ | rs6489896 | 177.80 |
| cg26621780 | 12 | 113,547,430 | intronic | *RASAL1* | FHS | 0.04 | 17 | 1.16 (1.07 ± 1.27) | 4.29 × 10^-4^ | 4.20 × 10^-2^ | rs6489896 | 172.36 |
| cg05157791 | 12 | 113,551,036 | intronic | *RASAL1* | FHS | 0.05 | 17 | 1.14 (1.06 ± 1.22) | 2.58 × 10^-4^ | 2.95 × 10^-2^ | rs6489896 | 168.75 |
| cg05139152 | 12 | 113,572,930 | intronic | *RASAL1* | FHS | 0.05 | 9 | 1.17 (1.08 ± 1.27) | 2.26 × 10^-4^ | 2.69 × 10^-2^ | rs6489896 | 146.86 |
| cg05139152 | 12 | 113,572,930 | intronic | *RASAL1* | BIOS | 0.05 | 19 | 1.03 (1.01 ± 1.05) | 3.83 × 10^-4^ | 3.86 × 10^-2^ | rs6489896 | 146.86 |
| cg06521025 | 12 | 113,573,143 | intronic | *RASAL1* | FHS | 0.01 | 20 | 1.35 (1.19 ± 1.53) | 1.97 × 10^-6^ | 6.80 × 10^-4^ | rs6489896 | 146.65 |
| cg06521025 | 12 | 113,573,143 | intronic | *RAPSN* | BIOS | 0.03 | 30 | 1.06 (1.03 ± 1.09) | 2.20 × 10^-5^ | 4.80 × 10^-3^ | rs6489896 | 146.65 |
| cg01110620 | 12 | 113,573,312 | UTR5 | *RASAL1* | BIOS | 0.02 | 55 | 1.04 (1.02 ± 1.06) | 1.52 × 10^-4^ | 2.03 × 10^-2^ | rs6489896 | 146.48 |
| cg15120925 | 12 | 113,573,809 | intronic | *RASAL1* | FHS | 0.06 | 10 | 0.88 (0.83 ± 0.94) | 1.46 × 10^-4^ | 1.99 × 10^-2^ | rs6489896 | 145.98 |
| cg21584422 | 12 | 113,573,942 | intronic | *CD302, LY75-CD302* | FHS | 0.02 | 23 | 0.78 (0.69 ± 0.88) | 2.95 × 10^-5^ | 6.01 × 10^-3^ | rs6489896 | 145.85 |
| cg21584422 | 12 | 113,573,942 | UTR5 | *RASAL1* | BIOS | 0.02 | 2 | 0.94 (0.91 ± 0.97) | 5.32 × 10^-4^ | 4.87 × 10^-2^ | rs6489896 | 145.85 |
| cg05149307 | 12 | 113,574,071 | intronic | *USP6* | FHS | 0.01 | 7 | 0.76 (0.67 ± 0.87) | 2.75 × 10^-5^ | 5.72 × 10^-3^ | rs6489896 | 145.72 |
| cg02939139 | 12 | 113,574,204 | upstream | *RASAL1* | FHS | 0.12 | 7 | 0.90 (0.85 ± 0.95) | 1.53 × 10^-4^ | 2.04 × 10^-2^ | rs6489896 | 145.58 |
| cg08865522 | 12 | 113,574,276 | upstream | *RASAL1* | FHS | 0.20 | 36 | 0.93 (0.89 ± 0.96) | 3.89 × 10^-5^ | 7.49 × 10^-3^ | rs6489896 | 145.51 |
| cg08865522 | 12 | 113,574,276 | upstream | *RASAL1* | BIOS | 0.27 | 5 | 0.99 (0.98 ± 0.99) | 3.71 × 10^-4^ | 3.78 × 10^-2^ | rs6489896 | 145.51 |
| cg09253125 | 12 | 113,658,854 | UTR5 | *IQCD* | BIOS | 0.01 | 14 | 0.90 (0.86 ± 0.95) | 3.45 × 10^-5^ | 6.83 × 10^-3^ | rs6489896 | 60.93 |
| cg11930955 | 12 | 113,660,045 | intronic | *TPCN1* | FHS | 0.03 | 7 | 0.79 (0.72 ± 0.87) | 3.07 × 10^-6^ | 9.69 × 10^-4^ | rs6489896 | 59.74 |
| cg11930955 | 12 | 113,660,045 | exonic | *ZNF668* | BIOS | 0.03 | 10 | 0.94 (0.91 ± 0.97) | 2.70 × 10^-5^ | 5.67 × 10^-3^ | rs6489896 | 59.74 |
| cg24281668 | 12 | 113,661,270 | intronic | *TPCN1* | BIOS | 0.09 | 23 | 1.04 (1.02 ± 1.06) | 8.74 × 10^-6^ | 2.27 × 10^-3^ | rs6489896 | 58.52 |
| cg24281668 | 12 | 113,661,270 | intronic | *TPCN1* | FHS | 0.03 | 56 | 1.13 (1.05 ± 1.21) | 4.80 × 10^-4^ | 4.54 × 10^-2^ | rs6489896 | 58.52 |
| cg02766895 | 12 | 113,662,240 | intronic | *TPCN1* | BIOS | 0.01 | 7 | 0.90 (0.86 ± 0.94) | 3.27 × 10^-6^ | 1.02 × 10^-3^ | rs6489896 | 57.55 |
| cg15125438 | 12 | 113,684,389 | intronic | *TPCN1* | BIOS | 0.07 | 8 | 0.96 (0.95 ± 0.98) | 8.24 × 10^-6^ | 2.18 × 10^-3^ | rs6489896 | 35.40 |
| cg15125438 | 12 | 113,684,389 | intronic | *TPCN1* | FHS | 0.01 | 32 | 0.77 (0.68 ± 0.87) | 4.43 × 10^-5^ | 8.24 × 10^-3^ | rs6489896 | 35.40 |
| cg12914100 | 12 | 113,734,359 | UTR3 | *TPCN1* | FHS | 0.04 | 11 | 1.19 (1.11 ± 1.29) | 6.15 × 10^-6^ | 1.71 × 10^-3^ | rs6489896 | -14.57 |
| cg12914100 | 12 | 113,734,359 | UTR3 | *TPCN1* | BIOS | 0.05 | 5 | 1.05 (1.03 ± 1.07) | 3.46 × 10^-5^ | 6.84 × 10^-3^ | rs6489896 | -14.57 |
| cg21498475 | 12 | 113,737,469 | UTR3 | *SLC8B1* | BIOS | 0.02 | 11 | 0.92 (0.88 ± 0.95) | 6.90 × 10^-6^ | 1.88 × 10^-3^ | rs6489896 | -17.68 |
| cg21208029 | 14 | 53,196,377 | upstream | *STYX* | BIOS | 0.19 | 5 | 1.04 (1.02 ± 1.06) | 4.50 × 10^-4^ | 4.34 × 10^-2^ | rs7146179 | 102.48 |
| cg18696900 | 14 | 53,419,080 | intergenic | *FERMT2, DDHD1* | BIOS | 0.02 | 21 | 1.15 (1.09 ± 1.22) | **1.05 × 10^-7^** | 6.11 × 10^-5^ | rs17125944 | -18.45 |
| cg03493774 | 14 | 92,879,474 | intronic | *SLC24A4* | BIOS | 0.09 | 50 | 0.95 (0.93 ± 0.97) | 1.72 × 10^-5^ | 3.98 × 10^-3^ | rs10498633 | 47.48 |
| cg03493774 | 14 | 92,879,474 | intronic | *SLC24A4* | FHS | 0.05 | 47 | 0.90 (0.84 ± 0.95) | 3.47 × 10^-4^ | 3.64 × 10^-2^ | rs10498633 | 47.48 |
| cg19811934 | 14 | 92,927,763 | intronic | *SLC24A4* | FHS | 0.42 | 70 | 1.06 (1.04 ± 1.08) | **4.38 × 10^-10^** | 5.74 × 10^-7^ | rs10498633 | -0.81 |
| cg19811934 | 14 | 92,927,763 | intronic | *SLC24A4* | BIOS | 0.53 | 74 | 1.02 (1.01 ± 1.03) | **3.10 × 10^-8^** | 2.21 × 10^-5^ | rs10498633 | -0.81 |
| cg11107966 | 14 | 92,927,875 | intronic | *SLC24A4* | FHS | 0.42 | 66 | 1.05 (1.03 ± 1.07) | **8.81 × 10^-9^** | 7.25 × 10^-6^ | rs10498633 | -0.92 |
| cg11107966 | 14 | 92,927,875 | intronic | *SLC24A4* | BIOS | 0.52 | 46 | 1.02 (1.01 ± 1.03) | 3.37 × 10^-7^ | 1.57 × 10^-4^ | rs10498633 | -0.92 |
| cg16757332 | 14 | 92,956,197 | intronic | *SLC24A4* | FHS | 0.07 | 18 | 1.14 (1.09 ± 1.20) | 3.58 × 10^-7^ | 1.63 × 10^-4^ | rs12590654 | -17.34 |
| cg16757332 | 14 | 92,956,197 | intronic | *SLC24A4* | BIOS | 0.07 | 23 | 1.05 (1.03 ± 1.08) | 1.41 × 10^-4^ | 1.95 × 10^-2^ | rs12590654 | -17.34 |
| cg14021523 | 14 | 92,959,873 | exonic | *SLC24A4* | FHS | 0.06 | 5 | 1.13 (1.06 ± 1.21) | 1.76 × 10^-4^ | 2.26 × 10^-2^ | rs12590654 | -21.02 |
| cg05200313 | 14 | 92,960,827 | UTR3 | *SLC24A4* | BIOS | 0.03 | 11 | 1.14 (1.08 ± 1.21) | 8.78 × 10^-7^ | 3.52 × 10^-4^ | rs12590654 | -21.97 |
| cg05200313 | 14 | 92,960,827 | UTR3 | *SLC24A4* | FHS | 0.02 | 43 | 1.20 (1.11 ± 1.31) | 1.52 × 10^-5^ | 3.62 × 10^-3^ | rs12590654 | -21.97 |
| cg13256308 | 14 | 93,106,286 | intronic | *RIN3* | BIOS | 0.16 | 41 | 0.97 (0.96 ± 0.99) | 2.69 × 10^-5^ | 5.67 × 10^-3^ | rs12590654 | -167.43 |
| cg16255729 | 14 | 93,106,394 | intronic | *RIN3* | FHS | 0.02 | 13 | 0.78 (0.70 ± 0.88) | 2.78 × 10^-5^ | 5.76 × 10^-3^ | rs12590654 | -167.54 |
| cg21075986 | 14 | 106,666,649 | intergenic | *ADAM6, LINC00226* | FHS | 0.03 | 28 | 1.41 (1.19 ± 1.66) | 6.42 × 10^-5^ | 1.08 × 10^-2^ | rs7157106 | -438.55 |
| cg10169449 | 14 | 106,938,915 | ncRNA_intronic | *LINC00221* | FHS | 0.01 | 17 | 1.36 (1.15 ± 1.62) | 3.52 × 10^-4^ | 3.67 × 10^-2^ | rs10131280 | 182.69 |
| cg16774084 | 14 | 107,210,989 | intergenic | *LINC00221, MIR5195* | FHS | 0.01 | 7 | 0.76 (0.66 ± 0.88) | 2.43 × 10^-4^ | 2.84 × 10^-2^ | rs10131280 | -89.38 |
| cg20968717 | 15 | 58,758,468 | ncRNA_intronic | *LIPC-AS1* | BIOS | 0.02 | 18 | 0.92 (0.88 ± 0.96) | 4.40 × 10^-4^ | 4.27 × 10^-2^ | rs442495 | 264.15 |
| cg24848787 | 15 | 58,844,359 | intronic | *LIPC* | FHS | 0.20 | 16 | 1.07 (1.04 ± 1.10) | 1.99 × 10^-5^ | 4.44 × 10^-3^ | rs442495 | 178.26 |
| cg20800039 | 15 | 58,891,233 | intronic | *ADAM10* | BIOS | 0.11 | 27 | 0.96 (0.94 ± 0.98) | 2.04 × 10^-5^ | 4.54 × 10^-3^ | rs442495 | 131.38 |
| cg08898775 | 15 | 59,042,684 | upstream | *ADAM10* | FHS | 0.07 | 20 | 0.83 (0.78 ± 0.87) | **5.81 × 10^-12^** | 1.44 × 10^-8^ | rs602602 | 14.34 |
| cg08898775 | 15 | 59,042,684 | upstream | *ADAM10* | BIOS | 0.12 | 33 | 0.93 (0.92 ± 0.95) | **9.25 × 10^-12^** | 2.11 × 10^-8^ | rs602602 | 14.34 |
| cg25595834 | 15 | 59,156,878 | intergenic | *MINDY2, SLTM* | FHS | 0.02 | 11 | 1.22 (1.11 ± 1.35) | 3.48 × 10^-5^ | 6.86 × 10^-3^ | rs602602 | -99.86 |
| cg21820656 | 15 | 59,157,075 | intergenic | *MINDY2, SLTM* | BIOS | 0.04 | 21 | 1.07 (1.03 ± 1.10) | 3.62 × 10^-4^ | 3.73 × 10^-2^ | rs602602 | -100.05 |
| cg08889009 | 15 | 59,157,123 | intergenic | *MINDY2, SLTM* | BIOS | 0.11 | 15 | 1.04 (1.02 ± 1.07) | 4.04 × 10^-5^ | 7.70 × 10^-3^ | rs602602 | -100.10 |
| cg08889009 | 15 | 59,157,123 | intergenic | *LINC00533, LINC01623* | FHS | 0.05 | 16 | 1.16 (1.08 ± 1.25) | 4.99 × 10^-5^ | 8.95 × 10^-3^ | rs602602 | -100.10 |
| cg26218411 | 15 | 59,226,409 | upstream | *SLTM* | BIOS | 0.02 | 30 | 1.09 (1.05 ± 1.14) | 7.19 × 10^-6^ | 1.94 × 10^-3^ | rs602602 | -169.39 |
| cg21221455 | 15 | 63,342,288 | intronic | *TPM1* | FHS | 0.09 | 25 | 0.91 (0.87 ± 0.95) | 2.22 × 10^-5^ | 4.83 × 10^-3^ | rs117618017 | 227.61 |
| cg21221455 | 15 | 63,342,288 | intronic | *TPM1* | BIOS | 0.10 | 53 | 0.96 (0.94 ± 0.98) | 3.77 × 10^-5^ | 7.28 × 10^-3^ | rs117618017 | 227.61 |
| cg11936410 | 15 | 63,345,124 | intronic | *TPM1* | BIOS | 0.02 | 18 | 0.90 (0.86 ± 0.94) | 1.03 × 10^-5^ | 2.60 × 10^-3^ | rs117618017 | 224.78 |
| cg07064595 | 15 | 63,426,843 | intronic | *LACTB* | BIOS | 0.01 | 8 | 1.14 (1.07 ± 1.22) | 1.10 × 10^-4^ | 1.62 × 10^-2^ | rs117618017 | 143.06 |
| cg02747950 | 15 | 63,526,883 | intronic | *RAB8B* | FHS | 0.03 | 13 | 1.18 (1.08 ± 1.30) | 2.62 × 10^-4^ | 2.97 × 10^-2^ | rs117618017 | 43.02 |
| cg21888989 | 15 | 63,569,245 | upstream | *APH1B* | FHS | 0.05 | 16 | 1.18 (1.11 ± 1.25) | **5.17 × 10^-8^** | 3.42 × 10^-5^ | rs117618017 | 0.66 |
| cg21888989 | 15 | 63,569,245 | upstream | *APH1B* | BIOS | 0.08 | 4 | 1.09 (1.06 ± 1.12) | **7.09 × 10^-8^** | 4.31 × 10^-5^ | rs117618017 | 0.66 |
| cg21064333 | 15 | 63,613,902 | UTR3 | *CA12* | FHS | 0.02 | 2 | 0.73 (0.64 ± 0.83) | 1.58 × 10^-6^ | 5.71 × 10^-4^ | rs117618017 | -44.00 |
| cg03491459 | 15 | 63,613,976 | UTR3 | *CA12* | FHS | 0.03 | 5 | 0.81 (0.75 ± 0.89) | 3.86 × 10^-6^ | 1.18 × 10^-3^ | rs117618017 | -44.07 |
| cg03491459 | 15 | 63,613,976 | UTR3 | *CA12* | BIOS | 0.07 | 16 | 0.95 (0.93 ± 0.98) | 1.28 × 10^-4^ | 1.80 × 10^-2^ | rs117618017 | -44.07 |
| cg04192393 | 15 | 63,673,286 | intronic | *CA12* | FHS | 0.02 | 14 | 1.22 (1.09 ± 1.36) | 5.37 × 10^-4^ | 4.90 × 10^-2^ | rs117618017 | -103.38 |
| cg15755265 | 15 | 63,673,502 | intronic | *CA12* | FHS | 0.02 | 16 | 1.26 (1.14 ± 1.38) | 1.55 × 10^-6^ | 5.62 × 10^-4^ | rs117618017 | -103.60 |
| cg15755265 | 15 | 63,673,502 | intronic | *CA12* | BIOS | 0.04 | 2 | 1.07 (1.04 ± 1.11) | 3.89 × 10^-6^ | 1.18 × 10^-3^ | rs117618017 | -103.60 |
| cg07015354 | 15 | 63,675,552 | intergenic | *CA12, LINC02568* | FHS | 0.04 | 4 | 1.18 (1.09 ± 1.29) | 8.49 × 10^-5^ | 1.34 × 10^-2^ | rs117618017 | -105.65 |
| cg07015354 | 15 | 63,675,552 | intergenic | *CA12, LINC02568* | BIOS | 0.01 | 13 | 1.12 (1.06 ± 1.19) | 1.21 × 10^-4^ | 1.72 × 10^-2^ | rs117618017 | -105.65 |
| cg26167930 | 15 | 64,363,681 | intergenic | *DAPK2, CIAO2A* | BIOS | 0.05 | 13 | 0.94 (0.92 ± 0.97) | 1.06 × 10^-5^ | 2.66 × 10^-3^ | rs3848143 | 59.83 |
| cg06153467 | 16 | 29,647,520 | upstream | *RRAD* | FHS | 0.08 | 9 | 1.10 (1.05 ± 1.15) | 2.92 × 10^-5^ | 5.97 × 10^-3^ | rs1140239 | 373.88 |
| cg09010156 | 16 | 29,836,211 | intronic | *MVP* | BIOS | 0.04 | 9 | 0.93 (0.89 ± 0.97) | 4.39 × 10^-4^ | 4.27 × 10^-2^ | rs1140239 | 185.19 |
| cg01827781 | 16 | 29,875,145 | ncRNA_intronic | *CDIPTOSP* | BIOS | 0.14 | 10 | 0.97 (0.95 ± 0.98) | 2.90 × 10^-4^ | 3.19 × 10^-2^ | rs1140239 | 146.26 |
| cg24235633 | 16 | 29,875,177 | ncRNA_exonic | *CDIPTOSP* | BIOS | 0.08 | 9 | 0.95 (0.93 ± 0.98) | 1.48 × 10^-4^ | 2.01 × 10^-2^ | rs1140239 | 146.23 |
| cg09584855 | 16 | 29,910,311 | exonic | *SEZ6L2* | FHS | 0.09 | 14 | 0.90 (0.86 ± 0.95) | 1.40 × 10^-5^ | 3.39 × 10^-3^ | rs1140239 | 111.09 |
| cg09584855 | 16 | 29,910,311 | exonic | *SEZ6L2* | BIOS | 0.13 | 9 | 0.96 (0.94 ± 0.98) | 3.44 × 10^-5^ | 6.82 × 10^-3^ | rs1140239 | 111.09 |
| cg02556718 | 16 | 29,910,594 | UTR5 | *SEZ6L2* | BIOS | 0.02 | 6 | 0.87 (0.81 ± 0.92) | 4.01 × 10^-6^ | 1.21 × 10^-3^ | rs1140239 | 110.81 |
| cg20401945 | 16 | 29,912,460 | exonic | *ASPHD1* | FHS | 0.07 | 20 | 0.88 (0.84 ± 0.92) | **7.68 × 10^-8^** | 4.61 × 10^-5^ | rs1140239 | 108.94 |
| cg20401945 | 16 | 29,912,460 | exonic | *ASPHD1* | BIOS | 0.13 | 31 | 0.97 (0.95 ± 0.99) | 4.53 × 10^-4^ | 4.36 × 10^-2^ | rs1140239 | 108.94 |
| cg10093648 | 16 | 29,912,700 | exonic | *ASPHD1* | FHS | 0.05 | 31 | 0.88 (0.84 ± 0.92) | **6.35 × 10^-8^** | 3.97 × 10^-5^ | rs1140239 | 108.70 |
| cg10093648 | 16 | 29,912,700 | exonic | *ASPHD1* | BIOS | 0.08 | 21 | 0.96 (0.93 ± 0.98) | 2.22 × 10^-4^ | 2.67 × 10^-2^ | rs1140239 | 108.70 |
| cg08515989 | 16 | 29,912,904 | exonic | *ASPHD1* | FHS | 0.11 | 30 | 0.91 (0.88 ± 0.94) | **1.72 × 10^-7^** | 8.86 × 10^-5^ | rs1140239 | 108.50 |
| cg08515989 | 16 | 29,912,904 | exonic | *ASPHD1* | BIOS | 0.17 | 30 | 0.97 (0.96 ± 0.99) | 5.10 × 10^-4^ | 4.72 × 10^-2^ | rs1140239 | 108.50 |
| cg05192831 | 16 | 29,913,007 | exonic | *ASPHD1* | BIOS | 0.04 | 10 | 0.93 (0.90 ± 0.97) | 4.19 × 10^-4^ | 4.14 × 10^-2^ | rs1140239 | 108.40 |
| cg02488299 | 16 | 29,913,223 | exonic | *ASPHD1* | FHS | 0.02 | 28 | 0.88 (0.83 ± 0.94) | 1.50 × 10^-4^ | 2.02 × 10^-2^ | rs1140239 | 108.18 |
| cg02488299 | 16 | 29,913,223 | exonic | *ASPHD1* | BIOS | 0.04 | 13 | 0.93 (0.89 ± 0.96) | 2.00 × 10^-4^ | 2.47 × 10^-2^ | rs1140239 | 108.18 |
| cg02747254 | 16 | 29,938,183 | upstream | *KCTD13* | BIOS | 0.05 | 12 | 1.10 (1.06 ± 1.14) | 1.47 × 10^-6^ | 5.40 × 10^-4^ | rs1140239 | 83.22 |
| cg02747254 | 16 | 29,938,183 | UTR5 | *TRIM40* | FHS | 0.05 | 3 | 1.18 (1.10 ± 1.26) | 1.62 × 10^-6^ | 5.81 × 10^-4^ | rs1140239 | 83.22 |
| cg16568360 | 16 | 29,987,261 | intronic | *STAG3* | FHS | 0.02 | 34 | 1.22 (1.13 ± 1.31) | **1.40 × 10^-7^** | 7.61 × 10^-5^ | rs1140239 | 34.14 |
| cg16568360 | 16 | 29,987,261 | intronic | *TAOK2* | BIOS | 0.01 | 9 | 1.17 (1.10 ± 1.25) | 6.87 × 10^-7^ | 2.87 × 10^-4^ | rs1140239 | 34.14 |
| cg06295687 | 16 | 30,006,300 | intronic | *HIRIP3* | BIOS | 0.02 | 13 | 0.89 (0.85 ± 0.93) | 3.54 × 10^-7^ | 1.62 × 10^-4^ | rs1140239 | 15.10 |
| cg04436971 | 16 | 30,016,811 | UTR3 | *INO80E* | BIOS | 0.11 | 18 | 0.93 (0.91 ± 0.95) | **5.61 × 10^-10^** | 7.18 × 10^-7^ | rs1140239 | 4.59 |
| cg04436971 | 16 | 30,016,811 | UTR3 | *INO80E* | FHS | 0.06 | 10 | 0.86 (0.81 ± 0.90) | **1.23 × 10^-8^** | 9.69 × 10^-6^ | rs1140239 | 4.59 |
| cg09245872 | 16 | 30,016,874 | UTR3 | *DOC2A, INO80E* | FHS | 0.06 | 15 | 0.87 (0.83 ± 0.92) | **1.85 × 10^-7^** | 9.27 × 10^-5^ | rs1140239 | 4.53 |
| cg09245872 | 16 | 30,016,874 | UTR3 | *DOC2A, INO80E* | BIOS | 0.11 | 38 | 0.96 (0.94 ± 0.97) | 6.66 × 10^-7^ | 2.79 × 10^-4^ | rs1140239 | 4.53 |
| cg01283141 | 16 | 30,020,506 | intronic | *DOC2A* | BIOS | 0.02 | 5 | 1.24 (1.16 ± 1.33) | **4.07 × 10^-11^** | 7.05 × 10^-8^ | rs1140239 | 0.90 |
| cg04502620 | 16 | 30,020,843 | exonic | *DOC2A* | FHS | 0.18 | 17 | 1.12 (1.08 ± 1.15) | **2.65 × 10^-12^** | 6.99 × 10^-9^ | rs1140239 | 0.56 |
| cg04502620 | 16 | 30,020,843 | exonic | *DOC2A* | BIOS | 0.37 | 13 | 1.05 (1.03 ± 1.06) | **1.19 × 10^-11^** | 2.57 × 10^-8^ | rs1140239 | 0.56 |
| cg06015834 | 16 | 30,021,696 | intronic | *DOC2A* | FHS | 0.11 | 23 | 1.15 (1.11 ± 1.19) | **1.04 × 10^-12^** | 3.20 × 10^-9^ | rs1140239 | -0.29 |
| cg06015834 | 16 | 30,021,696 | intronic | *DOC2A* | BIOS | 0.19 | 7 | 1.06 (1.05 ± 1.08) | **1.62 × 10^-11^** | 3.25 × 10^-8^ | rs1140239 | -0.29 |
| cg27151362 | 16 | 30,023,515 | intronic | *DOC2A* | FHS | 0.07 | 28 | 1.21 (1.15 ± 1.28) | **7.14 × 10^-13^** | 2.33 × 10^-9^ | rs1140239 | -2.11 |
| cg27151362 | 16 | 30,023,515 | intronic | *DOC2A* | BIOS | 0.21 | 11 | 1.05 (1.04 ± 1.07) | **1.41 × 10^-9^** | 1.51 × 10^-6^ | rs1140239 | -2.11 |
| cg03890691 | 16 | 30,023,615 | UTR5 | *DOC2A* | FHS | 0.02 | 11 | 1.42 (1.28 ± 1.57) | **5.71 × 10^-11^** | 9.50 × 10^-8^ | rs1140239 | -2.21 |
| cg03890691 | 16 | 30,023,615 | UTR5 | *DOC2A* | BIOS | 0.11 | 12 | 1.07 (1.05 ± 1.10) | **1.39 × 10^-9^** | 1.50 × 10^-6^ | rs1140239 | -2.21 |
| cg07041748 | 16 | 30,023,780 | intronic | *DOC2A* | FHS | 0.06 | 17 | 1.23 (1.16 ± 1.31) | **1.50 × 10^-11^** | 3.12 × 10^-8^ | rs1140239 | -2.38 |
| cg07041748 | 16 | 30,023,780 | intronic | *DOC2A* | BIOS | 0.17 | 16 | 1.06 (1.04 ± 1.08) | **1.05 × 10^-9^** | 1.20 × 10^-6^ | rs1140239 | -2.38 |
| cg01795660 | 16 | 30,024,074 | intronic | *DOC2A* | BIOS | 0.01 | 4 | 1.27 (1.18 ± 1.37) | **8.66 × 10^-10^** | 1.02 × 10^-6^ | rs1140239 | -2.67 |
| cg08020395 | 16 | 30,034,338 | upstream | *C16orf92* | FHS | 0.01 | 8 | 1.45 (1.29 ± 1.62) | **2.29 × 10^-10^** | 3.31 × 10^-7^ | rs1140239 | -12.94 |
| cg08020395 | 16 | 30,034,338 | upstream | *C16orf92* | BIOS | 0.04 | 4 | 1.14 (1.10 ± 1.19) | **2.89 × 10^-10^** | 4.07 × 10^-7^ | rs1140239 | -12.94 |
| cg05034471 | 16 | 30,034,801 | intronic | *C16orf92* | BIOS | 0.05 | 5 | 1.13 (1.09 ± 1.17) | **1.95 × 10^-10^** | 2.87 × 10^-7^ | rs1140239 | -13.40 |
| cg05034471 | 16 | 30,034,801 | intronic | *C16orf92* | FHS | 0.01 | 23 | 1.37 (1.24 ± 1.50) | **3.68 × 10^-10^** | 5.00 × 10^-7^ | rs1140239 | -13.40 |
| cg06985993 | 16 | 30,040,055 | intronic | *TLCD3B* | FHS | 0.14 | 11 | 1.11 (1.08 ± 1.15) | **1.17 × 10^-9^** | 1.30 × 10^-6^ | rs1140239 | -18.65 |
| cg06985993 | 16 | 30,040,055 | intronic | *TLCD3B* | BIOS | 0.32 | 10 | 1.04 (1.03 ± 1.06) | **1.30 × 10^-9^** | 1.43 × 10^-6^ | rs1140239 | -18.65 |
| cg13271951 | 16 | 30,041,760 | exonic | *TLCD3B* | FHS | 0.03 | 10 | 1.33 (1.22 ± 1.46) | **3.55 × 10^-10^** | 4.92 × 10^-7^ | rs1140239 | -20.36 |
| cg13271951 | 16 | 30,041,760 | exonic | *TLCD3B* | BIOS | 0.13 | 5 | 1.06 (1.04 ± 1.08) | **5.75 × 10^-8^** | 3.65 × 10^-5^ | rs1140239 | -20.36 |
| cg09308026 | 16 | 30,041,884 | UTR5 | *TLCD3B* | BIOS | 0.04 | 7 | 1.10 (1.06 ± 1.14) | 6.99 × 10^-7^ | 2.89 × 10^-4^ | rs1140239 | -20.48 |
| cg02507810 | 16 | 30,042,330 | UTR5 | *TLCD3B* | BIOS | 0.02 | 9 | 0.85 (0.81 ± 0.90) | **8.06 × 10^-10^** | 9.57 × 10^-7^ | rs1140239 | -20.93 |
| cg04963082 | 16 | 30,043,105 | intronic | *TLCD3B* | BIOS | 0.01 | 3 | 1.33 (1.21 ± 1.47) | **6.04 × 10^-9^** | 5.26 × 10^-6^ | rs1140239 | -21.70 |
| cg02190038 | 16 | 30,043,250 | intronic | *TLCD3B* | FHS | 0.09 | 12 | 1.13 (1.08 ± 1.18) | **3.33 × 10^-8^** | 2.36 × 10^-5^ | rs1140239 | -21.85 |
| cg02190038 | 16 | 30,043,250 | upstream | *ADAT3, SCAMP4* | BIOS | 0.19 | 15 | 1.04 (1.02 ± 1.05) | 2.96 × 10^-6^ | 9.45 × 10^-4^ | rs1140239 | -21.85 |
| cg21742836 | 16 | 30,086,175 | intergenic | *ALDOA, PPP4C* | FHS | 0.02 | 7 | 0.75 (0.67 ± 0.85) | 6.13 × 10^-6^ | 1.71 × 10^-3^ | rs1140239 | -64.77 |
| cg07249224 | 16 | 30,101,680 | intronic | *TBX6* | FHS | 0.01 | 10 | 1.24 (1.10 ± 1.39) | 4.97 × 10^-4^ | 4.64 × 10^-2^ | rs1140239 | -80.28 |
| cg05676562 | 16 | 30,102,457 | exonic | *TBX6* | FHS | 0.06 | 54 | 1.11 (1.07 ± 1.16) | 6.15 × 10^-7^ | 2.59 × 10^-4^ | rs1140239 | -81.06 |
| cg05676562 | 16 | 30,102,457 | exonic | *TBX6* | BIOS | 0.18 | 19 | 1.03 (1.01 ± 1.05) | 2.19 × 10^-4^ | 2.63 × 10^-2^ | rs1140239 | -81.06 |
| cg05806717 | 16 | 30,102,542 | UTR5 | *TBX6* | FHS | 0.05 | 40 | 1.13 (1.08 ± 1.19) | 3.29 × 10^-7^ | 1.53 × 10^-4^ | rs1140239 | -81.14 |
| cg05806717 | 16 | 30,102,542 | intronic | *NCK2* | BIOS | 0.09 | 18 | 1.06 (1.03 ± 1.08) | 1.92 × 10^-5^ | 4.34 × 10^-3^ | rs1140239 | -81.14 |
| cg00123512 | 16 | 30,102,919 | intronic | *TBX6* | BIOS | 0.02 | 17 | 1.11 (1.06 ± 1.16) | 5.02 × 10^-6^ | 1.45 × 10^-3^ | rs1140239 | -81.52 |
| cg26709300 | 16 | 30,106,682 | UTR3 | *NDUFS2* | FHS | 0.09 | 19 | 0.86 (0.82 ± 0.90) | **2.51 × 10^-9^** | 2.48 × 10^-6^ | rs1140239 | -85.28 |
| cg26709300 | 16 | 30,106,682 | exonic | *YPEL3* | BIOS | 0.18 | 10 | 0.95 (0.93 ± 0.97) | **1.55 × 10^-8^** | 1.20 × 10^-5^ | rs1140239 | -85.28 |
| cg16348385 | 16 | 30,106,822 | UTR5 | *YPEL3* | FHS | 0.03 | 12 | 0.76 (0.70 ± 0.82) | **2.01 × 10^-11^** | 3.80 × 10^-8^ | rs1140239 | -85.42 |
| cg16348385 | 16 | 30,106,822 | UTR5 | *YPEL3* | BIOS | 0.07 | 14 | 0.92 (0.89 ± 0.95) | **9.86 × 10^-8^** | 5.77 × 10^-5^ | rs1140239 | -85.42 |
| cg27106909 | 16 | 30,106,897 | UTR5 | *YPEL3* | FHS | 0.07 | 17 | 0.84 (0.80 ± 0.89) | **3.40 × 10^-10^** | 4.75 × 10^-7^ | rs1140239 | -85.50 |
| cg27106909 | 16 | 30,106,897 | UTR5 | *YPEL3* | BIOS | 0.13 | 11 | 0.94 (0.92 ± 0.96) | **2.27 × 10^-8^** | 1.71 × 10^-5^ | rs1140239 | -85.50 |
| cg12400121 | 16 | 30,108,092 | ncRNA_exonic | *LOC101928595* | BIOS | 0.02 | 4 | 0.86 (0.81 ± 0.90) | **4.17 × 10^-9^** | 3.83 × 10^-6^ | rs1140239 | -86.69 |
| cg09331206 | 16 | 30,108,321 | ncRNA_exonic | *LOC101928595* | BIOS | 0.04 | 5 | 1.15 (1.10 ± 1.20) | **1.33 × 10^-9^** | 1.46 × 10^-6^ | rs1140239 | -86.92 |
| cg27087781 | 16 | 30,108,404 | ncRNA_exonic | *LOC101928595* | FHS | 0.03 | 9 | 0.78 (0.72 ± 0.85) | **1.55 × 10^-9^** | 1.65 × 10^-6^ | rs1140239 | -87.00 |
| cg27087781 | 16 | 30,108,404 | ncRNA_exonic | *LOC101928595* | BIOS | 0.09 | 11 | 0.92 (0.90 ± 0.95) | **7.12 × 10^-9^** | 6.04 × 10^-6^ | rs1140239 | -87.00 |
| cg02335376 | 16 | 30,124,880 | upstream, downstream | *GDPD3, MAPK3* | BIOS | 0.03 | 10 | 0.90 (0.86 ± 0.94) | 6.34 × 10^-7^ | 2.66 × 10^-4^ | rs1140239 | -103.48 |
| cg00192773 | 16 | 30,131,641 | intronic | *MAPK3* | BIOS | 0.03 | 9 | 0.85 (0.78 ± 0.93) | 4.84 × 10^-4^ | 4.56 × 10^-2^ | rs1140239 | -110.24 |
| cg08966293 | 16 | 30,134,858 | upstream | *MAPK3* | BIOS | 0.01 | 10 | 1.23 (1.13 ± 1.32) | **2.60 × 10^-7^** | 1.25 × 10^-4^ | rs1140239 | -113.46 |
| cg08464513 | 16 | 30,136,024 | intergenic | *MAPK3, CORO1A* | FHS | 0.07 | 19 | 0.88 (0.84 ± 0.93) | 4.68 × 10^-7^ | 2.05 × 10^-4^ | rs1140239 | -114.62 |
| cg08464513 | 16 | 30,136,024 | intergenic | *MAPK3, CORO1A* | BIOS | 0.07 | 14 | 0.93 (0.90 ± 0.96) | 2.28 × 10^-6^ | 7.61 × 10^-4^ | rs1140239 | -114.62 |
| cg05731156 | 16 | 30,418,364 | upstream | *ZNF771* | FHS | 0.01 | 13 | 0.76 (0.67 ± 0.85) | 1.96 × 10^-6^ | 6.79 × 10^-4^ | rs1140239 | -396.96 |
| cg01402814 | 16 | 30,418,374 | upstream | *ZNF771* | FHS | 0.02 | 22 | 0.83 (0.77 ± 0.90) | 1.17 × 10^-5^ | 2.90 × 10^-3^ | rs1140239 | -396.97 |
| cg06323624 | 16 | 30,642,532 | intergenic | *ZNF689, PRR14* | FHS | 0.05 | 70 | 1.10 (1.05 ± 1.15) | 1.02 × 10^-4^ | 1.52 × 10^-2^ | rs59735493 | 490.57 |
| cg01792666 | 16 | 30,710,912 | intronic | *SRCAP* | FHS | 0.03 | 13 | 1.26 (1.15 ± 1.39) | 1.83 × 10^-6^ | 6.47 × 10^-4^ | rs59735493 | 422.19 |
| cg08246316 | 16 | 30,751,261 | UTR3 | *SRCAP* | BIOS | 0.12 | 8 | 0.95 (0.92 ± 0.98) | 3.03 × 10^-4^ | 3.30 × 10^-2^ | rs59735493 | 381.84 |
| cg08246316 | 16 | 30,751,261 | UTR3 | *SRCAP* | FHS | 0.07 | 12 | 0.88 (0.82 ± 0.94) | 3.67 × 10^-4^ | 3.75 × 10^-2^ | rs59735493 | 381.84 |
| cg15852700 | 16 | 30,772,531 | exonic | *CCDC189* | FHS | 0.02 | 9 | 1.43 (1.20 ± 1.70) | 4.68 × 10^-5^ | 8.57 × 10^-3^ | rs59735493 | 360.57 |
| cg09248197 | 16 | 30,799,283 | upstream | *ZNF629* | BIOS | 0.01 | 2 | 0.86 (0.79 ± 0.93) | 1.42 × 10^-4^ | 1.95 × 10^-2^ | rs59735493 | 333.82 |
| cg04477789 | 16 | 30,935,822 | intronic | *FBXL19* | BIOS | 0.01 | 3 | 1.19 (1.12 ± 1.27) | **2.04 × 10^-7^** | 1.02 × 10^-4^ | rs59735493 | 197.28 |
| cg10421029 | 16 | 30,936,028 | UTR5 | *FBXL19* | FHS | 0.15 | 6 | 1.09 (1.06 ± 1.13) | **2.74 × 10^-7^** | 1.32 × 10^-4^ | rs59735493 | 197.07 |
| cg10421029 | 16 | 30,936,028 | UTR5 | *FBXL19* | BIOS | 0.20 | 5 | 1.04 (1.02 ± 1.06) | 6.10 × 10^-7^ | 2.57 × 10^-4^ | rs59735493 | 197.07 |
| cg10238917 | 16 | 30,942,621 | intronic | *FBXL19* | FHS | 0.02 | 16 | 0.82 (0.75 ± 0.91) | 1.15 × 10^-4^ | 1.66 × 10^-2^ | rs59735493 | 190.48 |
| cg10238917 | 16 | 30,942,621 | intronic | *FBXL19* | BIOS | 0.02 | 5 | 0.89 (0.83 ± 0.95) | 4.57 × 10^-4^ | 4.38 × 10^-2^ | rs59735493 | 190.48 |
| cg06403422 | 16 | 30,970,886 | intronic | *SETD1A* | FHS | 0.01 | 8 | 0.77 (0.68 ± 0.87) | 2.80 × 10^-5^ | 5.80 × 10^-3^ | rs59735493 | 162.21 |
| cg06403422 | 16 | 30,970,886 | intronic | *SETD1A* | BIOS | 0.01 | 4 | 0.85 (0.79 ± 0.92) | 6.42 × 10^-5^ | 1.08 × 10^-2^ | rs59735493 | 162.21 |
| cg09162137 | 16 | 30,995,627 | UTR5 | *USP8* | FHS | 0.06 | 28 | 1.12 (1.06 ± 1.18) | 2.75 × 10^-5^ | 5.72 × 10^-3^ | rs59735493 | 137.47 |
| cg09162137 | 16 | 30,995,627 | UTR3 | *SETD1A* | BIOS | 0.10 | 10 | 1.05 (1.02 ± 1.07) | 1.19 × 10^-4^ | 1.70 × 10^-2^ | rs59735493 | 137.47 |
| cg07359991 | 16 | 30,995,944 | UTR3 | *SETD1A* | FHS | 0.03 | 17 | 1.19 (1.10 ± 1.29) | 9.11 × 10^-6^ | 2.35 × 10^-3^ | rs59735493 | 137.16 |
| cg07359991 | 16 | 30,995,944 | UTR3 | *SETD1A* | BIOS | 0.04 | 8 | 1.08 (1.04 ± 1.12) | 1.53 × 10^-5^ | 3.64 × 10^-3^ | rs59735493 | 137.16 |
| cg26949037 | 16 | 31,003,936 | UTR3 | *STX1B* | BIOS | 0.06 | 10 | 0.92 (0.89 ± 0.95) | 4.45 × 10^-6^ | 1.31 × 10^-3^ | rs59735493 | 129.16 |
| cg26949037 | 16 | 31,003,936 | UTR3 | *STX1B* | FHS | 0.06 | 46 | 0.90 (0.85 ± 0.94) | 2.98 × 10^-5^ | 6.06 × 10^-3^ | rs59735493 | 129.16 |
| cg05418781 | 16 | 31,004,853 | intronic | *GGA2* | BIOS | 0.02 | 5 | 0.79 (0.71 ± 0.88) | 1.95 × 10^-5^ | 4.39 × 10^-3^ | rs59735493 | 128.25 |
| cg05418781 | 16 | 31,004,853 | intronic | *STX1B* | FHS | 0.01 | 8 | 0.79 (0.70 ± 0.90) | 2.16 × 10^-4^ | 2.62 × 10^-2^ | rs59735493 | 128.25 |
| cg04018474 | 16 | 31,008,003 | splicing | *STX1B* | BIOS | 0.31 | 21 | 0.97 (0.95 ± 0.98) | 1.62 × 10^-5^ | 3.81 × 10^-3^ | rs59735493 | 125.10 |
| cg04018474 | 16 | 31,008,003 | exonic | *STX1B* | FHS | 0.21 | 37 | 0.95 (0.92 ± 0.97) | 2.29 × 10^-5^ | 4.94 × 10^-3^ | rs59735493 | 125.10 |
| cg01670771 | 16 | 31,009,232 | intronic | *STX1B* | BIOS | 0.04 | 6 | 0.86 (0.81 ± 0.92) | 5.42 × 10^-6^ | 1.55 × 10^-3^ | rs59735493 | 123.87 |
| cg01670771 | 16 | 31,009,232 | intronic | *STX1B* | FHS | 0.02 | 13 | 0.82 (0.75 ± 0.89) | 5.64 × 10^-6^ | 1.61 × 10^-3^ | rs59735493 | 123.87 |
| cg00249205 | 16 | 31,012,263 | splicing | *STX1B* | BIOS | 0.23 | 29 | 0.97 (0.95 ± 0.98) | 2.29 × 10^-5^ | 4.94 × 10^-3^ | rs59735493 | 120.84 |
| cg00249205 | 16 | 31,012,263 | intergenic | *THSD7A, TMEM106B* | FHS | 0.17 | 48 | 0.94 (0.91 ± 0.96) | 2.85 × 10^-5^ | 5.85 × 10^-3^ | rs59735493 | 120.84 |
| cg09499421 | 16 | 31,020,735 | intronic | *STX1B* | BIOS | 0.05 | 4 | 0.91 (0.87 ± 0.95) | 1.75 × 10^-6^ | 6.24 × 10^-4^ | rs59735493 | 112.37 |
| cg09499421 | 16 | 31,020,735 | intronic | *STX1B* | FHS | 0.03 | 13 | 0.81 (0.74 ± 0.89) | 5.34 × 10^-6^ | 1.53 × 10^-3^ | rs59735493 | 112.37 |
| cg03144232 | 16 | 31,022,351 | intronic | *ATP8B3* | BIOS | 0.05 | 8 | 1.12 (1.07 ± 1.18) | 1.38 × 10^-6^ | 5.08 × 10^-4^ | rs59735493 | 110.75 |
| cg03144232 | 16 | 31,022,351 | upstream | *STX1B* | FHS | 0.04 | 23 | 1.18 (1.08 ± 1.29) | 4.21 × 10^-4^ | 4.15 × 10^-2^ | rs59735493 | 110.75 |
| cg25033993 | 16 | 31,022,372 | intergenic | *FAM171A2, ITGA2B* | FHS | 0.05 | 13 | 1.23 (1.13 ± 1.34) | 2.29 × 10^-6^ | 7.62 × 10^-4^ | rs59735493 | 110.73 |
| cg25033993 | 16 | 31,022,372 | upstream | *STX1B* | BIOS | 0.08 | 23 | 1.06 (1.02 ± 1.09) | 4.96 × 10^-4^ | 4.64 × 10^-2^ | rs59735493 | 110.73 |
| cg05787209 | 16 | 31,022,521 | intergenic | *ZBED9, LINC00533* | FHS | 0.03 | 9 | 1.59 (1.32 ± 1.92) | 1.04 × 10^-6^ | 4.03 × 10^-4^ | rs59735493 | 110.58 |
| cg05787209 | 16 | 31,022,521 | upstream | *STX1B* | BIOS | 0.05 | 13 | 1.09 (1.04 ± 1.13) | 5.31 × 10^-5^ | 9.37 × 10^-3^ | rs59735493 | 110.58 |
| cg06233904 | 16 | 31,044,135 | upstream | *STX4* | BIOS | 0.05 | 3 | 1.1 (1.060 ± 1.14) | 4.86 × 10^-6^ | 1.41 × 10^-3^ | rs59735493 | 88.97 |
| cg06233904 | 16 | 31,044,135 | upstream | *STX4* | FHS | 0.05 | 13 | 1.24 (1.13 ± 1.37) | 7.58 × 10^-6^ | 2.04 × 10^-3^ | rs59735493 | 88.97 |
| cg08893833 | 16 | 31,045,809 | exonic | *STX4* | BIOS | 0.04 | 7 | 1.10 (1.05 ± 1.14) | 5.58 × 10^-6^ | 1.59 × 10^-3^ | rs59735493 | 87.29 |
| cg07404961 | 16 | 31,049,270 | exonic | *STX4* | FHS | 0.07 | 14 | 0.85 (0.79 ± 0.91) | 1.83 × 10^-6^ | 6.47 × 10^-4^ | rs59735493 | 83.83 |
| cg07404961 | 16 | 31,049,270 | exonic | *STX4* | BIOS | 0.10 | 6 | 0.94 (0.92 ± 0.97) | 3.71 × 10^-6^ | 1.14 × 10^-3^ | rs59735493 | 83.83 |
| cg11824827 | 16 | 31,075,547 | intronic | *DYDC2* | BIOS | 0.01 | 3 | 1.28 (1.18 ± 1.39) | **1.65 × 10^-9^** | 1.71 × 10^-6^ | rs59735493 | 57.55 |
| cg11824827 | 16 | 31,075,547 | intronic | *PCGF3* | FHS | 0.05 | 25 | 1.12 (1.06 ± 1.18) | 2.70 × 10^-5^ | 5.67 × 10^-3^ | rs59735493 | 57.55 |
| cg10380221 | 16 | 31,075,618 | exonic | *ZNF668* | BIOS | 0.02 | 9 | 1.16 (1.10 ± 1.22) | **7.10 × 10^-8^** | 4.31 × 10^-5^ | rs59735493 | 57.48 |
| cg10045909 | 16 | 31,075,842 | intronic | *ZNF668* | BIOS | 0.01 | 5 | 1.18 (1.10 ± 1.27) | 9.54 × 10^-6^ | 2.44 × 10^-3^ | rs59735493 | 57.26 |
| cg03134962 | 16 | 31,083,405 | intronic | *ZNF668* | FHS | 0.24 | 20 | 0.94 (0.92 ± 0.96) | 1.97 × 10^-6^ | 6.80 × 10^-4^ | rs59735493 | 49.70 |
| cg03134962 | 16 | 31,083,405 | intronic | *ZNF668* | BIOS | 0.35 | 5 | 0.97 (0.96 ± 0.98) | 4.10 × 10^-6^ | 1.23 × 10^-3^ | rs59735493 | 49.70 |
| cg01067137 | 16 | 31,090,322 | exonic | *ZNF646* | FHS | 0.23 | 13 | 1.08 (1.04 ± 1.13) | 4.41 × 10^-5^ | 8.21 × 10^-3^ | rs59735493 | 42.78 |
| cg09552399 | 16 | 31,091,326 | exonic | *ZNF646* | BIOS | 0.01 | 6 | 0.83 (0.78 ± 0.89) | **9.36 × 10^-8^** | 5.52 × 10^-5^ | rs59735493 | 41.77 |
| cg09925874 | 16 | 31,091,428 | exonic | *ZNF646* | BIOS | 0.03 | 11 | 0.89 (0.85 ± 0.93) | **1.53 × 10^-7^** | 8.08 × 10^-5^ | rs59735493 | 41.67 |
| cg09925874 | 16 | 31,091,428 | ncRNA_intronic | *TSBP1-AS1* | FHS | 0.02 | 14 | 0.72 (0.62 ± 0.84) | 1.91 × 10^-5^ | 4.33 × 10^-3^ | rs59735493 | 41.67 |
| cg09172973 | 16 | 31,091,608 | exonic | *ZNF646* | BIOS | 0.01 | 18 | 0.88 (0.83 ± 0.94) | 9.22 × 10^-5^ | 1.41 × 10^-2^ | rs59735493 | 41.49 |
| cg08374890 | 16 | 31,117,067 | intergenic | *VKORC1, BCKDK* | BIOS | 0.07 | 11 | 1.07 (1.04 ± 1.10) | 1.01 × 10^-6^ | 3.93 × 10^-4^ | rs59735493 | 16.03 |
| cg08374890 | 16 | 31,117,067 | intergenic | *VKORC1, BCKDK* | FHS | 0.06 | 30 | 1.13 (1.07 ± 1.19) | 2.08 × 10^-6^ | 7.10 × 10^-4^ | rs59735493 | 16.03 |
| cg03984055 | 16 | 31,117,318 | intergenic | *VKORC1, BCKDK* | BIOS | 0.03 | 5 | 1.12 (1.07 ± 1.18) | 1.29 × 10^-6^ | 4.88 × 10^-4^ | rs59735493 | 15.78 |
| cg03984055 | 16 | 31,117,318 | intergenic | *VKORC1, BCKDK* | FHS | 0.01 | 5 | 1.37 (1.20 ± 1.57) | 2.88 × 10^-6^ | 9.23 × 10^-4^ | rs59735493 | 15.78 |
| cg03418659 | 16 | 31,128,414 | upstream | *KAT8* | BIOS | 0.27 | 4 | 1.04 (1.02 ± 1.05) | 1.06 × 10^-6^ | 4.09 × 10^-4^ | rs59735493 | 4.69 |
| cg03418659 | 16 | 31,128,414 | upstream | *KAT8* | FHS | 0.24 | 30 | 1.06 (1.03 ± 1.09) | 4.73 × 10^-6^ | 1.38 × 10^-3^ | rs59735493 | 4.69 |
| cg00524708 | 16 | 31,159,558 | intronic | *PRSS36* | FHS | 0.03 | 10 | 1.25 (1.15 ± 1.37) | **2.38 × 10^-7^** | 1.16 × 10^-4^ | rs59735493 | -26.46 |
| cg00524708 | 16 | 31,159,558 | intronic | *GPX6* | BIOS | 0.08 | 11 | 1.06 (1.03 ± 1.08) | 8.02 × 10^-5^ | 1.29 × 10^-2^ | rs59735493 | -26.46 |
| cg26675395 | 16 | 31,159,623 | intronic | *PRSS36* | BIOS | 0.08 | 13 | 1.06 (1.03 ± 1.09) | 3.49 × 10^-5^ | 6.88 × 10^-3^ | rs59735493 | -26.52 |
| cg14301190 | 16 | 31,159,704 | intronic | *NCK2* | BIOS | 0.08 | 10 | 1.06 (1.03 ± 1.09) | 2.57 × 10^-5^ | 5.45 × 10^-3^ | rs59735493 | -26.60 |
| cg14301190 | 16 | 31,159,704 | intronic | *PRSS36* | FHS | 0.03 | 52 | 1.10 (1.04 ± 1.16) | 4.72 × 10^-4^ | 4.48 × 10^-2^ | rs59735493 | -26.60 |
| cg04149015 | 16 | 31,159,854 | exonic | *PRSS36* | FHS | 0.01 | 30 | 1.25 (1.15 ± 1.37) | 5.77 × 10^-7^ | 2.45 × 10^-4^ | rs59735493 | -26.75 |
| cg04149015 | 16 | 31,159,854 | exonic | *PRSS36* | BIOS | 0.06 | 9 | 1.07 (1.04 ± 1.11) | 1.48 × 10^-5^ | 3.55 × 10^-3^ | rs59735493 | -26.75 |
| cg01390564 | 16 | 31,162,282 | upstream | *PRSS36* | BIOS | 0.10 | 29 | 1.05 (1.02 ± 1.08) | 1.60 × 10^-4^ | 2.11 × 10^-2^ | rs59735493 | -29.18 |
| cg09425279 | 16 | 31,188,401 | intergenic | *PRSS36, FUS* | BIOS | 0.04 | 4 | 0.93 (0.89 ± 0.96) | 1.46 × 10^-4^ | 1.99 × 10^-2^ | rs59735493 | -55.30 |
| cg08081390 | 16 | 31,195,218 | exonic | *FUS* | BIOS | 0.04 | 5 | 0.91 (0.87 ± 0.95) | 2.23 × 10^-5^ | 4.84 × 10^-3^ | rs59735493 | -62.12 |
| cg08081390 | 16 | 31,195,218 | exonic | *FUS* | FHS | 0.02 | 9 | 0.78 (0.68 ± 0.89) | 1.63 × 10^-4^ | 2.13 × 10^-2^ | rs59735493 | -62.12 |
| cg12100791 | 16 | 31,214,417 | ncRNA_exonic | *PYCARD-AS1* | BIOS | 0.02 | 13 | 1.11 (1.06 ± 1.17) | 2.57 × 10^-5^ | 5.45 × 10^-3^ | rs59735493 | -81.32 |
| cg12100791 | 16 | 31,214,417 | ncRNA_exonic | *PYCARD-AS1* | FHS | 0.02 | 22 | 1.18 (1.09 ± 1.28) | 7.76 × 10^-5^ | 1.26 × 10^-2^ | rs59735493 | -81.32 |
| cg09115984 | 16 | 31,214,426 | ncRNA_exonic | *PYCARD-AS1* | FHS | 0.01 | 24 | 1.35 (1.21 ± 1.50) | **1.19 × 10^-7^** | 6.80 × 10^-5^ | rs59735493 | -81.33 |
| cg09115984 | 16 | 31,214,426 | ncRNA_exonic | *PYCARD-AS1* | BIOS | 0.02 | 7 | 1.13 (1.08 ± 1.20) | 3.05 × 10^-6^ | 9.66 × 10^-4^ | rs59735493 | -81.33 |
| cg02900356 | 16 | 31,214,494 | ncRNA_exonic | *PYCARD-AS1* | BIOS | 0.03 | 9 | 1.10 (1.06 ± 1.15) | 3.75 × 10^-6^ | 1.15 × 10^-3^ | rs59735493 | -81.39 |
| cg07461837 | 16 | 31,214,880 | upstream, downstream | *PYCARD, PYCARD-AS1* | FHS | 0.25 | 26 | 0.95 (0.93 ± 0.98) | 1.99 × 10^-4^ | 2.47 × 10^-2^ | rs59735493 | -81.78 |
| cg00327577 | 16 | 31,214,993 | upstream, downstream | *PYCARD, PYCARD-AS1* | BIOS | 0.01 | 5 | 1.17 (1.09 ± 1.25) | 8.16 × 10^-6^ | 2.17 × 10^-3^ | rs59735493 | -81.89 |
| cg09921810 | 16 | 31,227,497 | intronic | *PYDC1, TRIM72* | BIOS | 0.03 | 7 | 1.13 (1.07 ± 1.18) | 4.52 × 10^-6^ | 1.33 × 10^-3^ | rs59735493 | -94.40 |
| cg09921810 | 16 | 31,227,497 | exonic | *RING1* | FHS | 0.02 | 15 | 1.29 (1.15 ± 1.44) | 9.84 × 10^-6^ | 2.50 × 10^-3^ | rs59735493 | -94.40 |
| cg18682103 | 16 | 31,227,515 | intronic | *PYDC1, TRIM72* | FHS | 0.03 | 15 | 1.29 (1.16 ± 1.44) | 5.19 × 10^-6^ | 1.50 × 10^-3^ | rs59735493 | -94.42 |
| cg18682103 | 16 | 31,227,515 | intronic | *PYDC1, TRIM72* | BIOS | 0.03 | 8 | 1.12 (1.06 ± 1.18) | 3.31 × 10^-5^ | 6.58 × 10^-3^ | rs59735493 | -94.42 |
| cg10038009 | 16 | 31,228,202 | exonic | *PYDC1* | BIOS | 0.01 | 15 | 1.13 (1.06 ± 1.22) | 3.27 × 10^-4^ | 3.48 × 10^-2^ | rs59735493 | -95.10 |
| cg08346922 | 16 | 31,228,299 | exonic | *PYDC1* | BIOS | 0.02 | 5 | 1.14 (1.06 ± 1.21) | 1.66 × 10^-4^ | 2.16 × 10^-2^ | rs59735493 | -95.20 |
| cg16556397 | 16 | 31,228,515 | intergenic | *GIGYF1, POP7* | FHS | 0.02 | 5 | 1.54 (1.26 ± 1.87) | 1.74 × 10^-5^ | 4.01 × 10^-3^ | rs59735493 | -95.42 |
| cg16422492 | 16 | 31,228,555 | intronic | *TRIM72* | BIOS | 0.02 | 9 | 1.13 (1.06 ± 1.20) | 1.13 × 10^-4^ | 1.64 × 10^-2^ | rs59735493 | -95.46 |
| cg03458229 | 16 | 31,286,562 | exonic | *MYO1C* | FHS | 0.01 | 18 | 0.79 (0.71 ± 0.89) | 7.31 × 10^-5^ | 1.20 × 10^-2^ | rs59735493 | -153.46 |
| cg07435237 | 16 | 31,469,562 | upstream | *ARMC5* | FHS | 0.07 | 23 | 1.09 (1.04 ± 1.14) | 1.21 × 10^-4^ | 1.72 × 10^-2^ | rs59735493 | -336.46 |
| cg01027010 | 16 | 31,576,339 | UTR5 | *SCARA5* | FHS | 0.08 | 12 | 0.91 (0.86 ± 0.95) | 4.90 × 10^-5^ | 8.85 × 10^-3^ | rs59735493 | -443.24 |
| cg02128278 | 16 | 89,764,314 | upstream | *HYDIN* | FHS | 0.06 | 13 | 0.90 (0.85 ± 0.95) | 8.73 × 10^-5^ | 1.37 × 10^-2^ | rs56407236 | 405.78 |
| cg02128278 | 16 | 89,764,314 | exonic | *SPATA2L* | BIOS | 0.10 | 29 | 0.95 (0.92 ± 0.98) | 2.60 × 10^-4^ | 2.96 × 10^-2^ | rs56407236 | 405.78 |
| cg03740752 | 16 | 89,903,046 | intronic | *SPIRE2* | FHS | 0.04 | 15 | 1.12 (1.05 ± 1.19) | 5.08 × 10^-4^ | 4.70 × 10^-2^ | rs56407236 | 267.05 |
| cg06981439 | 16 | 90,114,123 | ncRNA_intronic | *URAHP* | FHS | 0.11 | 78 | 1.07 (1.03 ± 1.11) | 9.28 × 10^-5^ | 1.42 × 10^-2^ | rs56407236 | 55.97 |
| cg00974523 | 16 | 90,124,400 | UTR3 | *PRDM7* | BIOS | 0.05 | 23 | 1.05 (1.02 ± 1.07) | 3.91 × 10^-4^ | 3.91 × 10^-2^ | rs56407236 | 45.70 |
| cg16799087 | 16 | 90,128,361 | exonic | *PRDM7* | BIOS | 0.02 | 16 | 0.93 (0.89 ± 0.97) | 5.01 × 10^-4^ | 4.66 × 10^-2^ | rs56407236 | 41.73 |
| cg05106699 | 16 | 90,128,833 | exonic | *PRDM7* | FHS | 0.01 | 13 | 0.79 (0.71 ± 0.89) | 8.59 × 10^-5^ | 1.35 × 10^-2^ | rs56407236 | 41.26 |
| cg06295223 | 16 | 90,143,751 | upstream | *TSPAN14* | BIOS | 0.25 | 52 | 1.03 (1.02 ± 1.05) | **1.84 × 10^-7^** | 9.24 × 10^-5^ | rs56407236 | 26.34 |
| cg06295223 | 16 | 90,143,751 | intergenic | *PRDM7, FAM157B* | FHS | 0.20 | 99 | 1.07 (1.04 ± 1.09) | **2.81 × 10^-7^** | 1.35 × 10^-4^ | rs56407236 | 26.34 |
| cg05270750 | 16 | 90,143,788 | intergenic | *PRDM7, FAM157B* | BIOS | 0.20 | 49 | 1.04 (1.02 ± 1.05) | **1.48 × 10^-7^** | 7.89 × 10^-5^ | rs56407236 | 26.31 |
| cg05270750 | 16 | 90,143,788 | intergenic | *PRDM7, FAM157B* | FHS | 0.18 | 98 | 1.06 (1.03 ± 1.08) | 2.85 × 10^-6^ | 9.17 × 10^-4^ | rs56407236 | 26.31 |
| cg04298253 | 16 | 90,143,815 | intergenic | *LINC00533, LINC01623* | BIOS | 0.18 | 49 | 1.03 (1.02 ± 1.05) | 1.59 × 10^-6^ | 5.72 × 10^-4^ | rs56407236 | 26.28 |
| cg04298253 | 16 | 90,143,815 | intergenic | *PRDM7, FAM157B* | FHS | 0.14 | 54 | 1.08 (1.05 ± 1.11) | 2.05 × 10^-6^ | 7.01 × 10^-4^ | rs56407236 | 26.28 |
| cg26458287 | 16 | 90,143,852 | intergenic | *PRDM7, FAM157B* | FHS | 0.11 | 63 | 1.07 (1.04 ± 1.11) | 2.82 × 10^-5^ | 5.83 × 10^-3^ | rs56407236 | 26.24 |
| cg16611967 | 16 | 90,144,006 | intergenic | *PRDM7, FAM157B* | BIOS | 0.22 | 51 | 1.03 (1.02 ± 1.05) | **3.39 × 10^-7^** | 1.57 × 10^-4^ | rs56407236 | 26.09 |
| cg16611967 | 16 | 90,144,006 | intergenic | *PRDM7, FAM157B* | FHS | 0.20 | 133 | 1.06 (1.03 ± 1.09) | 4.30 × 10^-6^ | 1.28 × 10^-3^ | rs56407236 | 26.09 |
| cg09435890 | 16 | 90,144,788 | intergenic | *PRDM7, FAM157B* | FHS | 0.25 | 49 | 1.06 (1.03 ± 1.08) | 2.20 × 10^-6^ | 7.38 × 10^-4^ | rs56407236 | 25.31 |
| cg09435890 | 16 | 90,144,788 | intergenic | *PRDM7, FAM157B* | BIOS | 0.34 | 45 | 1.02 (1.01 ± 1.03) | 3.28 × 10^-5^ | 6.53 × 10^-3^ | rs56407236 | 25.31 |
| cg13049862 | 17 | 1,384,170 | intronic | *ITGAM* | FHS | 0.04 | 24 | 0.86 (0.80 ± 0.93) | 7.31 × 10^-5^ | 1.20 × 10^-2^ | rs35048651 | 247.17 |
| cg24055855 | 17 | 1,535,293 | exonic | *KNOP1* | BIOS | 0.01 | 21 | 0.90 (0.85 ± 0.94) | 3.00 × 10^-5^ | 6.08 × 10^-3^ | rs35048651 | 96.05 |
| cg11998307 | 17 | 1,549,434 | upstream, downstream | *SCARF1, RILP* | BIOS | 0.03 | 30 | 0.94 (0.91 ± 0.97) | 2.82 × 10^-4^ | 3.12 × 10^-2^ | rs35048651 | 81.91 |
| cg02529627 | 17 | 1,618,713 | ncRNA_intronic | *MIR22HG* | BIOS | 0.03 | 13 | 0.91 (0.88 ± 0.95) | 2.20 × 10^-6^ | 7.38 × 10^-4^ | rs35048651 | 12.63 |
| cg21824343 | 17 | 1,633,714 | exonic | *WDR81* | FHS | 0.03 | 24 | 1.15 (1.07 ± 1.24) | 1.25 × 10^-4^ | 1.77 × 10^-2^ | rs35048651 | -2.37 |
| cg10426084 | 17 | 1,640,472 | intronic | *WDR81* | FHS | 0.11 | 19 | 1.12 (1.07 ± 1.16) | **1.68 × 10^-7^** | 8.73 × 10^-5^ | rs35048651 | -9.13 |
| cg10426084 | 17 | 1,640,472 | intronic | *WDR81* | BIOS | 0.14 | 11 | 1.05 (1.03 ± 1.07) | 7.49 × 10^-6^ | 2.02 × 10^-3^ | rs35048651 | -9.13 |
| cg06387669 | 17 | 1,646,196 | UTR5 | *SERPINF2* | BIOS | 0.02 | 4 | 1.10 (1.05 ± 1.16) | 2.44 × 10^-4^ | 2.84 × 10^-2^ | rs35048651 | -14.86 |
| cg10516117 | 17 | 1,656,147 | intronic | *SERPINF2* | BIOS | 0.31 | 14 | 0.97 (0.96 ± 0.98) | 2.21 × 10^-5^ | 4.82 × 10^-3^ | rs35048651 | -24.81 |
| cg10516117 | 17 | 1,656,147 | intronic | *SERPINF2* | FHS | 0.14 | 28 | 0.93 (0.90 ± 0.97) | 6.31 × 10^-5^ | 1.07 × 10^-2^ | rs35048651 | -24.81 |
| cg13027184 | 17 | 1,657,448 | exonic | *SERPINF2* | BIOS | 0.03 | 11 | 1.08 (1.04 ± 1.13) | 4.33 × 10^-4^ | 4.23 × 10^-2^ | rs35048651 | -26.11 |
| cg17514665 | 17 | 1,657,533 | exonic | *SERPINF2* | BIOS | 0.02 | 2 | 1.12 (1.06 ± 1.18) | 2.73 × 10^-5^ | 5.72 × 10^-3^ | rs35048651 | -26.19 |
| cg21071097 | 17 | 4,803,053 | UTR5 | *C17orf107* | FHS | 0.01 | 14 | 1.32 (1.14 ± 1.52) | 1.88 × 10^-4^ | 2.36 × 10^-2^ | rs7209200 | 166.89 |
| cg21071097 | 17 | 4,803,053 | UTR5 | *C17orf107* | BIOS | 0.02 | 10 | 1.08 (1.03 ± 1.12) | 3.48 × 10^-4^ | 3.65 × 10^-2^ | rs7209200 | 166.89 |
| cg19753476 | 17 | 4,803,506 | intronic | *C17orf107, CHRNE* | BIOS | 0.01 | 21 | 1.11 (1.07 ± 1.16) | 1.08 × 10^-6^ | 4.16 × 10^-4^ | rs7209200 | 166.43 |
| cg09495303 | 17 | 4,804,838 | exonic | *CHRNE* | BIOS | 0.30 | 13 | 0.98 (0.97 ± 0.98) | **1.78 × 10^-7^** | 9.05 × 10^-5^ | rs7209200 | 165.10 |
| cg09495303 | 17 | 4,804,838 | upstream | *PICALM* | FHS | 0.25 | 78 | 0.95 (0.92 ± 0.97) | 9.10 × 10^-7^ | 3.61 × 10^-4^ | rs7209200 | 165.10 |
| cg05019311 | 17 | 4,807,065 | upstream, downstream | *CHRNE, C17orf107* | BIOS | 0.03 | 17 | 0.91 (0.89 ± 0.94) | **5.29 × 10^-8^** | 3.46 × 10^-5^ | rs7209200 | 162.88 |
| cg01835620 | 17 | 4,807,758 | intergenic | *CHRNE, GP1BA* | BIOS | 0.06 | 13 | 1.06 (1.04 ± 1.09) | **3.38 × 10^-8^** | 2.38 × 10^-5^ | rs7209200 | 162.18 |
| cg01835620 | 17 | 4,807,758 | intergenic | *CHRNE, GP1BA* | FHS | 0.05 | 35 | 1.17 (1.10 ± 1.23) | **6.04 × 10^-8^** | 3.80 × 10^-5^ | rs7209200 | 162.18 |
| cg04970434 | 17 | 4,838,096 | UTR3 | *GP1BA* | FHS | 0.01 | 11 | 0.78 (0.71 ± 0.86) | 1.33 × 10^-6^ | 4.95 × 10^-4^ | rs7209200 | 131.84 |
| cg04970434 | 17 | 4,838,096 | UTR3 | *GP1BA* | BIOS | 0.03 | 7 | 0.93 (0.90 ± 0.96) | 4.09 × 10^-6^ | 1.23 × 10^-3^ | rs7209200 | 131.84 |
| cg13557167 | 17 | 4,840,493 | UTR3 | *SLC25A11* | BIOS | 0.02 | 29 | 0.93 (0.90 ± 0.96) | 2.96 × 10^-5^ | 6.02 × 10^-3^ | rs7209200 | 129.45 |
| cg04958703 | 17 | 4,844,722 | intronic | *RNF167* | FHS | 0.04 | 21 | 0.85 (0.80 ± 0.91) | 1.28 × 10^-6^ | 4.86 × 10^-4^ | rs7209200 | 125.22 |
| cg04958703 | 17 | 4,844,722 | intronic | *RNF167* | BIOS | 0.07 | 74 | 0.97 (0.95 ± 0.98) | 4.65 × 10^-5^ | 8.53 × 10^-3^ | rs7209200 | 125.22 |
| cg19427746 | 17 | 4,889,932 | intronic | *CAMTA2* | FHS | 0.63 | 80 | 1.04 (1.03 ± 1.06) | **2.01 × 10^-8^** | 1.53 × 10^-5^ | rs7209200 | 80.01 |
| cg19427746 | 17 | 4,889,932 | intronic | *CAMTA2* | BIOS | 0.68 | 61 | 1.02 (1.01 ± 1.03) | **2.50 × 10^-8^** | 1.85 × 10^-5^ | rs7209200 | 80.01 |
| cg09307264 | 17 | 4,902,281 | intronic | *KIF1C* | BIOS | 0.29 | 25 | 0.97 (0.96 ± 0.98) | **1.61 × 10^-7^** | 8.42 × 10^-5^ | rs7209200 | 67.66 |
| cg09307264 | 17 | 4,902,281 | intronic | *KIF1C* | FHS | 0.13 | 29 | 0.91 (0.88 ± 0.95) | **2.27 × 10^-7^** | 1.12 × 10^-4^ | rs7209200 | 67.66 |
| cg03877174 | 17 | 4,923,126 | ncRNA_intronic | *LOC102724009* | FHS | 0.04 | 15 | 1.21 (1.13 ± 1.28) | **3.12 × 10^-9^** | 2.96 × 10^-6^ | rs7209200 | 46.81 |
| cg03877174 | 17 | 4,923,126 | ncRNA_intronic | *LOC102724009* | BIOS | 0.05 | 4 | 1.07 (1.05 ± 1.10) | **1.31 × 10^-7^** | 7.26 × 10^-5^ | rs7209200 | 46.81 |
| cg13294032 | 17 | 4,926,946 | exonic | *KIF1C* | BIOS | 0.01 | 4 | 1.17 (1.10 ± 1.23) | **3.75 × 10^-8^** | 2.60 × 10^-5^ | rs7209200 | 42.99 |
| cg16612511 | 17 | 4,927,006 | exonic | *KIF1C* | BIOS | 0.02 | 19 | 1.10 (1.07 ± 1.15) | **5.80 × 10^-8^** | 3.67 × 10^-5^ | rs7209200 | 42.93 |
| cg26848071 | 17 | 4,927,715 | UTR3 | *KIF1C* | FHS | 0.17 | 30 | 1.08 (1.05 ± 1.11) | **6.45 × 10^-8^** | 4.02 × 10^-5^ | rs7209200 | 42.23 |
| cg26848071 | 17 | 4,927,715 | UTR3 | *KIF1C* | BIOS | 0.31 | 51 | 1.03 (1.02 ± 1.04) | **1.79 × 10^-7^** | 9.07 × 10^-5^ | rs7209200 | 42.23 |
| cg07713929 | 17 | 5,000,803 | intergenic | *ZFP3, ZNF232* | BIOS | 0.04 | 23 | 0.91 (0.88 ± 0.94) | **6.63 × 10^-10^** | 8.35 × 10^-7^ | rs9916042 | -16.36 |
| cg07713929 | 17 | 5,000,803 | intergenic | *ZFP3, ZNF232* | FHS | 0.04 | 61 | 0.88 (0.83 ± 0.93) | 8.66 × 10^-6^ | 2.26 × 10^-3^ | rs9916042 | -16.36 |
| cg08331138 | 17 | 5,001,281 | intergenic | *ZFP3, ZNF232* | BIOS | 0.19 | 69 | 0.97 (0.96 ± 0.99) | 2.14 × 10^-5^ | 4.71 × 10^-3^ | rs9916042 | -16.83 |
| cg08331138 | 17 | 5,001,281 | intergenic | *ZFP3, ZNF232* | FHS | 0.18 | 26 | 0.94 (0.91 ± 0.97) | 1.50 × 10^-4^ | 2.02 × 10^-2^ | rs9916042 | -16.83 |
| cg03226208 | 17 | 5,004,183 | intergenic | *ZFP3, ZNF232* | BIOS | 0.05 | 16 | 1.09 (1.05 ± 1.12) | **1.75 × 10^-7^** | 8.95 × 10^-5^ | rs9916042 | -19.74 |
| cg23165431 | 17 | 5,014,519 | intronic | *ZNF232* | BIOS | 0.02 | 6 | 0.87 (0.83 ± 0.91) | **1.16 × 10^-9^** | 1.29 × 10^-6^ | rs9916042 | -30.07 |
| cg23165431 | 17 | 5,014,519 | intronic | *ZNF232* | FHS | 0.01 | 28 | 0.81 (0.72 ± 0.90) | 1.82 × 10^-4^ | 2.32 × 10^-2^ | rs9916042 | -30.07 |
| cg09151754 | 17 | 5,019,310 | intronic | *ZNF232* | BIOS | 0.03 | 42 | 0.92 (0.89 ± 0.95) | **3.00 × 10^-7^** | 1.43 × 10^-4^ | rs9916042 | -34.86 |
| cg05918002 | 17 | 5,019,452 | intronic | *ZNF232* | BIOS | 0.11 | 56 | 0.97 (0.96 ± 0.99) | 1.17 × 10^-4^ | 1.68 × 10^-2^ | rs9916042 | -35.01 |
| cg01120173 | 17 | 5,019,669 | intronic | *CRHR1, LINC02210-CRHR1* | BIOS | 0.08 | 40 | 0.96 (0.94 ± 0.97) | 3.23 × 10^-6^ | 1.01 × 10^-3^ | rs9916042 | -35.22 |
| cg11588975 | 17 | 5,026,759 | intronic | *USP6* | FHS | 0.07 | 26 | 0.89 (0.84 ± 0.94) | 8.24 × 10^-6^ | 2.18 × 10^-3^ | rs9916042 | -42.31 |
| cg05441897 | 17 | 5,026,769 | intronic | *ABHD17A* | FHS | 0.10 | 87 | 0.93 (0.90 ± 0.96) | 4.47 × 10^-5^ | 8.28 × 10^-3^ | rs9916042 | -42.32 |
| cg02114696 | 17 | 5,026,840 | intronic | *USP6* | BIOS | 0.12 | 25 | 0.96 (0.94 ± 0.98) | 1.37 × 10^-6^ | 5.07 × 10^-4^ | rs9916042 | -42.39 |
| cg02114696 | 17 | 5,026,840 | intronic | *USP6* | FHS | 0.03 | 46 | 0.86 (0.80 ± 0.92) | 1.16 × 10^-5^ | 2.88 × 10^-3^ | rs9916042 | -42.39 |
| cg12763546 | 17 | 5,027,051 | upstream | *RASAL1* | FHS | 0.03 | 16 | 0.83 (0.76 ± 0.91) | 2.75 × 10^-5^ | 5.72 × 10^-3^ | rs9916042 | -42.60 |
| cg05549575 | 17 | 5,137,892 | ncRNA_exonic | *LOC100130950* | BIOS | 0.04 | 8 | 0.89 (0.87 ± 0.92) | **2.29 × 10^-14^** | 9.11 × 10^-11^ | rs9916042 | -153.45 |
| cg05549575 | 17 | 5,137,892 | ncRNA_exonic | *LOC100130950* | FHS | 0.02 | 23 | 0.77 (0.70 ± 0.84) | **2.71 × 10^-9^** | 2.65 × 10^-6^ | rs9916042 | -153.45 |
| cg16837973 | 17 | 5,138,634 | ncRNA_exonic | *LOC100130950* | BIOS | 0.09 | 25 | 0.92 (0.91 ± 0.94) | **8.94 × 10^-15^** | 4.02 × 10^-11^ | rs9916042 | -154.19 |
| cg16837973 | 17 | 5,138,634 | ncRNA_exonic | *LOC100130950* | FHS | 0.06 | 10 | 0.79 (0.74 ± 0.84) | **2.27 × 10^-14^** | 9.11 × 10^-11^ | rs9916042 | -154.19 |
| cg21337881 | 17 | 5,138,645 | ncRNA_exonic | *LOC100130950* | BIOS | 0.14 | 35 | 0.94 (0.93 ± 0.96) | **2.48 × 10^-14^** | 9.59 × 10^-11^ | rs9916042 | -154.20 |
| cg21337881 | 17 | 5,138,645 | ncRNA_exonic | *LOC100130950* | FHS | 0.11 | 24 | 0.88 (0.84 ± 0.91) | **2.72 × 10^-12^** | 7.07 × 10^-9^ | rs9916042 | -154.20 |
| cg17588003 | 17 | 5,138,696 | ncRNA_exonic | *LOC100130950* | BIOS | 0.11 | 14 | 0.93 (0.92 ± 0.95) | **4.69 × 10^-14^** | 1.70 × 10^-10^ | rs9916042 | -154.25 |
| cg17588003 | 17 | 5,138,696 | ncRNA_exonic | *LOC100130950* | FHS | 0.14 | 36 | 0.90 (0.87 ± 0.92) | **9.60 × 10^-12^** | 2.16 × 10^-8^ | rs9916042 | -154.25 |
| cg15863539 | 17 | 17,716,950 | exonic | *SREBF1* | FHS | 0.01 | 27 | 0.84 (0.76 ± 0.92) | 4.45 × 10^-4^ | 4.29 × 10^-2^ | rs2242595 | 342.50 |
| cg13198297 | 17 | 18,046,564 | intronic | *MYO15A* | BIOS | 0.59 | 20 | 0.98 (0.97 ± 0.99) | 2.43 × 10^-4^ | 2.83 × 10^-2^ | rs2242595 | 12.89 |
| cg13198297 | 17 | 18,046,564 | intronic | *MYO15A* | FHS | 0.46 | 30 | 0.97 (0.95 ± 0.99) | 2.86 × 10^-4^ | 3.16 × 10^-2^ | rs2242595 | 12.89 |
| cg06147733 | 17 | 18,128,806 | upstream | *LLGL1* | BIOS | 0.02 | 5 | 0.89 (0.83 ± 0.95) | 5.39 × 10^-4^ | 4.91 × 10^-2^ | rs2242595 | -69.35 |
| cg19107120 | 17 | 42,430,376 | UTR3 | *GRN* | BIOS | 0.01 | 2 | 0.80 (0.74 ± 0.86) | **7.43 × 10^-10^** | 9.02 × 10^-7^ | rs5848 | -0.13 |
| cg13777937 | 17 | 42,441,432 | upstream | *FAM171A2* | BIOS | 0.01 | 5 | 0.85 (0.78 ± 0.92) | 1.02 × 10^-4^ | 1.52 × 10^-2^ | rs708382 | 0.91 |
| cg16233074 | 17 | 42,445,042 | upstream | *STX1B* | BIOS | 0.11 | 6 | 0.94 (0.92 ± 0.97) | 2.29 × 10^-6^ | 7.62 × 10^-4^ | rs708382 | -2.70 |
| cg16233074 | 17 | 42,445,042 | intergenic | *FAM171A2, ITGA2B* | FHS | 0.08 | 59 | 0.91 (0.86 ± 0.95) | 8.90 × 10^-5^ | 1.38 × 10^-2^ | rs708382 | -2.70 |
| cg17049621 | 17 | 42,635,778 | exonic | *FZD2* | BIOS | 0.33 | 31 | 1.02 (1.01 ± 1.04) | 7.58 × 10^-5^ | 1.23 × 10^-2^ | rs708382 | -193.43 |
| cg17049621 | 17 | 42,635,778 | exonic | *FZD2* | FHS | 0.17 | 26 | 1.06 (1.03 ± 1.09) | 1.50 × 10^-4^ | 2.02 × 10^-2^ | rs708382 | -193.43 |
| cg09339219 | 17 | 42,635,789 | exonic | *FZD2* | FHS | 0.17 | 21 | 1.06 (1.03 ± 1.09) | 1.11 × 10^-4^ | 1.62 × 10^-2^ | rs708382 | -193.45 |
| cg09339219 | 17 | 42,635,789 | exonic | *FZD2* | BIOS | 0.26 | 24 | 1.03 (1.01 ± 1.04) | 1.69 × 10^-4^ | 2.19 × 10^-2^ | rs708382 | -193.45 |
| cg20301962 | 17 | 42,636,006 | exonic | *FZD2* | FHS | 0.07 | 14 | 1.10 (1.05 ± 1.16) | 1.11 × 10^-4^ | 1.62 × 10^-2^ | rs708382 | -193.66 |
| cg10624665 | 17 | 42,636,570 | exonic | *FZD2* | BIOS | 0.29 | 29 | 1.02 (1.01 ± 1.04) | 1.45 × 10^-4^ | 1.98 × 10^-2^ | rs708382 | -194.23 |
| cg10624665 | 17 | 42,636,570 | exonic | *FZD2* | FHS | 0.22 | 24 | 1.05 (1.02 ± 1.08) | 2.16 × 10^-4^ | 2.62 × 10^-2^ | rs708382 | -194.23 |
| cg10387761 | 17 | 42,856,823 | intronic | *ADAM11* | FHS | 0.03 | 6 | 1.19 (1.09 ± 1.31) | 1.35 × 10^-4^ | 1.88 × 10^-2^ | rs708382 | -414.48 |
| cg27455331 | 17 | 47,338,178 | intergenic | *FLJ40194, MIR6129* | BIOS | 0.02 | 2 | 0.84 (0.78 ± 0.90) | 3.40 × 10^-7^ | 1.57 × 10^-4^ | rs28394864 | 112.60 |
| cg11906021 | 17 | 47,467,221 | intergenic | *LOC102724596, PHB* | FHS | 0.01 | 12 | 0.75 (0.67 ± 0.85) | 1.29 × 10^-6^ | 4.88 × 10^-4^ | rs28394864 | -16.45 |
| cg11906021 | 17 | 47,467,221 | intergenic | *LOC102724596, PHB* | BIOS | 0.02 | 10 | 0.88 (0.83 ± 0.93) | 2.63 × 10^-6^ | 8.53 × 10^-4^ | rs28394864 | -16.45 |
| cg04005334 | 17 | 47,482,084 | UTR3 | *PHB* | FHS | 0.03 | 7 | 0.82 (0.75 ± 0.89) | 9.71 × 10^-6^ | 2.48 × 10^-3^ | rs28394864 | -31.31 |
| cg02823457 | 17 | 47,583,920 | exonic | *NGFR* | BIOS | 0.01 | 13 | 0.89 (0.83 ± 0.95) | 5.49 × 10^-4^ | 4.95 × 10^-2^ | rs28394864 | -133.15 |
| cg04266202 | 17 | 56,352,895 | intronic | *MPO* | FHS | 0.03 | 26 | 1.16 (1.08 ± 1.24) | 2.88 × 10^-5^ | 5.90 × 10^-3^ | rs2632516 | 56.19 |
| cg04611801 | 17 | 56,402,029 | intronic | *TSPOAP1* | BIOS | 0.01 | 9 | 1.11 (1.05 ± 1.17) | 1.64 × 10^-4^ | 2.15 × 10^-2^ | rs2632516 | 7.06 |
| cg04740898 | 17 | 56,402,669 | intronic | *TSPOAP1* | BIOS | 0.07 | 31 | 0.94 (0.92 ± 0.97) | 1.31 × 10^-5^ | 3.22 × 10^-3^ | rs2632516 | 6.42 |
| cg04740898 | 17 | 56,402,669 | intronic | *TSPOAP1* | FHS | 0.02 | 30 | 0.85 (0.77 ± 0.93) | 3.65 × 10^-4^ | 3.74 × 10^-2^ | rs2632516 | 6.42 |
| cg20783697 | 17 | 56,407,147 | ncRNA_intronic | *TSPOAP1-AS1* | BIOS | 0.04 | 24 | 0.93 (0.90 ± 0.96) | 7.40 × 10^-6^ | 2.00 × 10^-3^ | rs2632516 | 1.94 |
| cg25537245 | 17 | 56,413,890 | ncRNA_intronic | *TSPOAP1-AS1* | BIOS | 0.02 | 36 | 0.89 (0.85 ± 0.93) | 3.62 × 10^-7^ | 1.64 × 10^-4^ | rs2632516 | -4.80 |
| cg14996805 | 17 | 56,430,170 | ncRNA_exonic | *TSPOAP1-AS1* | BIOS | 0.08 | 9 | 0.94 (0.92 ± 0.97) | 3.57 × 10^-6^ | 1.10 × 10^-3^ | rs2632516 | -21.08 |
| cg14996805 | 17 | 56,430,170 | ncRNA_exonic | *TSPOAP1-AS1* | FHS | 0.07 | 16 | 0.90 (0.86 ± 0.95) | 8.45 × 10^-5^ | 1.33 × 10^-2^ | rs2632516 | -21.08 |
| cg11610460 | 17 | 56,432,612 | intronic | *RNF43* | FHS | 0.01 | 13 | 0.81 (0.72 ± 0.91) | 4.84 × 10^-4^ | 4.56 × 10^-2^ | rs2632516 | -23.52 |
| cg04780984 | 17 | 56,454,528 | exonic | *SPI1* | BIOS | 0.03 | 4 | 1.13 (1.07 ± 1.19) | 1.57 × 10^-5^ | 3.71 × 10^-3^ | rs2632516 | -45.44 |
| cg04780984 | 17 | 56,454,528 | intergenic | *DUS3L, NRTN* | FHS | 0.08 | 16 | 1.11 (1.06 ± 1.17) | 2.60 × 10^-5^ | 5.50 × 10^-3^ | rs2632516 | -45.44 |
| cg15417365 | 17 | 56,609,082 | ncRNA_intronic | *SEPTIN4-AS1* | BIOS | 0.29 | 10 | 1.03 (1.02 ± 1.05) | 3.10 × 10^-5^ | 6.22 × 10^-3^ | rs2632516 | -199.99 |
| cg15417365 | 17 | 56,609,082 | ncRNA_intronic | *SEPTIN4-AS1* | FHS | 0.15 | 31 | 1.06 (1.03 ± 1.10) | 1.48 × 10^-4^ | 2.01 × 10^-2^ | rs2632516 | -199.99 |
| cg13262310 | 17 | 56,609,582 | ncRNA_intronic | *SEPTIN4-AS1* | FHS | 0.01 | 40 | 0.79 (0.71 ± 0.87) | 3.54 × 10^-6^ | 1.09 × 10^-3^ | rs2632516 | -200.49 |
| cg12778183 | 17 | 56,769,387 | UTR5 | *TEX14* | BIOS | 0.03 | 9 | 1.09 (1.04 ± 1.14) | 4.74 × 10^-4^ | 4.49 × 10^-2^ | rs2632516 | -360.30 |
| cg19532212 | 17 | 56,769,430 | upstream | *RAD51C, TEX14* | FHS | 0.01 | 35 | 1.26 (1.13 ± 1.39) | 1.21 × 10^-5^ | 2.99 × 10^-3^ | rs2632516 | -360.34 |
| cg19532212 | 17 | 56,769,430 | upstream | *RAD51C, TEX14* | BIOS | 0.03 | 3 | 1.12 (1.06 ± 1.19) | 1.29 × 10^-4^ | 1.81 × 10^-2^ | rs2632516 | -360.34 |
| cg16398362 | 17 | 56,769,633 | upstream | *RAD51C, TEX14* | FHS | 0.02 | 12 | 1.28 (1.15 ± 1.43) | 7.75 × 10^-6^ | 2.08 × 10^-3^ | rs2632516 | -360.54 |
| cg02110529 | 17 | 56,769,696 | upstream | *RAD51C, TEX14* | FHS | 0.01 | 21 | 1.26 (1.12 ± 1.41) | 6.96 × 10^-5^ | 1.16 × 10^-2^ | rs2632516 | -360.61 |
| cg26161329 | 17 | 56,832,991 | upstream | *PPM1E* | FHS | 0.01 | 3 | 0.78 (0.69 ± 0.88) | 1.11 × 10^-4^ | 1.62 × 10^-2^ | rs2632516 | -423.90 |
| cg26161329 | 17 | 56,832,991 | upstream | *PPM1E* | BIOS | 0.05 | 7 | 0.95 (0.92 ± 0.98) | 4.17 × 10^-4^ | 4.13 × 10^-2^ | rs2632516 | -423.90 |
| cg16477091 | 17 | 56,833,000 | upstream | *PPM1E* | FHS | 0.03 | 14 | 0.84 (0.78 ± 0.91) | 2.03 × 10^-5^ | 4.52 × 10^-3^ | rs2632516 | -423.91 |
| cg25533154 | 17 | 56,834,061 | intronic | *PPM1E* | BIOS | 0.01 | 12 | 1.13 (1.06 ± 1.21) | 1.59 × 10^-4^ | 2.10 × 10^-2^ | rs2632516 | -424.97 |
| cg04662983 | 17 | 56,834,321 | intronic | *HLA-E* | FHS | 0.02 | 25 | 0.84 (0.77 ± 0.91) | 3.05 × 10^-5^ | 6.16 × 10^-3^ | rs2632516 | -425.23 |
| cg04662983 | 17 | 56,834,321 | intronic | *PPM1E* | BIOS | 0.03 | 9 | 0.93 (0.89 ± 0.96) | 1.64 × 10^-4^ | 2.14 × 10^-2^ | rs2632516 | -425.23 |
| cg06641342 | 17 | 61,514,920 | exonic | *CYB561* | BIOS | 0.03 | 11 | 1.08 (1.03 ± 1.12) | 3.77 × 10^-4^ | 3.82 × 10^-2^ | rs6504163 | 30.86 |
| cg21657705 | 17 | 61,574,500 | exonic | *ACE* | FHS | 0.09 | 13 | 0.85 (0.81 ± 0.89) | **5.02 × 10^-13^** | 1.67 × 10^-9^ | rs6504163 | -28.72 |
| cg21657705 | 17 | 61,574,500 | exonic | *ACE* | BIOS | 0.18 | 8 | 0.93 (0.92 ± 0.95) | **1.74 × 10^-11^** | 3.40 × 10^-8^ | rs6504163 | -28.72 |
| cg20157577 | 17 | 61,780,203 | UTR3 | *STRADA* | BIOS | 0.04 | 35 | 0.93 (0.90 ± 0.95) | 3.54 × 10^-7^ | 1.62 × 10^-4^ | rs6504163 | -234.42 |
| cg11737831 | 19 | 980,707 | intergenic | *ARID3A, WDR18* | BIOS | 0.03 | 20 | 1.09 (1.04 ± 1.13) | 5.28 × 10^-5^ | 9.34 × 10^-3^ | rs12151021 | 70.17 |
| cg14010550 | 19 | 1,009,642 | UTR3 | *GRIN3B* | BIOS | 0.03 | 17 | 1.08 (1.04 ± 1.13) | 3.30 × 10^-4^ | 3.51 × 10^-2^ | rs12151021 | 41.23 |
| cg11015549 | 19 | 1,026,207 | upstream | *CNN2* | FHS | 0.02 | 14 | 0.81 (0.72 ± 0.90) | 2.11 × 10^-4^ | 2.56 × 10^-2^ | rs12151021 | 24.67 |
| cg10749413 | 19 | 1,041,020 | intronic | *ABCA7* | BIOS | 0.02 | 13 | 1.19 (1.12 ± 1.26) | **2.55 × 10^-8^** | 1.88 × 10^-5^ | rs12151021 | 9.85 |
| cg05989429 | 19 | 1,041,026 | intronic | *ABCA7* | BIOS | 0.02 | 24 | 1.19 (1.12 ± 1.25) | **9.00 × 10^-10^** | 1.05 × 10^-6^ | rs12151021 | 9.85 |
| cg05372495 | 19 | 1,063,624 | exonic | *ABCA7* | BIOS | 0.12 | 14 | 1.06 (1.04 ± 1.09) | 3.26 × 10^-7^ | 1.53 × 10^-4^ | rs4147929 | -0.18 |
| cg05372495 | 19 | 1,063,624 | exonic | *ABCA7* | FHS | 0.02 | 19 | 1.24 (1.11 ± 1.38) | 8.99 × 10^-5^ | 1.39 × 10^-2^ | rs4147929 | -0.18 |
| cg21995147 | 19 | 1,064,017 | intronic | *ABCA7* | BIOS | 0.11 | 14 | 1.06 (1.04 ± 1.09) | 1.32 × 10^-6^ | 4.93 × 10^-4^ | rs4147929 | -0.57 |
| cg26576206 | 19 | 1,064,938 | exonic | *ABCA7* | FHS | 0.13 | 15 | 0.91 (0.88 ± 0.95) | 2.03 × 10^-6^ | 6.97 × 10^-4^ | rs4147929 | -1.50 |
| cg19548313 | 19 | 1,065,712 | upstream, downstream | *ARHGAP45, ABCA7* | BIOS | 0.10 | 14 | 0.95 (0.92 ± 0.97) | 8.62 × 10^-6^ | 2.26 × 10^-3^ | rs4147929 | -2.27 |
| cg18073514 | 19 | 1,065,728 | upstream, downstream | *ARHGAP45, ABCA7* | FHS | 0.03 | 8 | 0.83 (0.76 ± 0.91) | 4.01 × 10^-5^ | 7.66 × 10^-3^ | rs4147929 | -2.29 |
| cg18073514 | 19 | 1,065,728 | upstream, downstream | *ARHGAP45, ABCA7* | BIOS | 0.09 | 9 | 0.95 (0.93 ± 0.98) | 2.24 × 10^-4^ | 2.68 × 10^-2^ | rs4147929 | -2.29 |
| cg24024661 | 19 | 1,074,425 | intronic | *ARHGAP45* | FHS | 0.07 | 10 | 1.14 (1.08 ± 1.20) | 5.41 × 10^-7^ | 2.32 × 10^-4^ | rs4147929 | -10.98 |
| cg24024661 | 19 | 1,074,425 | ncRNA_exonic | *LOC643072* | BIOS | 0.15 | 12 | 1.05 (1.03 ± 1.07) | 1.88 × 10^-6^ | 6.58 × 10^-4^ | rs4147929 | -10.98 |
| cg16239536 | 19 | 1,079,617 | intronic | *ARHGAP45* | BIOS | 0.01 | 7 | 0.85 (0.79 ± 0.92) | 1.99 × 10^-5^ | 4.44 × 10^-3^ | rs4147929 | -16.17 |
| cg03751944 | 19 | 1,081,936 | exonic | *ARHGAP45* | FHS | 0.01 | 23 | 0.74 (0.65 ± 0.84) | 1.53 × 10^-6^ | 5.57 × 10^-4^ | rs4147929 | -18.49 |
| cg03751944 | 19 | 1,081,936 | exonic | *ARHGAP45* | BIOS | 0.06 | 19 | 0.93 (0.90 ± 0.97) | 1.02 × 10^-4^ | 1.53 × 10^-2^ | rs4147929 | -18.49 |
| cg04903600 | 19 | 1,105,746 | exonic | *GPX4* | FHS | 0.10 | 15 | 1.11 (1.06 ± 1.15) | 1.22 × 10^-6^ | 4.64 × 10^-4^ | rs4147929 | -42.30 |
| cg04903600 | 19 | 1,105,746 | exonic | *GPX4* | BIOS | 0.13 | 26 | 1.05 (1.03 ± 1.07) | 1.86 × 10^-6^ | 6.55 × 10^-4^ | rs4147929 | -42.30 |
| cg14894245 | 19 | 1,106,640 | UTR3 | *GPX4* | BIOS | 0.05 | 13 | 1.08 (1.04 ± 1.12) | 5.97 × 10^-6^ | 1.68 × 10^-3^ | rs4147929 | -43.20 |
| cg14894245 | 19 | 1,106,640 | UTR3 | *GPX4* | FHS | 0.05 | 31 | 1.11 (1.06 ± 1.17) | 7.22 × 10^-5^ | 1.19 × 10^-2^ | rs4147929 | -43.20 |
| cg17728125 | 19 | 1,113,155 | intronic | *SBNO2* | FHS | 0.02 | 19 | 1.16 (1.08 ± 1.26) | 1.24 × 10^-4^ | 1.75 × 10^-2^ | rs4147929 | -49.71 |
| cg07228817 | 19 | 1,269,771 | ncRNA_intronic | *CIRBP-AS1* | FHS | 0.01 | 14 | 1.29 (1.12 ± 1.49) | 3.56 × 10^-4^ | 3.68 × 10^-2^ | rs4147929 | -206.33 |
| cg19499085 | 19 | 1,796,437 | intergenic | *LRRFIP2, LOC152048* | FHS | 0.01 | 4 | 1.54 (1.29 ± 1.84) | 1.38 × 10^-6^ | 5.08 × 10^-4^ | rs149080927 | 57.82 |
| cg25275011 | 19 | 1,796,985 | exonic | *ATP8B3* | FHS | 0.01 | 36 | 1.24 (1.13 ± 1.36) | 3.13 × 10^-6^ | 9.86 × 10^-4^ | rs149080927 | 57.27 |
| cg21885795 | 19 | 1,809,756 | intronic | *ATP8B3* | FHS | 0.01 | 3 | 1.45 (1.23 ± 1.70) | 8.28 × 10^-6^ | 2.18 × 10^-3^ | rs149080927 | 44.50 |
| cg03924776 | 19 | 1,826,924 | exonic | *REXO1* | FHS | 0.01 | 8 | 1.45 (1.25 ± 1.69) | 9.02 × 10^-7^ | 3.60 × 10^-4^ | rs149080927 | 27.33 |
| cg03924776 | 19 | 1,826,924 | exonic | *REXO1* | BIOS | 0.10 | 8 | 1.04 (1.02 ± 1.07) | 3.86 × 10^-4^ | 3.88 × 10^-2^ | rs149080927 | 27.33 |
| cg10370574 | 19 | 1,840,461 | intronic | *REXO1* | FHS | 0.27 | 67 | 0.93 (0.91 ± 0.96) | **7.57 × 10^-9^** | 6.39 × 10^-6^ | rs149080927 | 13.79 |
| cg10370574 | 19 | 1,840,461 | intronic | *REXO1* | BIOS | 0.63 | 12 | 0.98 (0.97 ± 0.99) | 3.65 × 10^-5^ | 7.13 × 10^-3^ | rs149080927 | 13.79 |
| cg11579407 | 19 | 1,847,575 | intronic | *REXO1* | BIOS | 0.25 | 15 | 1.04 (1.02 ± 1.06) | 2.62 × 10^-6^ | 8.51 × 10^-4^ | rs149080927 | 6.68 |
| cg11579407 | 19 | 1,847,575 | intronic | *REXO1* | FHS | 0.12 | 10 | 1.11 (1.06 ± 1.16) | 6.39 × 10^-6^ | 1.77 × 10^-3^ | rs149080927 | 6.68 |
| cg21201285 | 19 | 1,847,690 | intronic | *REXO1* | BIOS | 0.01 | 6 | 1.29 (1.18 ± 1.41) | 9.45 × 10^-9^ | 7.70 × 10^-6^ | rs149080927 | 6.56 |
| cg08620751 | 19 | 1,851,750 | downstream | *KLF16* | FHS | 0.02 | 27 | 1.23 (1.14 ± 1.34) | 3.77 × 10^-7^ | 1.70 × 10^-4^ | rs149080927 | 2.50 |
| cg13382072 | 19 | 1,851,882 | upstream | *FGF17* | FHS | 0.03 | 3 | 1.26 (1.13 ± 1.41) | 2.05 × 10^-5^ | 4.55 × 10^-3^ | rs149080927 | 2.37 |
| cg05006231 | 19 | 1,852,165 | downstream | *KLF16* | FHS | 0.02 | 15 | 1.27 (1.12 ± 1.43) | 2.20 × 10^-4^ | 2.64 × 10^-2^ | rs149080927 | 2.09 |
| cg08287334 | 19 | 1,854,633 | exonic | *KLF16* | BIOS | 0.02 | 5 | 1.10 (1.05 ± 1.16) | 2.26 × 10^-4^ | 2.69 × 10^-2^ | rs149080927 | -0.38 |
| cg08525314 | 19 | 1,854,819 | intronic | *KLF16* | FHS | 0.02 | 27 | 1.24 (1.15 ± 1.34) | **4.17 × 10^-8^** | 2.84 × 10^-5^ | rs149080927 | -0.57 |
| cg08525314 | 19 | 1,854,819 | intronic | *KLF16* | BIOS | 0.15 | 5 | 1.04 (1.02 ± 1.06) | 9.88 × 10^-5^ | 1.49 × 10^-2^ | rs149080927 | -0.57 |
| cg04998634 | 19 | 1,857,004 | intronic | *KLF16* | BIOS | 0.02 | 4 | 0.83 (0.77 ± 0.89) | 1.27 × 10^-7^ | 7.06 × 10^-5^ | rs149080927 | -2.75 |
| cg23290456 | 19 | 1,859,341 | intronic | *KLF16* | BIOS | 0.05 | 5 | 0.93 (0.90 ± 0.96) | 8.58 × 10^-5^ | 1.35 × 10^-2^ | rs149080927 | -5.09 |
| cg03157040 | 19 | 1,861,890 | intronic | *KLF16* | BIOS | 0.01 | 18 | 1.17 (1.11 ± 1.25) | **1.23 × 10^-7^** | 6.94 × 10^-5^ | rs149080927 | -7.64 |
| cg19687457 | 19 | 1,865,884 | intronic | *GPC2* | BIOS | 0.11 | 15 | 0.93 (0.91 ± 0.96) | **1.24 × 10^-7^** | 6.94 × 10^-5^ | rs149080927 | -11.63 |
| cg19687457 | 19 | 1,865,884 | intergenic | *KLF16, ABHD17A* | FHS | 0.03 | 22 | 0.85 (0.79 ± 0.93) | 2.78 × 10^-4^ | 3.09 × 10^-2^ | rs149080927 | -11.63 |
| cg07136873 | 19 | 1,876,580 | downstream | *ABHD17A* | BIOS | 0.36 | 16 | 0.97 (0.96 ± 0.99) | 3.12 × 10^-4^ | 3.37 × 10^-2^ | rs149080927 | -22.33 |
| cg07528237 | 19 | 1,884,145 | intronic | *USP6* | FHS | 0.06 | 10 | 1.13 (1.07 ± 1.20) | 4.47 × 10^-5^ | 8.28 × 10^-3^ | rs149080927 | -29.89 |
| cg22684041 | 19 | 1,904,554 | upstream | *ADAT3, SCAMP4* | FHS | 0.23 | 23 | 1.08 (1.05 ± 1.11) | **7.57 × 10^-8^** | 4.56 × 10^-5^ | rs149080927 | -50.30 |
| cg11783264 | 19 | 1,905,046 | intronic | *TLCD3B* | FHS | 0.13 | 19 | 1.09 (1.05 ± 1.13) | 2.96 × 10^-6^ | 9.45 × 10^-4^ | rs149080927 | -50.79 |
| cg25246158 | 19 | 1,940,184 | UTR5 | *BLNK* | FHS | 0.01 | 28 | 0.79 (0.71 ± 0.88) | 3.59 × 10^-5^ | 7.05 × 10^-3^ | rs149080927 | -85.93 |
| cg01396723 | 19 | 45,146,828 | upstream | *PVR* | FHS | 0.03 | 17 | 0.66 (0.60 ± 0.73) | **2.61 × 10^-16^** | 1.50 × 10^-12^ | rs429358 | 265.11 |
| cg01396723 | 19 | 45,146,828 | upstream | *PVR* | BIOS | 0.08 | 19 | 0.91 (0.89 ± 0.94) | **2.47 × 10^-11^** | 4.51 × 10^-8^ | rs429358 | 265.11 |
| cg22580353 | 19 | 45,146,900 | upstream | *PVR* | FHS | 0.04 | 16 | 0.81 (0.76 ± 0.87) | **3.66 × 10^-9^** | 3.42 × 10^-6^ | rs429358 | 265.04 |
| cg01496416 | 19 | 45,147,715 | intronic | *PVR* | FHS | 0.06 | 11 | 0.82 (0.77 ± 0.87) | **2.92 × 10^-11^** | 5.17 × 10^-8^ | rs429358 | 264.23 |
| cg01496416 | 19 | 45,147,715 | intronic | *PVR* | BIOS | 0.08 | 6 | 0.91 (0.89 ± 0.94) | **5.28 × 10^-11^** | 8.87 × 10^-8^ | rs429358 | 264.23 |
| cg23942508 | 19 | 45,201,924 | upstream | *CEACAM16* | BIOS | 0.01 | 13 | 1.21 (1.13 ± 1.30) | **1.60 × 10^-7^** | 8.39 × 10^-5^ | rs429358 | 210.02 |
| cg06673536 | 19 | 45,213,941 | UTR3 | *CEACAM16* | BIOS | 0.08 | 40 | 1.09 (1.07 ± 1.12) | **3.85 × 10^-15^** | 1.94 × 10^-11^ | rs429358 | 198.00 |
| cg06673536 | 19 | 45,213,941 | UTR3 | *CEACAM16* | FHS | 0.03 | 39 | 1.31 (1.20 ± 1.43) | **4.21 × 10^-10^** | 5.56 × 10^-7^ | rs429358 | 198.00 |
| cg26470501 | 19 | 45,252,955 | intronic | *BCL3* | FHS | 0.06 | 24 | 0.79 (0.75 ± 0.84) | **1.89 × 10^-14^** | 8.06 × 10^-11^ | rs429358 | 158.99 |
| cg26470501 | 19 | 45,252,955 | intronic | *BCL3* | BIOS | 0.07 | 30 | 1.06 (1.03 ± 1.08) | 2.19 × 10^-6^ | 7.37 × 10^-4^ | rs429358 | 158.99 |
| cg25644380 | 19 | 45,262,982 | UTR3 | *BCL3* | FHS | 0.02 | 5 | 1.32 (1.16 ± 1.49) | 1.69 × 10^-5^ | 3.93 × 10^-3^ | rs429358 | 148.96 |
| cg23635599 | 19 | 45,281,006 | upstream | *CBLC* | BIOS | 0.05 | 24 | 1.08 (1.04 ± 1.12) | 7.94 × 10^-5^ | 1.28 × 10^-2^ | rs429358 | 130.94 |
| cg16529268 | 19 | 45,281,284 | exonic | *CBLC* | BIOS | 0.17 | 53 | 1.04 (1.02 ± 1.06) | 2.15 × 10^-5^ | 4.72 × 10^-3^ | rs429358 | 130.66 |
| cg04434147 | 19 | 45,281,287 | exonic | *CBLC* | BIOS | 0.09 | 49 | 1.07 (1.05 ± 1.10) | **4.85 × 10^-9^** | 4.34 × 10^-6^ | rs429358 | 130.65 |
| cg06522456 | 19 | 45,311,781 | upstream | *BCAM* | BIOS | 0.02 | 44 | 1.15 (1.11 ± 1.20) | **9.91 × 10^-13^** | 3.11 × 10^-9^ | rs429358 | 100.16 |
| cg08319238 | 19 | 45,312,525 | intronic | *BCAM* | BIOS | 0.02 | 5 | 0.83 (0.77 ± 0.90) | 5.38 × 10^-6^ | 1.54 × 10^-3^ | rs429358 | 99.42 |
| cg05670193 | 19 | 45,324,246 | UTR3 | *BCAM* | BIOS | 0.05 | 7 | 1.08 (1.04 ± 1.13) | 2.49 × 10^-4^ | 2.88 × 10^-2^ | rs429358 | 87.70 |
| cg11670000 | 19 | 45,352,950 | intronic | *NECTIN2* | BIOS | 0.19 | 54 | 0.95 (0.94 ± 0.97) | **4.64 × 10^-9^** | 4.22 × 10^-6^ | rs429358 | 58.99 |
| cg14123992 | 19 | 45,407,868 | downstream | *TOMM40* | BIOS | 0.03 | 9 | 3.52 (2.62 ± 4.73) | **5.72 × 10^-17^** | 3.40 × 10^-13^ | rs429358 | 4.07 |
| cg04406254 | 19 | 45,407,945 | downstream | *TOMM40* | BIOS | 0.02 | 11 | 1.70 (1.41 ± 2.04) | **1.46 × 10^-8^** | 1.14 × 10^-5^ | rs429358 | 4.00 |
| cg13496662 | 19 | 45,416,369 | intergenic | *APOE, APOC1* | BIOS | 0.02 | 14 | 0.57 (0.51 ± 0.63) | **1.29 × 10^-24^** | 3.58 × 10^-20^ | rs75627662 | -2.79 |
| cg07773593 | 19 | 45,417,793 | intronic | *APOC1* | BIOS | 0.20 | 38 | 1.15 (1.10 ± 1.21) | **3.47 × 10^-9^** | 3.26 × 10^-6^ | rs75627662 | -4.22 |
| cg13880303 | 19 | 45,417,814 | intronic | *APOC1* | BIOS | 0.15 | 43 | 1.11 (1.05 ± 1.17) | 1.31 × 10^-4^ | 1.83 × 10^-2^ | rs75627662 | -4.24 |
| cg05644480 | 19 | 45,418,020 | intronic | *APOC1* | BIOS | 0.07 | 34 | 1.25 (1.19 ± 1.32) | **1.62 × 10^-17^** | 1.08 × 10^-13^ | rs75627662 | -4.44 |
| cg08121984 | 19 | 45,429,870 | upstream | *APOC1P1* | BIOS | 0.05 | 65 | 0.79 (0.76 ± 0.81) | **1.61 × 10^-48^** | 2.68 × 10^-43^ | rs75627662 | -16.29 |
| cg04766076 | 19 | 45,444,811 | upstream | *APOC4* | BIOS | 0.08 | 14 | 0.78 (0.73 ± 0.84) | **8.29 × 10^-12^** | 1.94 × 10^-8^ | rs75627662 | -31.24 |
| cg06736138 | 19 | 45,444,860 | upstream | *APOC4* | FHS | 0.09 | 10 | 1.32 (1.25 ± 1.39) | **5.88 × 10^-25^** | 1.96 × 10^-20^ | rs75627662 | -31.28 |
| cg06736138 | 19 | 45,444,860 | upstream | *APOC4* | BIOS | 0.15 | 4 | 1.19 (1.14 ± 1.24) | **8.55 × 10^-15^** | 4.02 × 10^-11^ | rs75627662 | -31.28 |
| cg17769836 | 19 | 45,445,437 | upstream | *APOC4* | FHS | 0.02 | 11 | 1.62 (1.45 ± 1.81) | **4.02 × 10^-17^** | 2.48 × 10^-13^ | rs75627662 | -31.86 |
| cg17769836 | 19 | 45,445,437 | upstream | *APOC4* | BIOS | 0.04 | 15 | 1.16 (1.11 ± 1.21) | **5.35 × 10^-12^** | 1.37 × 10^-8^ | rs75627662 | -31.86 |
| cg04401876 | 19 | 45,445,449 | upstream | *APOC4* | FHS | 0.02 | 8 | 1.76 (1.55 ± 2.01) | **1.61 × 10^-17^** | 1.08 × 10^-13^ | rs75627662 | -31.87 |
| cg04401876 | 19 | 45,445,449 | intergenic | *EPHX2, CLU* | BIOS | 0.04 | 22 | 1.12 (1.07 ± 1.16) | **1.34 × 10^-7^** | 7.38 × 10^-5^ | rs75627662 | -31.87 |
| cg27353824 | 19 | 45,445,521 | ncRNA_exonic | *APOC4* | FHS | 0.01 | 13 | 2.13 (1.85 ± 2.46) | **5.28 × 10^-26^** | 2.20 × 10^-21^ | rs75627662 | -31.95 |
| cg27353824 | 19 | 45,445,521 | ncRNA_exonic | *APOC4* | BIOS | 0.02 | 2 | 1.38 (1.27 ± 1.50) | **2.30 × 10^-14^** | 9.11 × 10^-11^ | rs75627662 | -31.95 |
| cg25017250 | 19 | 45,445,693 | ncRNA_intronic | *APOC4* | FHS | 0.03 | 13 | 1.48 (1.36 ± 1.62) | **1.95 × 10^-19^** | 1.80 × 10^-15^ | rs75627662 | -32.12 |
| cg25017250 | 19 | 45,445,693 | ncRNA_intronic | *APOC4* | BIOS | 0.03 | 11 | 1.21 (1.15 ± 1.27) | **4.04 × 10^-14^** | 1.53 × 10^-10^ | rs75627662 | -32.12 |
| cg10169327 | 19 | 45,448,959 | ncRNA_exonic | *APOC4* | FHS | 0.29 | 20 | 1.13 (1.10 ± 1.16) | **3.78 × 10^-21^** | 5.71 × 10^-17^ | rs75627662 | -35.38 |
| cg10169327 | 19 | 45,448,959 | ncRNA_exonic | *APOC4* | BIOS | 0.37 | 14 | 1.06 (1.04 ± 1.07) | **4.45 × 10^-14^** | 1.64 × 10^-10^ | rs75627662 | -35.38 |
| cg27436184 | 19 | 45,449,006 | ncRNA_exonic | *APOC4* | FHS | 0.08 | 15 | 1.30 (1.23 ± 1.37) | **1.72 × 10^-19^** | 1.68 × 10^-15^ | rs75627662 | -35.43 |
| cg27436184 | 19 | 45,449,006 | ncRNA_exonic | *APOC4* | BIOS | 0.08 | 7 | 1.18 (1.13 ± 1.24) | **1.30 × 10^-14^** | 5.69 × 10^-11^ | rs75627662 | -35.43 |
| cg01958934 | 19 | 45,449,099 | ncRNA_exonic | *APOC4* | FHS | 0.45 | 13 | 1.09 (1.06 ± 1.11) | **9.14 × 10^-16^** | 4.90 × 10^-12^ | rs75627662 | -35.52 |
| cg01958934 | 19 | 45,449,099 | exonic | *ZNF668* | BIOS | 0.57 | 33 | 1.04 (1.02 ± 1.05) | **1.65 × 10^-9^** | 1.71 × 10^-6^ | rs75627662 | -35.52 |
| cg25746394 | 19 | 45,450,501 | ncRNA_intronic | *APOC4* | BIOS | 0.31 | 31 | 1.03 (1.02 ± 1.04) | **1.40 × 10^-11^** | 2.98 × 10^-8^ | rs75627662 | -36.93 |
| cg20090143 | 19 | 45,452,003 | exonic | *APOC2* | FHS | 0.19 | 16 | 1.13 (1.09 ± 1.18) | **5.34 × 10^-10^** | 6.88 × 10^-7^ | rs75627662 | -38.43 |
| cg20090143 | 19 | 45,452,003 | exonic | *APOC2* | BIOS | 0.27 | 8 | 1.05 (1.03 ± 1.07) | 3.01 × 10^-7^ | 1.43 × 10^-4^ | rs75627662 | -38.43 |
| cg25917893 | 19 | 45,458,573 | intronic | *CLPTM1* | BIOS | 0.02 | 7 | 1.17 (1.09 ± 1.24) | 1.95 × 10^-6^ | 6.77 × 10^-4^ | rs75627662 | -45.00 |
| cg19417129 | 19 | 45,459,933 | intronic | *CLPTM1* | BIOS | 0.34 | 17 | 0.93 (0.92 ± 0.94) | **1.16 × 10^-20^** | 1.48 × 10^-16^ | rs75627662 | -46.36 |
| cg19417129 | 19 | 45,459,933 | intronic | *CLPTM1* | FHS | 0.20 | 26 | 0.82 (0.78 ± 0.86) | **7.11 × 10^-19^** | 5.91 × 10^-15^ | rs75627662 | -46.36 |
| cg22565251 | 19 | 45,461,093 | intronic | *CLPTM1* | BIOS | 0.02 | 15 | 1.29 (1.18 ± 1.40) | **1.10 × 10^-8^** | 8.71 × 10^-6^ | rs75627662 | -47.52 |
| cg18788725 | 19 | 45,512,122 | intronic | *RELB* | FHS | 0.01 | 10 | 1.35 (1.17 ± 1.56) | 5.81 × 10^-5^ | 1.00 × 10^-2^ | rs75627662 | -98.55 |
| cg15224348 | 19 | 45,543,538 | exonic | *CLASRP* | FHS | 0.56 | 42 | 1.05 (1.03 ± 1.06) | 4.16 × 10^-7^ | 1.85 × 10^-4^ | rs75627662 | -129.96 |
| cg25394109 | 19 | 45,563,250 | intronic | *CLASRP* | FHS | 0.19 | 13 | 0.94 (0.91 ± 0.97) | 3.57 × 10^-4^ | 3.69 × 10^-2^ | rs75627662 | -149.67 |
| cg24166457 | 19 | 45,590,882 | ncRNA_intronic | *GEMIN7-AS1* | BIOS | 0.03 | 12 | 0.90 (0.85 ± 0.94) | 9.32 × 10^-6^ | 2.40 × 10^-3^ | rs75627662 | -177.31 |
| cg18383668 | 19 | 45,594,410 | UTR3 | *GEMIN7* | BIOS | 0.49 | 29 | 0.98 (0.97 ± 0.99) | 2.11 × 10^-6^ | 7.18 × 10^-4^ | rs75627662 | -180.83 |
| cg18383668 | 19 | 45,594,410 | UTR3 | *GEMIN7* | FHS | 0.34 | 31 | 0.95 (0.93 ± 0.98) | 6.64 × 10^-5^ | 1.11 × 10^-2^ | rs75627662 | -180.83 |
| cg08436089 | 19 | 45,594,493 | UTR3 | *GEMIN7* | BIOS | 0.18 | 28 | 0.96 (0.94 ± 0.97) | 1.05 × 10^-6^ | 4.06 × 10^-4^ | rs75627662 | -180.92 |
| cg08436089 | 19 | 45,594,493 | UTR3 | *GEMIN7* | FHS | 0.13 | 62 | 0.93 (0.90 ± 0.97) | 2.08 × 10^-4^ | 2.54 × 10^-2^ | rs75627662 | -180.92 |
| cg22717593 | 19 | 45,647,012 | intronic | *PPP1R37* | BIOS | 0.02 | 17 | 0.79 (0.75 ± 0.83) | **9.35 × 10^-20^** | 1.04 × 10^-15^ | rs75627662 | -233.44 |
| cg22717593 | 19 | 45,647,012 | intronic | *PPP1R37* | FHS | 0.01 | 8 | 0.65 (0.56 ± 0.75) | **5.72 × 10^-9^** | 5.01 × 10^-6^ | rs75627662 | -233.44 |
| cg02872930 | 19 | 45,649,752 | intronic | *PPP1R37* | BIOS | 0.10 | 55 | 1.10 (1.08 ± 1.12) | **4.28 × 10^-23^** | 9.40 × 10^-19^ | rs75627662 | -236.18 |
| cg10750934 | 19 | 45,652,975 | downstream | *NKPD1* | BIOS | 0.09 | 16 | 0.92 (0.90 ± 0.94) | **9.45 × 10^-13^** | 3.02 × 10^-9^ | rs75627662 | -239.40 |
| cg27052073 | 19 | 45,654,213 | UTR3 | *NKPD1* | BIOS | 0.14 | 29 | 0.94 (0.93 ± 0.96) | **5.90 × 10^-12^** | 1.44 × 10^-8^ | rs75627662 | -240.64 |
| cg01674009 | 19 | 45,655,294 | exonic | *NKPD1* | BIOS | 0.08 | 9 | 1.11 (1.08 ± 1.14) | **2.08 × 10^-12^** | 5.76 × 10^-9^ | rs75627662 | -241.72 |
| cg01674009 | 19 | 45,655,294 | exonic | *NKPD1* | FHS | 0.03 | 17 | 1.27 (1.17 ± 1.37) | **9.06 × 10^-9^** | 7.42 × 10^-6^ | rs75627662 | -241.72 |
| cg22062555 | 19 | 45,664,251 | upstream | *NKPD1* | BIOS | 0.31 | 37 | 1.03 (1.01 ± 1.04) | 7.92 × 10^-5^ | 1.28 × 10^-2^ | rs75627662 | -250.68 |
| cg27525032 | 19 | 45,664,609 | intergenic | *NKPD1, TRAPPC6A* | BIOS | 0.14 | 20 | 1.04 (1.02 ± 1.06) | 2.04 × 10^-4^ | 2.51 × 10^-2^ | rs75627662 | -251.03 |
| cg07928695 | 19 | 45,681,899 | upstream | *BLOC1S3, TRAPPC6A* | BIOS | 0.06 | 22 | 0.92 (0.90 ± 0.95) | **6.18 × 10^-8^** | 3.88 × 10^-5^ | rs75627662 | -268.32 |
| cg06479902 | 19 | 45,720,199 | intronic | *EXOC3L2* | BIOS | 0.08 | 33 | 1.14 (1.11 ± 1.17) | **4.52 × 10^-23^** | 9.40 × 10^-19^ | rs75627662 | -306.62 |
| cg25293896 | 19 | 45,720,949 | intronic | *NDUFAF6* | BIOS | 0.09 | 31 | 1.08 (1.05 ± 1.11) | **1.10 × 10^-7^** | 6.35 × 10^-5^ | rs75627662 | -307.37 |
| cg25556122 | 19 | 45,721,000 | intronic | *EXOC3L2* | FHS | 0.02 | 23 | 0.78 (0.70 ± 0.86) | 9.51 × 10^-7^ | 3.75 × 10^-4^ | rs75627662 | -307.42 |
| cg25556122 | 19 | 45,721,000 | intronic | *EXOC3L2* | BIOS | 0.07 | 26 | 1.07 (1.04 ± 1.11) | 4.48 × 10^-6^ | 1.32 × 10^-3^ | rs75627662 | -307.42 |
| cg00449767 | 19 | 45,737,603 | upstream | *EXOC3L2* | FHS | 0.04 | 28 | 0.89 (0.84 ± 0.95) | 3.35 × 10^-4^ | 3.55 × 10^-2^ | rs75627662 | -324.03 |
| cg01565314 | 19 | 45,737,610 | upstream | *EXOC3L2* | FHS | 0.03 | 19 | 0.88 (0.82 ± 0.94) | 3.87 × 10^-4^ | 3.88 × 10^-2^ | rs75627662 | -324.03 |
| cg12009516 | 19 | 45,737,880 | upstream | *EXOC3L2* | FHS | 0.02 | 27 | 0.80 (0.73 ± 0.87) | 3.77 × 10^-7^ | 1.70 × 10^-4^ | rs75627662 | -324.30 |
| cg21959090 | 19 | 45,744,435 | intergenic | *EXOC3L2, MARK4* | BIOS | 0.03 | 25 | 1.13 (1.09 ± 1.18) | **2.84 × 10^-9^** | 2.74 × 10^-6^ | rs75627662 | -330.86 |
| cg11376215 | 19 | 45,829,628 | intergenic | *CKM, KLC3* | BIOS | 0.01 | 8 | 0.86 (0.79 ± 0.93) | 1.46 × 10^-4^ | 1.99 × 10^-2^ | rs76320948 | 412.21 |
| cg13668450 | 19 | 45,844,596 | intronic | *KLC3* | BIOS | 0.02 | 64 | 0.92 (0.88 ± 0.95) | 1.44 × 10^-5^ | 3.48 × 10^-3^ | rs76320948 | 397.25 |
| cg04382643 | 19 | 45,849,853 | exonic | *KLC3* | FHS | 0.29 | 28 | 0.95 (0.93 ± 0.98) | 2.62 × 10^-4^ | 2.98 × 10^-2^ | rs76320948 | 391.99 |
| cg14586180 | 19 | 45,852,789 | exonic | *KLC3* | BIOS | 0.05 | 30 | 1.08 (1.05 ± 1.12) | 7.72 × 10^-7^ | 3.16 × 10^-4^ | rs76320948 | 389.05 |
| cg03117793 | 19 | 45,864,112 | intronic | *ERCC2* | BIOS | 0.02 | 20 | 0.90 (0.86 ± 0.95) | 1.13 × 10^-4^ | 1.64 × 10^-2^ | rs76320948 | 377.73 |
| cg03117793 | 19 | 45,864,112 | intronic | *ERCC2* | FHS | 0.02 | 19 | 0.81 (0.73 ± 0.91) | 4.68 × 10^-4^ | 4.45 × 10^-2^ | rs76320948 | 377.73 |
| cg24180227 | 19 | 45,870,100 | intronic | *ERCC2* | FHS | 0.02 | 7 | 0.81 (0.73 ± 0.90) | 1.35 × 10^-4^ | 1.87 × 10^-2^ | rs76320948 | 371.74 |
| cg14570121 | 19 | 45,874,153 | upstream | *ERCC2* | FHS | 0.01 | 12 | 1.35 (1.14 ± 1.59) | 5.42 × 10^-4^ | 4.92 × 10^-2^ | rs76320948 | 367.69 |
| cg23397216 | 19 | 45,885,922 | intronic | *GABBR1* | FHS | 0.01 | 23 | 0.73 (0.65 ± 0.83) | 1.31 × 10^-6^ | 4.91 × 10^-4^ | rs76320948 | 355.92 |
| cg23397216 | 19 | 45,885,922 | exonic | *PPP1R13L* | BIOS | 0.03 | 12 | 0.89 (0.85 ± 0.94) | 4.47 × 10^-6^ | 1.32 × 10^-3^ | rs76320948 | 355.92 |
| cg18741372 | 19 | 45,906,156 | intronic | *PPP1R13L* | FHS | 0.01 | 26 | 1.26 (1.13 ± 1.39) | 1.41 × 10^-5^ | 3.41 × 10^-3^ | rs76320948 | 335.69 |
| cg02881684 | 19 | 45,978,153 | UTR3 | *FOSB* | FHS | 0.01 | 5 | 1.35 (1.15 ± 1.60) | 2.80 × 10^-4^ | 3.10 × 10^-2^ | rs76320948 | 263.69 |
| cg07068062 | 19 | 46,002,528 | exonic | *VARS* | FHS | 0.02 | 10 | 0.72 (0.62 ± 0.83) | 1.83 × 10^-5^ | 4.18 × 10^-3^ | rs76320948 | 239.31 |
| cg02942825 | 19 | 46,185,348 | intergenic | *KLF16, ABHD17A* | FHS | 0.04 | 24 | 0.82 (0.76 ± 0.88) | **1.24 × 10^-7^** | 6.94 × 10^-5^ | rs76320948 | 56.49 |
| cg13095627 | 19 | 46,544,662 | upstream | *IGFL4* | FHS | 0.01 | 19 | 0.69 (0.59 ± 0.80) | 2.53 × 10^-6^ | 8.28 × 10^-4^ | rs76320948 | -302.82 |
| cg12683641 | 19 | 51,587,102 | intronic | *KLK14* | BIOS | 0.03 | 28 | 1.10 (1.04 ± 1.15) | 2.41 × 10^-4^ | 2.82 × 10^-2^ | rs3865444 | 140.86 |
| cg25263454 | 19 | 51,587,409 | UTR5 | *KLK14* | BIOS | 0.01 | 67 | 1.08 (1.04 ± 1.13) | 3.44 × 10^-4^ | 3.62 × 10^-2^ | rs3865444 | 140.55 |
| cg19279346 | 19 | 54,785,518 | upstream | *LILRB2, MIR4752* | BIOS | 0.16 | 38 | 0.94 (0.92 ± 0.96) | **5.04 × 10^-9^** | 4.48 × 10^-6^ | rs587709 | -14.07 |
| cg07280593 | 19 | 55,085,260 | UTR5 | *LILRA2* | BIOS | 0.08 | 40 | 1.05 (1.03 ± 1.07) | 1.67 × 10^-5^ | 3.90 × 10^-3^ | rs1761461 | -260.09 |
| cg07280593 | 19 | 55,085,260 | UTR5 | *LILRA2* | FHS | 0.02 | 12 | 1.29 (1.14 ± 1.46) | 4.38 × 10^-5^ | 8.17 × 10^-3^ | rs1761461 | -260.09 |
| cg14575739 | 19 | 55,098,069 | intronic | *LILRA2* | FHS | 0.06 | 9 | 0.87 (0.82 ± 0.93) | 1.86 × 10^-5^ | 4.24 × 10^-3^ | rs1761461 | -272.90 |
| cg14575739 | 19 | 55,098,069 | intronic | *LILRA2* | BIOS | 0.04 | 33 | 0.94 (0.91 ± 0.97) | 1.60 × 10^-4^ | 2.11 × 10^-2^ | rs1761461 | -272.90 |
| cg00387658 | 20 | 54,986,793 | upstream | *CASS4* | BIOS | 0.03 | 40 | 1.08 (1.05 ± 1.10) | **1.21 × 10^-10^** | 1.86 × 10^-7^ | rs6069737 | 8.91 |
| cg16210447 | 20 | 54,987,076 | upstream | *CASS4* | BIOS | 0.05 | 16 | 1.10 (1.07 ± 1.13) | **3.10 × 10^-11^** | 5.43 × 10^-8^ | rs6069737 | 8.62 |
| cg16210447 | 20 | 54,987,076 | upstream | *CASS4* | FHS | 0.02 | 12 | 1.41 (1.26 ± 1.57) | **1.39 × 10^-9^** | 1.50 × 10^-6^ | rs6069737 | 8.62 |
| cg24519157 | 20 | 54,987,662 | intronic | *CASS4* | FHS | 0.07 | 64 | 1.09 (1.05 ± 1.14) | 1.01 × 10^-4^ | 1.51 × 10^-2^ | rs6069737 | 8.04 |
| cg24519157 | 20 | 54,987,662 | intronic | *CASS4* | BIOS | 0.07 | 32 | 1.05 (1.02 ± 1.07) | 2.33 × 10^-4^ | 2.75 × 10^-2^ | rs6069737 | 8.04 |
| cg23037777 | 20 | 55,072,651 | exonic | *GCNT7* | BIOS | 0.11 | 12 | 1.05 (1.03 ± 1.07) | 4.25 × 10^-6^ | 1.27 × 10^-3^ | rs7274581 | -54.39 |
| cg23037777 | 20 | 55,072,651 | exonic | *GCNT7* | FHS | 0.17 | 26 | 1.08 (1.05 ± 1.12) | 4.36 × 10^-6^ | 1.29 × 10^-3^ | rs7274581 | -54.39 |
| cg01756902 | 20 | 62,340,073 | exonic | *ZGPAT* | BIOS | 0.02 | 21 | 1.09 (1.04 ± 1.14) | 5.49 × 10^-4^ | 4.95 × 10^-2^ | rs6742 | 34.37 |
| cg04956806 | 20 | 62,693,657 | intronic | *TCEA2* | BIOS | 0.25 | 34 | 0.98 (0.96 ± 0.99) | 4.19 × 10^-4^ | 4.14 × 10^-2^ | rs6742 | -319.22 |
| cg27370028 | 20 | 62,693,943 | intronic | *TCEA2* | BIOS | 0.34 | 69 | 0.98 (0.97 ± 0.99) | 2.48 × 10^-4^ | 2.88 × 10^-2^ | rs6742 | -319.50 |
| cg10494860 | 20 | 62,693,951 | intronic | *TCEA2* | BIOS | 0.41 | 50 | 0.98 (0.97 ± 0.99) | 4.64 × 10^-4^ | 4.42 × 10^-2^ | rs6742 | -319.51 |
| cg24348495 | 20 | 62,693,971 | intronic | *TCEA2* | BIOS | 0.40 | 60 | 0.98 (0.97 ± 0.99) | 4.03 × 10^-4^ | 4.01 × 10^-2^ | rs6742 | -319.53 |
| cg03946671 | 20 | 62,693,973 | intronic | *TCEA2* | BIOS | 0.46 | 53 | 0.98 (0.98 ± 0.99) | 5.07 × 10^-4^ | 4.70 × 10^-2^ | rs6742 | -319.53 |
| cg12176783 | 20 | 62,694,000 | intronic | *TCEA2* | BIOS | 0.38 | 49 | 0.98 (0.97 ± 0.99) | 2.27 × 10^-4^ | 2.70 × 10^-2^ | rs6742 | -319.56 |
| cg02197387 | 20 | 62,694,005 | intronic | *TCEA2* | FHS | 0.20 | 42 | 0.95 (0.93 ± 0.98) | 3.31 × 10^-4^ | 3.52 × 10^-2^ | rs6742 | -319.56 |
| cg02197387 | 20 | 62,694,005 | intronic | *TCEA2* | BIOS | 0.38 | 54 | 0.98 (0.97 ± 0.99) | 5.25 × 10^-4^ | 4.82 × 10^-2^ | rs6742 | -319.56 |
| cg05668372 | 20 | 62,734,251 | intergenic | *OPRL1, NPBWR2* | FHS | 0.01 | 9 | 1.45 (1.22 ± 1.72) | 2.45 × 10^-5^ | 5.24 × 10^-3^ | rs6742 | -359.81 |
| cg02991238 | 20 | 62,734,259 | intergenic | *OPRL1, NPBWR2* | FHS | 0.03 | 8 | 1.20 (1.09 ± 1.32) | 2.38 × 10^-4^ | 2.78 × 10^-2^ | rs6742 | -359.82 |

^1^BIOS: Biobank-based Integrative Omics Studies; Chr: chromosome; CI: confidence interval; CpG: CpG sites; FHS: Framingham Heart Study; kb: kilobase; ncRNA: noncoding RNA; OR: odds ratio per SD increase in genetically predicated DNA methylation level (continuous variable); SNP: single-nucleotide polymorphisms; UTR: untranslated region; ^2^R^2^: prediction performance; ^3^S-PrediXcan was used to estimate association ORs, 95% CIs and *P* value. All statistical tests were two-sided; ^4^associations with FDR-corrected *P* value < 0.05 considered significant; ^5^the closest risk SNPs identified in previous GWAS studies[1-4]; ^6^the distance from CpG site to risk SNP and “-” indicate the CpG site located downstream of the risk SNP.

**Table S3. Categories were annotated through ANNOVAR.**

| **Classification^1^** | **Identified CpG sites associated**  **with AD risk (N = 1,421)** | **Overall tested**  **CpG sites (N = 104,102)** | ***P* for difference** |
| --- | --- | --- | --- |
| intergenic | 249 (17.52%) | 25,569 (24.56%) | 1.07 × 10^-9^ |
| exonic | 168 (11.82%) | 7,838 (7.53%) | 1.74 × 10^-9^ |
| ncRNA intronic | 84 5.91%) | 3,939 (3.78%) | 4.31 × 10^-5^ |
| downstream | 17 (1.20%) | 614 (0.59%) | 5.57 × 10^-3^ |
| ncRNA exonic | 49 (3.45%) | 2,497 (2.40%) | 0.01 |
| splicing | 2 (0.14%) | 20 (0.02%) | 0.03 |
| UTR5 | 67 (4.71%) | 5,784 (5.56%) | 0.19 |
| intronic | 508 (35.75%) | 38,481 (36.96%) | 0.36 |
| UTR3 | 61 (4.29%) | 3,978 (3.82%) | 0.40 |
| upstream | 200 (14.07%) | 14,303 (13.74%) | 0.74 |
| upstream, downstream | 16 (1.13%) | 1,058 (1.02%) | 0.78 |
| UTR5, UTR3 | 0 (0.00%) | 19 (0.02%) | 1 |
| ncRNA splicing | 0 (0.00%) | 2 (0.00%) | 1 |

^1^Substantial inflations of “exonic” and “ncRNA intronic”, and substantial decreased proportion of “intergenic” are found for 1,421 AD-associated CpG sites compared with the overall tested 108, 432 CpG sites; chi-square tests (two-sided); *P* value less than 0.05/13 = 3.85 × 10^-3^ as difference significant.

**Table S4. Correlation between DNA methylation of identified associated CpGs and expression of their flanking genes.**

| **CpG site** | **Gene** | **Correlation coefficient** | ***P* value** | ***P* value after FDR correction** |
| --- | --- | --- | --- | --- |
| cg00614959 | *PCGF3* | -0.29 | 2.20 × 10^-16^ | 1.90 × 10^-14^ |
| cg10864200 | *PCGF3* | -0.31 | 2.20 × 10^-16^ | 1.90 × 10^-14^ |
| cg12548824 | *MTUS1* | -0.29 | 2.20 × 10^-16^ | 1.90 × 10^-14^ |
| cg16837973 | *LOC100130950* | -0.22 | 2.20 × 10^-16^ | 1.90 × 10^-14^ |
| cg17397159 | *PCGF3* | -0.23 | 2.20 × 10^-16^ | 1.90 × 10^-14^ |
| cg17588003 | *LOC100130950* | -0.23 | 2.20 × 10^-16^ | 1.90 × 10^-14^ |
| cg17830204 | *PVRIG* | -0.25 | 2.20 × 10^-16^ | 1.90 × 10^-14^ |
| cg20172563 | *CD2AP* | 0.23 | 2.20 × 10^-16^ | 1.90 × 10^-14^ |
| cg21337881 | *LOC100130950* | -0.23 | 2.20 × 10^-16^ | 1.90 × 10^-14^ |
| cg23963071 | *SERPINB9* | -0.24 | 2.20 × 10^-16^ | 1.90 × 10^-14^ |
| cg25647583 | *FES* | -0.26 | 2.20 × 10^-16^ | 1.90 × 10^-14^ |
| cg25712567 | *PCGF3* | -0.30 | 2.20 × 10^-16^ | 1.90 × 10^-14^ |
| cg05549575 | *LOC100130950* | -0.22 | 5.76 × 10^-16^ | 4.60 × 10^-14^ |
| cg03950000 | *SLAMF1* | -0.21 | 2.64 × 10^-15^ | 1.96 × 10^-13^ |
| cg00797651 | *CD55* | 0.21 | 3.02 × 10^-15^ | 2.09 × 10^-13^ |
| cg23077453 | *PCGF3* | -0.21 | 8.82 × 10^-15^ | 5.72 × 10^-13^ |
| cg01993952 | *MTUS1* | -0.20 | 3.62 × 10^-14^ | 2.21 × 10^-12^ |
| cg02152968 | *CD55* | 0.20 | 1.77 × 10^-13^ | 9.89 × 10^-12^ |
| cg26426488 | *SLC12A9* | -0.20 | 1.81 × 10^-13^ | 9.89 × 10^-12^ |
| cg25630380 | *SH2D2A* | -0.20 | 2.02 × 10^-13^ | 1.05 × 10^-11^ |
| cg06895664 | *SAMD12* | 0.20 | 3.37 × 10^-13^ | 1.67 × 10^-11^ |
| cg20742981 | *LIMK2* | -0.19 | 6.84 × 10^-13^ | 3.23 × 10^-11^ |
| cg11015549 | *CNN2* | -0.19 | 8.04 × 10^-13^ | 3.63 × 10^-11^ |
| cg23659289 | *ARHGAP27* | -0.19 | 9.89 × 10^-13^ | 4.28 × 10^-11^ |
| cg14709253 | *MTUS1* | 0.19 | 1.27 × 10^-12^ | 5.27 × 10^-11^ |
| cg13702222 | *MBNL1* | -0.19 | 2.26 × 10^-12^ | 9.02 × 10^-11^ |
| cg24590430 | *RRP12* | -0.19 | 2.36 × 10^-12^ | 9.07 × 10^-11^ |
| cg14575739 | *LILRA2* | 0.19 | 3.36 × 10^-12^ | 1.25 × 10^-10^ |
| cg06881914 | *MS4A6A* | -0.19 | 4.04 × 10^-12^ | 1.43 × 10^-10^ |
| cg04266202 | *MPO* | -0.19 | 4.12 × 10^-12^ | 1.43 × 10^-10^ |
| cg09757525 | *BACH2* | -0.18 | 9.16 × 10^-12^ | 3.07 × 10^-10^ |
| cg02060717 | *RHOH* | -0.18 | 2.25 × 10^-11^ | 7.30 × 10^-10^ |
| cg02647874 | *SPI1* | -0.18 | 5.17 × 10^-11^ | 1.63 × 10^-9^ |
| cg01578585 | *SLC43A2* | -0.17 | 7.52 × 10^-11^ | 2.30 × 10^-9^ |
| cg11998307 | *SCARF1* | -0.17 | 7.89 × 10^-11^ | 2.34 × 10^-9^ |
| cg15595502 | *CNIH4* | -0.17 | 1.69 × 10^-10^ | 4.87 × 10^-9^ |
| cg07697227 | *PCGF3* | -0.17 | 2.62 × 10^-10^ | 7.35 × 10^-9^ |
| cg19279346 | *LILRB2* | 0.17 | 2.83 × 10^-10^ | 7.73 × 10^-9^ |
| cg08843064 | *TRIB1* | 0.17 | 3.36 × 10^-10^ | 8.94 × 10^-9^ |
| cg03725573 | *ZBTB16* | -0.17 | 4.08 × 10^-10^ | 1.06 × 10^-8^ |
| cg05931265 | *UBASH3B* | -0.17 | 7.23 × 10^-10^ | 1.83 × 10^-8^ |
| cg05200313 | *SLC24A4* | -0.16 | 9.56 × 10^-10^ | 2.36 × 10^-8^ |
| cg16281322 | *ARHGAP27* | -0.16 | 1.14 × 10^-9^ | 2.71 × 10^-8^ |
| cg09106231 | *CD55* | -0.16 | 1.15 × 10^-9^ | 2.71 × 10^-8^ |
| cg21110645 | *CR1* | -0.16 | 1.31 × 10^-9^ | 3.02 × 10^-8^ |
| cg15504662 | *LIMK2* | -0.16 | 1.93 × 10^-9^ | 4.36 × 10^-8^ |
| cg18989491 | *PFKFB3* | -0.16 | 3.53 × 10^-9^ | 7.43 × 10^-8^ |
| cg05526498 | *SPAG9* | -0.16 | 3.54 × 10^-9^ | 7.43 × 10^-8^ |
| cg15863539 | *SREBF1* | -0.16 | 3.57 × 10^-9^ | 7.43 × 10^-8^ |
| cg16915659 | *CISD1* | 0.16 | 3.58 × 10^-9^ | 7.43 × 10^-8^ |
| cg19048010 | *SSH2* | 0.16 | 5.17 × 10^-9^ | 1.05 × 10^-7^ |
| cg01904978 | *EED* | 0.15 | 1.20 × 10^-8^ | 2.40 × 10^-7^ |
| cg00249205 | *TMEM106B* | 0.15 | 1.86 × 10^-8^ | 3.64 × 10^-7^ |
| cg02130027 | *CD2AP* | 0.15 | 2.50 × 10^-8^ | 4.81 × 10^-7^ |
| cg16210447 | *CASS4* | -0.15 | 2.79 × 10^-8^ | 5.27 × 10^-7^ |
| cg02335376 | *MAPK3* | -0.15 | 3.36 × 10^-8^ | 6.23 × 10^-7^ |
| cg12487901 | *PCDHGC5* | 0.15 | 3.94 × 10^-8^ | 7.17 × 10^-7^ |
| cg25961567 | *NCK2* | -0.15 | 4.03 × 10^-8^ | 7.21 × 10^-7^ |
| cg25708777 | *ARHGAP27* | -0.15 | 4.11 × 10^-8^ | 7.23 × 10^-7^ |
| cg26343298 | *TP53INP1* | -0.15 | 7.07 × 10^-8^ | 1.22 × 10^-6^ |
| cg14021523 | *SLC24A4* | -0.14 | 7.61 × 10^-8^ | 1.29 × 10^-6^ |
| cg05360477 | *TRIB2* | 0.14 | 1.23 × 10^-7^ | 2.06 × 10^-6^ |
| cg24281668 | *TPCN1* | -0.14 | 1.27 × 10^-7^ | 2.09 × 10^-6^ |
| cg07180834 | *EED* | -0.14 | 1.79 × 10^-7^ | 2.90 × 10^-6^ |
| cg08354889 | *DEPDC7* | -0.14 | 1.83 × 10^-7^ | 2.92 × 10^-6^ |
| cg01120308 | *CHRNE* | -0.14 | 1.99 × 10^-7^ | 3.13 × 10^-6^ |
| cg16767590 | *PCGF3* | -0.14 | 4.40 × 10^-7^ | 6.82 × 10^-6^ |
| cg08901339 | *BLNK* | -0.13 | 6.31 × 10^-7^ | 9.63 × 10^-6^ |
| cg25670583 | *PCGF3* | -0.13 | 8.12 × 10^-7^ | 1.22 × 10^-5^ |
| cg07713946 | *LIMK2* | 0.13 | 8.98 × 10^-7^ | 1.33 × 10^-5^ |
| cg17769836 | *APOC4* | 0.13 | 9.97 × 10^-7^ | 1.46 × 10^-5^ |
| cg05659526 | *FCER1G* | -0.13 | 1.69 × 10^-6^ | 2.44 × 10^-5^ |
| cg00436254 | *BIN1* | 0.13 | 1.82 × 10^-6^ | 2.59 × 10^-5^ |
| cg16398362 | *RAD51C* | -0.13 | 2.84 × 10^-6^ | 3.96 × 10^-5^ |
| cg18410271 | *ARHGAP27* | -0.13 | 2.86 × 10^-6^ | 3.96 × 10^-5^ |
| cg27508378 | *CLNK* | -0.13 | 3.00 × 10^-6^ | 4.10 × 10^-5^ |
| cg16178415 | *TSPAN14* | -0.13 | 3.04 × 10^-6^ | 4.10 × 10^-5^ |
| cg15934958 | *LRRFIP2* | -0.12 | 4.33 × 10^-6^ | 5.76 × 10^-5^ |
| cg14012546 | *INPP5D* | -0.12 | 4.61 × 10^-6^ | 6.06 × 10^-5^ |
| cg11107966 | *SLC24A4* | -0.12 | 5.95 × 10^-6^ | 7.72 × 10^-5^ |
| cg13485809 | *PCGF3* | -0.12 | 7.34 × 10^-6^ | 9.41 × 10^-5^ |
| cg04692506 | *SPI1* | 0.12 | 8.77 × 10^-6^ | 1.11 × 10^-4^ |
| cg20196966 | *CD2AP* | 0.12 | 1.19 × 10^-5^ | 1.49 × 10^-4^ |
| cg17763743 | *THUMPD3* | -0.12 | 1.29 × 10^-5^ | 1.59 × 10^-4^ |
| cg02010481 | *JAZF1* | 0.12 | 1.64 × 10^-5^ | 2.00 × 10^-4^ |
| cg05208056 | *TBX6* | 0.12 | 1.91 × 10^-5^ | 2.31 × 10^-4^ |
| cg19417129 | *CLPTM1* | -0.11 | 2.03 × 10^-5^ | 2.42 × 10^-4^ |
| cg09106231 | *DGKQ* | 0.11 | 2.39 × 10^-5^ | 2.82 × 10^-4^ |
| cg26140475 | *TRIB1* | -0.11 | 2.53 × 10^-5^ | 2.95 × 10^-4^ |
| cg03984055 | *BCKDK* | -0.11 | 2.65 × 10^-5^ | 3.06 × 10^-4^ |
| cg16130019 | *JAZF1* | 0.11 | 2.83 × 10^-5^ | 3.23 × 10^-4^ |
| cg05868564 | *GRAMD1B* | -0.11 | 2.87 × 10^-5^ | 3.24 × 10^-4^ |
| cg21666367 | *EPHX2* | 0.11 | 3.01 × 10^-5^ | 3.36 × 10^-4^ |
| cg22808351 | *SH2D2A* | -0.11 | 3.17 × 10^-5^ | 3.50 × 10^-4^ |
| cg01814191 | *DUSP6* | -0.11 | 3.69 × 10^-5^ | 4.03 × 10^-4^ |
| cg15516558 | *DEDD* | -0.11 | 4.59 × 10^-5^ | 4.96 × 10^-4^ |
| cg27353824 | *APOC4* | 0.11 | 4.73 × 10^-5^ | 5.06 × 10^-4^ |
| cg08766099 | *PCGF3* | -0.11 | 5.83 × 10^-5^ | 6.18 × 10^-4^ |
| cg08464513 | *MAPK3* | -0.11 | 8.06 × 10^-5^ | 8.45 × 10^-4^ |
| cg10169327 | *APOC4* | 0.11 | 8.79 × 10^-5^ | 9.12 × 10^-4^ |
| cg06284479 | *LRRFIP2* | -0.11 | 8.97 × 10^-5^ | 9.22 × 10^-4^ |
| cg20716064 | *USP6NL* | -0.11 | 9.92 × 10^-5^ | 1.01 × 10^-3^ |
| cg00274203 | *COQ10A* | 0.10 | 1.15 × 10^-4^ | 1.16 × 10^-3^ |
| cg11314684 | *AKT3* | -0.10 | 1.27 × 10^-4^ | 1.27 × 10^-3^ |
| cg04203702 | *CLNK* | -0.10 | 1.50 × 10^-4^ | 1.48 × 10^-3^ |
| cg00685795 | *COASY* | 0.10 | 1.60 × 10^-4^ | 1.56 × 10^-3^ |
| cg05139152 | *RASAL1* | 0.10 | 1.68 × 10^-4^ | 1.63 × 10^-3^ |
| cg04401876 | *APOC4* | 0.10 | 1.80 × 10^-4^ | 1.73 × 10^-3^ |
| cg09070378 | *NDUFS2* | -0.10 | 1.86 × 10^-4^ | 1.77 × 10^-3^ |
| cg15742858 | *CCDC88C* | 0.10 | 1.96 × 10^-4^ | 1.85 × 10^-3^ |
| cg04353769 | *MS4A6A* | -0.10 | 2.07 × 10^-4^ | 1.93 × 10^-3^ |
| cg06189038 | *GAL3ST4* | -0.10 | 2.12 × 10^-4^ | 1.96 × 10^-3^ |
| cg26306976 | *CPSF3* | 0.10 | 2.24 × 10^-4^ | 2.05 × 10^-3^ |
| cg15320596 | *CCDC6* | 0.10 | 2.62 × 10^-4^ | 2.37 × 10^-3^ |
| cg26621780 | *RASAL1* | 0.00 | 2.62 × 10^-4^ | 2.37 × 10^-3^ |
| cg19590598 | *BIN1* | 0.10 | 3.06 × 10^-4^ | 2.74 × 10^-3^ |
| cg14012546 | *GPX6* | -0.10 | 3.13 × 10^-4^ | 2.78 × 10^-3^ |
| cg24794833 | *EPHX2* | -0.10 | 3.16 × 10^-4^ | 2.78 × 10^-3^ |
| cg04422903 | *CCDC88B* | -0.10 | 3.67 × 10^-4^ | 3.20 × 10^-3^ |
| cg26709300 | *YPEL3* | 0.10 | 3.83 × 10^-4^ | 3.29 × 10^-3^ |
| cg13027184 | *SERPINF2* | 0.10 | 3.84 × 10^-4^ | 3.29 × 10^-3^ |
| cg24590430 | *FRAT2* | -0.10 | 3.89 × 10^-4^ | 3.31 × 10^-3^ |
| cg00091760 | *PLEKHA1* | -0.10 | 4.03 × 10^-4^ | 3.40 × 10^-3^ |
| cg16754522 | *PCGF3* | 0.10 | 4.08 × 10^-4^ | 3.42 × 10^-3^ |
| cg22376361 | *BIN1* | 0.10 | 4.23 × 10^-4^ | 3.51 × 10^-3^ |
| cg18368669 | *PCGF3* | 0.10 | 4.41 × 10^-4^ | 3.63 × 10^-3^ |
| cg06321596 | *XYLT1* | -0.09 | 4.65 × 10^-4^ | 3.80 × 10^-3^ |
| cg24909660 | *NR1H3* | -0.09 | 4.99 × 10^-4^ | 4.05 × 10^-3^ |
| cg07862129 | *APOC4* | 0.09 | 5.05 × 10^-4^ | 4.06 × 10^-3^ |
| cg17960164 | *PLEC* | -0.09 | 5.39 × 10^-4^ | 4.27 × 10^-3^ |
| cg04359840 | *XYLT1* | -0.09 | 5.39 × 10^-4^ | 4.27 × 10^-3^ |
| cg07404961 | *STX4* | 0.09 | 5.47 × 10^-4^ | 4.28 × 10^-3^ |
| cg25647583 | *FURIN* | -0.09 | 5.48 × 10^-4^ | 4.28 × 10^-3^ |
| cg19153828 | *BIN1* | 0.09 | 5.57 × 10^-4^ | 4.31 × 10^-3^ |
| cg20135002 | *MTCH2* | 0.09 | 5.61 × 10^-4^ | 4.31 × 10^-3^ |
| cg01578585 | *SCARF1* | -0.09 | 5.75 × 10^-4^ | 4.39 × 10^-3^ |
| cg13731523 | *GRK4* | -0.09 | 6.10 × 10^-4^ | 4.62 × 10^-3^ |
| cg04822495 | *PLXNC1* | -0.09 | 6.85 × 10^-4^ | 5.15 × 10^-3^ |
| cg05825950 | *CR1* | -0.09 | 7.27 × 10^-4^ | 5.43 × 10^-3^ |
| cg08347373 | *RASAL1* | -0.09 | 8.84 × 10^-4^ | 6.56 × 10^-3^ |
| cg17717010 | *SPAG9* | -0.09 | 9.10 × 10^-4^ | 6.70 × 10^-3^ |
| cg14714629 | *TRIM4* | 0.09 | 9.21 × 10^-4^ | 6.73 × 10^-3^ |
| cg25017250 | *APOC4* | 0.09 | 9.55 × 10^-4^ | 6.94 × 10^-3^ |
| cg03399927 | *PLCB3* | 0.09 | 1.06 × 10^-3^ | 7.61 × 10^-3^ |
| cg01496416 | *PVR* | -0.09 | 1.10 × 10^-3^ | 7.87 × 10^-3^ |
| cg25748958 | *THADA* | 0.09 | 1.15 × 10^-3^ | 8.19 × 10^-3^ |
| cg07067577 | *ARHGAP27* | -0.09 | 1.20 × 10^-3^ | 8.45 × 10^-3^ |
| cg02335376 | *GDPD3* | -0.09 | 1.35 × 10^-3^ | 9.45 × 10^-3^ |
| cg26471390 | *ARHGAP27* | -0.09 | 1.38 × 10^-3^ | 9.63 × 10^-3^ |
| cg25595834 | *SLTM* | 0.09 | 1.46 × 10^-3^ | 1.01 × 10^-2^ |
| cg00715050 | *ARHGAP27* | -0.08 | 1.77 × 10^-3^ | 1.22 × 10^-2^ |
| cg22500518 | *TMEM184A* | -0.08 | 1.79 × 10^-3^ | 1.22 × 10^-2^ |
| cg05676562 | *TBX6* | -0.08 | 1.83 × 10^-3^ | 1.24 × 10^-2^ |
| cg22099723 | *EPHX2* | 0.08 | 1.89 × 10^-3^ | 1.28 × 10^-2^ |
| cg17740822 | *DUSP6* | -0.08 | 1.94 × 10^-3^ | 1.30 × 10^-2^ |
| cg07368061 | *MAPT* | 0.08 | 2.03 × 10^-3^ | 1.35 × 10^-2^ |
| cg06233904 | *STX4* | -0.08 | 2.15 × 10^-3^ | 1.42 × 10^-2^ |
| cg05457903 | *PARP10* | 0.08 | 2.26 × 10^-3^ | 1.49 × 10^-2^ |
| cg25941154 | *PPP1R13L* | 0.08 | 2.30 × 10^-3^ | 1.50 × 10^-2^ |
| cg06148264 | *SERPINB1* | -0.08 | 2.37 × 10^-3^ | 1.53 × 10^-2^ |
| cg11588975 | *USP6* | 0.08 | 2.37 × 10^-3^ | 1.53 × 10^-2^ |
| cg04401876 | *EPHX2* | 0.08 | 2.67 × 10^-3^ | 1.71 × 10^-2^ |
| cg07675031 | *RNF43* | 0.08 | 2.79 × 10^-3^ | 1.78 × 10^-2^ |
| cg21666367 | *CLU* | 0.08 | 2.87 × 10^-3^ | 1.82 × 10^-2^ |
| cg22985146 | *LRRFIP2* | 0.08 | 3.07 × 10^-3^ | 1.93 × 10^-2^ |
| cg04434147 | *CBLC* | -0.08 | 3.23 × 10^-3^ | 2.02 × 10^-2^ |
| cg17705041 | *PCGF3* | -0.08 | 3.38 × 10^-3^ | 2.10 × 10^-2^ |
| cg23207054 | *CSF3* | -0.08 | 3.51 × 10^-3^ | 2.17 × 10^-2^ |
| cg19800032 | *TMEM106B* | -0.08 | 3.57 × 10^-3^ | 2.19 × 10^-2^ |
| cg11291798 | *EPHA1* | -0.08 | 3.69 × 10^-3^ | 2.25 × 10^-2^ |
| cg02823457 | *NGFR* | 0.08 | 3.76 × 10^-3^ | 2.28 × 10^-2^ |
| cg01795660 | *DOC2A* | -0.08 | 3.98 × 10^-3^ | 2.39 × 10^-2^ |
| cg00480298 | *MAPT* | -0.08 | 3.98 × 10^-3^ | 2.39 × 10^-2^ |
| cg18465082 | *MXD3* | -0.08 | 4.08 × 10^-3^ | 2.42 × 10^-2^ |
| cg23858360 | *TSPAN14* | 0.08 | 4.09 × 10^-3^ | 2.42 × 10^-2^ |
| cg26268276 | *DEPDC7* | -0.08 | 4.18 × 10^-3^ | 2.46 × 10^-2^ |
| cg02881684 | *FOSB* | 0.08 | 4.19 × 10^-3^ | 2.46 × 10^-2^ |
| cg22084182 | *JAZF1* | 0.08 | 4.63 × 10^-3^ | 2.70 × 10^-2^ |
| cg01784819 | *INTS1* | 0.08 | 4.95 × 10^-3^ | 2.86 × 10^-2^ |
| cg18989491 | *RBM17* | 0.08 | 4.96 × 10^-3^ | 2.86 × 10^-2^ |
| cg11582226 | *FBXL3* | -0.08 | 5.00 × 10^-3^ | 2.87 × 10^-2^ |
| cg05668372 | *OPRL1* | -0.08 | 5.06 × 10^-3^ | 2.89 × 10^-2^ |
| cg23599110 | *TRIB2* | 0.08 | 5.31 × 10^-3^ | 3.01 × 10^-2^ |
| cg09552399 | *ZNF646* | -0.08 | 5.41 × 10^-3^ | 3.05 × 10^-2^ |
| cg18106548 | *ZNF221* | -0.08 | 5.44 × 10^-3^ | 3.05 × 10^-2^ |
| cg15140703 | *STAG3* | -0.08 | 5.53 × 10^-3^ | 3.09 × 10^-2^ |
| cg01097611 | *TRIM38* | -0.07 | 5.70 × 10^-3^ | 3.16 × 10^-2^ |
| cg07178064 | *UFC1* | 0.07 | 5.80 × 10^-3^ | 3.20 × 10^-2^ |
| cg10516117 | *SERPINF2* | -0.07 | 6.18 × 10^-3^ | 3.39 × 10^-2^ |
| cg12688576 | *RNF144B* | -0.07 | 6.43 × 10^-3^ | 3.51 × 10^-2^ |
| cg07634191 | *SCARA5* | 0.07 | 6.53 × 10^-3^ | 3.55 × 10^-2^ |
| cg18477569 | *BACH2* | 0.07 | 6.78 × 10^-3^ | 3.67 × 10^-2^ |
| cg16516316 | *MTCH2* | -0.07 | 7.11 × 10^-3^ | 3.82 × 10^-2^ |
| cg22684041 | *SCAMP4* | -0.07 | 7.22 × 10^-3^ | 3.86 × 10^-2^ |
| cg03070550 | *HTRA1* | -0.07 | 7.32 × 10^-3^ | 3.90 × 10^-2^ |
| cg19792908 | *ZNF221* | -0.07 | 7.50 × 10^-3^ | 3.97 × 10^-2^ |
| cg16612511 | *KIF1C* | 0.07 | 7.79 × 10^-3^ | 4.10 × 10^-2^ |
| cg08563189 | *BIN1* | 0.07 | 8.20 × 10^-3^ | 4.30 × 10^-2^ |
| cg24072947 | *NCK1* | 0.07 | 8.27 × 10^-3^ | 4.31 × 10^-2^ |
| cg15095913 | *MAFK* | 0.07 | 8.39 × 10^-3^ | 4.35 × 10^-2^ |
| cg12682931 | *RRAGD* | -0.07 | 8.57 × 10^-3^ | 4.41 × 10^-2^ |
| cg07356342 | *NDUFS2* | -0.07 | 8.57 × 10^-3^ | 4.41 × 10^-2^ |
| cg03711944 | *SPI1* | -0.07 | 8.65 × 10^-3^ | 4.42 × 10^-2^ |
| cg06736138 | *APOC4* | 0.07 | 9.28 × 10^-3^ | 4.72 × 10^-2^ |
| cg01674009 | *NKPD1* | -0.07 | 9.38 × 10^-3^ | 4.75 × 10^-2^ |
| cg14367014 | *ICA1L* | 0.07 | 0.01 | 0.05 |
| cg00810986 | *PCGF3* | -0.07 | 0.01 | 0.05 |
| cg14066757 | *KCNN4* | -0.07 | 0.01 | 0.05 |
| cg25208677 | *SLC35D1* | -0.07 | 0.01 | 0.06 |
| cg09246103 | *RERE* | -0.07 | 0.01 | 0.06 |
| cg05962511 | *SEMA4G* | 0.07 | 0.01 | 0.06 |
| cg13496662 | *APOE* | -0.07 | 0.01 | 0.06 |
| cg18512352 | *C1QTNF4* | -0.07 | 0.01 | 0.06 |
| cg09070378 | *YPEL3* | -0.07 | 0.01 | 0.06 |
| cg25746394 | *APOC4* | -0.07 | 0.01 | 0.07 |
| cg06643284 | *BLNK* | 0.07 | 0.01 | 0.07 |
| cg13432737 | *IKZF3* | 0.07 | 0.01 | 0.07 |
| cg00411097 | *TMEM184A* | 0.07 | 0.01 | 0.07 |
| cg19668951 | *SLC39A13* | 0.07 | 0.02 | 0.07 |
| cg16906765 | *PPCDC* | 0.07 | 0.02 | 0.07 |
| cg07280593 | *LILRA2* | -0.07 | 0.02 | 0.07 |
| cg07397033 | *CSNK1G2* | 0.07 | 0.02 | 0.07 |
| cg04577268 | *ZNF221* | -0.07 | 0.02 | 0.07 |
| cg23558213 | *SLC25A17* | 0.07 | 0.02 | 0.07 |
| cg27310024 | *IQGAP1* | -0.07 | 0.02 | 0.07 |
| cg02333467 | *POP7* | 0.07 | 0.02 | 0.07 |
| cg17448481 | *EXT1* | 0.06 | 0.02 | 0.08 |
| cg00353353 | *PTPRN2* | 0.06 | 0.02 | 0.08 |
| cg02333467 | *GABBR1* | -0.06 | 0.02 | 0.08 |
| cg07461837 | *PYCARD* | -0.06 | 0.02 | 0.08 |
| cg14301190 | *NCK2* | 0.06 | 0.02 | 0.08 |
| cg03898962 | *CDK5R1* | -0.06 | 0.02 | 0.08 |
| cg14105019 | *SUSD3* | 0.06 | 0.02 | 0.08 |
| cg10780367 | *TSPAN14* | 0.06 | 0.02 | 0.08 |
| cg24519157 | *CASS4* | 0.06 | 0.02 | 0.08 |
| cg19286986 | *GPR162* | 0.06 | 0.02 | 0.08 |
| cg26831220 | *IL34* | -0.06 | 0.02 | 0.08 |
| cg19811934 | *SLC24A4* | -0.06 | 0.02 | 0.08 |
| cg07501827 | *LYPD5* | 0.06 | 0.02 | 0.09 |
| cg21593001 | *DTX1* | -0.06 | 0.02 | 0.09 |
| cg20728490 | *DNTT* | 0.06 | 0.02 | 0.09 |
| cg19687457 | *KLF16* | -0.06 | 0.02 | 0.09 |
| cg16448702 | *INPP5D* | -0.06 | 0.02 | 0.09 |
| cg12575674 | *SH2D4B* | 0.06 | 0.02 | 0.09 |
| cg22977317 | *SERPINB1* | -0.06 | 0.02 | 0.10 |
| cg18090197 | *GPC2* | 0.06 | 0.02 | 0.10 |
| cg08203460 | *STX1B* | 0.06 | 0.02 | 0.10 |
| cg21666367 | *APOC4* | 0.06 | 0.02 | 0.10 |
| cg04156293 | *EXOC2* | 0.06 | 0.02 | 0.10 |
| cg10233654 | *DUSP6* | -0.06 | 0.02 | 0.10 |
| cg22876739 | *TET1* | 0.06 | 0.02 | 0.10 |
| cg07465899 | *PCGF3* | 0.06 | 0.03 | 0.10 |
| cg00048759 | *STAG3* | -0.06 | 0.03 | 0.11 |
| cg00961792 | *PIGR* | 0.06 | 0.03 | 0.11 |
| cg10421029 | *FBXL19* | -0.06 | 0.03 | 0.11 |
| cg19198483 | *C1QTNF4* | 0.06 | 0.03 | 0.11 |
| cg09245872 | *INO80E* | -0.06 | 0.03 | 0.11 |
| cg00736406 | *GGA2* | -0.06 | 0.03 | 0.11 |
| cg07052063 | *MMS19* | -0.06 | 0.03 | 0.11 |
| cg26325430 | *KL* | -0.06 | 0.03 | 0.11 |
| cg16906765 | *C15orf39* | 0.06 | 0.03 | 0.11 |
| cg15602972 | *DACT3* | -0.06 | 0.03 | 0.12 |
| cg00688810 | *FXR2* | 0.06 | 0.03 | 0.12 |
| cg23595451 | *RNF212* | -0.06 | 0.03 | 0.12 |
| cg18090197 | *GIPR* | 0.06 | 0.03 | 0.12 |
| cg02942825 | *KLF16* | -0.06 | 0.03 | 0.12 |
| cg03836283 | *MAPT* | 0.06 | 0.03 | 0.13 |
| cg06420512 | *SCNN1B* | -0.06 | 0.03 | 0.13 |
| cg14894245 | *GPX4* | 0.06 | 0.03 | 0.13 |
| cg05659526 | *NDUFS2* | -0.06 | 0.03 | 0.13 |
| cg03157040 | *KLF16* | -0.06 | 0.03 | 0.13 |
| cg10084644 | *STAG3* | -0.06 | 0.03 | 0.13 |
| cg16718999 | *CCDC88B* | -0.06 | 0.03 | 0.13 |
| cg27031099 | *TRIB1* | -0.06 | 0.03 | 0.13 |
| cg21167157 | *HIP1R* | -0.06 | 0.04 | 0.13 |
| cg16477091 | *PPM1E* | 0.06 | 0.04 | 0.13 |
| cg08441314 | *C1QTNF4* | -0.06 | 0.04 | 0.14 |
| cg13557167 | *SLC25A11* | -0.06 | 0.04 | 0.14 |
| cg15977816 | *KCNN4* | -0.06 | 0.04 | 0.14 |
| cg15125438 | *TPCN1* | -0.06 | 0.04 | 0.15 |
| cg04833845 | *KCNN4* | -0.06 | 0.04 | 0.15 |
| cg08217716 | *PAX2* | 0.06 | 0.04 | 0.15 |
| cg02879656 | *LDB3* | 0.06 | 0.04 | 0.15 |
| cg11321190 | *STX1B* | 0.06 | 0.04 | 0.15 |
| cg07026910 | *INPP5D* | -0.06 | 0.04 | 0.15 |
| cg21238440 | *TMEM106B* | -0.06 | 0.04 | 0.15 |
| cg19548313 | *ABCA7* | -0.06 | 0.04 | 0.15 |
| cg15443166 | *TMEM106B* | -0.06 | 0.04 | 0.15 |
| cg01784819 | *MAFK* | 0.06 | 0.04 | 0.15 |
| cg13536080 | *PICALM* | -0.05 | 0.04 | 0.15 |
| cg17705041 | *GPX5* | -0.05 | 0.04 | 0.15 |
| cg24949488 | *DNTT* | 0.05 | 0.04 | 0.15 |
| cg02114696 | *USP6* | 0.05 | 0.05 | 0.17 |
| cg20069407 | *SORL1* | -0.05 | 0.05 | 0.17 |
| cg03935956 | *GGA2* | 0.05 | 0.05 | 0.17 |
| cg27106909 | *YPEL3* | 0.05 | 0.05 | 0.17 |
| cg03055520 | *SORL1* | -0.05 | 0.05 | 0.17 |
| cg12503394 | *TMEM184A* | 0.05 | 0.05 | 0.17 |
| cg14402224 | *DDC* | 0.05 | 0.05 | 0.17 |
| cg26467631 | *BIN1* | -0.05 | 0.05 | 0.17 |
| cg18200150 | *MYO1D* | 0.05 | 0.05 | 0.17 |
| cg23684502 | *ST13* | -0.05 | 0.05 | 0.17 |
| cg23077453 | *SLC39A13* | -0.05 | 0.05 | 0.17 |
| cg26709300 | *NDUFS2* | 0.05 | 0.05 | 0.17 |
| cg05735639 | *SPAG9* | -0.05 | 0.05 | 0.17 |
| cg21554034 | *BIN1* | 0.05 | 0.05 | 0.17 |
| cg06494872 | *DGKQ* | 0.05 | 0.05 | 0.17 |
| cg04970434 | *GP1BA* | -0.05 | 0.05 | 0.17 |
| cg06581718 | *PEX11G* | 0.05 | 0.05 | 0.17 |
| cg09274826 | *HDGF* | 0.05 | 0.05 | 0.17 |
| cg21824343 | *WDR81* | -0.05 | 0.05 | 0.18 |
| cg04401876 | *CLU* | 0.05 | 0.05 | 0.18 |
| cg00346446 | *MRPL24* | 0.05 | 0.05 | 0.18 |
| cg19107120 | *GRN* | 0.05 | 0.05 | 0.18 |
| cg27052073 | *NKPD1* | -0.05 | 0.05 | 0.18 |
| cg00797651 | *YWHAQ* | 0.05 | 0.05 | 0.18 |
| cg25275011 | *ATP8B3* | 0.05 | 0.06 | 0.18 |
| cg05157791 | *RASAL1* | 0.05 | 0.06 | 0.18 |
| cg14097619 | *ABCF3* | -0.05 | 0.06 | 0.19 |
| cg05298252 | *CCDC62* | 0.05 | 0.06 | 0.19 |
| cg20908785 | *CDKN3* | -0.05 | 0.06 | 0.19 |
| cg03936955 | *CNTN2* | 0.05 | 0.06 | 0.19 |
| cg04590610 | *LYPD5* | -0.05 | 0.06 | 0.19 |
| cg03766620 | *BIN1* | 0.05 | 0.06 | 0.19 |
| cg03397724 | *AXIN1* | -0.05 | 0.06 | 0.19 |
| cg22801913 | *C11orf49* | 0.05 | 0.06 | 0.19 |
| cg08769634 | *MARVELD3* | -0.05 | 0.06 | 0.19 |
| cg18883472 | *CNIH4* | 0.05 | 0.06 | 0.19 |
| cg10038009 | *PYDC1* | -0.05 | 0.06 | 0.19 |
| cg10152097 | *FHL2* | -0.05 | 0.06 | 0.19 |
| cg24450653 | *RBBP5* | -0.05 | 0.06 | 0.19 |
| cg07528237 | *USP6* | 0.05 | 0.06 | 0.19 |
| cg06885782 | *KCNQ4* | -0.05 | 0.06 | 0.19 |
| cg10750934 | *NKPD1* | 0.05 | 0.06 | 0.19 |
| cg05372495 | *ABCA7* | 0.05 | 0.06 | 0.19 |
| cg23290456 | *KLF16* | 0.05 | 0.06 | 0.19 |
| cg08287334 | *KLF16* | -0.05 | 0.06 | 0.20 |
| cg16757332 | *SLC24A4* | -0.05 | 0.06 | 0.20 |
| cg11659317 | *PLEKHH2* | -0.05 | 0.06 | 0.20 |
| cg09319822 | *MRPL43* | 0.05 | 0.06 | 0.20 |
| cg26102728 | *JAZF1* | -0.05 | 0.06 | 0.20 |
| cg05298628 | *VPS37B* | -0.05 | 0.07 | 0.20 |
| cg01067137 | *ZNF646* | -0.05 | 0.07 | 0.20 |
| cg24741873 | *TSPAN14* | -0.05 | 0.07 | 0.20 |
| cg26115667 | *TRAF3* | 0.05 | 0.07 | 0.20 |
| cg08081390 | *FUS* | -0.05 | 0.07 | 0.20 |
| cg22718636 | *DGKQ* | 0.05 | 0.07 | 0.20 |
| cg02197387 | *TCEA2* | -0.05 | 0.07 | 0.20 |
| cg06521025 | *RASAL1* | -0.05 | 0.07 | 0.20 |
| cg24949191 | *HEXB* | -0.05 | 0.07 | 0.20 |
| cg09662430 | *AHRR* | -0.06 | 0.07 | 0.20 |
| cg23914149 | *ANK3* | 0.05 | 0.07 | 0.20 |
| cg02190038 | *SCAMP4* | -0.05 | 0.07 | 0.21 |
| cg08354187 | *CD6* | 0.05 | 0.07 | 0.21 |
| cg07713929 | *ZNF232* | -0.05 | 0.07 | 0.21 |
| cg06147733 | *LLGL1* | 0.05 | 0.07 | 0.21 |
| cg18512352 | *MTCH2* | 0.05 | 0.07 | 0.21 |
| cg22904711 | *KCNN4* | -0.05 | 0.07 | 0.21 |
| cg01904978 | *PICALM* | -0.05 | 0.07 | 0.21 |
| cg03144232 | *ATP8B3* | 0.05 | 0.07 | 0.21 |
| cg08525314 | *KLF16* | -0.05 | 0.07 | 0.21 |
| cg04778012 | *STAG3* | -0.05 | 0.07 | 0.21 |
| cg11376215 | *CKM* | -0.05 | 0.07 | 0.21 |
| cg26471390 | *PLEKHM1* | 0.05 | 0.08 | 0.22 |
| cg03493774 | *SLC24A4* | 0.05 | 0.08 | 0.22 |
| cg27268574 | *FCHO1* | -0.05 | 0.08 | 0.22 |
| cg05772917 | *MAPT* | 0.05 | 0.08 | 0.23 |
| cg16260349 | *TSPAN14* | -0.05 | 0.08 | 0.23 |
| cg23418219 | *CREB5* | -0.05 | 0.08 | 0.23 |
| cg18696900 | *FERMT2* | -0.05 | 0.08 | 0.23 |
| cg09495303 | *PICALM* | 0.05 | 0.08 | 0.23 |
| cg01283141 | *DOC2A* | -0.05 | 0.08 | 0.23 |
| cg13351161 | *SPI1* | -0.05 | 0.08 | 0.23 |
| cg27551605 | *CRHR1* | -0.05 | 0.09 | 0.24 |
| cg08539965 | *EIF4G3* | 0.05 | 0.09 | 0.24 |
| cg04780984 | *SPI1* | -0.05 | 0.09 | 0.24 |
| cg02711929 | *HTRA1* | -0.05 | 0.09 | 0.24 |
| cg11548732 | *BAZ2B* | -0.05 | 0.09 | 0.24 |
| cg15718572 | *CR1L* | -0.05 | 0.09 | 0.24 |
| cg09626299 | *TSPAN14* | 0.05 | 0.09 | 0.24 |
| cg26427908 | *SLC6A19* | -0.05 | 0.09 | 0.25 |
| cg01724917 | *XYLT1* | -0.05 | 0.09 | 0.25 |
| cg04703951 | *PLEKHM1* | 0.05 | 0.09 | 0.25 |
| cg11092048 | *MEIS2* | 0.05 | 0.09 | 0.25 |
| cg13880303 | *APOC1* | -0.05 | 0.09 | 0.25 |
| cg02128278 | *HYDIN* | -0.05 | 0.10 | 0.26 |
| cg26218411 | *SLTM* | -0.04 | 0.10 | 0.26 |
| cg08331138 | *ZFP3* | -0.04 | 0.10 | 0.26 |
| cg01958934 | *APOC4* | 0.04 | 0.10 | 0.26 |
| cg18200075 | *DUSP12* | -0.04 | 0.10 | 0.26 |
| cg09921810 | *TRIM72* | -0.04 | 0.10 | 0.28 |
| cg19811076 | *DPYSL3* | 0.04 | 0.10 | 0.28 |
| cg09921810 | *RING1* | -0.04 | 0.10 | 0.28 |
| cg09010156 | *MVP* | 0.04 | 0.11 | 0.28 |
| cg15773893 | *DOCK10* | 0.04 | 0.11 | 0.28 |
| cg19276736 | *ITSN2* | 0.04 | 0.11 | 0.28 |
| cg20428596 | *DGKD* | 0.04 | 0.11 | 0.29 |
| cg17728125 | *SBNO2* | -0.04 | 0.11 | 0.29 |
| cg21820656 | *SLTM* | 0.04 | 0.11 | 0.29 |
| cg00876272 | *TRAF3* | -0.04 | 0.11 | 0.30 |
| cg12176783 | *TCEA2* | -0.04 | 0.11 | 0.30 |
| cg09925874 | *ZNF646* | -0.04 | 0.11 | 0.30 |
| cg15742858 | *GPR68* | -0.04 | 0.12 | 0.30 |
| cg07068062 | *VARS* | 0.04 | 0.12 | 0.30 |
| cg27436184 | *APOC4* | 0.04 | 0.12 | 0.30 |
| cg24794833 | *CLU* | -0.04 | 0.12 | 0.30 |
| cg05585544 | *C1QTNF4* | 0.04 | 0.12 | 0.31 |
| cg04298253 | *PRDM7* | 0.04 | 0.12 | 0.31 |
| cg23165431 | *ZNF232* | -0.04 | 0.12 | 0.31 |
| cg15590387 | *SPAG9* | -0.04 | 0.13 | 0.32 |
| cg22684041 | *ADAT3* | -0.04 | 0.13 | 0.32 |
| cg07015354 | *CA12* | 0.04 | 0.13 | 0.33 |
| cg07501827 | *SCNN1B* | 0.04 | 0.13 | 0.33 |
| cg23550016 | *TCAP* | -0.04 | 0.13 | 0.33 |
| cg16556397 | *GIGYF1* | -0.04 | 0.13 | 0.34 |
| cg16516316 | *C1QTNF4* | -0.04 | 0.13 | 0.34 |
| cg06800231 | *DHRS2* | 0.04 | 0.14 | 0.34 |
| cg18691434 | *STAG3* | -0.04 | 0.14 | 0.34 |
| cg18073514 | *ABCA7* | -0.04 | 0.14 | 0.34 |
| cg01958934 | *ZNF668* | -0.04 | 0.14 | 0.34 |
| cg04956806 | *TCEA2* | -0.04 | 0.14 | 0.34 |
| cg09905416 | *MS4A2* | -0.04 | 0.14 | 0.34 |
| cg17501982 | *NCK2* | -0.04 | 0.14 | 0.34 |
| cg12009516 | *EXOC3L2* | 0.04 | 0.14 | 0.34 |
| cg09770579 | *XYLT1* | -0.04 | 0.14 | 0.34 |
| cg10494860 | *TCEA2* | -0.04 | 0.14 | 0.35 |
| cg14313833 | *ABCA1* | 0.04 | 0.14 | 0.35 |
| cg23037777 | *GCNT7* | -0.04 | 0.14 | 0.35 |
| cg09307264 | *KIF1C* | -0.04 | 0.14 | 0.35 |
| cg25792473 | *RNF212* | 0.04 | 0.14 | 0.35 |
| cg03075966 | *GRK5* | 0.04 | 0.15 | 0.35 |
| cg04958703 | *RNF167* | -0.04 | 0.15 | 0.36 |
| cg22375610 | *NCK2* | -0.04 | 0.15 | 0.36 |
| cg06153467 | *RRAD* | -0.04 | 0.15 | 0.36 |
| cg21888989 | *APH1B* | -0.04 | 0.15 | 0.36 |
| cg05668372 | *NPBWR2* | -0.04 | 0.15 | 0.37 |
| cg06685340 | *TSPAN14* | 0.04 | 0.15 | 0.37 |
| cg07997737 | *NRTN* | 0.04 | 0.15 | 0.37 |
| cg05918002 | *ZNF232* | -0.04 | 0.16 | 0.37 |
| cg04122657 | *ADCY9* | 0.04 | 0.16 | 0.37 |
| cg05019311 | *CHRNE* | -0.04 | 0.16 | 0.37 |
| cg07974891 | *CPSF3* | -0.04 | 0.16 | 0.37 |
| cg19591950 | *ZNF221* | -0.04 | 0.16 | 0.37 |
| cg03984055 | *VKORC1* | -0.04 | 0.16 | 0.37 |
| cg18788725 | *RELB* | -0.04 | 0.16 | 0.37 |
| cg24035928 | *NRTN* | 0.04 | 0.16 | 0.38 |
| cg12095353 | *UBC* | -0.04 | 0.16 | 0.38 |
| cg07366082 | *PCDH1* | -0.04 | 0.16 | 0.38 |
| cg07150862 | *STX1B* | -0.04 | 0.17 | 0.38 |
| cg02128278 | *SPATA2L* | -0.04 | 0.17 | 0.38 |
| cg04666465 | *GGA2* | 0.04 | 0.17 | 0.38 |
| cg00514353 | *PCDH1* | -0.04 | 0.17 | 0.38 |
| cg09324608 | *MYO1D* | 0.04 | 0.17 | 0.38 |
| cg18493147 | *ODF3L2* | -0.04 | 0.17 | 0.38 |
| cg04149015 | *PRSS36* | -0.04 | 0.17 | 0.38 |
| cg04723449 | *COG7* | -0.04 | 0.17 | 0.38 |
| cg08113562 | *ARHGAP27* | 0.04 | 0.17 | 0.39 |
| cg08464513 | *CORO1A* | -0.04 | 0.17 | 0.39 |
| cg23397216 | *GABBR1* | 0.04 | 0.17 | 0.39 |
| cg07661564 | *WNT3* | 0.04 | 0.17 | 0.39 |
| cg13792823 | *LCORL* | 0.04 | 0.18 | 0.40 |
| cg23871276 | *NRTN* | -0.04 | 0.18 | 0.40 |
| cg01727686 | *NUDT22* | 0.04 | 0.18 | 0.40 |
| cg01565314 | *EXOC3L2* | 0.04 | 0.18 | 0.40 |
| cg09433588 | *LYPD5* | 0.04 | 0.18 | 0.40 |
| cg03924776 | *REXO1* | 0.04 | 0.18 | 0.40 |
| cg04024572 | *CCDC85B* | 0.04 | 0.18 | 0.40 |
| cg22346765 | *UNC5CL* | 0.04 | 0.18 | 0.40 |
| cg05418781 | *GGA2* | 0.04 | 0.18 | 0.40 |
| cg02190038 | *ADAT3* | -0.04 | 0.18 | 0.40 |
| cg09157813 | *CASP12* | -0.04 | 0.18 | 0.40 |
| cg14301190 | *PRSS36* | 0.04 | 0.18 | 0.41 |
| cg02110529 | *RAD51C* | -0.04 | 0.19 | 0.41 |
| cg24657473 | *PSMF1* | -0.04 | 0.19 | 0.41 |
| cg01643690 | *TMEM106B* | -0.04 | 0.19 | 0.41 |
| cg05711037 | *DGKD* | -0.04 | 0.19 | 0.41 |
| cg24900654 | *KCNN4* | -0.04 | 0.19 | 0.41 |
| cg23478124 | *PICALM* | 0.04 | 0.19 | 0.42 |
| cg07868561 | *SH3TC2* | -0.04 | 0.19 | 0.42 |
| cg20363891 | *SNED1* | -0.04 | 0.19 | 0.42 |
| cg08989478 | *MAT1A* | -0.04 | 0.19 | 0.42 |
| cg00249205 | *STX1B* | -0.04 | 0.19 | 0.42 |
| cg04227961 | *RASAL1* | 0.04 | 0.19 | 0.42 |
| cg00553149 | *STAG3* | -0.03 | 0.20 | 0.42 |
| cg10582946 | *LCORL* | 0.03 | 0.20 | 0.42 |
| cg19532212 | *TEX14* | -0.04 | 0.20 | 0.43 |
| cg19914904 | *LZTS2* | 0.03 | 0.20 | 0.43 |
| cg02870744 | *AXIN1* | 0.03 | 0.20 | 0.43 |
| cg10045909 | *ZNF668* | -0.03 | 0.20 | 0.43 |
| cg00694258 | *ARHGAP12* | 0.03 | 0.20 | 0.43 |
| cg17614165 | *RASAL1* | 0.03 | 0.21 | 0.43 |
| cg11906021 | *PHB* | 0.03 | 0.21 | 0.44 |
| cg01835620 | *GP1BA* | 0.03 | 0.21 | 0.45 |
| cg00346446 | *HDGF* | 0.03 | 0.21 | 0.45 |
| cg16529268 | *CBLC* | -0.03 | 0.21 | 0.45 |
| cg10380221 | *ZNF668* | 0.03 | 0.22 | 0.45 |
| cg09509673 | *CCR10* | 0.03 | 0.22 | 0.45 |
| cg25533154 | *PPM1E* | -0.03 | 0.22 | 0.45 |
| cg24158141 | *CD55* | 0.03 | 0.22 | 0.46 |
| cg13198297 | *MYO15A* | 0.03 | 0.22 | 0.46 |
| cg01831454 | *SH3TC2* | -0.03 | 0.22 | 0.46 |
| cg20301962 | *FZD2* | 0.03 | 0.22 | 0.46 |
| cg24360197 | *MAP2K1* | 0.03 | 0.22 | 0.46 |
| cg14123992 | *TOMM40* | -0.03 | 0.23 | 0.46 |
| cg20800039 | *ADAM10* | -0.03 | 0.23 | 0.47 |
| cg02488299 | *ASPHD1* | 0.03 | 0.23 | 0.47 |
| cg03054050 | *ZDHHC14* | 0.03 | 0.23 | 0.47 |
| cg10619398 | *KCNB1* | -0.03 | 0.23 | 0.48 |
| cg18471635 | *CASP12* | -0.03 | 0.24 | 0.48 |
| cg07928695 | *TRAPPC6A* | -0.03 | 0.24 | 0.48 |
| cg17155859 | *PLCB3* | -0.03 | 0.24 | 0.48 |
| cg02833725 | *ISG20L2* | 0.03 | 0.24 | 0.48 |
| cg07736657 | *IQCK* | -0.03 | 0.24 | 0.49 |
| cg13299728 | *VPS37B* | -0.03 | 0.24 | 0.49 |
| cg09106231 | *C4BPA* | -0.03 | 0.24 | 0.49 |
| cg09584855 | *SEZ6L2* | 0.03 | 0.24 | 0.49 |
| cg11321190 | *LRRFIP2* | 0.03 | 0.24 | 0.49 |
| cg24348495 | *TCEA2* | -0.03 | 0.24 | 0.49 |
| cg07249224 | *TBX6* | 0.03 | 0.25 | 0.49 |
| cg15770106 | *PLXNA2* | -0.03 | 0.25 | 0.49 |
| cg10633906 | *TMEM184A* | 0.03 | 0.25 | 0.49 |
| cg04406254 | *TOMM40* | -0.03 | 0.25 | 0.49 |
| cg20135002 | *SORL1* | -0.03 | 0.25 | 0.49 |
| cg03226208 | *ZFP3* | 0.03 | 0.25 | 0.49 |
| cg04305808 | *ZKSCAN1* | -0.03 | 0.25 | 0.49 |
| cg21208029 | *STYX* | -0.03 | 0.25 | 0.49 |
| cg03458229 | *MYO1C* | 0.03 | 0.25 | 0.49 |
| cg00192773 | *MAPK3* | 0.03 | 0.25 | 0.49 |
| cg05249026 | *DEXI* | -0.03 | 0.25 | 0.49 |
| cg22500738 | *CD46* | -0.03 | 0.25 | 0.49 |
| cg01934064 | *MAPT* | -0.03 | 0.25 | 0.49 |
| cg26615017 | *ERBB2* | -0.03 | 0.25 | 0.49 |
| cg07198170 | *MAFK* | -0.03 | 0.25 | 0.50 |
| cg11641595 | *SERPINE2* | -0.03 | 0.26 | 0.50 |
| cg13496662 | *APOC1* | -0.03 | 0.26 | 0.50 |
| cg07071809 | *TMEM184A* | -0.03 | 0.26 | 0.50 |
| cg04895225 | *EED* | -0.03 | 0.26 | 0.50 |
| cg15390894 | *GPR68* | 0.03 | 0.26 | 0.50 |
| cg20401945 | *ASPHD1* | 0.03 | 0.26 | 0.50 |
| cg02333467 | *GIGYF1* | 0.03 | 0.26 | 0.51 |
| cg04780984 | *DUS3L* | -0.03 | 0.26 | 0.51 |
| cg03267442 | *TSPAN14* | 0.03 | 0.26 | 0.51 |
| cg05809481 | *ISG20L2* | -0.03 | 0.26 | 0.51 |
| cg24360197 | *TIPIN* | 0.03 | 0.27 | 0.51 |
| cg03457195 | *TBR1* | 0.03 | 0.27 | 0.51 |
| cg15120925 | *RASAL1* | 0.03 | 0.27 | 0.51 |
| cg17852791 | *SPAG9* | -0.03 | 0.27 | 0.51 |
| cg13095627 | *IGFL4* | -0.03 | 0.27 | 0.51 |
| cg13572146 | *FUT6* | -0.03 | 0.27 | 0.51 |
| cg27582585 | *KLHDC9* | -0.03 | 0.27 | 0.51 |
| cg05731156 | *ZNF771* | -0.03 | 0.27 | 0.51 |
| cg03584351 | *ZFAND2A* | -0.03 | 0.27 | 0.51 |
| cg16429505 | *ACHE* | -0.03 | 0.27 | 0.52 |
| cg07698783 | *SPI1* | 0.03 | 0.28 | 0.52 |
| cg08893833 | *STX4* | 0.03 | 0.28 | 0.53 |
| cg09326345 | *INSRR* | 0.03 | 0.28 | 0.53 |
| cg10370574 | *REXO1* | -0.03 | 0.29 | 0.53 |
| cg25941154 | *PSORS1C1* | 0.03 | 0.29 | 0.53 |
| cg08515989 | *ASPHD1* | 0.03 | 0.29 | 0.53 |
| cg24750513 | *BIN1* | 0.03 | 0.29 | 0.53 |
| cg07079724 | *CPLX1* | 0.03 | 0.29 | 0.53 |
| cg11930955 | *TPCN1* | -0.03 | 0.29 | 0.53 |
| cg24037389 | *SCARA5* | 0.03 | 0.29 | 0.53 |
| cg09245872 | *DOC2A* | -0.03 | 0.29 | 0.53 |
| cg01089331 | *LCORL* | 0.03 | 0.29 | 0.53 |
| cg10426084 | *WDR81* | -0.03 | 0.29 | 0.53 |
| cg26848071 | *KIF1C* | 0.03 | 0.29 | 0.53 |
| cg06641342 | *CYB561* | -0.03 | 0.29 | 0.53 |
| cg09162137 | *USP8* | -0.03 | 0.29 | 0.54 |
| cg11376215 | *KLC3* | -0.03 | 0.30 | 0.54 |
| cg21657705 | *ACE* | -0.03 | 0.30 | 0.55 |
| cg09172973 | *ZNF646* | -0.03 | 0.30 | 0.55 |
| cg01814191 | *LRRFIP2* | -0.03 | 0.30 | 0.55 |
| cg07661564 | *WNT9B* | 0.03 | 0.30 | 0.55 |
| cg23300486 | *TRAF3* | 0.03 | 0.30 | 0.55 |
| cg02747254 | *KCTD13* | 0.03 | 0.31 | 0.55 |
| cg21368161 | *TMEM184A* | 0.03 | 0.31 | 0.55 |
| cg22291189 | *MAPT* | 0.03 | 0.31 | 0.55 |
| cg13049862 | *ITGAM* | -0.03 | 0.31 | 0.55 |
| cg25222089 | *BASP1* | -0.03 | 0.31 | 0.55 |
| cg03181966 | *PRSS36* | 0.03 | 0.31 | 0.55 |
| cg07934434 | *GRID1* | 0.03 | 0.31 | 0.55 |
| cg10001987 | *ZBTB4* | -0.03 | 0.31 | 0.56 |
| cg24925163 | *WDR33* | -0.03 | 0.31 | 0.56 |
| cg25510838 | *INTS1* | -0.03 | 0.31 | 0.56 |
| cg22062555 | *NKPD1* | -0.03 | 0.32 | 0.56 |
| cg03117793 | *ERCC2* | -0.03 | 0.32 | 0.56 |
| cg12095353 | *SCARB1* | -0.03 | 0.32 | 0.57 |
| cg10749413 | *ABCA7* | 0.03 | 0.33 | 0.58 |
| cg03946671 | *TCEA2* | 0.33 | 0.33 | 0.58 |
| cg20456258 | *DGKQ* | 0.03 | 0.33 | 0.58 |
| cg10387761 | *ADAM11* | -0.03 | 0.33 | 0.58 |
| cg20373012 | *UBASH3B* | 0.03 | 0.33 | 0.58 |
| cg21742836 | *ALDOA* | -0.03 | 0.33 | 0.58 |
| cg04113154 | *GAK* | -0.03 | 0.33 | 0.58 |
| cg24677220 | *MAPT* | 0.03 | 0.33 | 0.58 |
| cg01753241 | *TMEM184A* | 0.03 | 0.34 | 0.58 |
| cg02556718 | *SEZ6L2* | 0.03 | 0.34 | 0.59 |
| cg24060040 | *FLOT1* | 0.03 | 0.34 | 0.59 |
| cg04541293 | *PTPRT* | -0.03 | 0.34 | 0.59 |
| cg04436971 | *INO80E* | -0.03 | 0.34 | 0.59 |
| cg25246158 | *BLNK* | -0.03 | 0.34 | 0.59 |
| cg04839683 | *SOX6* | -0.03 | 0.34 | 0.59 |
| cg00259097 | *VAC14* | 0.03 | 0.34 | 0.59 |
| cg22593342 | *TSPAN14* | 0.03 | 0.34 | 0.59 |
| cg00958217 | *PHF13* | -0.03 | 0.34 | 0.59 |
| cg21995147 | *ABCA7* | 0.03 | 0.34 | 0.59 |
| cg13548946 | *VPS37B* | 0.03 | 0.35 | 0.59 |
| cg13210467 | *STAG3* | -0.03 | 0.35 | 0.60 |
| cg24029517 | *HS3ST1* | -0.03 | 0.35 | 0.60 |
| cg18696900 | *DDHD1* | 0.03 | 0.35 | 0.60 |
| cg14731657 | *DUS3L* | -0.03 | 0.35 | 0.60 |
| cg08374890 | *VKORC1* | -0.03 | 0.35 | 0.60 |
| cg00449767 | *EXOC3L2* | 0.03 | 0.35 | 0.60 |
| cg09990600 | *TMEM184A* | 0.03 | 0.35 | 0.60 |
| cg05585544 | *MTCH2* | 0.02 | 0.36 | 0.60 |
| cg09326345 | *NTRK1* | 0.02 | 0.36 | 0.60 |
| cg05920870 | *PCOLCE* | -0.02 | 0.36 | 0.60 |
| cg07969646 | *IER5* | 0.02 | 0.36 | 0.60 |
| cg06420512 | *FGF17* | -0.02 | 0.36 | 0.60 |
| cg07778819 | *CRHR1* | -0.02 | 0.36 | 0.60 |
| cg21701774 | *LPCAT3* | 0.02 | 0.36 | 0.60 |
| cg00123512 | *TBX6* | -0.02 | 0.36 | 0.60 |
| cg11579407 | *REXO1* | 0.02 | 0.36 | 0.60 |
| cg26576206 | *ABCA7* | 0.02 | 0.36 | 0.60 |
| cg26470501 | *BCL3* | -0.02 | 0.36 | 0.60 |
| cg13536080 | *CCDC83* | -0.02 | 0.36 | 0.60 |
| cg06925179 | *PLEKHM1* | 0.02 | 0.37 | 0.60 |
| cg17634650 | *CLNK* | -0.02 | 0.37 | 0.60 |
| cg19687457 | *GPC2* | -0.02 | 0.37 | 0.60 |
| cg13382072 | *FGF17* | -0.02 | 0.37 | 0.61 |
| cg14599956 | *LMAN2* | 0.02 | 0.38 | 0.62 |
| cg01777341 | *GALNT6* | -0.02 | 0.38 | 0.62 |
| cg08474334 | *MARVELD3* | 0.02 | 0.38 | 0.62 |
| cg02991238 | *NPBWR2* | -0.02 | 0.38 | 0.62 |
| cg17487705 | *CCDC81* | 0.02 | 0.38 | 0.62 |
| cg12453228 | *NCR2* | 0.02 | 0.38 | 0.62 |
| cg22711218 | *DSTYK* | -0.02 | 0.38 | 0.62 |
| cg08889009 | *SLTM* | 0.02 | 0.38 | 0.62 |
| cg25809561 | *MYO1D* | -0.02 | 0.38 | 0.62 |
| cg13604020 | *DDX17* | 0.02 | 0.38 | 0.62 |
| cg04326499 | *CLIC5* | 0.02 | 0.39 | 0.62 |
| cg01670771 | *STX1B* | -0.02 | 0.39 | 0.62 |
| cg00515669 | *LYPD5* | 0.02 | 0.39 | 0.62 |
| cg04477789 | *FBXL19* | -0.02 | 0.39 | 0.62 |
| cg08300860 | *LDB3* | 0.02 | 0.39 | 0.62 |
| cg25644380 | *BCL3* | -0.02 | 0.39 | 0.62 |
| cg25033993 | *STX1B* | 0.02 | 0.39 | 0.62 |
| cg21064333 | *CA12* | -0.02 | 0.39 | 0.62 |
| cg03890691 | *DOC2A* | -0.02 | 0.39 | 0.62 |
| cg16673712 | *UFC1* | 0.02 | 0.39 | 0.62 |
| cg24580001 | *CCDC88B* | -0.02 | 0.39 | 0.62 |
| cg22608507 | *VPS37B* | -0.02 | 0.39 | 0.62 |
| cg01133262 | *KDELR3* | -0.02 | 0.39 | 0.62 |
| cg13485809 | *TPCN1* | -0.02 | 0.39 | 0.62 |
| cg17548735 | *APOBEC2* | -0.02 | 0.40 | 0.63 |
| cg08524344 | *DUS3L* | -0.02 | 0.40 | 0.63 |
| cg22163406 | *MANBAL* | -0.02 | 0.40 | 0.63 |
| cg16233074 | *FAM171A2* | 0.02 | 0.40 | 0.63 |
| cg00524708 | *GPX6* | -0.02 | 0.40 | 0.63 |
| cg06387669 | *SERPINF2* | -0.02 | 0.40 | 0.63 |
| cg07150862 | *GGA2* | 0.02 | 0.40 | 0.63 |
| cg05881698 | *DUS3L* | 0.04 | 0.41 | 0.63 |
| cg13201808 | *FHL2* | -0.02 | 0.41 | 0.64 |
| cg03812107 | *APOBEC2* | -0.02 | 0.41 | 0.64 |
| cg23871276 | *FUT6* | -0.02 | 0.41 | 0.64 |
| cg21959090 | *EXOC3L2* | 0.02 | 0.41 | 0.64 |
| cg23427912 | *TRIML1* | 0.02 | 0.41 | 0.64 |
| cg08124317 | *GGA2* | 0.02 | 0.42 | 0.64 |
| cg16568360 | *STAG3* | 0.02 | 0.42 | 0.64 |
| cg26429636 | *AZGP1* | -0.02 | 0.42 | 0.65 |
| cg04662983 | *PPM1E* | 0.02 | 0.42 | 0.65 |
| cg08319238 | *BCAM* | -0.02 | 0.42 | 0.65 |
| cg04650094 | *UNC5CL* | -0.02 | 0.42 | 0.65 |
| cg06189038 | *GPC2* | 0.02 | 0.42 | 0.65 |
| cg08898775 | *ADAM10* | 0.02 | 0.42 | 0.65 |
| cg00296018 | *PCDH1* | -0.02 | 0.42 | 0.65 |
| cg09645925 | *ZNF764* | 0.02 | 0.43 | 0.66 |
| cg21810604 | *AP4M1* | -0.02 | 0.43 | 0.66 |
| cg24158141 | *C4BPA* | -0.02 | 0.43 | 0.66 |
| cg18011803 | *FBXL3* | 0.02 | 0.43 | 0.66 |
| cg11935896 | *SH3TC2* | 0.02 | 0.43 | 0.66 |
| cg13259925 | *LRAT* | 0.02 | 0.43 | 0.66 |
| cg24035928 | *DUS3L* | 0.02 | 0.43 | 0.66 |
| cg25219643 | *CCDC81* | -0.02 | 0.44 | 0.66 |
| cg24554944 | *SCARA5* | 0.02 | 0.44 | 0.66 |
| cg08281777 | *IL34* | -0.02 | 0.44 | 0.66 |
| cg14795672 | *PPIL3* | 0.02 | 0.44 | 0.66 |
| cg04780984 | *NRTN* | 0.02 | 0.44 | 0.66 |
| cg07928695 | *BLOC1S3* | 0.02 | 0.44 | 0.66 |
| cg25917893 | *CLPTM1* | -0.02 | 0.44 | 0.67 |
| cg01229998 | *MS4A4A* | -0.02 | 0.44 | 0.67 |
| cg01090611 | *HNRNPD* | -0.02 | 0.45 | 0.67 |
| cg12234009 | *TMEM184A* | -0.02 | 0.45 | 0.67 |
| cg18283321 | *DDC* | 0.02 | 0.45 | 0.67 |
| cg01120173 | *CRHR1* | -0.02 | 0.45 | 0.67 |
| cg06323624 | *ZNF689* | -0.02 | 0.45 | 0.67 |
| cg14047091 | *PCOLCE* | 0.02 | 0.45 | 0.67 |
| cg09662430 | *PDCD6* | 0.03 | 0.45 | 0.67 |
| cg01550271 | *DRG1* | 0.02 | 0.45 | 0.67 |
| cg19276736 | *NCOA1* | -0.02 | 0.45 | 0.67 |
| cg25792473 | *FGFRL1* | 0.02 | 0.45 | 0.67 |
| cg16556397 | *POP7* | -0.02 | 0.45 | 0.67 |
| cg19622675 | *PFDN1* | -0.02 | 0.45 | 0.67 |
| cg03226208 | *ZNF232* | -0.02 | 0.46 | 0.68 |
| cg16348385 | *YPEL3* | 0.02 | 0.46 | 0.68 |
| cg04192393 | *CA12* | -0.02 | 0.47 | 0.68 |
| cg09435890 | *PRDM7* | -0.02 | 0.47 | 0.68 |
| cg09758420 | *NCR2* | 0.02 | 0.47 | 0.68 |
| cg06242000 | *ALG3* | -0.02 | 0.47 | 0.68 |
| cg21946667 | *CNTNAP1* | -0.02 | 0.47 | 0.68 |
| cg09934309 | *COG7* | -0.02 | 0.47 | 0.68 |
| cg00553149 | *MDC1* | 0.02 | 0.47 | 0.68 |
| cg05806717 | *TBX6* | -0.02 | 0.47 | 0.68 |
| cg13732302 | *LRRFIP2* | -0.02 | 0.47 | 0.69 |
| cg17365250 | *ACHE* | 0.02 | 0.47 | 0.69 |
| cg19077231 | *GABBR1* | 0.02 | 0.48 | 0.69 |
| cg08441314 | *MTCH2* | 0.02 | 0.48 | 0.70 |
| cg20891655 | *CCDC83* | 0.02 | 0.48 | 0.70 |
| cg08312330 | *DEFA6* | -0.02 | 0.48 | 0.70 |
| cg12857074 | *KIF21B* | -0.02 | 0.48 | 0.70 |
| cg01027010 | *SCARA5* | 0.02 | 0.48 | 0.70 |
| cg09576143 | *HTRA1* | -0.02 | 0.49 | 0.70 |
| cg24848787 | *LIPC* | -0.02 | 0.49 | 0.70 |
| cg17049621 | *FZD2* | 0.02 | 0.49 | 0.70 |
| cg03491459 | *CA12* | 0.02 | 0.49 | 0.70 |
| cg26338757 | *LDB3* | -0.02 | 0.49 | 0.70 |
| cg03642555 | *RAI14* | 0.02 | 0.49 | 0.70 |
| cg26458287 | *PRDM7* | 0.02 | 0.50 | 0.70 |
| cg02991238 | *OPRL1* | -0.02 | 0.50 | 0.70 |
| cg18861713 | *CD55* | -0.02 | 0.50 | 0.71 |
| cg01396723 | *PVR* | 0.02 | 0.51 | 0.72 |
| cg04344923 | *INTS1* | 0.02 | 0.51 | 0.72 |
| cg05418781 | *STX1B* | 0.02 | 0.51 | 0.72 |
| cg02616906 | *HYDIN* | 0.02 | 0.51 | 0.72 |
| cg13884879 | *ASAP2* | -0.02 | 0.51 | 0.72 |
| cg05646515 | *LMF1* | -0.02 | 0.51 | 0.72 |
| cg13234569 | *NRTN* | -0.02 | 0.51 | 0.72 |
| cg00120948 | *RERE* | 0.02 | 0.51 | 0.72 |
| cg02766895 | *TPCN1* | -0.02 | 0.51 | 0.72 |
| cg07997737 | *DUS3L* | 0.02 | 0.52 | 0.72 |
| cg16049864 | *EXOC3L2* | -0.02 | 0.52 | 0.72 |
| cg00387658 | *CASS4* | 0.02 | 0.52 | 0.72 |
| cg05299077 | *INPP5D* | -0.02 | 0.52 | 0.72 |
| cg11157611 | *EIF4G3* | 0.02 | 0.52 | 0.72 |
| cg26161329 | *PPM1E* | -0.02 | 0.52 | 0.72 |
| cg18910534 | *CLK1* | -0.02 | 0.52 | 0.72 |
| cg01756902 | *ZGPAT* | 0.02 | 0.52 | 0.72 |
| cg14586180 | *KLC3* | -0.02 | 0.52 | 0.72 |
| cg03144232 | *STX1B* | -0.02 | 0.52 | 0.72 |
| cg13668450 | *KLC3* | 0.02 | 0.52 | 0.72 |
| cg04903600 | *GPX4* | 0.02 | 0.53 | 0.73 |
| cg19935065 | *BLNK* | -0.02 | 0.53 | 0.73 |
| cg17152989 | *GJC3* | 0.02 | 0.53 | 0.73 |
| cg23635599 | *CBLC* | -0.02 | 0.53 | 0.73 |
| cg23754934 | *MS4A4A* | 0.02 | 0.53 | 0.73 |
| cg05644480 | *APOC1* | -0.02 | 0.53 | 0.73 |
| cg18383668 | *GEMIN7* | -0.02 | 0.53 | 0.73 |
| cg10624665 | *FZD2* | -0.02 | 0.53 | 0.73 |
| cg04254681 | *ADAM17* | -0.02 | 0.53 | 0.73 |
| cg04005334 | *PHB* | 0.02 | 0.54 | 0.73 |
| cg11824827 | *DYDC2* | -0.02 | 0.54 | 0.73 |
| cg20336341 | *TAP2* | 0.02 | 0.54 | 0.73 |
| cg00008446 | *INSRR* | 0.02 | 0.54 | 0.73 |
| cg03134962 | *ZNF668* | -0.02 | 0.54 | 0.74 |
| cg18200075 | *FCRLB* | 0.02 | 0.54 | 0.74 |
| cg20442379 | *IPMK* | -0.02 | 0.55 | 0.74 |
| cg12914100 | *TPCN1* | 0.02 | 0.55 | 0.74 |
| cg13074682 | *GPR176* | -0.02 | 0.55 | 0.74 |
| cg25535587 | *DYDC1* | 0.02 | 0.55 | 0.75 |
| cg06295687 | *HIRIP3* | -0.02 | 0.55 | 0.75 |
| cg11737831 | *ARID3A* | 0.02 | 0.56 | 0.75 |
| cg05550612 | *GLG1* | -0.02 | 0.56 | 0.75 |
| cg21959090 | *MARK4* | 0.02 | 0.56 | 0.75 |
| cg10850838 | *NODAL* | 0.02 | 0.56 | 0.75 |
| cg18682103 | *PYDC1* | -0.02 | 0.56 | 0.75 |
| cg03584351 | *C7orf50* | -0.02 | 0.56 | 0.75 |
| cg25556122 | *EXOC3L2* | -0.02 | 0.56 | 0.75 |
| cg13777937 | *FAM171A2* | 0.02 | 0.56 | 0.75 |
| cg00327577 | *PYCARD* | -0.02 | 0.57 | 0.75 |
| cg00788025 | *DGKQ* | -0.02 | 0.57 | 0.75 |
| cg08865522 | *RASAL1* | 0.02 | 0.57 | 0.75 |
| cg04697562 | *STAG3* | -0.02 | 0.57 | 0.75 |
| cg17445845 | *ATP8B4* | 0.02 | 0.57 | 0.75 |
| cg09352908 | *EPM2AIP1* | 0.02 | 0.57 | 0.76 |
| cg14207553 | *SH3TC2* | -0.02 | 0.57 | 0.76 |
| cg08436089 | *GEMIN7* | -0.02 | 0.58 | 0.76 |
| cg05006231 | *KLF16* | -0.02 | 0.58 | 0.76 |
| cg03635300 | *DUS3L* | -0.02 | 0.58 | 0.76 |
| cg16438688 | *INSRR* | 0.02 | 0.58 | 0.76 |
| cg21584422 | *RASAL1* | -0.01 | 0.58 | 0.76 |
| cg19668951 | *PCGF3* | -0.01 | 0.58 | 0.76 |
| cg08769634 | *TAT* | -0.01 | 0.58 | 0.76 |
| cg19998289 | *RNF34* | -0.01 | 0.58 | 0.76 |
| cg19098710 | *RASAL1* | 0.01 | 0.58 | 0.76 |
| cg24893073 | *DNAH2* | -0.01 | 0.58 | 0.76 |
| cg24474319 | *CRABP2* | 0.01 | 0.59 | 0.76 |
| cg07007312 | *DGKQ* | 0.01 | 0.59 | 0.76 |
| cg17024523 | *MLH1* | 0.01 | 0.59 | 0.76 |
| cg06129210 | *XYLT1* | -0.01 | 0.59 | 0.76 |
| cg08524344 | *NRTN* | -0.01 | 0.59 | 0.76 |
| cg12408990 | *RASGEF1C* | -0.01 | 0.59 | 0.76 |
| cg18705808 | *TMEM184A* | 0.01 | 0.59 | 0.76 |
| cg15770106 | *CD34* | 0.01 | 0.59 | 0.76 |
| cg01757806 | *MYH10* | -0.01 | 0.60 | 0.77 |
| cg04998634 | *KLF16* | -0.01 | 0.60 | 0.77 |
| cg09495282 | *TMEM184A* | -0.01 | 0.60 | 0.77 |
| cg02747254 | *TRIM40* | -0.01 | 0.60 | 0.77 |
| cg15755265 | *CA12* | 0.01 | 0.60 | 0.77 |
| cg02870744 | *MRPL28* | 0.01 | 0.60 | 0.77 |
| cg03629107 | *SNX31* | -0.01 | 0.60 | 0.77 |
| cg11304212 | *UHRF1BP1* | -0.01 | 0.61 | 0.77 |
| cg10238917 | *FBXL19* | -0.01 | 0.61 | 0.78 |
| cg11930955 | *ZNF668* | 0.01 | 0.61 | 0.78 |
| cg19465374 | *AZGP1* | -0.01 | 0.61 | 0.78 |
| cg19935065 | *DNTT* | -0.01 | 0.62 | 0.78 |
| cg19753476 | *CHRNE* | 0.01 | 0.62 | 0.79 |
| cg11998307 | *RILP* | -0.01 | 0.62 | 0.79 |
| cg23684502 | *SLC25A17* | 0.01 | 0.62 | 0.79 |
| cg19532212 | *RAD51C* | 0.01 | 0.63 | 0.79 |
| cg18185554 | *TRAF3* | 0.01 | 0.63 | 0.79 |
| cg07461273 | *MCM7* | -0.01 | 0.63 | 0.80 |
| cg22883290 | *BIN1* | 0.01 | 0.63 | 0.80 |
| cg27525032 | *TRAPPC6A* | -0.01 | 0.64 | 0.80 |
| cg24180227 | *ERCC2* | -0.01 | 0.64 | 0.80 |
| cg24580001 | *PRDX5* | -0.01 | 0.64 | 0.80 |
| cg16831889 | *HDC* | 0.01 | 0.64 | 0.80 |
| cg26744081 | *JAZF1* | 0.01 | 0.64 | 0.80 |
| cg08594681 | *CLU* | -0.01 | 0.64 | 0.80 |
| cg11738421 | *AP4M1* | -0.01 | 0.64 | 0.81 |
| cg16799087 | *PRDM7* | 0.01 | 0.65 | 0.81 |
| cg00409696 | *APOBEC2* | -0.01 | 0.65 | 0.81 |
| cg13294032 | *KIF1C* | -0.01 | 0.65 | 0.81 |
| cg03008525 | *MAFK* | -0.01 | 0.66 | 0.82 |
| cg07838048 | *VAC14* | 0.01 | 0.66 | 0.82 |
| cg17397159 | *BTNL2* | -0.01 | 0.66 | 0.82 |
| cg09794131 | *SPATA2L* | 0.01 | 0.66 | 0.82 |
| cg16233074 | *STX1B* | -0.01 | 0.67 | 0.83 |
| cg12019109 | *AZGP1* | -0.01 | 0.67 | 0.83 |
| cg02557922 | *TMEM18* | -0.01 | 0.67 | 0.83 |
| cg04156293 | *ACHE* | -0.01 | 0.67 | 0.83 |
| cg12763546 | *RASAL1* | -0.01 | 0.68 | 0.83 |
| cg20090143 | *APOC2* | 0.01 | 0.68 | 0.83 |
| cg08346922 | *PYDC1* | -0.01 | 0.68 | 0.84 |
| cg14183865 | *CPLX1* | 0.01 | 0.68 | 0.84 |
| cg09626299 | *PRDM7* | 0.01 | 0.68 | 0.84 |
| cg23595451 | *FGFRL1* | -0.01 | 0.68 | 0.84 |
| cg20291846 | *ADAM17* | 0.01 | 0.68 | 0.84 |
| cg09921810 | *PYDC1* | -0.01 | 0.69 | 0.84 |
| cg01699740 | *GRAMD1B* | 0.01 | 0.69 | 0.84 |
| cg23397216 | *PPP1R13L* | 0.01 | 0.69 | 0.84 |
| cg05989429 | *ABCA7* | 0.01 | 0.69 | 0.84 |
| cg11737831 | *WDR18* | 0.01 | 0.69 | 0.84 |
| cg03526776 | *TREML2* | 0.01 | 0.70 | 0.85 |
| cg00392257 | *ISG20L2* | 0.01 | 0.70 | 0.85 |
| cg01402814 | *ZNF771* | -0.01 | 0.70 | 0.85 |
| cg19427746 | *CAMTA2* | -0.01 | 0.70 | 0.85 |
| cg14002172 | *AP4M1* | 0.01 | 0.70 | 0.85 |
| cg11107149 | *DGKQ* | -0.01 | 0.70 | 0.85 |
| cg09645925 | *ZNF688* | -0.01 | 0.70 | 0.85 |
| cg16233074 | *ITGA2B* | -0.01 | 0.70 | 0.85 |
| cg01792666 | *SRCAP* | -0.01 | 0.70 | 0.85 |
| cg10093648 | *ASPHD1* | 0.01 | 0.71 | 0.85 |
| cg22360649 | *CCDC85B* | 0.01 | 0.71 | 0.85 |
| cg06866423 | *BACH2* | 0.01 | 0.71 | 0.85 |
| cg00974523 | *PRDM7* | -0.01 | 0.71 | 0.85 |
| cg23463186 | *TSPAN14* | -0.01 | 0.71 | 0.85 |
| cg01390564 | *PRSS36* | 0.01 | 0.71 | 0.85 |
| cg26949037 | *STX1B* | 0.01 | 0.72 | 0.85 |
| cg01231165 | *ADAM17* | -0.01 | 0.72 | 0.85 |
| cg24455236 | *PLEKHH3* | 0.01 | 0.72 | 0.85 |
| cg07064595 | *LACTB* | -0.01 | 0.72 | 0.85 |
| cg03057409 | *SEMA4G* | 0.01 | 0.72 | 0.85 |
| cg20157577 | *STRADA* | 0.01 | 0.72 | 0.86 |
| cg16292768 | *CLU* | -0.01 | 0.72 | 0.86 |
| cg21433558 | *CNTNAP1* | 0.01 | 0.73 | 0.86 |
| cg05149307 | *USP6* | -0.01 | 0.73 | 0.86 |
| cg13879655 | *CLU* | 0.01 | 0.73 | 0.86 |
| cg08331138 | *ZNF232* | 0.01 | 0.73 | 0.86 |
| cg12778183 | *TEX14* | -0.01 | 0.73 | 0.87 |
| cg05937818 | *HGC6.3* | 0.01 | 0.74 | 0.87 |
| cg06015834 | *DOC2A* | -0.01 | 0.74 | 0.87 |
| cg06411551 | *DYDC2* | -0.01 | 0.74 | 0.87 |
| cg00296018 | *ARAP3* | -0.01 | 0.74 | 0.87 |
| cg16398362 | *TEX14* | 0.01 | 0.74 | 0.87 |
| cg22580353 | *PVR* | -0.01 | 0.74 | 0.87 |
| cg14053764 | *FBXO34* | -0.01 | 0.74 | 0.87 |
| cg16611967 | *PRDM7* | -0.01 | 0.74 | 0.87 |
| cg06491244 | *WNT3* | -0.01 | 0.74 | 0.87 |
| cg09253125 | *IQCD* | 0.01 | 0.74 | 0.87 |
| cg01266287 | *SPN* | 0.01 | 0.75 | 0.87 |
| cg10792307 | *TREML1* | 0.01 | 0.75 | 0.87 |
| cg23427912 | *TRIML2* | -0.01 | 0.76 | 0.88 |
| cg17829936 | *TAAR5* | -0.01 | 0.76 | 0.88 |
| cg04049981 | *WFIKKN2* | 0.01 | 0.76 | 0.88 |
| cg07713929 | *ZFP3* | 0.01 | 0.76 | 0.88 |
| cg16557858 | *ERBB2* | -0.01 | 0.76 | 0.88 |
| cg06329491 | *SPAG9* | -0.01 | 0.76 | 0.88 |
| cg18506018 | *KCNN4* | -0.01 | 0.76 | 0.88 |
| cg14731657 | *NRTN* | -0.01 | 0.76 | 0.88 |
| cg18682103 | *TRIM72* | -0.01 | 0.76 | 0.88 |
| cg21075986 | *ADAM6* | 0.01 | 0.76 | 0.88 |
| cg08966293 | *MAPK3* | 0.01 | 0.77 | 0.88 |
| cg08203460 | *TAP2* | 0.01 | 0.77 | 0.88 |
| cg16934685 | *FAM131B* | -0.01 | 0.77 | 0.88 |
| cg04633141 | *TMEM184A* | -0.01 | 0.77 | 0.88 |
| cg02853428 | *TMEM106B* | -0.01 | 0.78 | 0.89 |
| cg07180834 | *PICALM* | 0.01 | 0.78 | 0.89 |
| cg01110620 | *RASAL1* | 0.01 | 0.78 | 0.89 |
| cg12487901 | *PCDHB14* | -0.01 | 0.78 | 0.89 |
| cg25263454 | *KLK14* | -0.01 | 0.78 | 0.89 |
| cg04895225 | *PICALM* | -0.01 | 0.78 | 0.89 |
| cg06295223 | *TSPAN14* | 0.01 | 0.79 | 0.89 |
| cg22565251 | *CLPTM1* | 0.01 | 0.79 | 0.89 |
| cg04502620 | *DOC2A* | -0.01 | 0.79 | 0.90 |
| cg02647874 | *SCARA3* | 0.01 | 0.79 | 0.90 |
| cg10139370 | *FUT5* | -0.01 | 0.80 | 0.90 |
| cg12358443 | *MCM7* | -0.01 | 0.80 | 0.90 |
| cg14316118 | *CCDC6* | -0.01 | 0.80 | 0.90 |
| cg25446361 | *HTRA1* | -0.01 | 0.80 | 0.90 |
| cg00579402 | *FUT6* | 0.01 | 0.80 | 0.90 |
| cg27525032 | *NKPD1* | 0.01 | 0.80 | 0.90 |
| cg01527459 | *DYDC2* | 0.01 | 0.80 | 0.90 |
| cg02944084 | *PNMT* | -0.01 | 0.81 | 0.91 |
| cg10967191 | *ZNF423* | 0.01 | 0.81 | 0.91 |
| cg25510838 | *MAFK* | 0.01 | 0.81 | 0.91 |
| cg09006514 | *BIN1* | -0.01 | 0.81 | 0.91 |
| cg06479902 | *EXOC3L2* | 0.01 | 0.81 | 0.91 |
| cg23404435 | *TMEM184A* | -0.01 | 0.81 | 0.91 |
| cg07435237 | *ARMC5* | 0.01 | 0.81 | 0.91 |
| cg21005607 | *MPP6* | -0.01 | 0.82 | 0.91 |
| cg23550016 | *STARD3* | -0.01 | 0.82 | 0.91 |
| cg05806717 | *NCK2* | 0.01 | 0.82 | 0.91 |
| cg10669449 | *ATG5* | -0.01 | 0.82 | 0.91 |
| cg01835620 | *CHRNE* | 0.01 | 0.83 | 0.92 |
| cg04168554 | *RAB11FIP3* | -0.01 | 0.83 | 0.92 |
| cg08707875 | *WDR26* | 0.01 | 0.83 | 0.92 |
| cg08246316 | *SRCAP* | 0.01 | 0.83 | 0.92 |
| cg07260927 | *HS3ST1* | -0.01 | 0.84 | 0.92 |
| cg15058036 | *SLC26A1* | -0.01 | 0.84 | 0.92 |
| cg06232205 | *GPR176* | -0.01 | 0.84 | 0.93 |
| cg16255729 | *RIN3* | 0.01 | 0.84 | 0.93 |
| cg05881698 | *NRTN* | -0.01 | 0.85 | 0.93 |
| cg27312916 | *SYTL2* | -0.01 | 0.85 | 0.93 |
| cg07868561 | *TTC17* | -0.01 | 0.85 | 0.93 |
| cg20822540 | *SLC2A7* | -0.01 | 0.85 | 0.93 |
| cg07041748 | *DOC2A* | -0.01 | 0.85 | 0.93 |
| cg18741372 | *PPP1R13L* | 0.00 | 0.85 | 0.93 |
| cg22647546 | *RAB10* | 0.00 | 0.85 | 0.93 |
| cg07773593 | *APOC1* | 0.00 | 0.86 | 0.93 |
| cg21005607 | *NPY* | 0.00 | 0.86 | 0.93 |
| cg25033993 | *ITGA2B* | 0.00 | 0.86 | 0.93 |
| cg05106699 | *PRDM7* | 0.00 | 0.86 | 0.93 |
| cg04853218 | *FBXO34* | 0.00 | 0.86 | 0.93 |
| cg12559685 | *CNTNAP1* | 0.00 | 0.86 | 0.94 |
| cg24060040 | *RNF43* | 0.00 | 0.86 | 0.94 |
| cg24616366 | *SIRPA* | 0.00 | 0.86 | 0.94 |
| cg16422492 | *TRIM72* | 0.00 | 0.87 | 0.94 |
| cg26675395 | *PRSS36* | 0.00 | 0.87 | 0.94 |
| cg19164175 | *VWA5B1* | 0.00 | 0.87 | 0.94 |
| cg06631810 | *EPHA1* | 0.00 | 0.87 | 0.94 |
| cg00303876 | *HNRNPAB* | 0.00 | 0.87 | 0.94 |
| cg25500444 | *ACHE* | 0.00 | 0.88 | 0.94 |
| cg03958363 | *TMEM184A* | 0.00 | 0.88 | 0.95 |
| cg23871276 | *MAFK* | 0.00 | 0.88 | 0.95 |
| cg09151754 | *ZNF232* | 0.00 | 0.89 | 0.95 |
| cg08843902 | *VAC14* | 0.00 | 0.89 | 0.95 |
| cg14570121 | *ERCC2* | 0.00 | 0.89 | 0.95 |
| cg16568360 | *TAOK2* | 0.00 | 0.89 | 0.95 |
| cg08447739 | *HTRA1* | 0.00 | 0.89 | 0.95 |
| cg11610460 | *RNF43* | 0.00 | 0.89 | 0.95 |
| cg25741533 | *NRTN* | 0.00 | 0.89 | 0.95 |
| cg16317516 | *MADD* | 0.00 | 0.89 | 0.95 |
| cg09339219 | *FZD2* | 0.00 | 0.90 | 0.95 |
| cg16661269 | *PAX3* | 0.00 | 0.90 | 0.95 |
| cg21201285 | *REXO1* | 0.00 | 0.90 | 0.96 |
| cg23707590 | *ZDHHC14* | 0.00 | 0.91 | 0.96 |
| cg24063856 | *CRHR1* | 0.00 | 0.91 | 0.96 |
| cg06521025 | *RAPSN* | 0.00 | 0.91 | 0.96 |
| cg05270750 | *PRDM7* | 0.00 | 0.91 | 0.96 |
| cg19767205 | *PCDH1* | 0.00 | 0.91 | 0.96 |
| cg05493751 | *GRB10* | 0.00 | 0.91 | 0.96 |
| cg22488857 | *ACHE* | 0.00 | 0.91 | 0.96 |
| cg07957995 | *CNIH3* | 0.00 | 0.91 | 0.96 |
| cg24704510 | *MED24* | 0.00 | 0.91 | 0.96 |
| cg04344923 | *MAFK* | 0.00 | 0.91 | 0.96 |
| cg04662983 | *HLA_E* | 0.00 | 0.92 | 0.97 |
| cg13704531 | *NCK2* | 0.00 | 0.92 | 0.97 |
| cg19499085 | *LRRFIP2* | 0.00 | 0.92 | 0.97 |
| cg10043954 | *PCDHB14* | 0.00 | 0.92 | 0.97 |
| cg02538169 | *GIGYF1* | 0.00 | 0.92 | 0.97 |
| cg00025823 | *ZNF232* | 0.00 | 0.93 | 0.97 |
| cg06295223 | *PRDM7* | 0.00 | 0.93 | 0.97 |
| cg24682159 | *BAG5* | 0.00 | 0.93 | 0.97 |
| cg09433588 | *KLF16* | 0.00 | 0.93 | 0.97 |
| cg05428706 | *SEMA4G* | 0.00 | 0.93 | 0.97 |
| cg00277334 | *TSPAN14* | 0.00 | 0.93 | 0.97 |
| cg09499421 | *STX1B* | 0.00 | 0.93 | 0.97 |
| cg08620751 | *KLF16* | 0.00 | 0.93 | 0.97 |
| cg24072947 | *IL20RB* | 0.00 | 0.94 | 0.97 |
| cg07871971 | *CNTNAP1* | 0.00 | 0.94 | 0.97 |
| cg02110529 | *TEX14* | 0.00 | 0.94 | 0.97 |
| cg11824827 | *PCGF3* | 0.00 | 0.94 | 0.97 |
| cg17514665 | *SERPINF2* | 0.00 | 0.94 | 0.98 |
| cg21221455 | *TPM1* | 0.00 | 0.94 | 0.98 |
| cg13879655 | *EPHX2* | 0.00 | 0.95 | 0.98 |
| cg00524708 | *PRSS36* | 0.00 | 0.95 | 0.98 |
| cg04326499 | *RUNX2* | 0.00 | 0.95 | 0.98 |
| cg13996522 | *TRAF3* | 0.00 | 0.95 | 0.98 |
| cg00694258 | *ZEB1* | 0.00 | 0.96 | 0.98 |
| cg04582938 | *UAP1L1* | 0.00 | 0.96 | 0.98 |
| cg03635300 | *NRTN* | 0.00 | 0.96 | 0.98 |
| cg04766076 | *APOC4* | 0.00 | 0.96 | 0.98 |
| cg27151362 | *DOC2A* | 0.00 | 0.96 | 0.99 |
| cg07862129 | *DYDC2* | 0.00 | 0.97 | 0.99 |
| cg09414983 | *TFAP2A* | 0.00 | 0.97 | 0.99 |
| cg21742836 | *PPP4C* | 0.00 | 0.97 | 0.99 |
| cg04382643 | *KLC3* | 0.00 | 0.97 | 0.99 |
| cg05192831 | *ASPHD1* | 0.00 | 0.97 | 0.99 |
| cg11926460 | *DGKD* | 0.00 | 0.98 | 0.99 |
| cg04501217 | *TMEM184A* | 0.00 | 0.98 | 0.99 |
| cg09764761 | *MAPT* | 0.00 | 0.98 | 0.99 |
| cg12683641 | *KLK14* | 0.00 | 0.98 | 0.99 |
| cg21496948 | *KLC1* | 0.00 | 0.98 | 0.99 |
| cg23520688 | *TRAF3* | 0.00 | 0.98 | 0.99 |
| cg25033993 | *FAM171A2* | 0.00 | 0.98 | 0.99 |
| cg10454568 | *AK7* | 0.00 | 0.98 | 0.99 |
| cg11936410 | *TPM1* | 0.00 | 0.98 | 0.99 |
| cg17365250 | *UFSP1* | 0.00 | 0.99 | 0.99 |
| cg16073427 | *CCDC85B* | 0.00 | 0.99 | 0.99 |
| cg06522456 | *BCAM* | 0.00 | 0.99 | 0.99 |
| cg18292394 | *MAT1A* | 0.00 | 0.99 | 0.99 |
| cg04018474 | *STX1B* | 0.00 | 0.99 | 0.99 |
| cg13351161 | *SCARA3* | 0.00 | 0.99 | 0.99 |
| cg04697562 | *TAOK2* | 0.00 | 0.99 | 0.99 |
| cg01140102 | *LMAN2* | 0.00 | 0.99 | 1.00 |
| cg02747950 | *RAB8B* | 0.00 | 1.00 | 1.00 |
| cg00091760 | *BTBD16* | 0.00 | 1.00 | 1.00 |

**Table S5. Associations between genetically predicted mRNA expression levels of 46 candidate target genes of identified 69 CpG sites and AD risk.**

| **CpG^1^** | **Chr** | **Position** | **DNA methylation and AD risk** | | | **DNA methylation and gene expression** | | **Gene expression and AD risk** | | | **Associated gene** |
| --- | --- | --- | --- | --- | --- | --- | --- | --- | --- | --- | --- |
|  |  |  | **Model** | **OR (95% CI)^2^** | ***P* value** | **Correlation coefficient** | ***P* value** | **Model** | **OR (95% CI)^2^** | ***P* value** |  |
| cg09070378 | 1 | 161,183,762 | FHS | 1.10 (1.07 ± 1.13) | 9.02 × 10^-7^ | -0.10 | 1.77 × 10^-3^ | BIOS | 0.85 (0.79 ± 0.91) | 1.19× 10^-3^ | ***NDUFS2*** |
|  |  |  |  |  |  |  |  | UTMOST | 0.87 (0.81 ± 0.93) | 1.27× 10^-2^ |  |
| cg07356342 | 1 | 161,183,820 | BIOS | 1.10 (1.07 ± 1.14) | 5.17 × 10^-8^ | -0.07 | 4.41 × 10^-2^ | BIOS | 0.85 (0.79 ± 0.91) | 1.19× 10^-3^ | ***NDUFS2*** |
|  |  |  | FHS | 1.19 (1.12 ± 1.26) | 7.89 × 10^-6^ |  |  | UTMOST | 0.87 (0.81 ± 0.93) | 1.27× 10^-2^ |  |
| cg05659526 | 1 | 161,184,528 | BIOS | 1.14 (1.09 ± 1.20) | 3.64 × 10^-5^ | -0.13 | 2.44 × 10^-5^ | UTMOST | 0.80 (0.74 ± 0.85) | 2.39× 10^-7^ | ***FCER1G*** |
|  |  |  |  |  |  |  |  | BIOS | 0.89 (0.85 ± 0.92) | 2.90× 10^-7^ |  |
| cg09106231 | 1 | 207,455,715 | BIOS | 0.94 (0.91 ± 0.97) | 5.21 × 10^-3^ | 0.11 | 2.82 × 10^-4^ | UTMOST | 1.04 (1.02 ± 1.06) | 1.46× 10^-3^ | *DGKQ* |
| cg05825950 | 1 | 207,669,576 | FHS | 1.11 (1.04 ± 1.17) | 4.97 × 10^-2^ | -0.09 | 5.43 × 10^-3^ | BIOS | 1.25 (1.12 ± 1.41) | 1.02× 10^-2^ | *CR1* |
| cg21110645 | 1 | 207,815,933 | BIOS | 1.02 (1.01 ± 1.03) | 2.26 × 10^-3^ | -0.16 | 3.02 × 10^-8^ | BIOS | 1.25 (1.12 ± 1.41) | 1.02× 10^-2^ | *CR1* |
|  |  |  | FHS | 1.03 (1.02 ± 1.05) | 9.53 × 10^-3^ |  |  |  |  |  |  |
| cg15595502 | 1 | 224,564,870 | FHS | 1.04 (1.02 ± 1.07) | 1.30 × 10^-2^ | -0.17 | 4.87 × 10^-9^ | UTMOST | 0.91 (0.87 ± 0.95) | 2.01× 10^-3^ | ***CNIH4*** |
|  |  |  | BIOS | 1.02 (1.01 ± 1.03) | 1.84 × 10^-2^ |  |  | BIOS | 0.92 (0.88 ± 0.95) | 1.36× 10^-3^ |  |
| cg26306976 | 2 | 9,564,901 | BIOS | 0.98 (0.97 ± 0.99) | 2.43 × 10^-4^ | 0.10 | 2.05 × 10^-3^ | UTMOST | 1.44 (1.18 ± 1.76) | 2.55× 10^-2^ | *CPSF3* |
|  |  |  | FHS | 0.96 (0.94 ± 0.98) | 3.17 × 10^-4^ |  |  |  |  |  |  |
| cg05208056 | 2 | 106,502,915 | BIOS | 1.13 (1.07 ± 1.19) | 4.34 × 10^-3^ | 0.12 | 2.31 × 10^-4^ | BIOS | 0.94 (0.90 ± 0.97) | 4.68× 10^-2^ | *TBX6* |
|  |  |  |  |  |  |  |  | UTMOST | 0.93 (0.90 ± 0.96) | 1.20× 10^-3^ |  |
| cg08563189 | 2 | 127,780,654 | FHS | 1.40 (1.27 ± 1.54) | 3.40 × 10^-8^ | 0.07 | 4.30 × 10^-2^ | BIOS | 1.28 (1.21 ± 1.34) | 2.20× 10^-16^ | ***BIN1*** |
|  |  |  | BIOS | 1.13 (1.09 ± 1.17) | 3.62 × 10^-8^ |  |  | UTMOST | 1.28 (1.19 ± 1.38) | 1.45× 10^-7^ |  |
| cg19153828 | 2 | 127,782,651 | FHS | 1.06 (1.03 ± 1.08) | 1.17 × 10^-3^ | 0.09 | 4.31 × 10^-3^ | BIOS | 1.28 (1.21 ± 1.34) | 2.20× 10^-16^ | ***BIN1*** |
|  |  |  | BIOS | 1.02 (1.01 ± 1.04) | 4.40 × 10^-3^ |  |  | UTMOST | 1.28 (1.19 ± 1.38) | 1.45× 10^-7^ |  |
| cg19590598 | 2 | 127,782,813 | FHS | 1.05 (1.03 ± 1.08) | 3.86 × 10^-4^ | 0.10 | 2.74 × 10^-3^ | BIOS | 1.28 (1.21 ± 1.34) | 2.20× 10^-16^ | ***BIN1*** |
|  |  |  | BIOS | 1.02 (1.01 ± 1.03) | 6.54 × 10^-3^ |  |  | UTMOST | 1.28 (1.19 ± 1.38) | 1.45× 10^-7^ |  |
| cg22376361 | 2 | 127,815,133 | FHS | 1.60 (1.39 ± 1.84) | 1.19 × 10^-7^ | 0.10 | 3.51 × 10^-3^ | BIOS | 1.28 (1.21 ± 1.34) | 2.20× 10^-16^ | ***BIN1*** |
|  |  |  |  |  |  |  |  | UTMOST | 1.28 (1.19 ± 1.38) | 1.45× 10^-7^ |  |
| cg00436254 | 2 | 127,862,614 | FHS | 1.10 (1.06 ± 1.13) | 7.49 × 10^-5^ | 0.13 | 2.59 × 10^-5^ | BIOS | 1.28 (1.21 ± 1.34) | 2.20× 10^-16^ | ***BIN1*** |
|  | 2 | 127,862,614 | BIOS | 1.03 (1.01 ± 1.04) | 1.24 × 10^-2^ |  |  | UTMOST | 1.28 (1.19 ± 1.38) | 1.45× 10^-7^ |  |
| cg14012546 | 2 | 233,981,788 | BIOS | 0.81 (0.76 ± 0.85) | 4.02 × 10^-11^ | -0.12 | 6.06 × 10^-5^ | BIOS | 0.74 (0.68 ± 0.81) | 7.75× 10^-8^ | *INPP5D* |
|  |  |  |  |  |  |  |  | UTMOST | 0.70 (0.64 ± 0.77) | 4.14× 10^-11^ |  |
| cg17763743 | 3 | 9,343,967 | BIOS | 0.97 (0.95 ± 0.99) | 4.83 × 10^-2^ | -0.12 | 1.59 × 10^-4^ | BIOS | 1.10 (1.04 ± 1.16) | 4.18× 10^-2^ | ***THUMPD3*** |
| cg06284479 | 3 | 37,173,546 | FHS | 1.09 (1.05 ± 1.13) | 5.85 × 10^-3^ | -0.11 | 9.22 × 10^-4^ | BIOS | 0.89 (0.83 ± 0.94) | 1.64× 10^-2^ | ***LRRFIP2*** |
|  | 3 | 37,173,546 | BIOS | 1.05 (1.02 ± 1.07) | 1.39 × 10^-2^ |  |  | UTMOST | 0.89 (0.84 ± 0.94) | 3.15× 10^-3^ |  |
| cg15934958 | 3 | 37,212,084 | BIOS | 1.04 (1.02 ± 1.07) | 1.22 × 10^-2^ | -0.12 | 5.76 × 10^-5^ | BIOS | 0.89 (0.83 ± 0.94) | 1.64× 10^-2^ | ***LRRFIP2*** |
|  |  |  | FHS | 1.05 (1.02 ± 1.07) | 1.42 × 10^-2^ |  |  | UTMOST | 0.89 (0.84 ± 0.94) | 3.15× 10^-3^ |  |
| cg22985146 | 3 | 37,219,077 | FHS | 0.95 (0.92 ± 0.97) | 1.52 × 10^-2^ | 0.08 | 1.93 × 10^-2^ | BIOS | 0.89 (0.83 ± 0.94) | 1.64× 10^-2^ | *LRRFIP2* |
|  |  |  |  |  |  |  |  | UTMOST | 0.89 (0.84 ± 0.94) | 3.15× 10^-3^ |  |
| cg06148264 | 6 | 2,841,468 | FHS | 0.91 (0.87 ± 0.95) | 1.07 × 10^-2^ | -0.08 | 1.53 × 10^-2^ | UTMOST | 0.77 (0.67 ± 0.88) | 8.67× 10^-3^ | *SERPINB1* |
| cg23963071 | 6 | 2,901,712 | BIOS | 1.02 (1.01 ± 1.02) | 3.83 × 10^-3^ | -0.24 | 1.90 × 10^-14^ | BIOS | 0.91 (0.88 ± 0.95) | 7.92× 10^-4^ | ***SERPINB9*** |
| cg02130027 | 6 | 47,444,894 | FHS | 1.15 (1.11 ± 1.20) | 1.94 × 10^-8^ | 0.15 | 4.81 × 10^-7^ | UTMOST | 1.94 (1.54 ± 2.42) | 3.86× 10^-6^ | ***CD2AP*** |
|  |  |  | BIOS | 1.07 (1.05 ± 1.09) | 1.57 × 10^-7^ |  |  |  |  |  |  |
| cg20196966 | 6 | 47,445,060 | FHS | 1.25 (1.17 ± 1.33) | 3.77 × 10^-8^ | 0.12 | 1.49 × 10^-4^ | UTMOST | 1.94 (1.54 ± 2.42) | 3.86× 10^-6^ | ***CD2AP*** |
|  |  |  | BIOS | 1.09 (1.06 ± 1.13) | 8.85 × 10^-7^ |  |  |  |  |  |  |
| cg20172563 | 6 | 47,487,173 | FHS | 1.10 (1.07 ± 1.13) | 2.18 × 10^-8^ | 0.23 | 1.903 × 10^-14^ | UTMOST | 1.94 (1.54 ± 2.42) | 3.86× 10^-6^ | ***CD2AP*** |
|  |  |  | BIOS | 1.04 (1.02 ± 1.05) | 4.26 × 10^-6^ |  |  |  |  |  |  |
| cg15095913 | 7 | 1,576,976 | BIOS | 0.90 (0.85 ± 0.95) | 2.21 × 10^-2^ | 0.07 | 4.35 × 10^-2^ | BIOS | 0.72 (0.61 ± 0.86) | 2.03× 10^-2^ | *MAFK* |
| cg22500518 | 7 | 1,585,359 | FHS | 1.18 (1.08 ± 1.29) | 2.03 × 10^-2^ | -0.08 | 1.22 × 10^-2^ | BIOS | 1.12 (1.05 ± 1.19) | 3.33× 10^-2^ | *TMEM184A* |
| cg19800032 | 7 | 12,134,189 | FHS | 0.94 (0.92 ± 0.97) | 4.48 × 10^-3^ | -0.08 | 2.19 × 10^-2^ | UTMOST | 0.81 (0.75 ± 0.88) | 1.48× 10^-4^ | *TMEM106B* |
|  |  |  | BIOS | 0.97 (0.96 ± 0.99) | 2.14 × 10^-2^ |  |  |  |  |  |  |
| cg06189038 | 7 | 99,767,134 | BIOS | 1.13 (1.06 ± 1.21) | 1.95 × 10^-2^ | -0.10 | 1.96 × 10^-3^ | UTMOST | 0.01 (0.01 ± 0.00) | 6.33× 10^-5^ | ***GAL3ST4*** |
| cg11291798 | 7 | 143,103,859 | FHS | 1.13 (1.06 ± 1.21) | 2.97 × 10^-2^ | -0.08 | 2.25 × 10^-2^ | BIOS | 0.83 (0.75 ± 0.92) | 2.39× 10^-2^ | ***EPHA1*** |
| cg14709253 | 8 | 17,519,419 | BIOS | 0.97 (0.95 ± 0.98) | 9.98 × 10^-3^ | 0.19 | 5.27 × 10^-11^ | UTMOST | 0.76 (0.67 ± 0.86) | 2.23× 10^-3^ | ***MTUS1*** |
|  |  |  |  |  |  |  |  | BIOS | 0.93 (0.90 ± 0.96) | 7.94× 10^-3^ |  |
| cg12548824 | 8 | 17,554,892 | FHS | 1.03 (1.02 ± 1.05) | 2.52 × 10^-2^ | -0.29 | 1.90 × 10^-14^ | UTMOST | 0.76 (0.67 ± 0.86) | 2.23× 10^-3^ | ***MTUS1*** |
|  |  |  |  |  |  |  |  | BIOS | 0.93 (0.90 ± 0.96) | 7.94× 10^-3^ |  |
| cg01993952 | 8 | 17,554,904 | FHS | 1.04 (1.02 ± 1.06) | 3.04 × 10^-2^ | -0.20 | 2.21 × 10^-12^ | UTMOST | 0.76 (0.67 ± 0.86) | 2.23× 10^-3^ | ***MTUS1*** |
|  |  |  |  |  |  |  |  | BIOS | 0.93 (0.90 ± 0.96) | 7.94× 10^-3^ |  |
| cg22099723 | 8 | 27,348,453 | FHS | 1.11 (1.06 ± 1.16) | 3.29 × 10^-4^ | 0.08 | 1.28 × 10^-2^ | BIOS | 1.14 (1.06 ± 1.24) | 4.70× 10^-2^ | ***EPHX2*** |
|  |  |  | BIOS | 1.02 (1.01 ± 1.03) | 3.66 × 10^-2^ |  |  |  |  |  |  |
| cg21666367 | 8 | 27,450,279 | BIOS | 1.06 (1.03 ± 1.08) | 3.90 × 10^-4^ | 0.11 | 3.36 × 10^-4^ | UTMOST | 1.12 (1.06 ± 1.18) | 3.15× 10^-3^ | ***EPHX2*** |
|  |  |  |  |  |  |  |  | BIOS | 1.14 (1.06 ± 1.24) | 4.70× 10^-2^ |  |
| cg24794833 | 8 | 27,450,748 | FHS | 0.81 (0.76 ± 0.87) | 3.82 × 10^-6^ | -0.10 | 2.78 × 10^-3^ | UTMOST | 1.12 (1.06 ± 1.18) | 3.15× 10^-3^ | ***EPHX2*** |
|  |  |  |  |  |  |  |  | BIOS | 1.14 (1.06 ± 1.24) | 4.70× 10^-2^ |  |
| cg26343298 | 8 | 95,960,752 | FHS | 1.08 (1.05 ± 1.11) | 1.81 × 10^-5^ | -0.15 | 1.22 × 10^-6^ | UTMOST | 0.69 (0.61 ± 0.78) | 4.81× 10^-6^ | ***TP53INP1*** |
|  |  |  | BIOS | 1.04 (1.02 ± 1.05) | 6.98 × 10^-5^ |  |  |  |  |  |  |
| cg05457903 | 8 | 145,052,304 | BIOS | 1.05 (1.02 ± 1.08) | 3.42 × 10^-2^ | 0.08 | 1.49 × 10^-2^ | BIOS | 0.67 (0.54 ± 0.83) | 1.79× 10^-2^ | *PARP10* |
| cg16915659 | 10 | 60,032,665 | BIOS | 1.05 (1.02 ± 1.07) | 8.16 × 10^-3^ | 0.16 | 7.43 × 10^-8^ | BIOS | 1.06 (1.03 ± 1.10) | 4.04× 10^-3^ | ***CISD1*** |
|  |  |  |  |  |  |  |  | UTMOST | 1.06 (1.03 ± 1.10) | 4.26× 10^-3^ |  |
| cg15320596 | 10 | 61,604,738 | BIOS | 0.97 (0.96 ± 0.99) | 2.78 × 10^-2^ | 0.10 | 2.37 × 10^-3^ | BIOS | 0.60 (0.48 ± 0.75) | 1.35× 10^-3^ | ***CCDC6*** |
|  |  |  | FHS | 0.96 (0.93 ± 0.98) | 4.83 × 10^-2^ |  |  | UTMOST | 0.86 (0.79 ± 0.93) | 2.25× 10^-2^ |  |
| cg23858360 | 10 | 82,213,490 | BIOS | 0.94 (0.92 ± 0.96) | 1.62 × 10^-5^ | 0.08 | 2.42 × 10^-2^ | UTMOST | 0.76 (0.67 ± 0.87) | 4.83× 10^-3^ | ***TSPAN14*** |
|  |  |  | FHS | 0.90 (0.86 ± 0.94) | 3.52 × 10^-4^ |  |  |  |  |  |  |
| cg16178415 | 10 | 82,265,445 | FHS | 1.09 (1.05 ± 1.13) | 6.82 × 10^-4^ | -0.13 | 4.10 × 10^-5^ | UTMOST | 0.76 (0.67 ± 0.87) | 4.83× 10^-3^ | ***TSPAN14*** |
|  |  |  | BIOS | 1.05 (1.03 ± 1.08) | 1.37 × 10^-3^ |  |  |  |  |  |  |
| cg24590430 | 10 | 99,097,076 | BIOS | 1.02 (1.01 ± 1.03) | 3.65 × 10^-2^ | -0.10 | 3.31 × 10^-3^ | BIOS | 0.91 (0.87 ± 0.96) | 2.47× 10^-2^ | ***FRAT2*** |
|  |  |  | FHS | 1.03 (1.01 ± 1.05) | 4.35 × 10^-2^ |  |  | UTMOST | 0.77 (0.67 ± 0.88) | 1.65× 10^-2^ |  |
| cg00091760 | 10 | 124,131,072 | BIOS | 0.83 (0.77 ± 0.90) | 1.00 × 10^-3^ | -0.10 | 3.40 × 10^-3^ | UTMOST | 1.12 (1.05 ± 1.19) | 3.34× 10^-2^ | ***PLEKHA1*** |
|  |  |  |  |  |  |  |  | BIOS | 1.28 (1.14 ± 1.44) | 3.11× 10^-3^ |  |
| cg07675031 | 11 | 47,399,893 | FHS | 1.30 (1.15 ± 1.46) | 3.71 × 10^-3^ | 0.08 | 1.78 × 10^-2^ | BIOS | 0.83 (0.75 ± 0.92) | 2.36× 10^-2^ | *RNF43* |
| cg04353769 | 11 | 59,951,557 | BIOS | 0.69 (0.64 ± 0.75) | 1.22 × 10^-15^ | -0.10 | 1.93 × 10^-3^ | BIOS | 1.65 (1.47 ± 1.85) | 1.77× 10^-13^ | ***MS4A6A*** |
| cg06881914 | 11 | 59,951,663 | FHS | 0.86 (0.83 ± 0.89) | 6.25 × 10^-15^ | -0.19 | 1.43 × 10^-10^ | BIOS | 1.65 (1.47 ± 1.85) | 1.77× 10^-13^ | ***MS4A6A*** |
|  |  |  | BIOS | 0.91 (0.89 ± 0.93) | 2.76 × 10^-12^ |  |  |  |  |  |  |
| cg04422903 | 11 | 64,108,550 | FHS | 1.03 (1.02 ± 1.05) | 4.42 × 10^-2^ | -0.10 | 3.20 × 10^-3^ | UTMOST | 0.88 (0.83 ± 0.95) | 2.25× 10^-2^ | ***CCDC88B*** |
| cg01120308 | 11 | 85,780,971 | BIOS | 0.87 (0.82 ± 0.92) | 3.61 × 10^-4^ | -0.14 | 3.13 × 10^-6^ | BIOS | 1.11 (1.07 ± 1.15) | 5.96× 10^-5^ | ***CHRNE*** |
|  |  |  |  |  |  |  |  | UTMOST | 1.10 (1.07 ± 1.15) | 3.21× 10^-5^ |  |
| cg05931265 | 11 | 122,527,736 | BIOS | 0.92 (0.88 ± 0.96) | 3.10 × 10^-2^ | -0.17 | 1.83 × 10^-8^ | UTMOST | 0.84 (0.78 ± 0.91) | 1.73× 10^-3^ | *UBASH3B* |
| cg11107966 | 14 | 92,927,875 | FHS | 1.05 (1.03 ± 1.07) | 7.25 × 10^-6^ | -0.12 | 7.72 × 10^-5^ | BIOS | 0.92 (0.89 ± 0.95) | 1.01× 10^-4^ | ***SLC24A4*** |
|  |  |  | BIOS | 1.02 (1.01 ± 1.03) | 1.57 × 10^-4^ |  |  | UTMOST | 0.82 (0.78 ± 0.86) | 2.05× 10^-11^ |  |
| cg14021523 | 14 | 92,959,873 | FHS | 1.13 (1.06 ± 1.21) | 2.26 × 10^-2^ | -0.14 | 1.29 × 10^-6^ | BIOS | 0.92 (0.89 ± 0.95) | 1.01× 10^-4^ | ***SLC24A4*** |
|  |  |  |  |  |  |  |  | UTMOST | 0.82 (0.78 ± 0.86) | 2.05× 10^-11^ |  |
| cg05200313 | 14 | 92,960,827 | BIOS | 1.14 (1.08 ± 1.21) | 3.52 × 10^-4^ | -0.16 | 2.36 × 10^-8^ | BIOS | 0.92 (0.89 ± 0.95) | 1.01× 10^-4^ | ***SLC24A4*** |
|  |  |  | FHS | 1.20 (1.11 ± 1.31) | 3.62 × 10^-3^ |  |  | UTMOST | 0.82 (0.78 ± 0.86) | 2.05× 10^-11^ |  |
| cg25647583 | 15 | 91,427,184 | BIOS | 0.93 (0.89 ± 0.97) | 4.95 × 10^-2^ | -0.26 | 1.90 × 10^-14^ | UTMOST | 1.19 (1.08 ± 1.30) | 2.25× 10^-2^ | ***FES*** |
| cg05676562 | 16 | 30,102,457 | FHS | 1.11 (1.07 ± 1.16) | 2.59 × 10^-4^ | -0.08 | 1.24 × 10^-2^ | BIOS | 0.94 (0.90 ± 0.97) | 4.68× 10^-2^ | ***TBX6*** |
|  |  |  | BIOS | 1.03 (1.01 ± 1.05) | 2.63 × 10^-2^ |  |  | UTMOST | 0.93 (0.90 ± 0.96) | 1.20× 10^-3^ |  |
| cg26709300 | 16 | 30,106,682 | BIOS | 0.95 (0.93 ± 0.97) | 1.20 × 10^-5^ | 0.10 | 3.29 × 10^-3^ | BIOS | 0.82 (0.76 ± 0.87) | 1.89× 10^-6^ | ***YPEL3*** |
|  |  |  |  |  |  |  |  | UTMOST | 0.87 (0.83 ± 0.91) | 8.15× 10^-7^ |  |
| cg02335376 | 16 | 30,124,880 | BIOS | 0.90 (0.86 ± 0.94) | 2.66 × 10^-4^ | -0.15 | 6.23 × 10^-7^ | UTMOST | 1.07 (1.03 ± 1.10) | 1.23× 10^-2^ | ***MAPK3*** |
|  |  |  |  |  |  |  |  | BIOS | 1.12 (1.06 ± 1.17) | 1.19× 10^-3^ |  |
| cg08464513 | 16 | 30,136,024 | FHS | 0.88 (0.84 ± 0.93) | 2.05 × 10^-4^ | -0.11 | 8.45 × 10^-4^ | UTMOST | 1.07 (1.03 ± 1.10) | 1.23× 10^-2^ | ***MAPK3*** |
|  |  |  | BIOS | 0.93 (0.90 ± 0.96) | 7.61 × 10^-4^ |  |  | BIOS | 1.12 (1.06 ± 1.17) | 1.19× 10^-3^ |  |
| cg00249205 | 16 | 31,012,263 | FHS | 0.94 (0.91 ± 0.96) | 5.85 × 10^-3^ | 0.15 | 3.64 × 10^-7^ | UTMOST | 0.81 (0.75 ± 0.88) | 1.48× 10^-4^ | ***TMEM106B*** |
| cg06233904 | 16 | 31,044,135 | BIOS | 1.10 (1.060 ± 1.14) | 1.41 × 10^-3^ | -0.08 | 1.42 × 10^-2^ | BIOS | 0.87 (0.81 ± 0.94) | 1.65× 10^-2^ | ***STX4*** |
|  |  |  | FHS | 1.24 (1.13 ± 1.37) | 2.04 × 10^-3^ |  |  |  |  |  |  |
| cg07404961 | 16 | 31,049,270 | FHS | 0.85 (0.79 ± 0.91) | 6.47 × 10^-4^ | 0.09 | 4.28 × 10^-3^ | BIOS | 0.87 (0.81 ± 0.94) | 1.65× 10^-2^ | ***STX4*** |
|  |  |  | BIOS | 0.94 (0.92 ± 0.97) | 1.14 × 10^-3^ |  |  |  |  |  |  |
| cg03984055 | 16 | 31,117,318 | BIOS | 1.12 (1.07 ± 1.18) | 4.88 × 10^-4^ | -0.11 | 3.06 × 10^-4^ | BIOS | 1.27 (1.16 ± 1.40) | 6.33× 10^-5^ | *BCKDK* |
|  |  |  | FHS | 1.37 (1.20 ± 1.57) | 9.23 × 10^-4^ |  |  | UTMOST | 2.62 (1.89 ± 3.64) | 3.31× 10^-6^ |  |
| cg19048010 | 17 | 28,084,996 | FHS | 1.07 (1.03 ± 1.11) | 3.58 × 10^-2^ | 0.16 | 1.05 × 10^-7^ | UTMOST | 1.28 (1.11 ± 1.48) | 3.62× 10^-2^ | ***SSH2*** |
| cg00685795 | 17 | 40,713,781 | FHS | 1.12 (1.06 ± 1.20) | 2.96 × 10^-2^ | 0.10 | 1.56 × 10^-3^ | BIOS | 1.44 (1.21 ± 1.73) | 6.42× 10^-3^ | ***COASY*** |
|  |  |  | BIOS | 1.06 (1.03 ± 1.01) | 4.80 × 10^-2^ |  |  |  |  |  |  |
| cg04266202 | 17 | 56,352,895 | FHS | 1.16 (1.08 ± 1.24) | 5.90 × 10^-3^ | -0.19 | 1.43 × 10^-10^ | BIOS | 0.85 (0.77 ± 0.93) | 2.73× 10^-2^ | ***MPO*** |
| cg11015549 | 19 | 1,026,207 | FHS | 0.81 (0.72 ± 0.90) | 2.56 × 10^-2^ | -0.19 | 3.63 × 10^-11^ | UTMOST | 1.11 (1.05 ± 1.18) | 3.31× 10^-2^ | ***CNN2*** |
| cg01496416 | 19 | 45,147,715 | FHS | 0.82 (0.77 ± 0.87) | 5.17 × 10^-8^ | -0.09 | 7.87 × 10^-3^ | UTMOST | 0.84 (0.79 ± 0.90) | 1.01× 10^-4^ | *PVR* |
|  |  |  | BIOS | 0.91 (0.89 ± 0.94) | 8.87 × 10^-8^ |  |  |  |  |  |  |
| cg04401876 | 19 | 45,445,449 | BIOS | 1.12 (1.07 ± 1.16) | 7.38 × 10^-5^ | 0.08 | 1.71 × 10^-2^ | UTMOST | 1.12 (1.06 ± 1.18) | 3.15× 10^-3^ | ***EPHX2*** |
|  |  |  |  |  |  |  |  | BIOS | 1.14 (1.06 ± 1.24) | 4.70× 10^-2^ |  |
| cg01674009 | 19 | 45,655,294 | BIOS | 1.11 (1.08 ± 1.14) | 5.76 × 10^-9^ | -0.07 | 4.75 × 10^-2^ | BIOS | 1.55 (1.32 ± 1.82) | 3.93× 10^-5^ | *NKPD1* |
|  |  |  | FHS | 1.27 (1.17 ± 1.37) | 7.42 × 10^-6^ |  |  |  |  |  |  |
| cg16210447 | 20 | 54,987,076 | BIOS | 1.10 (1.07 ± 1.13) | 5.43 × 10^-8^ | -0.15 | 5.27 × 10^-7^ | UTMOST | 4.39 (2.85 ± 6.76) | 1.92× 10^-8^ | *CASS4* |
|  |  |  | FHS | 1.41 (1.26 ± 1.57) | 1.50 × 10^-6^ |  |  |  |  |  |  |

^1^BIOS: Biobank-based Integrative Omics Studies; Chr: chromosome; CI: confidence interval; CpG: CpG sites; FHS: Framingham Heart Study; OR: odds ratio per SD increase in genetically predicated DNA methylation level (continuous variable); *P* value: *P* value after false discovery rate (FDR) correction; UTR: untranslated region; ^2^MetaXcan was used to estimate ORs, 95% CIs and *P* value. All statistical tests were two-sided; ^3^the bold genes shown consistent directions of associations across DNA methylation, gene expression and AD risk.

**Table S6. Top ten canonical pathways of the genes that consistent association directions for the methylation-gene expression-AD risk.**

| **Ingenuity Canonical Pathways** | ***P* value** | **Molecules** |
| --- | --- | --- |
| Melatonin Degradation III | 1.32 × 10^-3^ | MPO |
| Semaphorin Signaling in Neurons | 2.95 × 10^-3^ | FES, MAPK3 |
| Neutrophil degranulation | 3.47 × 10^-3^ | CNN2, FCER1G, MPO, TSPAN14 |
| Role of JAK1 and JAK3 in γc Cytokine Signaling | 3.89 × 10^-3^ | FES, MAPK3 |
| Coenzyme A Biosynthesis | 3.98 × 10^-3^ | COASY |
| L-cysteine Degradation III | 3.98 × 10^-3^ | CISD1 |
| TREM1 Signaling | 4.68 × 10^-3^ | MAPK3, MPO |
| Fc Epsilon RI Signaling | 1.10 × 10^-2^ | FCER1G, MAPK3 |
| IL-15 Production | 1.15 × 10^-2^ | EPHA1, FES |
| Insulin Receptor Signaling | 1.48 × 10^-2^ | MAPK3, STX4 |

**Table S7.** **Top ten disease and biological functions categories** **of** **the genes that consistent association directions for the methylation-gene expression-AD risk.**

| **Categories** | **Diseases/Functions Annotation** | ***P* Value** | **Molecules** |
| --- | --- | --- | --- |
| Neurological Disease, Organismal Injury and Abnormalities, Psychological Disorders | Dementia | 7.46 × 10^-6^ | TMEM106B, EPHA1, CD2AP, CHRNE, MPO, BIN1, MS4A6A, EPHX2, MAPK3 |
| Cellular Compromise, Inflammatory Response | Degranulation of phagocytes | 8.21 × 10^-6^ | MPO, FCER1G, FES, STX4, MAPK3 |
| Metabolic Disease, Neurological Disease, Organismal Injury and Abnormalities, Psychological Disorders | Late-onset Alzheimer disease | 1.02 × 10^-5^ | EPHA1, CD2AP, BIN1 |
| Cellular Compromise, Inflammatory Response | Degranulation of myeloid cells | 1.09 × 10^-5^ | MPO, FCER1G, FES, STX4, MAPK3 |
| Neurological Disease, Organismal Injury and Abnormalities, Psychological Disorders | Degenerative dementia | 2.86 × 10^-5^ | EPHA1, CD2AP, CHRNE, MPO, BIN1, MS4A6A, EPHX2, MAPK3 |
| Cellular Compromise, Hypersensitivity Response, Inflammatory Response | Degranulation of mast cells | 9.27 × 10^-5^ | FCER1G, FES, STX4, MAPK3 |
| Cell-To-Cell Signaling and Interaction, Hematological System Development and Function, Immune Cell Trafficking, Inflammatory Response | Priming of mononuclear leukocytes | 9.52 × 10^-5^ | SERPINB9, FCER1G, MAPK3 |
| Hematological System Development and Function, Inflammatory Response, Tissue Morphology | Quantity of monocytes | 1.19 × 10^-4^ | MPO, FES, CNN2, EPHX2 |
| Metabolic Disease, Neurological Disease, Organismal Injury and Abnormalities, Psychological Disorders | Alzheimer disease | 1.85 × 10^-4^ | EPHA1, CD2AP, CHRNE, MPO, BIN1, MS4A6A, MAPK3 |
| Cellular Development, Cellular Growth and Proliferation, Hematological System Development and Function, Lymphoid Tissue Structure and Development | Proliferation of immune cells | 2.32 × 10^-4^ | CCDC88B, CD2AP, SERPINB9, PLEKHA1, FCER1G, TP53INP1, CNN2, MAPK3 |

**Table S8. Networks of the genes that consistent association directions for the methylation-gene expression-AD risk.**

| **Molecules in Network** | **Score** | **Focus Molecules** | **Top Diseases and Functions** |
| --- | --- | --- | --- |
| Akt, BCR (complex), **BIN1**, **CCDC6**, **CD2AP**, CD3, **CNIH4**, **CNN2**, **COASY**, **EPHA1**, **EPHX2**, ERK, ERK1/2, **FCER1G**, **FES**, Fcer1, Histone h3, IgG, Igm, Immunoglobulin, **MAPK3**, **MS4A6A**, **MTUS1**, Mek, NFkB (complex), PI3K (complex), **PLEKHA1**, PTK, **STX4**, **TBX6**, TCR, **TMEM106B**, **TP53INP1**, **YPEL3**, p85 (pik3r) | 54 | 19 | Metabolic Disease, Neurological Disease, Organismal Injury and Abnormalities |
| ACTB, **CCDC88B**, **CHRNE**, **CISD1**, Ca^2+^, D-glyceraldehyde, **FRAT2**, **GAL3ST4**, ITGA3, Insulin, KRAS, L-glutamic acid 5-methyl ester, **LRRFIP2**, **MPO**, **NDUFS2**, PRMT5, Ppp1r15a, S-Farn-Me KRAS4B:CALM1:4xCa^2+^, S-Farn-Me KRAS4B:CALM1:4xCa^2+^:PDE6D, S-Farn-Me KRAS4B:CALM1:4xCa^2+^:PDE6D:ARL2:GTP, **SERPINB9**, **SLC24A4**, **SSH2**, Siah1b, **THUMPD3**, TNF, TP53, TP53 Tetramer:TTC5:EP300:JMY:PRMT5, **TSPAN14**, mead acid, miR-324-3p (miRNAs w/seed CCACUGC), mir-432, mir-491, mir-650, platelet activating factor phosphatidate | 33 | 13 | Cellular Compromise, Inflammatory Response, Drug Metabolism |


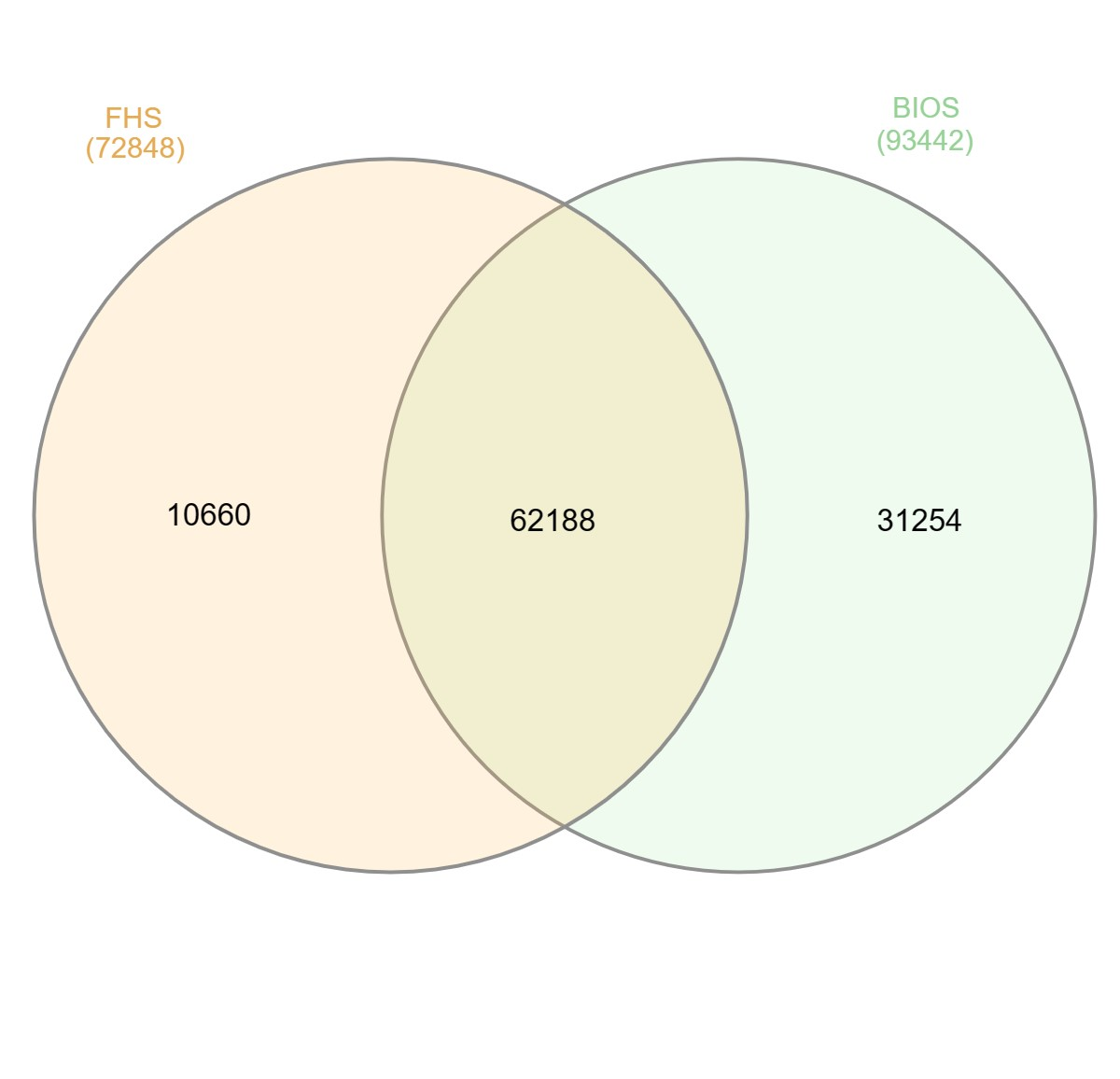


**Figure S1. Venn diagram of CpG sites in both Framingham Heart Study (FHS) and Biobank-based integrative omics study (BIOS) models.**


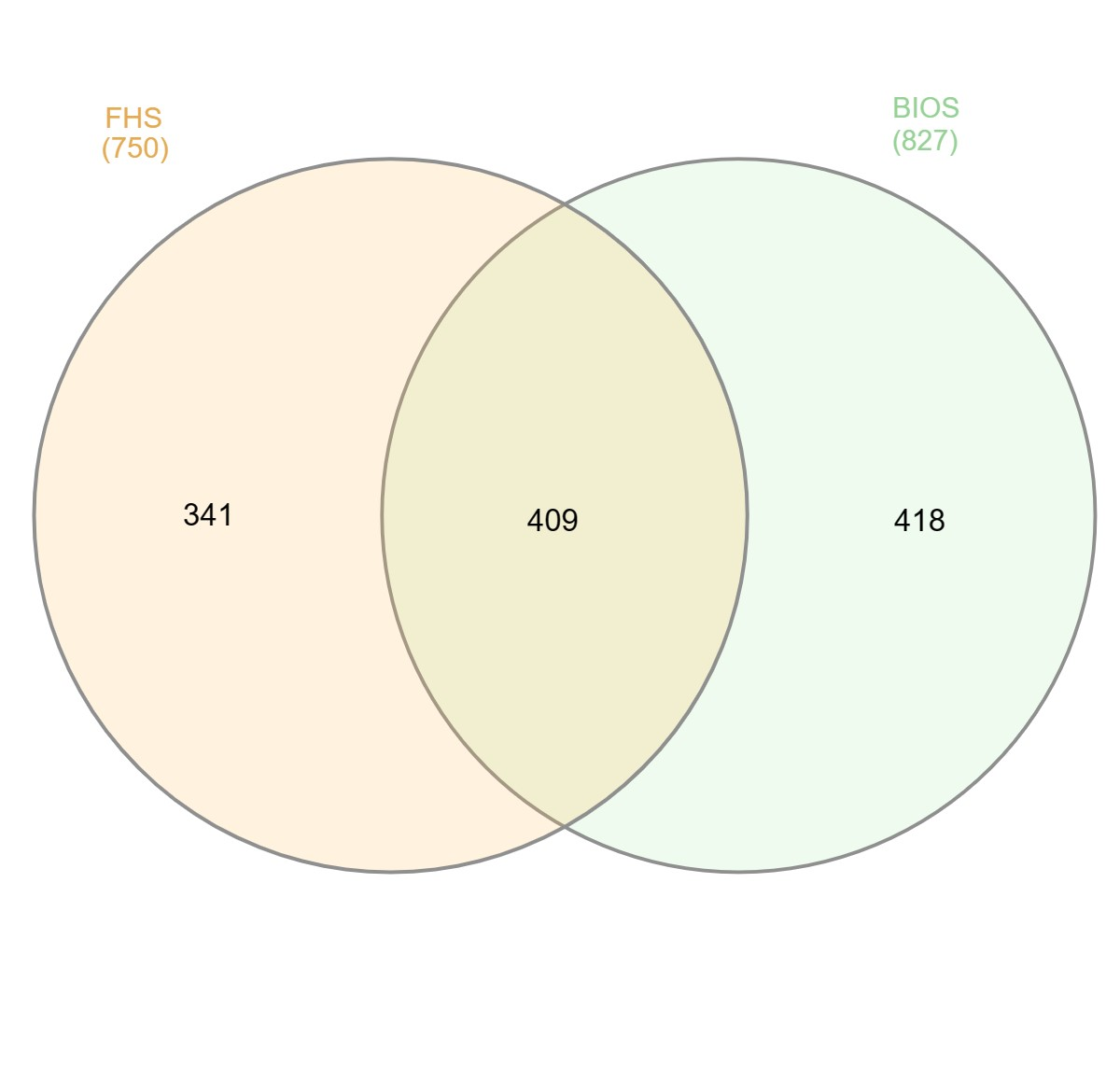
**Figure S2. Venn diagram of significant CpG sites in both Framingham Heart Study (FHS) and Biobank-based integrative omics study (BIOS) models.**


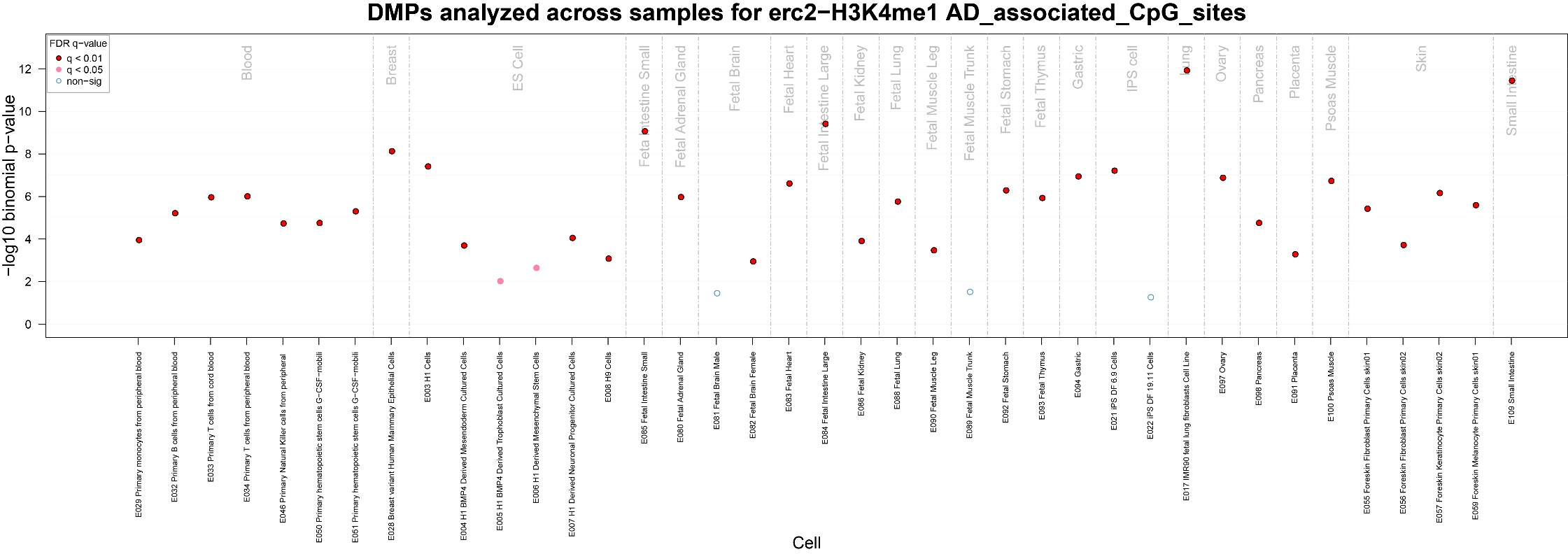


**Figure S3. Enrichment of AD associated CpG sites in regions overlapping H3K4me1 markers from the consolidated Roadmap Epigenomics data.**


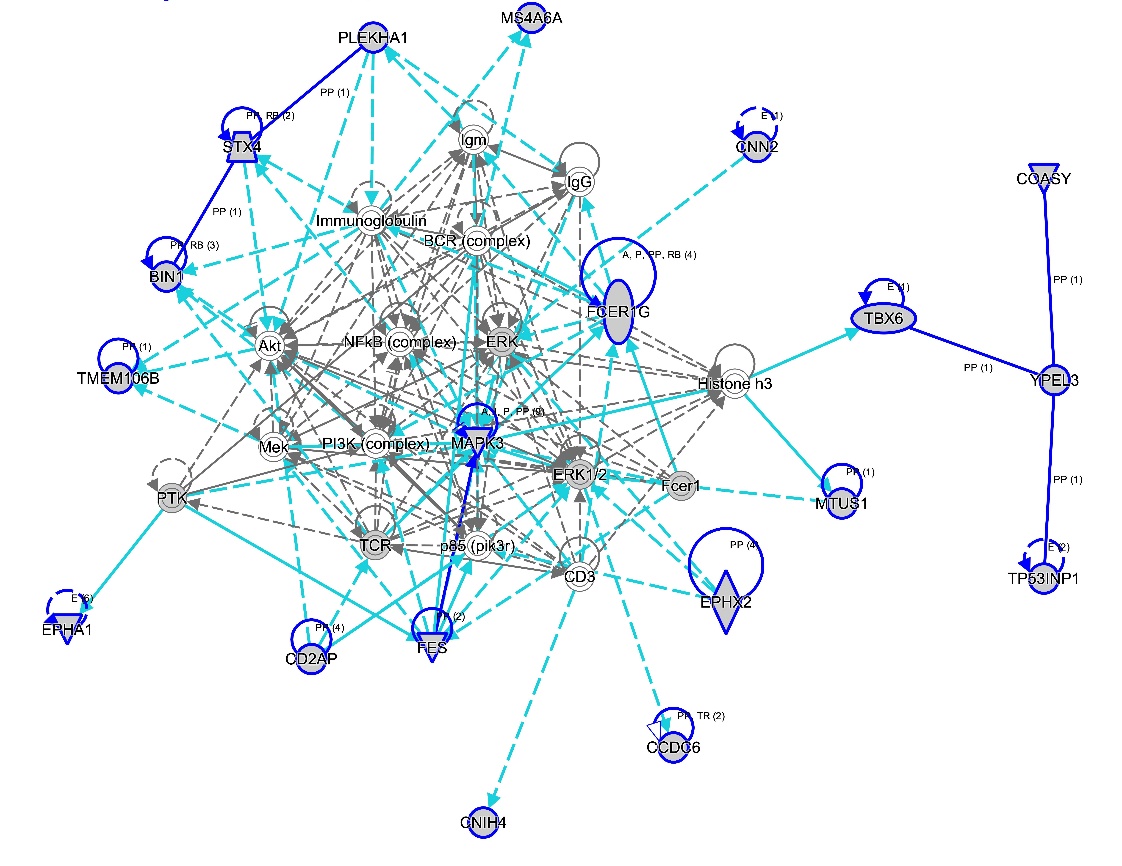


**B**

**A**

**
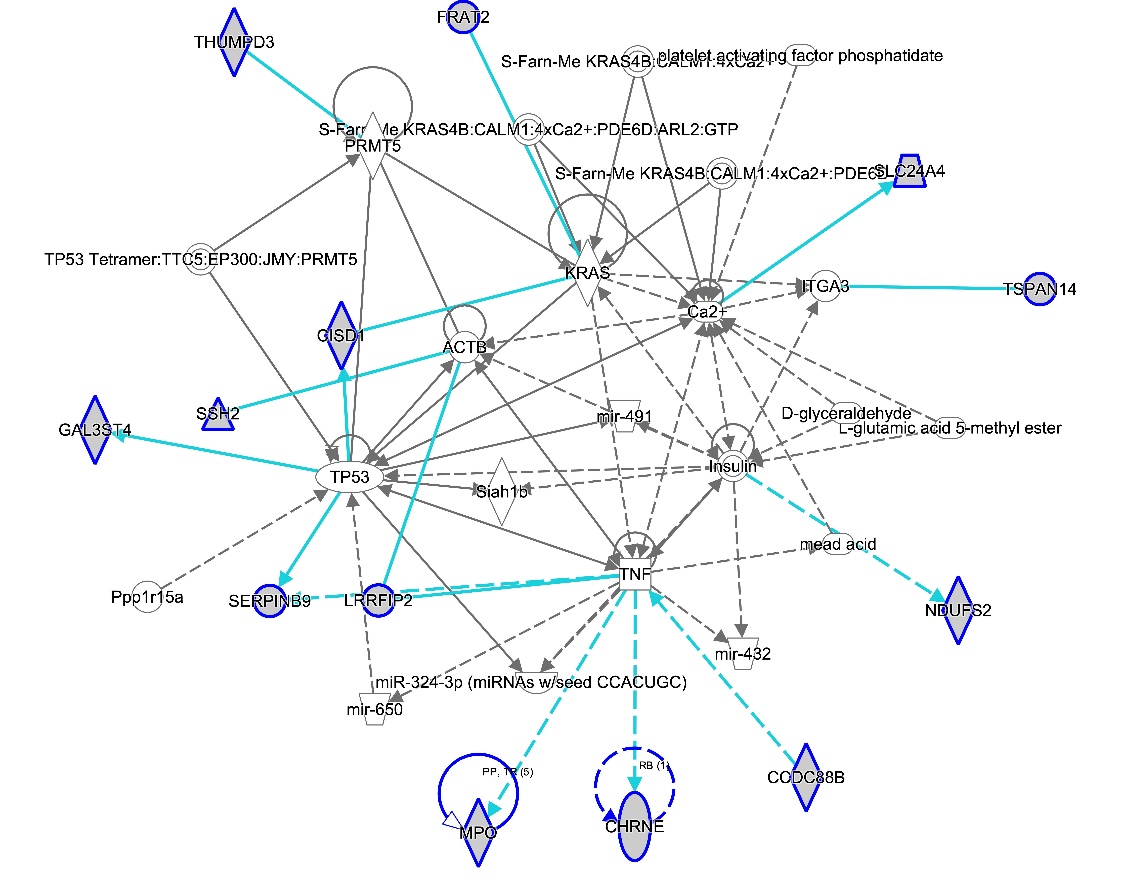
**

**Figure S4. Networks of the genes that consistent association directions for the methylation-gene expression-AD risk.**

1. Network 1: Metabolic Disease, Neurological Disease, Organismal Injury and Abnormalities
2. Network 2: Cellular Compromise, Inflammatory Response, Drug Metabolism

Gene from the Knowledge Base – not part of our MWAS identified genes for AD risk

Our MWAS identified genes for AD risk

direct interaction

indirect interaction

**Reference**

[1] Jansen IE, Savage JE, Watanabe K, Bryois J, Williams DM, Steinberg S*, et al.* Genome-wide meta-analysis identifies new loci and functional pathways influencing Alzheimer’s disease risk. *Nat Genet*. 2019;51:404-13.

[2] Lambert J-C, Ibrahim-Verbaas CA, Harold D, Naj AC, Sims R, Bellenguez C*, et al.* Meta-analysis of 74,046 individuals identifies 11 new susceptibility loci for Alzheimer's disease. *Nat Genet*. 2013;45:1452.

[3] Wightman DP, Jansen IE, Savage JE, Shadrin AA, Bahrami S, Holland D*, et al.* A genome-wide association study with 1,126,563 individuals identifies new risk loci for Alzheimer's disease. *Nat Genet*. 2021;53:1276-82.

[4] C. B, F K, IE J, L K, S M-G, N A*, et al.* New insights into the genetic etiology of Alzheimer's disease and related dementias. *Nat Genet*. 2022;54:412-36.
